# TRIM45 restricts influenza virus infection through modulating the chaperone-mediated autophagic degradation of viral PB2 protein

**DOI:** 10.1371/journal.ppat.1013630

**Published:** 2025-10-23

**Authors:** Yihan Wang, Li Jiang, Qibing Li, Mengya Li, Wenjun Shi, Bo Wang, Guangwen Wang, Guohua Deng, Jianzhong Shi, Guobin Tian, Xianying Zeng, Hualan Chen, Chengjun Li

**Affiliations:** State Key Laboratory for Animal Disease Control and Prevention, Harbin Veterinary Research Institute, Chinese Academy of Agricultural Sciences, Harbin, People’s Republic of China; Icahn School of Medicine at Mount Sinai, UNITED STATES OF AMERICA

## Abstract

The host defense system employs elaborate mechanisms to combat invading viruses. Here, we demonstrate that tripartite motif containing 45 (TRIM45) restricts the replication of different subtypes of influenza virus. TRIM45 interacted with and reduced the level of viral polymerase basic protein 2 (PB2). PB2 associated with heat shock cognate protein 70 (HSC70) and lysosomal-associated membrane protein type 2A (LAMP-2A), and was directed for lysosomal degradation via chaperone-mediated autophagy (CMA). TRIM45 promoted LAMP-2A expression and enhanced PB2/LAMP-2A binding, thereby facilitating CMA-dependent PB2 degradation. Mechanistically, TRIM45 employed its E3 ubiquitin ligase activity to mediate the K48-linked polyubiquitination and proteasomal degradation of Ca2 + -dependent cysteine protease calpain 1 (CAPN1), which prevented CAPN1-mediated cleavage of LAMP-2A. Sequence analysis identified a highly conserved QMRDV motif at position 602–606 of PB2, which was required for its binding with LAMP-2A or HSC70. Strikingly, mutations of this motif abolished this binding and the degradation effect of TRIM45 on PB2, and a PB2-Q602A mutant virus exhibited increased replication and enhanced pathogenicity in mice. Collectively, our findings reveal that TRIM45 restricts influenza virus infection by promoting the degradation of viral PB2 protein via CMA.

## Author summary

Autophagy is a powerful mechanism used by the host defense system to combat viral infections. However, whether CMA is involved in defending against influenza virus infection remains unknown. In this study, we found that TRIM45 inhibits the replication of influenza virus by reducing the level of viral PB2 protein. TRIM45 promoted the expression of the CMA receptor LAMP-2A and enhanced the interaction between PB2 and LAMP-2A, leading to the degradation of PB2 through the CMA pathway. Our investigation revealed that TRIM45 mediates the K48-linked polyubiquitination and proteasomal degradation of protease CAPN1, thereby blocking CAPN1-mediated cleavage of LAMP-2A. Importantly, we identified a KFERQ-like motif, _602_QMRDV_606_ in PB2, that is essential for the targeting and degradation of CMA substrates in lysosomes. Introduction of mutations into this motif abolished the degradation of PB2 via CMA pathway, with a PB2-Q602A mutant virus showing enhanced pathogenicity in mice. Together, our study revealed that the pathogenesis of influenza virus can be antagonized by the CMA pathway with the involvement of TRIM45.

## Introduction

Despite extensive prevention and control efforts, the threat posed by influenza A virus to the health of animals and humans remains substantial. The genome of influenza A virus is composed of eight single-stranded negative-sense RNA segments. The accumulation of genetic mutations and the exchange of gene segments among different virus strains enable influenza A virus to endlessly evolve. In addition to the threats posed by seasonal H1N1 and H3N2 influenza viruses, avian influenza viruses (e.g., H5N1, H5N6, H7N9, H9N2 and H3N8 subtypes) sporadically cross the species barrier to cause human infections and death [1–6], and have the potential to cause a new influenza pandemic. In the replication cycle of influenza virus, host cellular factors employ elaborate mechanisms to combat the virus infection. Gaining mechanistic insights on the interplay between influenza virus and the host defense system is important for the development of novel antiviral countermeasures.

Autophagy is a lysosome-dependent pathway involved in the delivery of cytosolic constituents into the lysosome for degradation and recycling. There are three main types of autophagy: macroautophagy, microautophagy, and chaperone-mediated autophagy (CMA) [7]. Macroautophagy involves the formation of autophagosomes, which fuse with endosomes or lysosomes to form autolysosomes, leading to the degradation of cytoplasmic cargos [8]. Microautophagy is the direct uptake and breakdown of cellular components by lysosomes [9]. By contrast, CMA is a highly selective autophagy mechanism that only performs lysosomal degradation for specific soluble proteins [10,11]. All CMA substrates contain a pentapeptide KFERQ motif or functional KFERQ-like motif in their amino acid sequences [12], which is recognized by the heat shock cognate protein 70 (HSC70) chaperone [13]. The substrate-chaperone complex binds the lysosomal-associated membrane protein type 2A (LAMP-2A) that serves as the specific receptor for autophagic components [10]. Association of the substrate with LAMP-2A triggers its multimerization, resulting in the translocation of the autophagic substrate into the lysosome. Finally, internalization of the substrate leads to its autophagic degradation in the lysosomal lumen [14]. The level of LAMP-2A is vulnerable to cellular proteases, e.g., Ca2 + -dependent cysteine protease calpain1 (CAPN1) [15], thereby affecting the function of the CMA pathway. Growing evidence suggests that macroautophagy is involved in the replication cycle of influenza virus. The M2, NS1, and HA proteins of influenza virus can initiate autophagosome formation and/or inhibit autolysosome and autophagic degradation in infected cells to benefit viral proliferation [16–20]. Meanwhile, influenza proteins can be targets of the macroautophagic degradation system [21], which is used by the host to defend against influenza virus infection. However, whether the CMA pathway is also involved in the pathogenesis of influenza virus remains unknown.

In a previous screen to identify tripartite motif (TRIM) proteins that play roles in the replication of influenza virus, we found that TRIM45, also known as RNF99, is a potential host restriction factor for virus growth [22]. However, the precise role of TRIM45 in the replication cycle of influenza virus remains unknown. Here, we revealed that TRIM45 promoted the expression of LAMP-2A and increased the interaction between influenza PB2 and LAMP-2A, leading to the degradation of PB2 through the CMA pathway. The E3 ubiquitin ligase activity of TRIM45 mediated the K48-linked polyubiquitination and proteasomal degradation of CAPN1, thereby preventing CAPN1-mediated cleavage of LAMP-2A. Of note, we identified a KFERQ-like motif in PB2. Mutation of this motif abolished the degradation of PB2 via the CMA pathway, resulting in increased growth of influenza virus in cell culture and enhanced pathogenicity in mice.

## Results

### TRIM45 negatively regulates the replication of different subtypes of influenza virus

**Fig 1:**
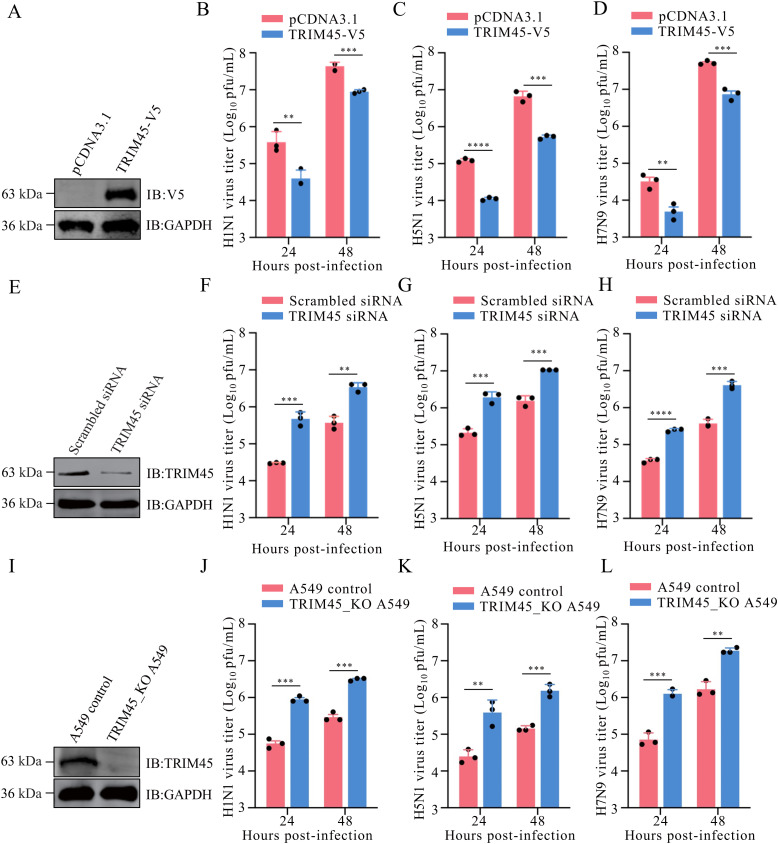
TRIM45 suppresses the replication of influenza virus. **(A)** TRIM45 protein levels in HEK293T cells transfected with plasmids expressing TRIM45-V5 or empty vector were detected by western blotting with a rabbit anti-V5 pAb. **(B-D)** HEK293T cells were transfected with plasmids expressing TRIM45-V5 or empty vector for 24 h, and then infected with WSN (H1N1) **(B)**, AH05 (H5N1) **(C)**, or AH13 (H7N9) **(D)** virus at an MOI of 0.1. Virus titers were measured by use of plaque assays at 24 and 48 h p.i. **(E)** TRIM45 protein levels in A549 cells transfected with siRNA targeting TRIM45 or scrambled siRNA for 48 h were detected by western blotting with a mouse anti-TRIM45 mAb. **(F-H)** A549 cells were transfected with siRNA targeting TRIM45 or scrambled siRNA for 24 h, and then infected with WSN (H1N1) **(F)**, AH05 (H5N1) **(G)**, or AH13 (H7N9) **(H)** virus at an MOI of 0.1. Virus titers were measured by using plaque assays at 24 and 48 h p.i. **(I)** Knockout of TRIM45 in TRIM45_KO A549 cells was confirmed by western blotting with a mouse anti-TRIM45 mAb. **(J-L)** TRIM45_KO or control A549 cells were infected with WSN (H1N1) **(J)**, AH05 (H5N1) **(K)**, or AH13 (H7N9) **(L)** virus at an MOI of 0.1. Virus titers were measured with plaque assays at 24 and 48 h p.i. For B-D, F-H, and J-L, error bars indicate SEMs calculated from three replicates. *n* = 3; two-tailed unpaired Student’s t-test.

To investigate the biological effect of TRIM45 on the replication of influenza virus, we transfected HEK293T cells with plasmids expressing TRIM45-V5, and confirmed the overexpression of TRIM45-V5 by western blotting at 48 h post-transfection (Fig 1A). The TRIM45-overexpressing cells were infected with A/WSN/1933 (WSN, H1N1), A/Anhui/2/2005 (AH05, H5N1), or A/Anhui/1/2013 (AH13, H7N9), and at 24 and 48 h post-infection (p.i.), the culture supernatant was titrated for infectious viruses. We found that the overexpression of TRIM45 reduced the growth titers of WSN (H1N1), AH05 (H5N1), and AH13 (H7N9) virus by 90.2%/79.7%, 90.9%/92.3%, and 84.6%/85.4%, respectively, at 24/48 h p.i. (Fig 1B–1D). Next, we analyzed the effect of siRNA-mediated knockdown of TRIM45 expression on the replication of influenza virus in A549 cells. TRIM45-specific siRNA effectively reduced TRIM45 expression (Fig 1E). The siRNA-treated cells were infected with WSN (H1N1) virus. Downregulation of TRIM45 expression increased the growth titer of WSN (H1N1), AH05 (H5N1), and AH13 (H7N9) virus by 15.7-/9.3-, 9.1-/6.9-, and 6.6-/10.8-fold, respectively, at 24/48 h p.i (Fig 1F–1H). To further verify the role of TRIM45 in influenza virus replication, we generated a TRIM45 knockout (TRIM45_KO) A549 cell line by using the CRISPR/Cas9 system (Fig 1I). The growth titers of WSN (H1N1), AH05 (H5N1), and AH13 (H7N9) virus were much higher in TRIM45_KO A549 cells than those in control cells at 24 and 48 h p.i. (Fig 1J–1L). Consistent with these data, in TRIM45 siRNA- versus scrambled siRNA-treated A549 cells or TRIM45_KO versus control A549 cells, more severe cytopathic lesions were observed at 48 h p.i. with WSN (H1N1) virus (S1A and S1B Fig). Moreover, we found that the level of TRIM45 was induced in A549 cells infected with WSN (H1N1) virus (S2A and S2B Fig).

Meanwhile, we examined the expression of a representative interferon-stimulated gene (ISG), MX1, in the absence or presence of exogenous treatment with interferon (IFN)-α or IFN-β, and also assessed the growth of an unrelated virus, recombinant vesicular stomatitis virus expressing EGFP (VSV-EGFP), in TRIM45_KO or control A549 cells. We found that the expression of MX1 was similarly induced by the exogenous treatment with IFN-α or IFN-β between TRIM45_KO and control A549 cells (S3A and S3B Fig), indicating the knockout of TRIM45 produces no effect on the IFN signaling pathway. In addition, no difference in the replication of VSV-EGFP was observed between TRIM45_KO and control A549 cells (S4 Fig), demonstrating that TRIM45 specifically exerts antiviral activity against influenza virus.

Collectively, these data indicate that TRIM45 is a potent host restriction factor for the replication of influenza virus.

### TRIM45 interacts with influenza PB2 protein

**Fig 2:**
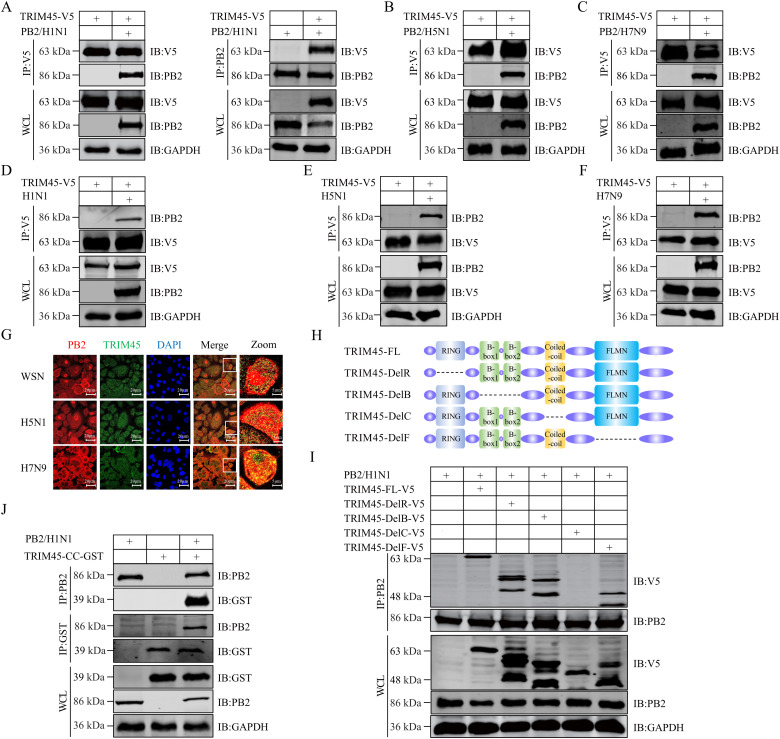
TRIM45 interacts with influenza PB2 protein. **(A-C)** HEK293T cells were transfected with the indicated plasmids for 24 **h.** Cell lysates were immunoprecipitated with a mouse anti-V5 or anti-PB2 mAb. Bound proteins were western blotted with a rabbit anti-V5 or anti-PB2 pAb. **(D-F)** HEK293T cells were transfected with plasmids expressing TRIM45-V5 for 12 h, and then infected with WSN (H1N1) **(D)**, AH05 (H5N1) **(E)**, or AH13 (H7N9) **(F)** virus at an MOI of 5 for 12 **h.** Cell lysates were immunoprecipitated with a mouse anti-V5 mAb. Bound proteins were western blotted with a rabbit anti-V5 or anti-PB2 pAb. **(G)** HEK293T cells were transfected with plasmids expressing TRIM45-V5 for 24 h, infected with WSN (H1N1), AH05 (H5N1), or AH13 (H7N9) virus (MOI = 5), and visualized by confocal microscopy at 12 h p.i. For clearer visualization, the DAPI staining was not shown in the Merge and Zoom panel. **(H)** Schematic representation of wild-type and truncation mutants of TRIM45. **(I)** HEK293T cells were transfected with the indicated plasmids for 24 **h.** Cell lysates were immunoprecipitated with a mouse anti-PB2 mAb. Bound proteins were western blotted with a rabbit anti-V5 or anti-PB2 pAb. **(J)** HEK293T cells were transfected with the indicated plasmids for 24 **h.** Cell lysates were immunoprecipitated with a mouse anti-PB2 or anti-GST mAb. Bound proteins were western blotted with a rabbit anti-GST or anti-PB2 pAb.

To explore the role of TRIM45 in the replication of influenza virus, we determined whether TRIM45 interacts with any influenza proteins. To this end, we performed Co-IP experiments in HEK293T cells that were transfected with plasmids expressing TRIM45-V5 or TRIM45-Flag, together with plasmids expressing one of the ten essential viral proteins (PB2, PB1, PA, HA, NP, NA, M1, M2, NS1, or NS2) of WSN (H1N1) virus. We found that among the ten essential influenza proteins, TRIM45 only interacted with PB2 (Figs 2A and S5A–S5J). When Co-IP experiments were performed to examine the interaction between TRIM45 and the PB2 of different subtypes of influenza virus, we found that TRIM45 interacted with PB2 of AH05 (H5N1) and AH13 (H7N9) virus in transfected cells (Fig 2B and 2C). We next performed the Co-IP experiment in HEK293T cells that were first transfected to express V5-tagged TRIM45 for 24 h, followed by infection with WSN (H1N1), AH05 (H5N1) or AH13 (H7N9) virus (MOI = 5) for 12 h. We found that TRIM45 also interacted with PB2 in the course of infection with these viruses (Fig 2D–2F). Meanwhile, we also conducted Co-IP experiments in A549 cells that were infected with WSN (H1N1), AH05 (H5N1) or AH13 (H7N9) virus, and found that PB2 interacted with endogenous TRIM45 (S6A–S6C Fig). Based on these findings, we investigated the localization of TRIM45 and PB2 in infected cells. HEK293T cells were first transfected with plasmids expressing TRIM45-V5. At 24 h post-transfection, the cells were infected with WSN (H1N1), AH05 (H5N1), or AH13 (H7N9) virus (MOI = 5), followed by confocal microscopy analysis at 12 h p.i. TRIM45 and PB2 co-localized in the cells infected with different virus strains (Figs 2G and S7A–S7C).

TRIM45 consists of a RING finger domain (residues 29–98), a B-box domain (residues 130–227), a coiled-coil (CC) domain (residues 281–335), and a filamin-type immunoglobulin (FLMN) domain (residues 394–497). To determine which domain of TRIM45 interacts with PB2, we constructed TRIM45 truncation mutants (Fig 2H), and assessed their interaction with PB2 in Co-IP experiments. We found that the TRIM45 mutant lacking the CC domain (TRIM45-DelC) lost the ability to interact with PB2 (Fig 2I). Furthermore, the CC domain of TRIM45 alone was sufficient for the interaction with PB2 (Fig 2J).

### TRIM45 reduces the expression level of influenza PB2 protein

**Fig 3:**
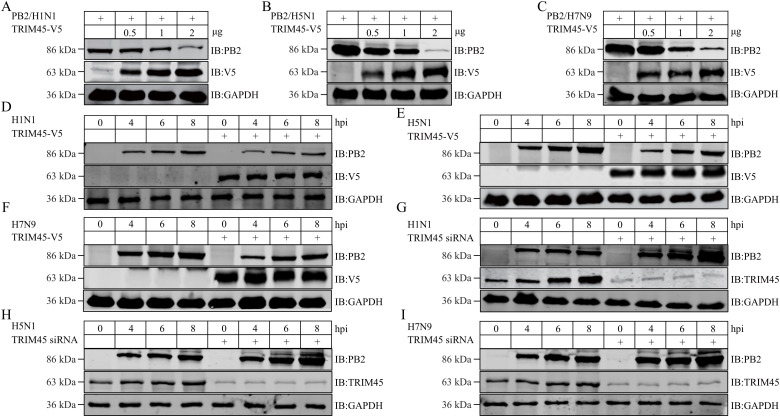
TRIM45 reduces the level of influenza PB2 protein. **(A-C)** HEK293T cells were transfected with plasmids expressing WSN (H1N1) PB2 **(A)**, AH05 (H5N1) PB2 **(B)**, or AH13 (H7N9) PB2 **(C)**, together with gradually increasing quantities of TRIM45-V5-expressing plasmids. At 24 h post-transfection, the level of PB2 protein was determined by western blotting with a mouse anti-PB2 mAb. **(D-F)** HEK293T cells were transfected with plasmids expressing TRIM45-V5 or empty vector for 24 h, and were then infected with WSN (H1N1) **(D)**, AH05 (H5N1) **(E)**, or AH13 (H7N9) **(F)** virus (MOI = 5). At 0, 4, 6, and 8 h p.i., cell lysates were western blotted with a rabbit anti-V5 or anti-PB2 pAb. (**G-I**) A549 cells were transfected with siRNA targeting TRIM45 or scrambled siRNA for 36 h, and then infected with WSN (H1N1) **(G)**, AH05 (H5N1) **(H)**, or AH13 (H7N9) **(I)** virus (MOI = 5). At 0, 4, 6, and 8 h p.i., cell lysates were western blotted with a rabbit anti-TRIM45 or anti-PB2 pAb.

TRIM proteins are usually involved in the degradation of target proteins through their ubiquitin E3 ligase activity [23]. Therefore, we asked whether TRIM45 affects the expression of the PB2 protein. HEK293T cells were transfected with plasmids expressing WSN PB2, together with gradually increasing amounts of TRIM45-V5-expressing plasmids. At 24 h post-transfection, the level of PB2 protein was determined by western blotting. We found that increasing amounts of TRIM45 reduced the expression level of WSN PB2 in a dose-dependent manner (Fig 3A). Moreover, this effect of TRIM45 on the expression of WSN PB2 in transfected cells was also observed for the PB2 of AH05 (H5N1) and AH13 (H7N9) virus (Fig 3B and 3C).

We then determined the effect of TRIM45 on the expression of PB2 during influenza infection. HEK293T cells were transfected with plasmids expressing TRIM45-V5 or empty vector. At 24 h post-transfection, the cells were infected with WSN (H1N1), AH05 (H5N1), or AH13 (H7N9) virus. At 0, 4, 6, and 8 h p.i., the expression level of the PB2 protein was determined by western blotting. We found that the presence of exogenously expressed TRIM45 reduced the level of PB2 in cells infected with any of the three subtypes of influenza virus (Fig 3D–3F).

Next, we analyzed the effect of siRNA-mediated knockdown of TRIM45 on the expression level of influenza PB2 by western blotting. We found that at 4, 6, and 8 h p.i., the levels of the PB2 of WSN (H1N1), AH05 (H5N1), and AH13 (H7N9) virus were increased in TRIM45 siRNA-treated cells compared with those of scrambled siRNA-treated cells (Fig 3G-3I).

Furthermore, we examined whether murine TRIM45 has an effect on the replication of WSN (H1N1) virus as well as the level of viral PB2 protein. As shown in S8A Fig, the growth titers of WSN (H1N1) virus were reduced by 85.9% and 81.7% at 24 and 48 h p.i. in murine lung epithelial-12 (MLE12) cells overexpressing murine TRIM45 compared with control MLE12 cells. Meanwhile, the overexpression of murine TRIM45 led to obvious reduction of viral PB2 protein at 4, 6, and 8 h p.i. in MLE12 cells (S8B Fig). These results indicate that similar to human TRIM45, murine TRIM45 also functions to inhibit the replication of influenza virus by impairing the expression of viral PB2 protein.

Given that PB2 is a component of the viral ribonucleoprotein complex (vRNP), we examined the effect of TRIM45-mediated downregulation of PB2 expression on vRNP complex activity. HEK293T cells were transfected with minireplicon plasmids of influenza virus and different amounts of plasmids expressing TRIM45. We found that the expression of TRIM45 inhibited the vRNP complex activity in a dose-dependent manner (S9 Fig).

Together, these data indicate that TRIM45 downregulates the expression of influenza PB2, thereby impairing the vRNP complex activity.

### TRIM45 degrades influenza PB2 through the lysosomal degradation pathway

**Fig 4:**
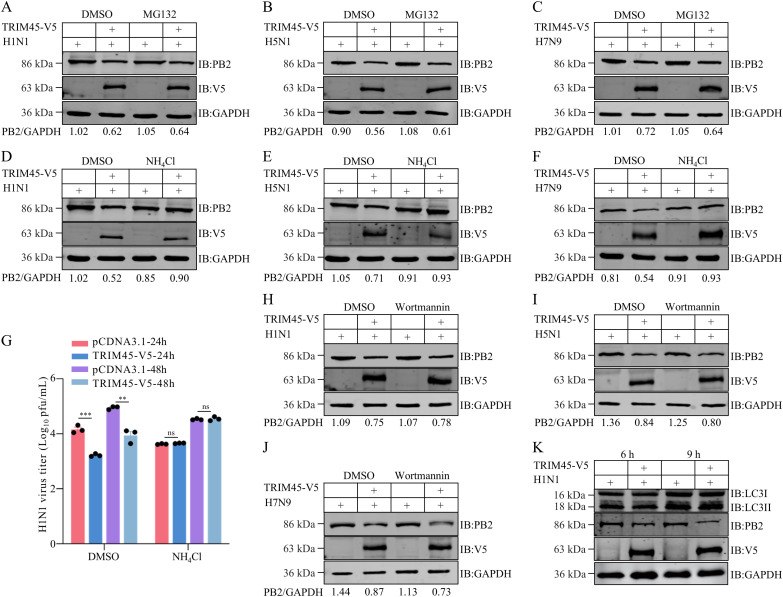
TRIM45 degrades PB2 protein through the lysosomal pathway. **(A-C)** HEK293T cells were transfected with plasmids expressing TRIM45-V5 or empty vector for 24 h, and then infected with WSN (H1N1) **(A)**, AH05 (H5N1) **(B)**, or AH13 (H7N9) **(C)** virus (MOI = 5). At 16 h p.i., the cells were treated with DMSO or MG132 (10 μg/mL) for 8 h, and cell lysates were western blotted with a rabbit anti-V5 or anti-PB2 pAb. **(D-F)** HEK293T cells were transfected with plasmids expressing TRIM45-V5 or empty vector for 24 h, and then infected with WSN (H1N1) **(D)**, AH05 (H5N1) **(E)**, or AH13 (H7N9) **(F)** virus (MOI = 5). At 16 h p.i., the cells were treated with DMSO or NH_4_Cl (5 mM) for 8 h, and cell lysates were western blotted with a rabbit anti-V5 or anti-PB2 pAb. **(G)** HEK293T cells were transfected with plasmids expressing TRIM45-V5 or empty vector for 12 h, and then infected with WSN (H1N1) virus (MOI = 0.1) in the presence of DMSO or NH_4_Cl. Virus titers were measured by use of plaque assays at 24 and 48 h p.i. **(H-J)** HEK293T cells were transfected with plasmids expressing TRIM45-V5 or empty vector for 24 h, and then infected with WSN (H1N1) **(H)**, AH05 (H5N1) **(I)**, or AH13 (H7N9) **(J)** virus (MOI = 5). At 16 h p.i., the cells were treated with DMSO or Wortmannin (100 nM) for 8 h, and cell lysates were western blotted with a rabbit anti-V5 or anti-PB2 pAb. **(K)** HEK293T cells were transfected with plasmids expressing TRIM45-V5 or empty vector for 24 h, and then infected with WSN (H1N1) virus (MOI = 5). At 6 and 9 h p.i., cell lysates were western blotted with a rabbit anti-LC3I, anti-LC3II, anti-V5, or anti-PB2 pAb. For A-F and H-J, the band intensities of PB2, quantified by using ImageJ software (1.53k), were normalized to GAPDH. For G, error bars indicate SEMs calculated from three replicates. *n* = 3; two-tailed unpaired Student’s t-test.

TRIM45 is an established ubiquitin E3 ligase [24]. To determine whether TRIM45 mediates PB2 degradation through directly targeting PB2 into the proteasomal degradation pathway, we examined the effect of TRIM45 on the stability of PB2 in the presence of the proteasome inhibitor MG132. We found that the suppressing effect of TRIM45 on the expression of PB2 of WSN (H1N1), AH05 (H5N1), and AH13 (H7N9) virus was not disrupted by MG132 (Fig 4A–4C), indicating that the downregulating effect of TRIM45 on the expression of influenza PB2 is not achieved through directly targeting PB2 into the proteasomal degradation pathway.

The autophagy lysosomal pathway is also a major mechanism for degrading intracellular macromolecules. Therefore, we examined the effect of TRIM45 on the level of influenza PB2 by introducing NH_4_Cl, an inhibitor of the autophagy lysosomal pathway, which is known to neutralize the acidic lysosomal pH. In the presence of NH_4_Cl, TRIM45 lost its ability to downregulate the expression of the PB2 of WSN (H1N1), AH05 (H5N1), or AH13 (H7N9) virus (Fig 4D–4F), indicating that TRIM45 degrades influenza PB2 through the lysosomal degradation pathway. Furthermore, the inhibitory effect of TRIM45 on the replication of WSN (H1N1) virus was abolished in the presence of NH_4_Cl (Fig 4G).

The autophagy lysosomal degradation pathway has three different degradation modes [25], macroautophagy, microautophagy, and CMA [26]. Macroautophagy and CMA occur most extensively. Therefore, we examined separately the role of these two autophagic pathways in the degradation of influenza PB2. First, the potential role of macroautophagy pathway was examined by introducing Wortmannin, an inhibitor of macroautophagy, and rapamycin (RAP), an accelerator of macroautophagy. We found that the degradative effect of TRIM45 on influenza PB2 was not affected by Wortmannin (Fig 4H–4J) or RAP (S10 Fig), indicating that TRIM45 does not degrade PB2 through the macroautophagy pathway. Next, we detected the expression of LC3-II, an autophagosomal marker that reflects macroautophagic activity [27], in an experiment to examine the effect of TRIM45 on the stability of influenza PB2. We found that there was no difference in the level of LC3-II between cells transfected with TRIM45-expressing plasmid or empty vector under the condition of WSN (H1N1) virus infection (Fig 4K). These data confirmed that in the process of degrading influenza PB2, TRIM45 does not employ the macroautophagy pathway.

### Influenza PB2 interacts with the chaperone HSC70 and lysosomal receptor LAMP-2A of the CMA pathway

**Fig 5:**
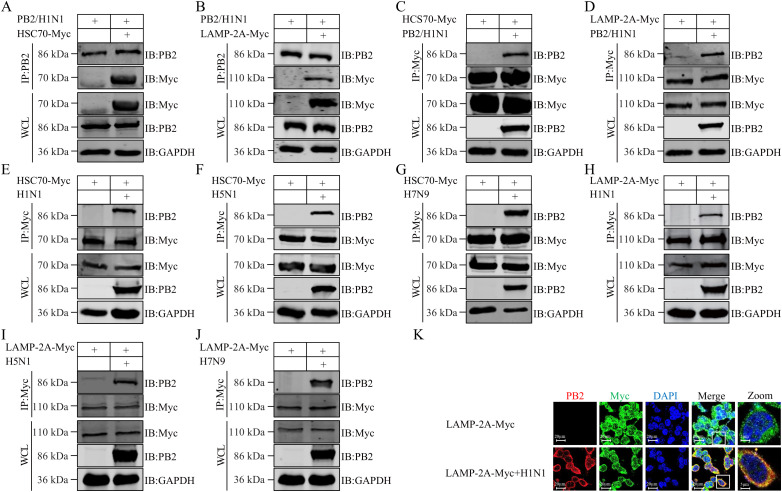
TRIM45 interacts with HSC70 and LAMP-2A of the CMA pathway. **(A-D)** HEK293T cells were transfected with the indicated plasmids for 24 **h.** Cell lysates were immunoprecipitated with a mouse anti-PB2 or anti-Myc mAb. Bound proteins were western blotted with a rabbit anti-PB2 or anti-Myc pAb. **(E-J)** HEK293T cells were transfected with plasmids expressing HSC70-Myc **(E-G)** or LAMP-2A-Myc **(H-J)** for 24 h, and then infected with WSN (H1N1) **(E, H)**, AH05 (H5N1) **(F, I)**, or AH13 (H7N9) **(G, J)** virus (MOI = 5) for 12 **h.** Cell lysates were immunoprecipitated with a mouse anti-Myc mAb. Bound proteins were western blotted with a rabbit anti-Myc or anti-PB2 pAb. **(K)** HEK293 cells were transfected with plasmids expressing Myc-tagged LAMP-2A for 24 h, and left uninfected or were infected with WSN (H1N1) virus (MOI = 5). The localization of PB2 and LAMP-2A-Myc was visualized by use of confocal microscopy at 12 h p.i.

After excluding the macroautophagy pathway, we examined the potential role of the CMA pathway in TRIM45-mediated degradation of influenza PB2. CMA delivers selected protein substrates into lysosomes for degradation, mediated by their interaction with the chaperone HSC70 and binding with LAMP-2A [28]. We therefore assessed whether influenza PB2 interacts with HSC70 and LAMP-2A by using a Co-IP assay in HEK293T cells transfected with plasmids expressing WSN (H1N1) PB2 and Myc-tagged HSC70 or LAMP-2A. We found that both HSC70 and LAMP-2A interact with the PB2 protein of WSN (H1N1) virus (Fig 5A–5D). This interaction was verified in HEK293T cells transfected with Myc-tagged HSC70 or LAMP-2A and subsequently infected with WSN (H1N1), AH05 (H5N1), or AH13 (H7N9) virus (Fig 5E–5J). In addition, we performed a confocal microscopy assay in HEK293 cells transfected with plasmids expressing Myc-tagged LAMP-2A, and then infected with WSN (H1N1) virus (MOI = 5) at 12 h post-transfection. The PB2 of WSN (H1N1) virus clearly colocalized with LAMP-2A at 12 h p.i. (Figs 5K and S11). Together, these results suggest that TRIM45 most likely degrades influenza PB2 through the CMA pathway.

### The QMRDV motif of influenza PB2 is a key motif for CMA degradation

**Fig 6:**
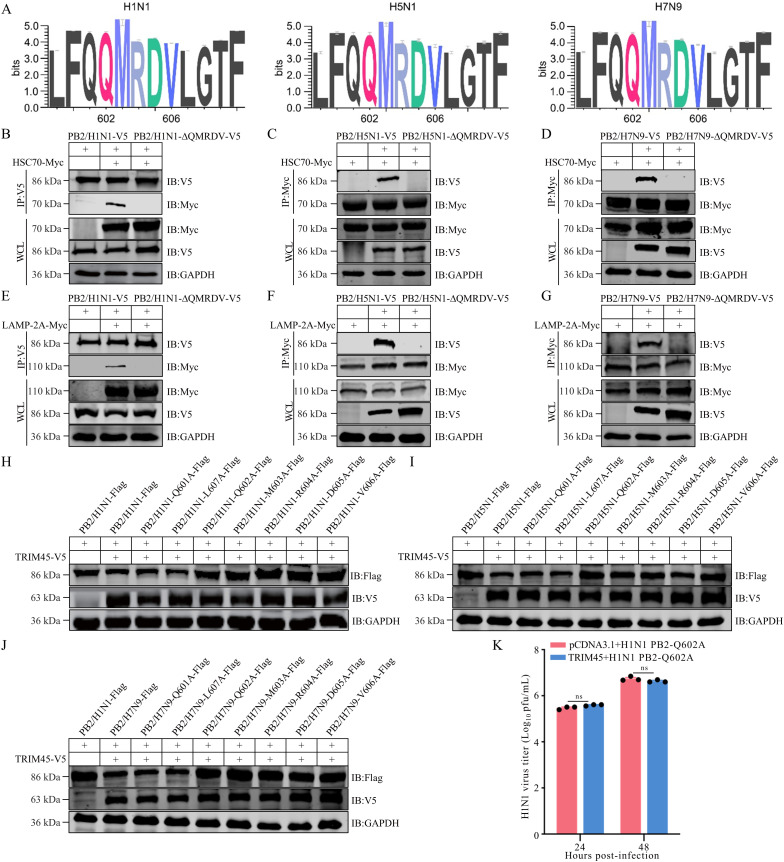
TRIM45 degrades PB2 protein through the 602–606 QMRDV motif. **(A)** Sequence analysis to identify a KFERQ-like motif at positions 602–606 of the influenza PB2 protein. The data for the logos consist of 62617, 457, and 1354 sequences from the PB2 alignment of human H1N1, H5N1, and H7N9 viruses, respectively. **(B-G)** HEK293T cells were transfected with plasmids expressing HSC70-Myc or LAMP-2A-Myc, together with the V5-tagged wild-type PB2 or QMRDV-deleted PB2 of WSN (H1N1) **(B, E)**, AH05 (H5N1) **(C, F)**, or AH13 (H7N9) **(D, G)** virus. At 24 h post-transfection, cell lysates were immunoprecipitated with a mouse anti-V5 or anti-Myc mAb. Bound proteins were western blotted with a rabbit anti-V5 or anti-Myc pAb. **(H-J)** HEK293T cells were transfected with plasmids expressing TRIM45-V5, together with Flag-tagged wild-type PB2 or point mutant PB2 of WSN (H1N1) **(H)**, AH05 (H5N1) **(I)**, or AH13 (H7N9) **(J)** virus for 12 **h.** Cell lysates were western blotted with a rabbit anti-Flag or anti-V5 pAb. **(K)** HEK293 cells were transfected with plasmids expressing TRIM45 or empty vector for 24 h, and then infected with WSN (H1N1) PB2-Q602A mutant virus at an MOI of 0.1. Virus titers were measured by performing plaque assays at 24 and 48 h p.i. For K, error bars indicate SEMs calculated from three replicates. *n* = 3; two-tailed unpaired Student’s t-test.

The degradation of proteins through the CMA pathway requires that the target proteins contain a KFERQ-like motif, which is essential for the subsequent targeting and degradation of the substrate proteins in lysosomes [29,30]. After analyzing the PB2 sequences of human H1N1, H5N1, and H7N9 viruses, we found that a KFERQ-like motif, QMRDV, at positions 602–606, was highly conserved in PB2 among different subtypes of influenza virus (Fig 6A). We then determined whether this QMRDV motif is responsible for mediating the interaction between influenza PB2 and HSC70 and LAMP-2A by using a Co-IP assay in HEK293T cells that were transfected individually or in combination with plasmids expressing QMRDV-deficient PB2 protein from different virus strains, and Myc-tagged HSC70 or LAMP-2A. We found that when the pentapeptide QMRDV motif was deleted, the mutant PB2 protein could no longer interact with either HSC70 or LAMP-2A (Fig 6B–6G). Based on these findings, we further examined the degradative effect of TRIM45 on wild-type PB2 or mutant PB2 lacking the QMRDV motif in the absence or presence of NH_4_Cl. We found that the overexpression of TRIM45-V5 led to the degradation of Flag-tagged wild-type PB2, whereas the further treatment with NH_4_Cl restored the level of PB2 by blocking the lysosomal degradation pathway to inhibit TRIM45-mediated degradative effect. By contrast, the Flag-tagged PB2 mutant lacking the QMRDV motif was resistant to TRIM45-mediated degradation irrespective of the treatment of NH_4_Cl (S12A-S12C Fig). These data indicate that TRIM45 indeed mediates the degradation of influenza PB2 through the CMA pathway.

Next, we introduced individual alanine mutations into the five amino acids of the 602–606 QMRDV motif as well as two adjacent amino acids at positions 601 and 607 of PB2 to identify the key residue in PB2 that is important for TRIM45-mediated degradation. The results showed that none of the five PB2 mutants bearing single mutations in the QMRDV motif could be degraded by TRIM45; by contrast, TRIM45 could still degrade the two PB2 mutants bearing mutations at positions 601 or 607 (Fig 6H–6J). These data demonstrate that the QMRDV motif of influenza PB2 is essential for TRIM45-mediated degradation through the CMA pathway and that all five amino acid residues of the QMRDV motif are essential to this process.

To further determine the importance of the QMRDV motif in TRIM45-mediated restriction of influenza virus replication, HEK293 cells were transfected with plasmids expressing TRIM45 or empty vector, and then infected with a PB2-Q602A mutant WSN (H1N1) virus (MOI = 0.1). The PB2-Q602A mutant WSN (H1N1) virus grew to similar titers in cells transfected with empty vector or TRIM45-expressing plasmids (Fig 6K). Meanwhile, we complemented wild-type TRIM45 or RING domain-deleted TRIM45 mutant in TRIM45_KO A549 cells, and then assessed the replication of wild-type or PB2-Q602A mutant of WSN (H1N1) virus as well as PB2 degradation. We found that the complement of wild-type TRIM45 led to more remarkable degradation of wild-type PB2 compared with the RING domain-deleted TRIM45 mutant. By contrast, the stability of Q602A mutant PB2 was unaffected by either of the two forms of TRIM45 (S13A Fig). Consistent with these data, the growth of wild-type WSN (H1N1) virus was more obviously inhibited by the complement of wild-type TRIM45 compared with RING domain-deleted TRIM45 mutant, whereas the replication of PB2-Q602A mutant virus was unaffected by either of the two forms of TRIM45 (S13B Fig). These findings indicate that the TRIM45-mediated degradation of influenza PB2, attributed to the presence of the QMRDV motif in PB2, is required for TRIM45 to restrict the replication of influenza virus.

### TRIM45 enhances the expression of LAMP-2A and its interaction with PB2

**Fig 7:**
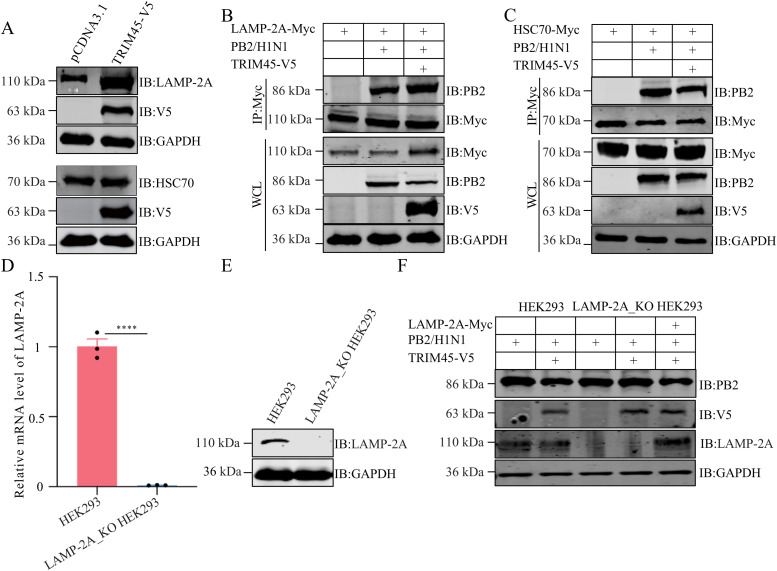
TRIM45 enhances LAMP-2A expression and the PB2-LAMP-2A interaction. **(A)** HEK293T cells were transfected with plasmids expressing TRIM45-V5 or empty vector for 24 h, and cell lysates were western blotted with a rabbit anti-V5 pAb and a rabbit anti-LAMP-2A or anti-HSC70 pAb. **(B, C)** HEK293T cells were transfected with the indicated plasmids for 24 **h.** Cell lysates were immunoprecipitated with a mouse anti-Myc mAb. Bound proteins were western blotted with a rabbit anti-Myc or anti-PB2 pAb. **(D)** LAMP-2A mRNA levels in LAMP-2A_KO or control HEK293 cells were quantified by use of RT-qPCR. Results are shown as relative values, with the LAMP-2A mRNA level of HEK293 control cells set as 1. **(E)** LAMP-2A protein levels in LAMP-2A_KO or control HEK293 cells were detected by western blotting. **(F)** The LAMP-2A_KO or control HEK293 cells were transfected with the indicated plasmids expressing WSN (H1N1) PB2, TRIM45-V5, LAMP-2A-Myc or empty vector for 24 **h.** Cell lysates were western blotted with a rabbit anti-V5, anti-PB2 or anti-LAMP-2A pAb. For D, error bars indicate SEMs calculated from three replicates. *n* = 3; two-tailed unpaired Student’s t-test.

To determine the role of TRIM45 in the CMA-mediated degradation of PB2, we examined the effect of TRIM45 on the expression of LAMP-2A and HSC70, the two essential mediators of the CMA pathway. The exogenous expression of TRIM45-V5 in HEK293T cells markedly enhanced the level of endogenous LAMP-2A, but had no effect on the level of endogenous HSC70 (Fig 7A). We next performed Co-IP experiments to investigate the impact of TRIM45 on the interaction between influenza PB2 and LAMP-2A or HSC70. To this end, HEK293T cells were co-transfected with different combinations of plasmids expressing TRIM45-V5, PB2, and LAMP-2A-Myc or HSC70-Myc. We found that the co-expression of TRIM45-V5 increased the level of LAMP-2A-Myc, and enhanced its interaction with PB2 (Fig 7B). By contrast, the expression of HSC70-Myc as well as its interaction with PB2 was not enhanced by the co-expression of TRIM45-V5 (Fig 7C). Furthermore, the co-expression of TRIM45-V5 and PB2 in HEK293T cells also enhanced the level of endogenous LAMP-2A as well as its interaction with PB2 (S14 Fig). Together, these results indicate that TRIM45 promotes the expression of LAMP-2A and its interaction with PB2, thereby directing PB2 into the CMA degradation pathway.

To validate the role of the CMA pathway in the degradation of influenza PB2, we generated a LAMP-2A_KO HEK293 cell line (Fig 7D and 7E). A functional rescue experiment was conducted by reintroducing LAMP-2A into LAMP-2A_KO HEK293 cells to assess its impact on PB2 stability. As shown in Fig 7F, TRIM45-mediated degradation of WSN (H1N1) PB2 was abolished in LAMP-2A_KO HEK293 cells, whereas reintroduction of LAMP-2A into the LAMP-2A_KO cells restored the ability of TRIM45 to degrade PB2 (Fig 7F), indicating that an intact CMA pathway is required for TRIM45 to mediate the degradation of influenza PB2.

### TRIM45 subverts CAPN1-mediated cleavage of LAMP-2A

**Fig 8:**
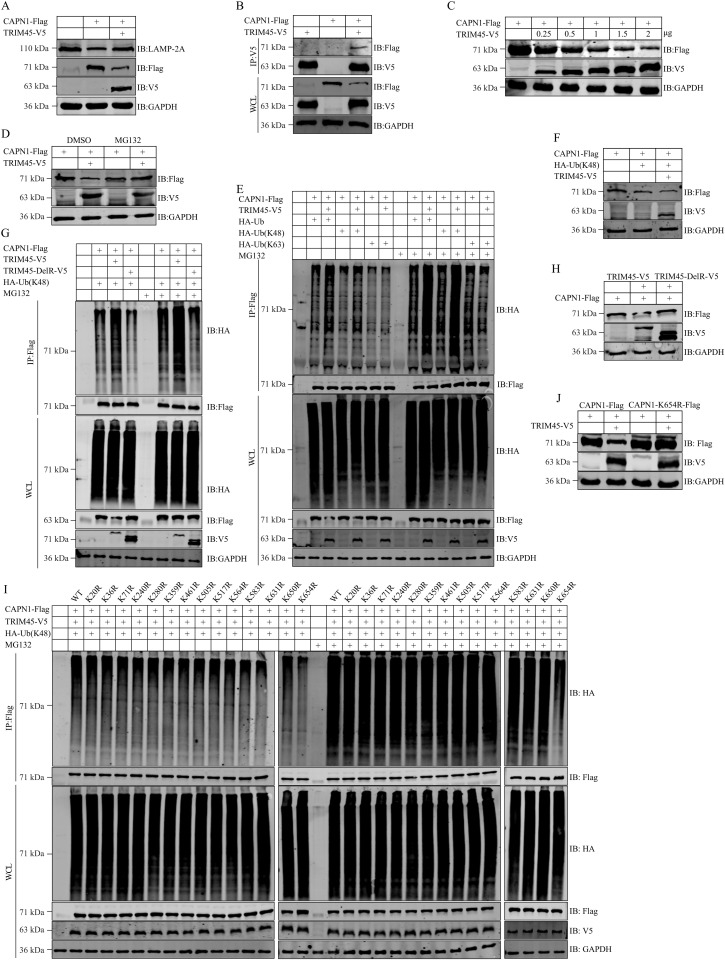
TRIM45 targets CAPN1 for K48-linked polyubiquitination and proteasomal degradation. **(A)** HEK293T cells were transfected with the indicated combinations of plasmids for 24 **h.** Cell lysates were western blotted with a rabbit anti-Flag, anti-V5 or anti-LAMP-2A pAb. **(B)** HEK293T cells were transfected as indicated for 24 **h.** Cell lysates were immunoprecipitated with a mouse anti-V5 mAb. Bound proteins were western blotted with a rabbit anti-Flag or anti-V5 pAb. **(C)** HEK293T cells were transfected as indicated for 24 **h.** Cell lysates were western blotted with a rabbit anti-Flag or anti-V5 pAb. **(D)** HEK293T cells were transfected with the indicated combinations of plasmids for 16 **h.** The cells were treated with DMSO or MG132 for 8 h, followed by western blotting with a rabbit anti-Flag or anti-V5 pAb. **(E)** HEK293T cells were transfected as indicated for 16 h, and then treated with DMSO or MG132 for 8 **h.** Cell lysates were immunoprecipitated with a mouse anti-Flag mAb, and the bound proteins were western blotted with a rabbit anti-Flag or anti-HA pAb. **(F)** HEK293T cells were transfected with the indicated combinations of plasmids for 24 **h.** Cell lysates were western blotted with a rabbit anti-Flag or anti-V5 pAb. **(G)** HEK293T cells were transfected with the indicated combinations of plasmids expressing CAPN1-Flag, TRIM45-V5, V5-tagged RING domain-deleted TRIM45 (TRIM45-DelR), and HA-Ub(K48) for 16 h, and then treated with DMSO or MG132 for 8 **h.** Cell lysates were immunoprecipitated with a mouse anti-Flag mAb, and the bound proteins were western blotted with a rabbit anti-Flag or anti-HA pAb. **(H)** HEK293T cells were transfected as indicated for 24 **h.** Cell lysates were western blotted with a rabbit anti-Flag or anti-V5 pAb. **(I)** HEK293T cells were transfected with the indicated combinations of plasmids expressing Flag-tagged wild-type or mutant CAPN1, TRIM45-V5, and HA-Ub(K48) for 16 h, and then treated with DMSO or MG132 for 8 **h.** Cell lysates were immunoprecipitated with a mouse anti-Flag mAb, and the bound proteins were western blotted with a rabbit anti-Flag or anti-HA pAb. **(J)** HEK293T cells were transfected with the indicated combinations of plasmids for 24 **h.** Cell lysates were western blotted with a rabbit anti-Flag or anti-V5 pAb.

It has previously been reported that CAPN1 cleaves LAMP-2A to induce lysosomal permeabilization [15]. Given that TRIM45 enhances the expression of LAMP-2A, we asked whether TRIM45 modulates the expression of LAMP-2A by influencing the role of CAPN1. To this end, HEK293T cells were transfected with the indicated combinations of plasmids expressing CAPN1-Flag and TRIM45-V5. At 24 h post-transfection, the endogenous level of LAMP-2A was reduced in cells expressing CAPN1 alone, whereas the complement of TRIM45-V5 largely abolished the CAPN1-mediated reduction of LAMP-2A level (Fig 8A). Subsequently, we transfected A549 cells with CAPN1 siRNA or scrambled siRNA for 36 h, followed by infection with WSN (H1N1) virus. At 12 h p.i., the cell lysates were subjected to western blotting to detect the levels of PB2 and LAMP-2A proteins. We found that the knockdown of CAPN1 led to an elevated level of LAMP-2A as well as the degradation of PB2 (S15 Fig).

To explore the interplay between CAPN1 and TRIM45 in regulating the expression of LAMP-2A, we performed Co-IP experiments in HEK293T cells to examine whether CAPN1 associates with TRIM45. The results showed that CAPN1 interacts with TRIM45 when they are co-expressed (Fig 8B).

TRIM proteins are often involved in post-translational modification and degradation of target proteins through their ubiquitin E3 ligase activity. We therefore investigated whether TRIM45 could degrade CAPN1. Co-expression of gradually increasing amounts of TRIM45-V5 reduced the level of CAPN1-Flag in a dose-dependent manner (Fig 8C). Consistent with this data, we found that TRIM45_KO A549 cells exhibited an increased level of endogenous CAPN1 alongside a reduced level of endogenous LAMP-2A (S16 Fig). We speculated that the ubiquitin E3 ligase activity of TRIM45 may mediate the ubiquitination of CAPN1 and direct ubiquitinated CAPN1 into the proteasomal degradation pathway. To investigate this possibility, HEK293T cells were transfected with constructs to express CAPN1 alone or in combination with TRIM45, and MG132 was added to inhibit protein degradation through the proteasomal pathway. We found that the addition of MG132 prevented TRIM45-mediated degradation of CAPN1 (Fig 8D), indicating that TRIM45 indeed mediates the degradation of CAPN1 through the proteasomal pathway. We then investigated the type of CAPN1 polyubiquitination that is catalyzed by TRIM45. By replacing ubiquitin (Ub) with its K48 (all lysine residues except K48 were mutated to arginine) or K63 (all lysine residues except K63 were mutated to arginine) mutant in the Co-IP experiment, we found that TRIM45 mediated K48-linked polyubiquitination of CAPN1 but had no role in K63-linked CAPN1 polyubiquitination. Of note, the MG132-treated group exhibited a significantly stronger ubiquitination signal intensity in comparison to the DMSO control group (Fig 8E). When HEK293T cells were transfected with different combinations of plasmids to express CAPN1, TRIM45, and Ub(K48) mutant, we found that the co-expression of TRIM45 and Ub(K48) mutant markedly promoted the degradation of CAPN1 (Fig 8F). To further determine whether the E3 ligase activity of TRIM45 is essential for the K48-linked polyubiquitination of CAPN1, CAPN1, Ub(K48) mutant, and TRIM45 or its RING domain-deleted (DelR) mutant were expressed in HEK293T cells. TRIM45 increased the levels of K48-linked polyubiquitination of CAPN1, whereas the DelR TRIM45 mutant lost the ability to catalyze K48-linked polyubiquitination of CAPN1 (Fig 8G). We also assessed whether CAPN1 is ubiquitinated by TRIM45 with endogenous ubiquitin in HEK293T cells co-transfected with plasmids expressing CAPN1-Flag and TRIM45-V5, and found that by utilizing endogenous ubiquitin, TRIM45 specifically enhanced K48-linked polyubiquitination of CAPN1 while exerting no effect on K63-linked polyubiquitination (S17A and S17B Fig). Consistent with these data, we found that compared with wild-type TRIM45, the DelR TRIM45 mutant failed to mediate K48-linked polyubiquitination of CAPN1 utilizing endogenous ubiquitin (S17C Fig), and to reduce the expression level of CAPN1 (Fig 8H), confirming that TRIM45 employs its ubiquitin E3 ligase activity to promote the K48-linked polyubiquitination and proteasomal degradation of CAPN1.

Ubiquitination involves the attachment of ubiquitin to acceptor lysine residues on substrate proteins. Fourteen lysine residues are predicted to be conserved in CAPN1. To identify the specific lysine residues of CAPN1 ubiquitinated by TRIM45, we generated CAPN1 mutants containing individual lysine-to-arginine mutations. We found that the ubiquitination level of the CAPN1-K654R mutant was reduced compared with that of wild-type CAPN1 (Fig 8I), indicating that residue K654 is the major ubiquitination site on CAPN1. As expected, the CAPN1-K654R mutant was not degraded by co-expressed TRIM45 (Fig 8J).

Together, these results reveal that TRIM45 mediates K48-linked polyubiquitination and proteasomal degradation of CAPN1, which abrogates CAPN1-mediated cleavage of LAMP-2A, thereby promoting the degradation of influenza PB2 through the CMA pathway.

### CMA-mediated degradation of PB2 limits influenza virus infection in mice

**Fig 9:**
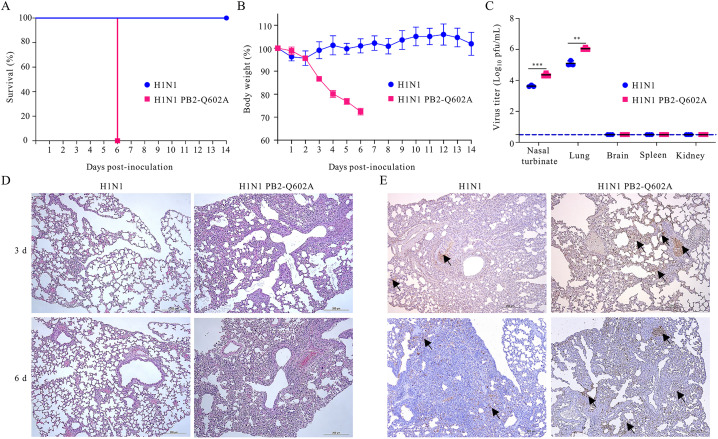
CMA-mediated degradation of PB2 limits influenza infection in mice. **(A-E)** Groups of 6-week-old female C57BL/6J mice were intranasally infected with 2 × 10^3^ PFU of wild-type or PB2-Q602A mutant WSN (H1N1) virus. The survival **(A)** and body weight changes **(B)** of the mice were monitored daily for 14 days. Virus titers in organ homogenates of mice were determined by performing plaque assay on Day 3 p.i. **(C)**. Lung sections prepared on Days 3 and 6 p.i. were stained with hematoxylin-and-eosin **(D)** or immunohistochemically stained with a mouse anti-NP mAb **(E)**. For B and C, error bars indicate SEMs calculated from five **(B)** or three **(C)** mice. *n* = 5 (A, B) or *n* = 3 **(C)**; two-tailed unpaired Student’s t-test.

To explore the role of the CMA-mediated degradation of PB2 in the pathogenicity of influenza virus in vivo, 6-week-old female C57BL/6J mice were intranasally infected with 2 × 10^3^ plaque-forming unit (PFU) of wild-type or PB2-Q602A mutant WSN (H1N1) virus, and their survival and body weight changes were monitored daily for 14 days. All five mice infected with the wild-type virus survived the infection. In contrast, 100% of mice infected with the PB2-Q602A mutant WSN (H1N1) virus died of their infection on Day 6 (Fig 9A). In addition, the mice infected with the PB2-Q602A mutant virus continued to lose body weight until death, whereas all of the mice infected with wild-type virus gained or maintained their body weight during the 14-day observation period (Fig 9B). We also determined virus titers in lung homogenates on Day 3 p.i. and found that the viral load was increased in mice infected with the PB2-Q602A mutant virus compared with those infected with wild-type virus (Fig 9C). Histopathology examination revealed more severe bronchopneumonia with more prominent viral antigen expression in the lungs of PB2-Q602A mutant virus-infected mice compared with wild-type virus-infected mice (Fig 9D and 9E). These data indicate that the CMA-mediated degradation of PB2, which is enhanced by TRIM45, inhibits virus replication and limits virus-induced lethality and disease severity during influenza virus infection in vivo.

## Discussion

Influenza is a serious respiratory infectious disease, with influenza A virus as the dominant causative agent, resulting in frequent seasonal endemics and occasional pandemics in humans. The genome of influenza A virus encodes ten essential proteins and several accessory proteins [31]. Due to the limited encoding capacity of influenza A virus, each of the viral proteins interacts extensively with cellular factors to enable the virus to adapt and efficiently replicate in host cells. Each of the viral proteins also becomes the target of the host defense system, restricting virus replication and limiting disease severity. As a component of the viral RNA-dependent RNA polymerase complex, the PB2 protein plays an important role in the transcription and replication of the viral genome and bears several critical pathogenicity signatures, such as the E627K and D701N substitutions [32–35]. To facilitate efficient virus replication in host cells, PB2 interacts with and employs numerous host cellular factors in its replication cycle, such as CCT [36], CRL4s [37], and Rab11a [38]. PB2 is also targeted by host restriction factors, such as TUFM and TRIM35 [30,39], to limit virus replication. Here, we demonstrated that the host factor TRIM45 interacts with and reduces the expression level of PB2, restricting virus replication. Mechanistic studies revealed that TRIM45 enhances the expression of LAMP-2A and promotes the interaction of PB2 with LAMP-2A, thereby driving the degradation of PB2 through the CMA pathway. We found that TRIM45 leverages its E3 ubiquitin ligase activity to mediate the K48-linked polyubiquitination and proteasomal degradation of the protease CAPN1, thereby preventing CAPN1-mediated cleavage of LAMP-2A. The CMA-mediated degradation of influenza PB2 is an important host defense mechanism that restricts the virulence of influenza virus in vivo.

The TRIM family proteins are multifunctional proteins with ubiquitin E3 ligase activity and are involved in a variety of cellular processes [40]. In particular, TRIM proteins play an important role in the host defense system by counteracting viral infections [23]. In the context of influenza virus infection, TRIM proteins (e.g., TRIM4, TRIM14, TRIM22, TRIM32, TRIM35, and TRIM41) mostly utilize their ubiquitin E3 ligase activity to mediate the proteasomal degradation of viral proteins [30,41–45]. In this study, we found that TRIM45 mediates the degradation of influenza PB2. Interestingly, TRIM45 did not employ its E3 ubiquitin ligase activity to directly degrade PB2 via the proteasomal degradation pathway. Instead, it facilitated the capture of PB2 by the CMA pathway for degradation. Our data thus reveal a novel mechanism by which TRIM proteins antagonize invading pathogens by linking essential viral proteins with the host autophagy degradation system.

Of the three autophagy pathways, (i.e., macroautophagy, microautophagy and CMA), only macroautophagy has been shown to be involved in the influenza virus life cycle. The interplay of M2, NS1, HA, or PB1 with the macroautophagy pathway has beneficial effects on the replication of influenza virus [16–19,28,41,46], whereas the interaction between PB2 and the selective autophagic receptor p62 results in its autophagic degradation [21]. In this study, we found that TRIM45 promotes the degradation of influenza PB2 through autophagy, but without activation of the macroautophagy pathway. This result prompted us to investigate the possibility of PB2 degradation via the CMA pathway. The degradation of target proteins via the CMA pathway requires the presence of a KFERQ-like motif in the protein sequences [29,30]. The search for such a motif led us to identify a stretch of QMRDV residues at positions 602–606 of PB2. Further studies confirmed that the presence of the QMRDV motif was indeed required for PB2 to interact with HSC70 and LAMP-2A, the two central mediators of the CMA pathway. Notably, mutation of any of the five residues of the QMRDV motif abolished the degradation of PB2 via the CMA pathway, and the PB2-Q602A mutant WSN (H1N1) virus grew to significantly higher titers in mouse organs, and exhibited enhanced virulence in mice compared with the wild-type virus. These findings highlight the importance of the CMA pathway in limiting the replication and pathogenicity of influenza virus.

CMA is a multi-step process that begins with the recognition of the substrate protein in the cytosol, where the pentapeptide KFERQ-like motif in the substrate amino acid sequence binds to the chaperone HSC70 [47], and then further binds to the cytoplasmic tail of LAMP-2A, a single transmembrane protein located on the surface of the lysosome as a monomer. The monomeric LAMP-2A interacts with other proteins and forms multimers for the substrate transport process [26,48,49]. To reveal the underlying mechanism by which TRIM45 enhances the expression of LAMP-2A, we investigated the effect of TRIM45 on the stability of CAPN1, a protease that cleaves LAMP-2A. We found that TRIM45 interacted with CAPN1, and mediated the K48-linked polyubiquitination and proteasomal degradation of CAPN1, thereby abrogating CAPN1-mediated cleavage of LAMP-2A. Further investigation identified the residue K654 as the specific targeting site of TRIM45-mediated K48-linked polyubiquitination and proteasomal degradation of CAPN1. These data thus demonstrate that TRIM45 indirectly regulates the expression of LAMP-2A by destabilizing the intermediate regulating factor CAPN1.

**Fig 10:**
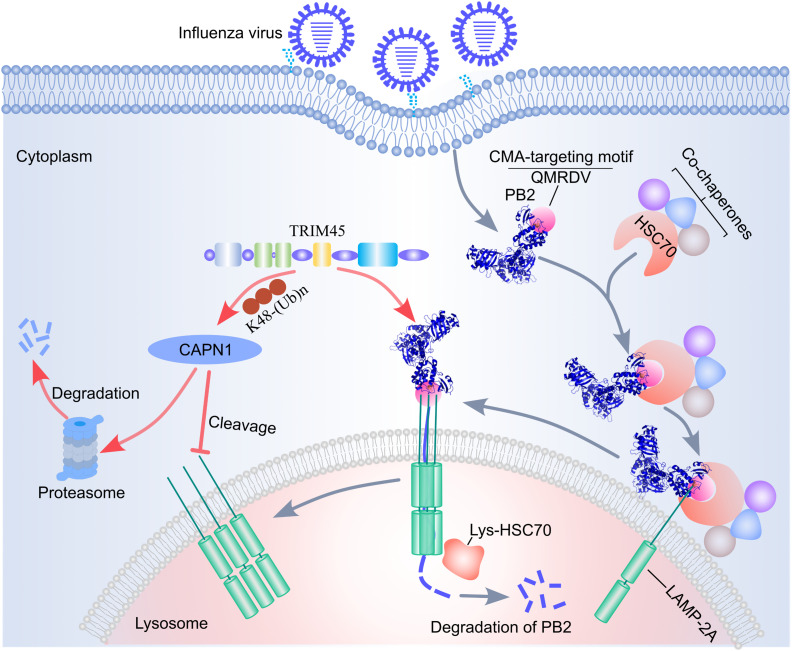
Schematic model showing the role of TRIM45 in modulating the CMA pathway to destabilize PB2 and restrict influenza virus replication. HSC70 and LAMP-2A recognize influenza PB2 via a QMRDV motif, leading to the degradation of PB2 through the CMA pathway. TRIM45 catalyzes the K48-linked polyubiquitination and proteasomal degradation of CAPN1, thereby abrogating CAPN1-mediated cleavage of LAMP-2A. TRIM45 associates with both PB2 and LAMP-2A, which enhances the interaction between LAMP-2A and PB2. In this way, TRIM45 destabilizes PB2 via the CMA pathway and restricts the replication of influenza virus.

In summary, here we demonstrate that TRIM45 interacts with influenza PB2 and restricts virus replication and pathogenesis. Mechanistically, a QMRDV motif in PB2 interacts with HSC70 and LAMP-2A, leading to the degradation of PB2 through the CMA pathway; TRIM45 catalyzes the K48-linked polyubiquitination and proteasomal degradation of CAPN1, thereby enhancing the expression of LAMP-2A as well as the interaction between LAMP-2A and PB2 (Fig 10). The expression of TRIM45 is induced upon influenza infection, and the pathogenicity of the PB2-Q602A mutant virus is significantly enhanced in mice, revealing the biological significance of TRIM45 and the CMA pathway in limiting the replication and virulence of influenza virus. Manipulation of this virus-host interaction mechanism may facilitate the control of influenza virus infections, for example, substitution of QMRDV motif could be employed to increase the growth properties of a vaccine seed virus.

## Materials and methods

### Ethics statements

This study was conducted in strict accordance with the recommendations in the Guide for the Care and Use of Laboratory Animals of the Ministry of Science and Technology of the People’s Republic of China. The protocols for mouse studies were approved by the Committee on the Ethics of Animal Experiments of the Harbin Veterinary Research Institute (HVRI) of the Chinese Academy of Agricultural Sciences (CAAS) (approval number BRDW-XBS–19).

### Biosafety statement and facility

All experiments with live H5N1 and H7N9 viruses were carried out within the enhanced animal biosafety level 3 (ABSL3+) facility at the HVRI, CAAS approved for such use by the Ministry of Agriculture and Rural Affairs of the People’s Republic of China and the China National Accreditation Service for Conformity Assessment.

### Cells and viruses

Human embryonic kidney cells (HEK293 and HEK293T) were cultured in DMEM (Gibco, 11995500BT) supplemented with 10% fetal bovine serum (FBS; Gibco, A5669701). Human lung carcinoma cells (A549) were cultured in F12K (Life Technologies, 312–250-CL) supplemented with 10% FBS. Madin-Darby canine kidney (MDCK) cells were cultured in DMEM containing 5% BCS (Sigma-Aldrich, 12138C). Murine lung epithelial-12 (MLE12) cells were cultured in DMEM containing 10% FBS. All media contained 100 units/mL penicillin and 100 μg/mL streptomycin (Gibco, 15140–122), and all cells were maintained in a humidified incubator at 37˚C with 5% CO_2_.

A/WSN/1933 (WSN, H1N1), A/Anhui/2/2005 (AH05, H5N1), and A/Anhui/1/2013 (AH13, H7N9) viruses were propagated in MDCK cells or 10-day-old embryonated specific-pathogen-free (SPF) eggs as described previously [50]. Recombinant vesicular stomatitis virus carrying EGFP gene (VSV-EGFP) amplified in Vero cells was stored in our laboratory.

### Antibodies

Mouse monoclonal antibodies (mAbs) against influenza PB2 and NP were prepared and stored in our laboratory [51,52]. The primary antibodies obtained from commercial sources included: rabbit anti-PB2 polyclonal antibody (pAb) (GeneTex, GTX125926; 1:1000), rabbit anti-GAPDH pAb (Proteintech, 10494–1-AP; 1:2000), mouse anti-TRIM45 mAb (Proteintech, 66896–1-Ig; 1:1000), rabbit anti-TRIM45 pAb (Sigma-Aldrich, SAB2102559; 1:1000), rabbit anti-LAMP-2A pAb (Abcam, ab125068; 1:1000), rabbit anti-HSC70 pAb (Abcam, ab51052; 1:1000), rabbit anti-CAPN1 pAb (Proteintech, 10538–1-AP; 1:1000), rabbit anti-LC3B pAb (Sigma-Aldrich, L7543; 1:1000), rabbit anti-MX1 pAb (Proteintech, 13750–1-AP; 1:1000), rabbit anti-K48-linkage specific polyubiquitin mAb (Cell Signaling Technology, 8081S; 1:1000), rabbit anti-K63-linkage specific polyubiquitin mAb (Cell Signaling Technology, 5621S; 1:1000), mouse anti-Flag mAb (Sigma-Aldrich, F1804; 1:100), rabbit anti-Flag pAb (Sigma-Aldrich, F7425; 1:1000), mouse anti-V5 mAb (Genscript, A01724; 1:100), rabbit anti-V5 pAb (Sigma-Aldrich, V8012; 1:1000), rabbit anti-GST pAb (Genscript, A00097; 1:1000), mouse anti-Myc mAb (Genscript, A00704; 1:100), rabbit anti-Myc pAb (Genscript, A00172; 1:1000), rabbit anti-HA tag pAb (Proteintech, 51064–2-AP; 1:1000), and mouse IgG (Beyotime Biotechnology, A7028; 1:200). Secondary antibodies DyLight 680 goat anti-mouse IgG (H + L) (ImmunoWay Biotechnology, RS23710; 1:8000) and DyLight 680 goat anti-rabbit IgG (H + L) (ImmunoWay Biotechnology, RS23720; 1:8000) were used for western blotting. Alexa Fluor 633 goat anti-mouse IgG (H + L) (Invitrogen, A21050; 1:500) and Alexa Fluor 488 donkey anti-rabbit IgG (H + L) (Invitrogen, A21206; 1:500) used for confocal microscopy were obtained from Life Technologies.

### Plasmids

The construction of pCAGGS plasmids expressing the PB2, PB1, PA, and NP proteins of WSN (H1N1) virus has been described previously [50]. The open reading frame (ORF) of PB2 from AH05 (H5N1) and AH13 (H7N9) viruses was cloned into pCAGGS with or without a Flag or V5 tag at the C-terminus. The deletion and point mutants of the PB2 of WSN (H1N1), AH05 (H5N1), and AH13 (H7N9) virus were generated by PCR and cloned into pCAGGS with a Flag or V5 tag at the C-terminus. The Q602A mutation was introduced into the full-length PB2 gene of WSN (H1N1) virus by use of PCR and cloned into the pHH21 vector [53]. The ORFs of the PB1 and PA of WSN (H1N1) virus were cloned into pCAGGS with a C-terminal Flag tag, the ORFs of HA and NA were cloned into pCAGGS with a C-terminal Myc tag, the ORFs of M1, M2, and NS2 were cloned into pCAGGS with a C-terminal GST tag, and the ORF of NS1 was cloned into pCAGGS with a C-terminal V5 tag. The ORF of TRIM45 was cloned into pCDNA3.1 vector with a V5 tag at the C-terminus. The ORF of murine TRIM45 (Mm-TRIM45) was inserted into pCAGGS with an HA tag at the N-terminus. The ORFs of HSC70 and LAMP-2A were inserted into pCAGGS with a Myc tag at the C-terminus. Truncation mutants of TRIM45 were generated by using PCR and cloned into pCDNA3.1 with a V5 tag at the C-terminus, and the coiled-coil (CC) domain of TRIM45 was amplified by PCR and cloned into pCDNA3.1 with a C-terminal GST tag. The ORF of CAPN1 was cloned into pCAGGS with a Flag tag at the C-terminus. Point mutants of CAPN1 were generated by use of PCR and cloned into pCAGGS with a Flag tag at the C-terminus. The plasmids expressing HA-tagged ubiquitin (Ub) and its K48 and K63 mutants, and the plasmid pHH21-SC09NS F-Luc, used to express a viral RNA-like firefly luciferase gene under the control of the human RNA polymerase I promoter, have been reported previously [30,50]. All constructs were sequenced to ensure that no unwanted mutations were present.

### Co-immunoprecipitation assay (Co-IP)

HEK293T cells grown in 6-well plates were transfected with the indicated plasmids for 48 h by using Lipofectamine 2000 (Invitrogen, 11668019), or were transfected with the indicated plasmids for 24 h and further infected with the indicated virus (MOI = 5) for 12 h; and A549 cells grown in 6-well plates were infected with WSN (H1N1) virus (MOI = 5) for 12 h. The cells were then washed twice with ice-cold PBS and lysed with IP buffer (containing 25 mM Tris-HCl pH 7.4, 150 mM NaCl, 1% NP-40, 1 mM EDTA, 5% glycerol; Pierce, 87788) supplemented with complete protease inhibitor cocktail (Roche, 05892953001) on ice for 30 min. The cell lysates were centrifuged at 13,000 *g* at 4˚C for 10 min, and the supernatants were mixed with the indicated antibodies and Protein G- Agarose beads (Roche, 11,243,233,001). The mixture was incubated at 4˚C overnight, and washed 4–6 times with wash buffer (25 mM Tris-HCl pH 7.4, 150 mM NaCl, 1 mM PMSF; Beyotime Biotechnology, ST506). The bound proteins were then boiled at 95°C for 5 min in 2x Laemmli sample buffer, separated by 10% sodium dodecyl sulfate-polyacrylamide gel electrophoresis (SDS-PAGE), and subjected to western blotting.

### Western blotting

Protein samples were separated by SDS-PAGE and transferred onto nitrocellulose membranes (GE Healthcare, USA). After blocking with 5% skim milk in PBS at room temperature for 1 h, the membranes were incubated at 4˚C for 12 h with appropriately diluted primary antibodies in PBS. After three washes with PBST, the membranes were incubated with DyLight 680 goat anti-mouse IgG (H + L) and DyLight 680 goat anti-rabbit IgG (H + L) at room temperature for 1 h. After another three washes with PBST, an Odyssey CLX infrared imaging system (Li-Cor BioSciences, USA) was used to visualize the blots.

### Immunofluorescence assay

HEK293T cells grown in glass-bottom culture dishes were transfected with plasmids expressing V5-tagged TRIM45 for 24 h and then infected with WSN (H1N1), AH05 (H5N1), or AH13 (H7N9) virus (MOI = 5), or were transfected with plasmids expressing Myc-tagged LAMP-2A for 24 h and then infected with WSN (H1N1) virus (MOI = 5); and A549 cells grown in glass-bottom culture dishes were infected with WSN (H1N1) virus (MOI = 5). At the indicated timepoints p.i., the cells were fixed with 4% paraformaldehyde (PFA) in PBS for 30 min and permeabilized with 0.1% Triton-X-100 in PBS for 20 min. After blocking with 5% bovine serum albumin in PBS for 1 h, the cells were stained with the indicated primary antibody and incubated with the corresponding secondary antibody coupled to Alexa Fluor 488 or Alexa Fluor 633. Nuclei were stained with DAPI (Sigma-Aldrich, F6057). Images were acquired by using a LSM 800 confocal microscope with Airyscan (Zeiss, Germany).

### siRNA sequences

The siRNA sequences used in this study were: TRIM45 siRNA (5’-GCUGAACAAAGUUCAAUAUTT-3’) and scrambled siRNA (5’-UUCUUCGAACGUGUCACGU-3’). All siRNAs were obtained from Genepharma (Shanghai, China).

### siRNA transfection and virus infection

A549 cells seeded in 12-well plates were transfected with siRNA targeting TRIM45 or scrambled siRNA at a concentration of 30 nM by using the Lipofectamine RNAiMAX transfection reagent (Invitrogen, 13778–150). The knockdown efficiency was checked by western blotting at 36 h post-transfection. To determine the effect of TRIM45 silencing on the replication of influenza virus, A549 cells treated with siRNA for 24 h were infected with WSN (H1N1) (MOI = 0.1), AH05 (H5N1) (MOI = 0.1), or AH13 (H7N9) (MOI = 0.1) virus. Supernatants were collected at 24 and 48 h post-infection (p.i.), and virus titers were determined by performing plaque assays on MDCK cells. To determine the effect of siRNA treatment on viral PB2 protein expression, A549 cells were transfected with TRIM45 siRNA or scrambled siRNA, and at 36 h post-transfection, the cells were infected with WSN (H1N1) (MOI = 5), AH05 (H5N1) (MOI = 5), or AH13 (H7N9) (MOI = 5) virus. At 0, 4, 6, and 8 h p.i., the level of viral PB2 protein was detected by western blotting.

### Generation of knockout (KO) cell lines and virus infection

The TRIM45_KO A549 and LAMP-2A_KO HEK293 cell lines were generated by using the CRISPR/Cas9 system. The TRIM45-targeting guide RNA (gRNA) sequence, CACCGCTACTTCCCTGTCGTTGCAC, or LAMP-2A-targeting gRNA sequence, CACCGTCTTCCCGGTTCCGGGCTCA, was inserted into the gRNA expression cassette of the pX458 vector. A Neon Transfection System (ThermoFisher Scientific, USA) was used to transfect the gRNA constructs of TRIM45 into A549 cells or the gRNA constructs of LAMP-2A into HEK293 cells. The electrotransfected cells were cultured for 2 days, and were then trypsinized and separated into single cells by using a SH800S Cell Sorter (Sony Biotechnology, USA). The sorted cells were individually propagated in 24-well plates, and the knockout of TRIM45 or LAMP-2A expression was confirmed by direct sequencing, RT-qPCR assays, and/or western blotting. The TRIM45_KO or control A549 cells were infected with WSN (H1N1), AH05 (H5N1), or AH13 (H7N9) virus at an MOI of 0.1. Virus titers in the supernatant were determined by use of plaque assays on MDCK cells at 24 and 48 h p.i.

Additionally, the TRIM45_KO or control A549 cells grown in 12-well plates were infected with VSV-EGFP [100 50% tissue culture infective doses (TCID_50_)]. Supernatants were collected at 8 and 12 h p.i. and titrated on MDCK cells by calculating the TCID_50_.

### RT-qPCR assays

Total RNAs of LAMP-2A_KO or control HEK293 cells were extracted by using the RNAsimple Total RNA Kit (Tiangen, A0117A). The first-strand cDNAs were synthesized with an oligo(dT) primer by using the HiScript II Q RT SuperMix with gDNA wiper (Vazyme, R223-01). RT-qPCR assays were performed with ChamQ SYBR qPCR Master Mix (Vazyme, Q711-03) using QuantStudio 5 (Applied Biosystems, USA). The primers used for RT-qPCR were: LAMP-2A-F, 5’-CCGCTCGAGCGGATGGTGTGCTTCCGCCTCTTCCC-3’; LAMP-2A-R, 5’-GGAAGATCTTCCCTAAAATTGCTCATATCCAGCATGATGGTGCTTGAGACC-3’; GAPDH-F, 5’-AGGTGAAGGTCGGAGTCA-3’; and GAPDH-R, 5’-GGTCATTGATGGCAACAA-3’. Relative RNA quantities were determined by using the comparative cycle threshold method in which the GAPDH gene serves as the internal control.

### MX1 expression under IFN-α or IFN-β stimulation

The TRIM45_KO or control A549 cells grown in 12-well plates were left untreated or treated with 100 U/mL of IFN-α (Sigma-Aldrich, 14276) or 25 pg/mL of IFN-β (R&D Systems, P01574) for 24 h. Cell lysates were then subjected to western blotting with a rabbit anti-MX1 pAb to determine the level of MX1 protein expression.

### Effect of murine TRIM45 on virus replication

MLE12 cells were transfected with plasmids expressing Mm-TRIM45-HA or empty vector for 24 h, and then infected with WSN (H1N1) virus at an MOI of 0.1. Virus titers in the supernatants were measured by use of plaque assays on MDCK cells at 24 and 48 h p.i. In an additional experiment, MLE12 cells were transfected with plasmids expressing Mm-TRIM45-HA or empty vector for 24 h, and then infected with WSN (H1N1) virus (MOI = 5). At 0, 4, 6, and 8 h p.i., cell lysates were western blotted with a rabbit anti-HA or anti-PB2 pAb.

### Luciferase assay

HEK293T cells were transfected with the four constructs for the expression of the RNP complex proteins from WSN (H1N1) virus (pCAGGS-PB2, pCAGGS-PB1, pCAGGS-PA, and pCAGGS-NP), pHH21-SC09NS F-Luc, and pRL-TK, together with gradually increasing amounts of the plasmid expressing TRIM45-V5. At 24 h post-transfection, cell lysates were subjected to the luciferase assay with a dual luciferase reporter assay system on a GloMax 96 microplate luminometer (Promega, USA). Data were normalized for transfection efficiency by calculating the ratio of firefly luciferase activity to Renilla luciferase activity.

### Ubiquitination assay

To analyze the effect of TRIM45 on the ubiquitination of CAPN1, HEK293T cells were transfected with the indicated combinations of plasmids expressing TRIM45-V5, TRIM45- DelR-V5, HA-Ub, HA-Ub(K48), HA-Ub(K63), CAPN1-Flag, and the Flag-tagged CAPN1 mutants for 16 h, and then treated for 8 h with DMSO or MG132 (10 μg/mL). Whole cell lysates were immunoprecipitated with a mouse anti-Flag mAb, and the bound proteins were analyzed by western blotting with a rabbit anti-HA pAb, rabbit anti-Flag pAb, and rabbit anti-V5 pAb.

Additionally, to assess the ubiquitination of CAPN1 mediated by endogenous ubiquitin, HEK293T cells were transfected with the indicated combinations of plasmids expressing CAPN1-Flag, TRIM45-V5 and TRIM45-DelR-V5 for 16 h, and then treated for 8 h with DMSO or MG132 (10 μg/mL). Whole cell lysates were immunoprecipitated with a mouse anti-Flag mAb, and the bound proteins were western blotted with a rabbit anti-Flag pAb and a rabbit anti-K48 polyubiquitin or anti-K63 polyubiquitin mAb.

### Plaque assay

Plaque assays were performed in MDCK cells as described previously [54]. Briefly, virus samples were 10-fold serially diluted in 1 × MEM (0.3% BSA) and used to infect MDCK cells in 12-well plates at 37˚C for 1 h. After removing the inoculum, the cells were washed once with PBS and overlaid with 1% SeaPlaque agarose (Lonza, 50100) in 1 × MEM containing 0.3% BSA and 0.5 μg/mL TPCK-treated trypsin. After 48–72 h of incubation, the cells were fixed with formalin, and the plaques were stained with 0.1% crystal violet and counted.

### Generation of mutant virus and cell infection

WSN (H1N1) virus bearing the PB2-Q602A mutation was generated by using the reverse genetics system [53]. The rescued viruses were fully sequenced to ensure the absence of unwanted mutations. HEK293 cells were transfected with plasmids expressing TRIM45 or empty vector for 24 h, and then infected with PB2-Q602A mutant WSN (H1N1) virus at an MOI of 0.1. Virus titers in the supernatant were determined by using plaque assays on MDCK cells at 24 and 48 h p.i.

Moreover, the TRIM45_KO A549 cells were transfected with plasmids expressing TRIM45-V5, TRIM45-DelR-V5 or empty vector for 24 h, and then infected with WSN (H1N1) virus or PB2-Q602A mutant WSN (H1N1) virus at an MOI of 0.1. Virus titers in the supernatants were measured by use of plaque assays on MDCK cells at 24 and 48 h p.i.

### Mouse study

C57BL/6J mice (6-week-old, female) were anesthetized with CO_2_ and intranasally infected with wild-type or PB2-Q602A mutant WSN (H1N1) virus (2 × 10^3^ PFU). Body weight and survival of groups of five mice were monitored daily for 14 days. To assess virus replication, the infected mice (three mice per group) were euthanized on Day 3 p.i., and their lungs, nasal turbinate, brain, spleen, and kidneys were collected, homogenized, and titrated for infectious virus by use of plaque assays on MDCK cells.

Lung samples from mice infected with wild-type or PB2-Q602A mutant WSN (H1N1) virus were collected on Days 3 and 6 p.i., fixed in 10% neutral buffered formalin, embedded in paraffin, and cut into 4-μm sections. The sections were stained with hematoxylin-eosin (H&E) or used in immunohistochemical (IHC) assays with a rabbit anti-NP pAb and horseradish peroxidase-conjugated goat anti-rabbit IgG.

### Statistical analysis

Data were statistically analyzed by using GraphPad Prism 7.0 software (San Diego, USA) with a two-tailed unpaired Student’s t-test. A *P* value < 0.05 was considered statistically significant.

## Supporting information

S1 Fig TRIM45 negatively regulates the replication of IAV. (A) A549 cells were treated with TRIM45 siRNA or scrambled siRNA for 36 h, and then infected with WSN (H1N1) virus (MOI = 0.1). At 48 h p.i., the cells were observed under an inverted microscope. (B) TRIM45_KO and control A549 cells were infected with WSN (H1N1) virus (MOI = 0.1). At 48 h p.i., the cells were observed under an inverted microscope. Scale bar, 200 μm (A), 400 μm (B).
(TIF)

S2 Fig The expression of TRIM45 is induced upon IAV infection. (A) A549 cells were infected with WSN (H1N1) virus (MOI = 5). At 0, 4, 8, and 12 h p.i., the levels of TRIM45 were examined by western blotting with a mouse anti-TRIM45 mAb. (B) A549 cells infected as in (A) were subjected to confocal microscopy with a rabbit anti-TRIM45 pAb and a mouse anti-NP mAb. The levels of TRIM45 expression, indicated by the mean fluorescence intensity, was quantified using ImageJ software (1.53k). Scale bar, 10 μm. For the right part of B, error bars indicate SEMs calculated from three replicates. *n* = 3; two-tailed unpaired Student’s t-test.
(TIF)

S3 Fig TRIM45 does not affect the interferon (IFN) signaling pathway. (A, B) A549 or TRIM45_KO A549 cells were left untreated or treated with IFN-α (A) or IFN-β (B) for 24 h. The cell lysates were western blotted with a rabbit anti-MX1 pAb for the detection of the MX1 protein.
(TIF)

S4 Fig TRIM45 does not affect the replication of VSV-EGFP virus. A549 or TRIM45_KO A549 cells were infected with VSV-EGFP virus at a dose of 100 TCID_50_. Supernatants were collected at 8 and 12 h p.i. and titrated on MDCK cells by calculating the TCID_50_. Error bars indicate SEMs calculated from three replicates. *n* = 3; two-tailed unpaired Student’s t-test.
(TIFF)

S5 Fig TRIM45 interacts only with IAV PB2 protein. (A-J) Co-IP and western blotting analysis of HEK293T cells expressing TRIM45-V5 or TRIM45-Flag and PB2 (A), PB1-Flag (B), PA-Flag (C), HA-Myc (D), NP (E), NA-Myc (F), M1-GST (G), M2-GST (H), NS1-V5 (I), or NS2-GST (J) of WSN (H1N1) virus.
(TIF)

S6 Fig IAV PB2 interacts with endogenous TRIM45. (A-C) A549 cells were infected with WSN (H1N1) (A), AH05 (H5N1) (B) or AH13 (H7N9) (C) virus (MOI = 5). At 12 h p.i., the cell lysates were immunoprecipitated with a mouse anti-TRIM45 mAb, and the bound proteins were western blotted with a rabbit anti-TRIM45 or anti-PB2 pAb.
(TIF)

S7 Fig TRIM45 co-localizes with IAV PB2 protein. (A-C) The co-localization of TRIM45 and PB2 of WSN (H1N1) (A), AH05 (H5N1) (B), or AH13 (H7N9) (C) virus as in Fig 2G was analyzed by ImageJ (1.53k).
(TIF)

S8 Fig Murine TRIM45 inhibits the replication of IAV. (A) MLE12 cells were transfected with plasmids expressing HA-tagged murine TRIM45 (HA-Mm-TRIM45) or empty vector for 24 h, and then infected with WSN (H1N1) virus at an MOI of 0.1. Virus titers in the supernatants were measured by plaque assay at 24 and 48 h p.i. (B) MLE12 cells were transfected with plasmids expressing HA-Mm-TRIM45 or empty vector for 24 h, and then infected with WSN (H1N1) virus (MOI = 5). At 0, 4, 6, and 8 h p.i., cell lysates were western blotted with a rabbit anti-HA or anti-PB2 pAb. For the right part of A, error bars indicate SEMs calculated from three replicates. *n* = 3; two-tailed unpaired Student’s t-test.
(TIF)

S9 Fig The expression of TRIM45 reduces the vRNP complex activity of IAV. HEK293T cells were transfected with the vRNP complex reconstitution plasmids (pCAGGS-WSN PB2, pCAGGS-WSN PB1, pCAGGS-WSN PA, pCAGGS-WSN NP, and pHH21-SC09NS F-Luc), pRL-TK, together with gradually increasing amount of TRIM45-V5-expressing plasmids. At 24 h post-transfection, cell lysates were subjected to luciferase assay with a dual luciferase reporter assay system. Data were normalized for transfection efficiency by calculating the ratio between the firefly luciferase activity and the Renilla luciferase activity. Error bars indicate SEMs calculated from three replicates. n = 3; two-tailed unpaired Student’s t-test.
(TIF)

S10 Fig Rapamycin treatment does not affect TRIM45-mediated degradation of the IAV PB2 protein. HEK293T cells were transfected with plasmids expressing TRIM45-V5 or empty vector for 12 h, and then infected with WSN (H1N1) virus (MOI = 5). At 12 h p.i., the cells were treated with DMSO or rapamycin (RAP) (100 nM) for 12 h, and cell lysates were western blotted with a rabbit anti-V5 or anti-PB2 pAb.
(S10_Fig.TIF)

S11 Fig LAMP-2A co-localizes with IAV PB2 protein. The co-localization of LAMP-2A-Myc and PB2 of WSN (H1N1) virus as in Fig 5K was analyzed by ImageJ (1.53k).
(S11_Fig.TIF)

S12 Fig 602–606 QMRDV motif in PB2 is essential for TRIM45-mediated PB2 degradation. (A-C) HEK293T cells were transfected with the indicated combinations of plasmids expressing TRIM45-V5, and Flag-tagged wild-type PB2 or QMRDV-deleted PB2 of WSN (H1N1) (A), AH05 (H5N1) (B), or AH13 (H7N9) (C) virus. At 16 h post-transfection, the cells were treated with DMSO or NH_4_Cl (5 mM) for 12 h, and cell lysates were western blotted with a rabbit anti-V5 or anti-Flag pAb.
(S12_Fig.TIF)

S13 Fig Complement of TRIM45 or TRIM45-DelR mutant in TRIM45_KO A549 cells does not affect the replication of PB2-Q602A mutant WSN (H1N1) virus. (A) TRIM45_KO A549 cells were transfected with plasmids expressing TRIM45-V5, TRIM45-DelR-V5 or empty vector for 12 h, and then infected with wild-type or PB2-Q602A mutant of WSN (H1N1) virus (MOI = 5). At 12 h p.i., the cell lysates were western blotted with a rabbit anti-V5 or anti-PB2 pAb. (B) TRIM45_KO A549 cells were transfected with plasmids expressing TRIM45-V5, TRIM45-DelR-V5 or empty vector for 24 h, and then infected with wild-type or PB2-Q602A mutant of WSN (H1N1) virus (MOI = 0.1). Virus titers in the supernatants were measured by plaque assays at 24 and 48 h p.i. For B, error bars indicate SEMs calculated from three replicates. *n* = 3; two-tailed unpaired Student’s t-test.
(TIF)

S14 Fig TRIM45 enhances the expression of endogenous LAMP-2A and its interaction with PB2. HEK293T cells were transfected with the indicated combinations of plasmids expressing TRIM45-V5 and WSN (H1N1) PB2 for 24 h. The cell lysates were immunoprecipitated with a mouse anti-PB2 mAb, and the bound proteins were western blotted with a rabbit anti-LAMP-2A or anti-PB2 pAb.
(S14_Fig.TIF)

S15 Fig CAPN1 knockdown elevates LAMP-2A level and consequently causes PB2 degradation. A549 cells were transfected with siRNA targeting CAPN1 or scrambled siRNA for 36 h, and then infected with WSN (H1N1) virus at an MOI of 5. At 12 h p.i., cell lysates were western blotted with a rabbit anti-LAMP-2A, anti-PB2 or anti-CAPN1 pAb.
(TIF)

S16 Fig TRIM45 knockout increases CAPN1 level and concomitantly reduces LAMP-2A level. TRIM45_KO or control A549 cells were infected with WSN (H1N1) virus at an MOI of 5. At 12 h p.i., cell lysates were western blotted with a rabbit anti-CAPN1 pAb, a rabbit anti-LAMP-2A pAb or a mouse anti-TRIM45 mAb.
(TIF)

S17 Fig TRIM45 mediates K48-linked polyubiquitination of CAPN1 using endogenous ubiquitin. (A-C) HEK293T cells were transfected with the indicated combinations of plasmids expressing CAPN1-Flag, TRIM45-V5 and TRIM45-DelR-V5 for 16 h, and then treated with DMSO or MG132 for 8 h. The cell lysates were immunoprecipitated with a mouse anti-Flag mAb, and the bound proteins were western blotted with a rabbit anti-Flag pAb and a rabbit anti-K48 (A, C) or anti-K63 (B) mAb.
(TIF)

S1 File Source data - with values. (XLSX)

S2 File Source data - Western blotting images. (PDF)
