# Supplementary figures and images for: TRIM45 restricts influenza virus infection through modulating the chaperone-mediated autophagic degradation of viral PB2 protein
Source: PLoS Pathog. 2025 Oct 23;21(10):e1013630. doi: 10.1371/journal.ppat.1013630 (PMC12578348; doi:10.1371/journal.ppat.1013630)

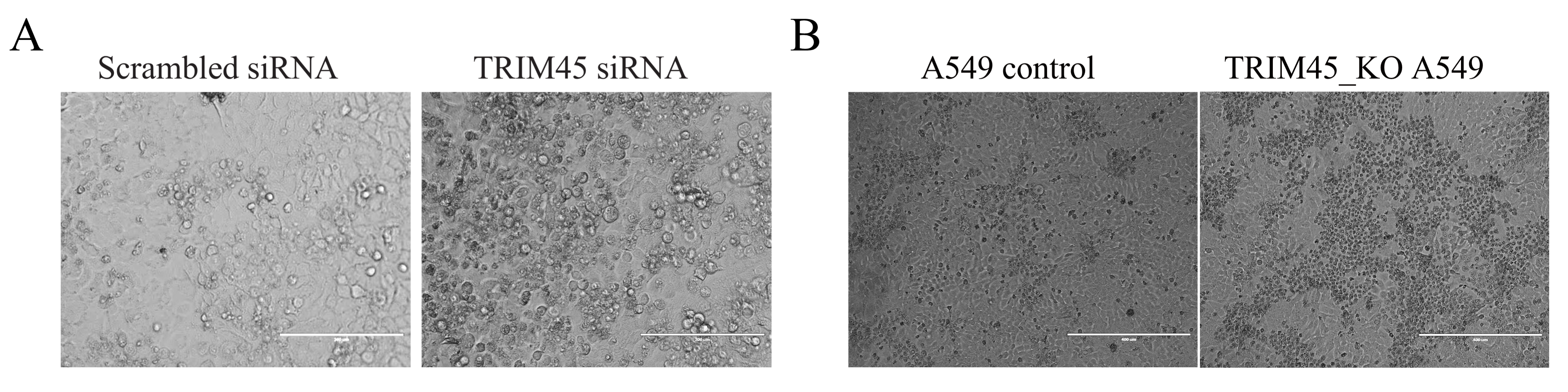

Supplement: S1 Fig — (A) A549 cells were treated with TRIM45 siRNA or scrambled siRNA for 36 h, and then infected with WSN (H1N1) virus (MOI = 0.1). At 48 h p.i., the cells were observed under an inverted microscope. (B) TRIM45_KO and control A549 cells were infected with WSN (H1N1) virus (MOI = 0.1). At 48 h p.i., the cells were observed under an inverted microscope. Scale bar, 200 μm (A), 400 μm (B). (TIF) [file ppat.1013630.s001.tif]

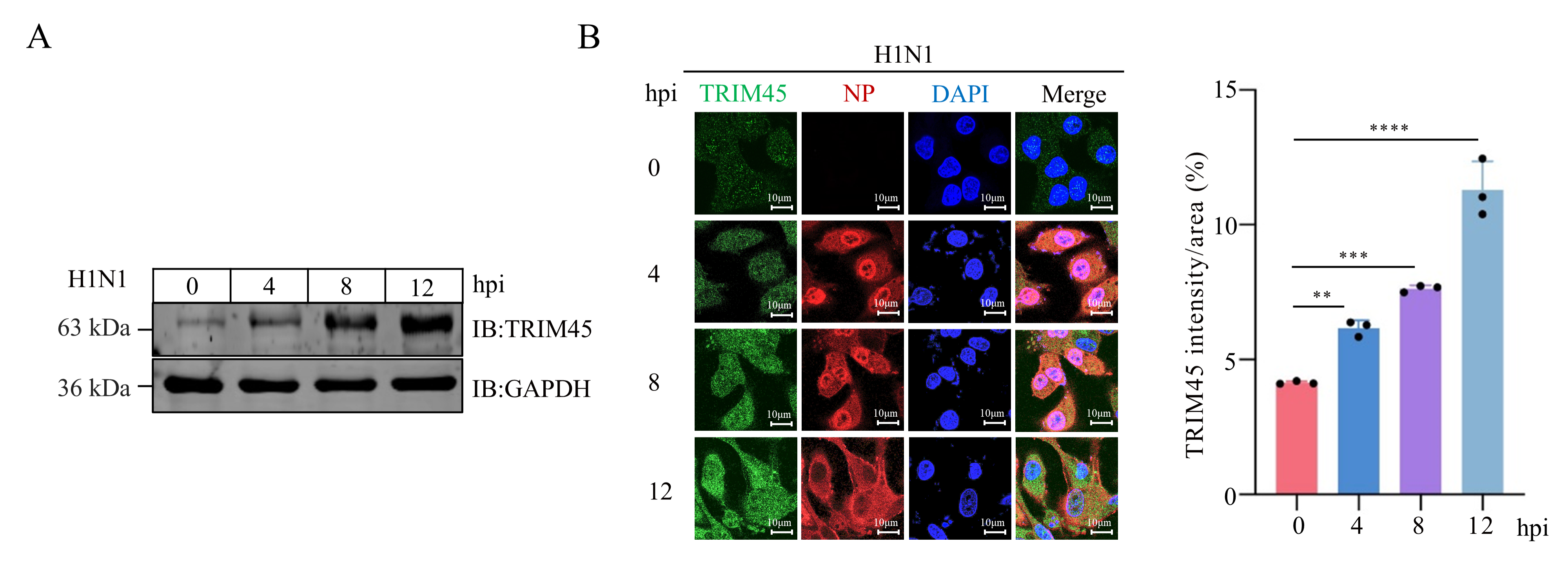

Supplement: S2 Fig — (A) A549 cells were infected with WSN (H1N1) virus (MOI = 5). At 0, 4, 8, and 12 h p.i., the levels of TRIM45 were examined by western blotting with a mouse anti-TRIM45 mAb. (B) A549 cells infected as in (A) were subjected to confocal microscopy with a rabbit anti-TRIM45 pAb and a mouse anti-NP mAb. The levels of TRIM45 expression, indicated by the mean fluorescence intensity, was quantified using ImageJ software (1.53k). Scale bar, 10 μm. For the right part of B, error bars indicate SEMs calculated from three replicates. n = 3; two-tailed unpaired Student’s t-test. (TIF) [file ppat.1013630.s002.tif]

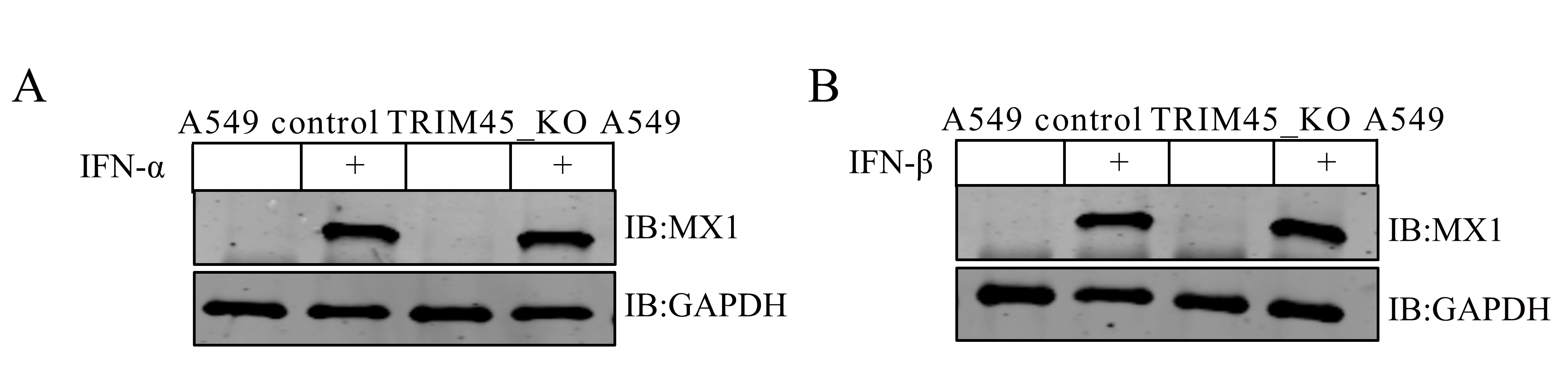

Supplement: S3 Fig — (A, B) A549 or TRIM45_KO A549 cells were left untreated or treated with IFN-α (A) or IFN-β (B) for 24 h. The cell lysates were western blotted with a rabbit anti-MX1 pAb for the detection of the MX1 protein. (TIF) [file ppat.1013630.s003.tif]

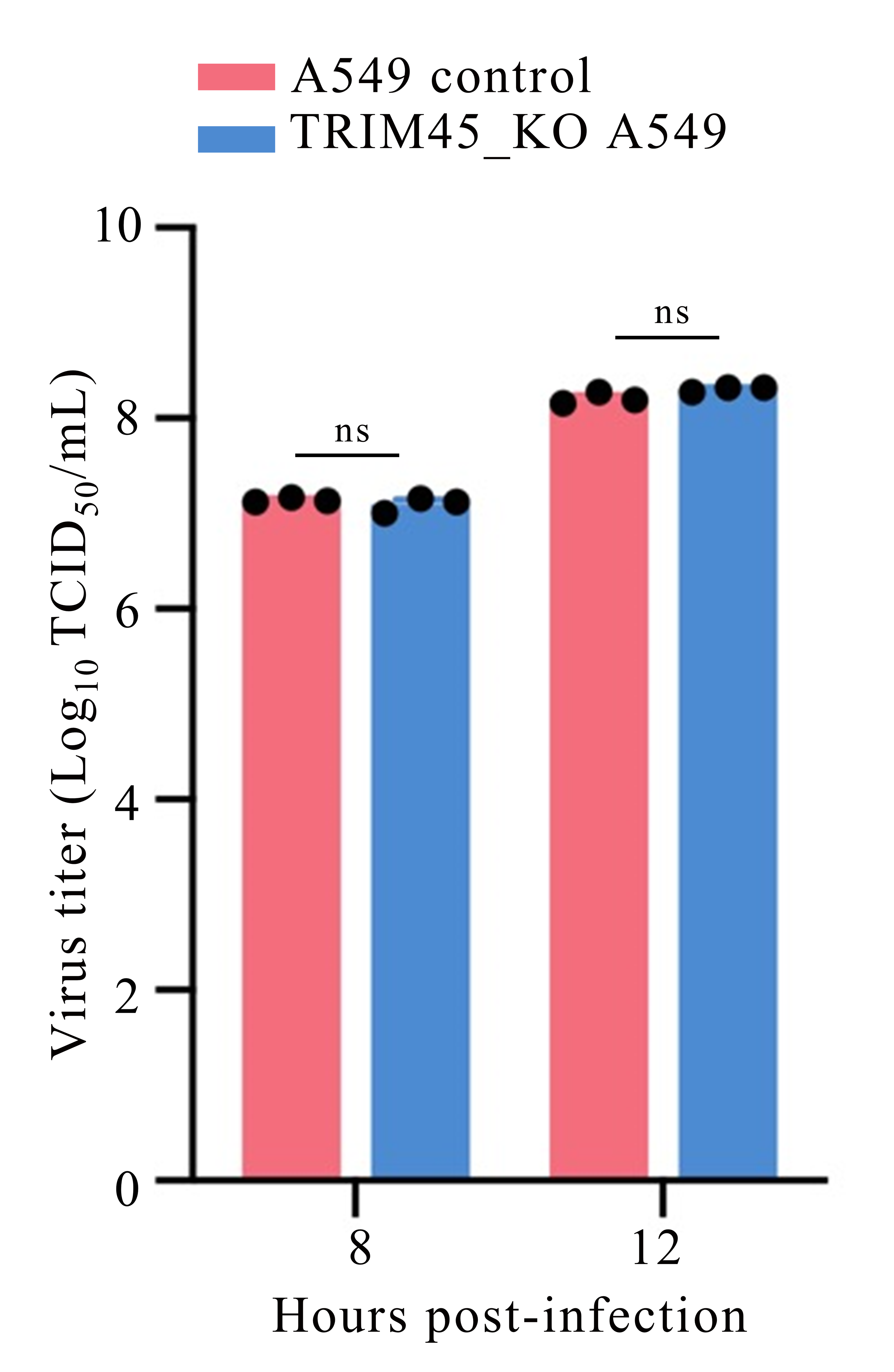

Supplement: S4 Fig — A549 or TRIM45_KO A549 cells were infected with VSV-EGFP virus at a dose of 100 TCID50. Supernatants were collected at 8 and 12 h p.i. and titrated on MDCK cells by calculating the TCID50. Error bars indicate SEMs calculated from three replicates. n = 3; two-tailed unpaired Student’s t-test. (TIFF) [file ppat.1013630.s004.tiff]

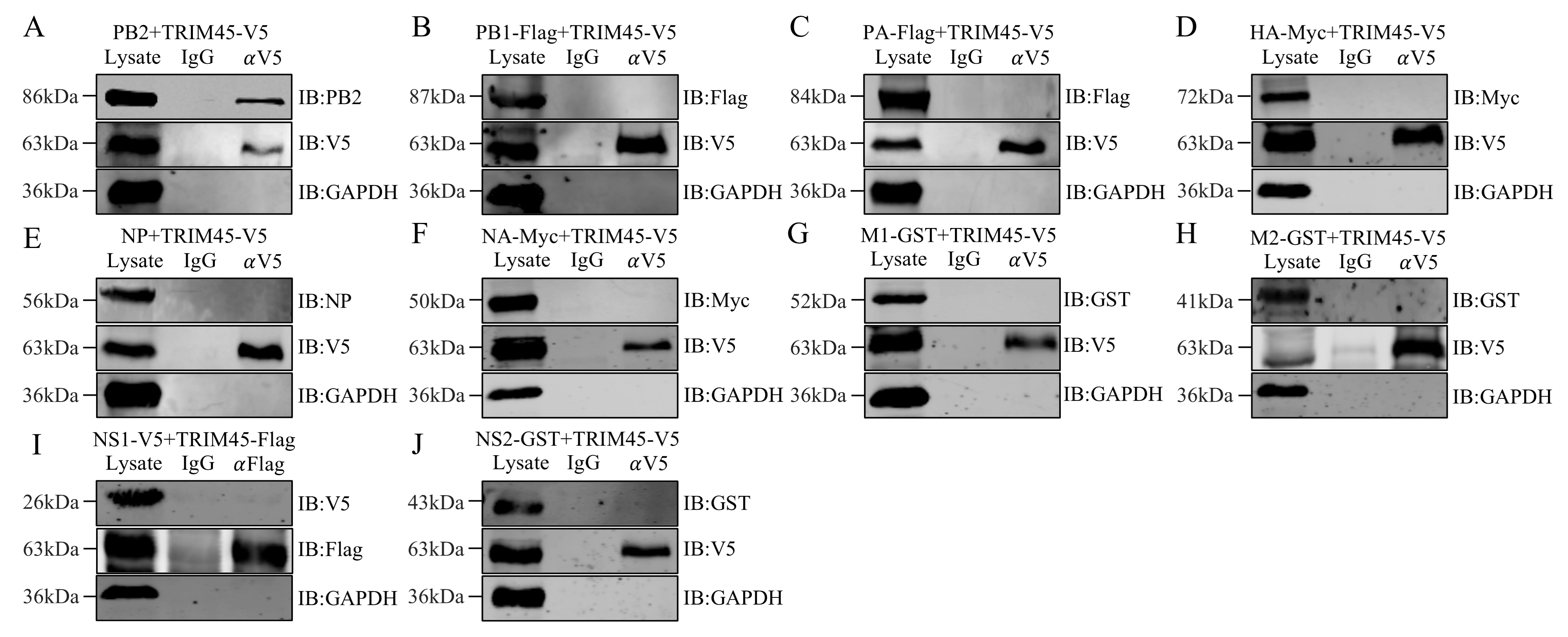

Supplement: S5 Fig — (A-J) Co-IP and western blotting analysis of HEK293T cells expressing TRIM45-V5 or TRIM45-Flag and PB2 (A), PB1-Flag (B), PA-Flag (C), HA-Myc (D), NP (E), NA-Myc (F), M1-GST (G), M2-GST (H), NS1-V5 (I), or NS2-GST (J) of WSN (H1N1) virus. (TIF) [file ppat.1013630.s005.tif]

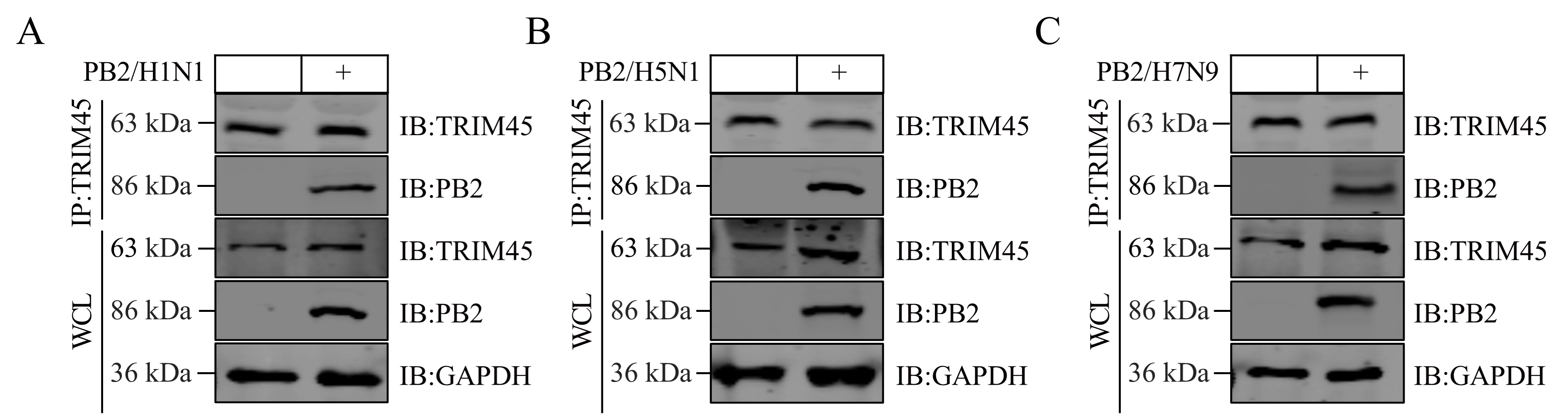

Supplement: S6 Fig — (A-C) A549 cells were infected with WSN (H1N1) (A), AH05 (H5N1) (B) or AH13 (H7N9) (C) virus (MOI = 5). At 12 h p.i., the cell lysates were immunoprecipitated with a mouse anti-TRIM45 mAb, and the bound proteins were western blotted with a rabbit anti-TRIM45 or anti-PB2 pAb. (TIF) [file ppat.1013630.s006.tif]

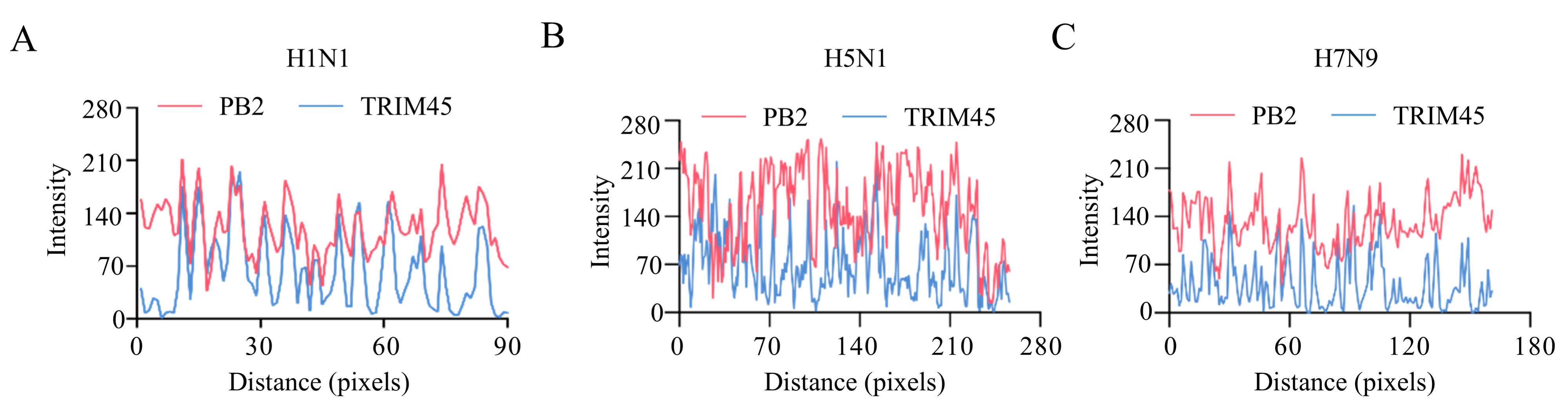

Supplement: S7 Fig — (A-C) The co-localization of TRIM45 and PB2 of WSN (H1N1) (A), AH05 (H5N1) (B), or AH13 (H7N9) (C) virus as in Fig 2G was analyzed by ImageJ (1.53k). (TIF) [file ppat.1013630.s007.tif]

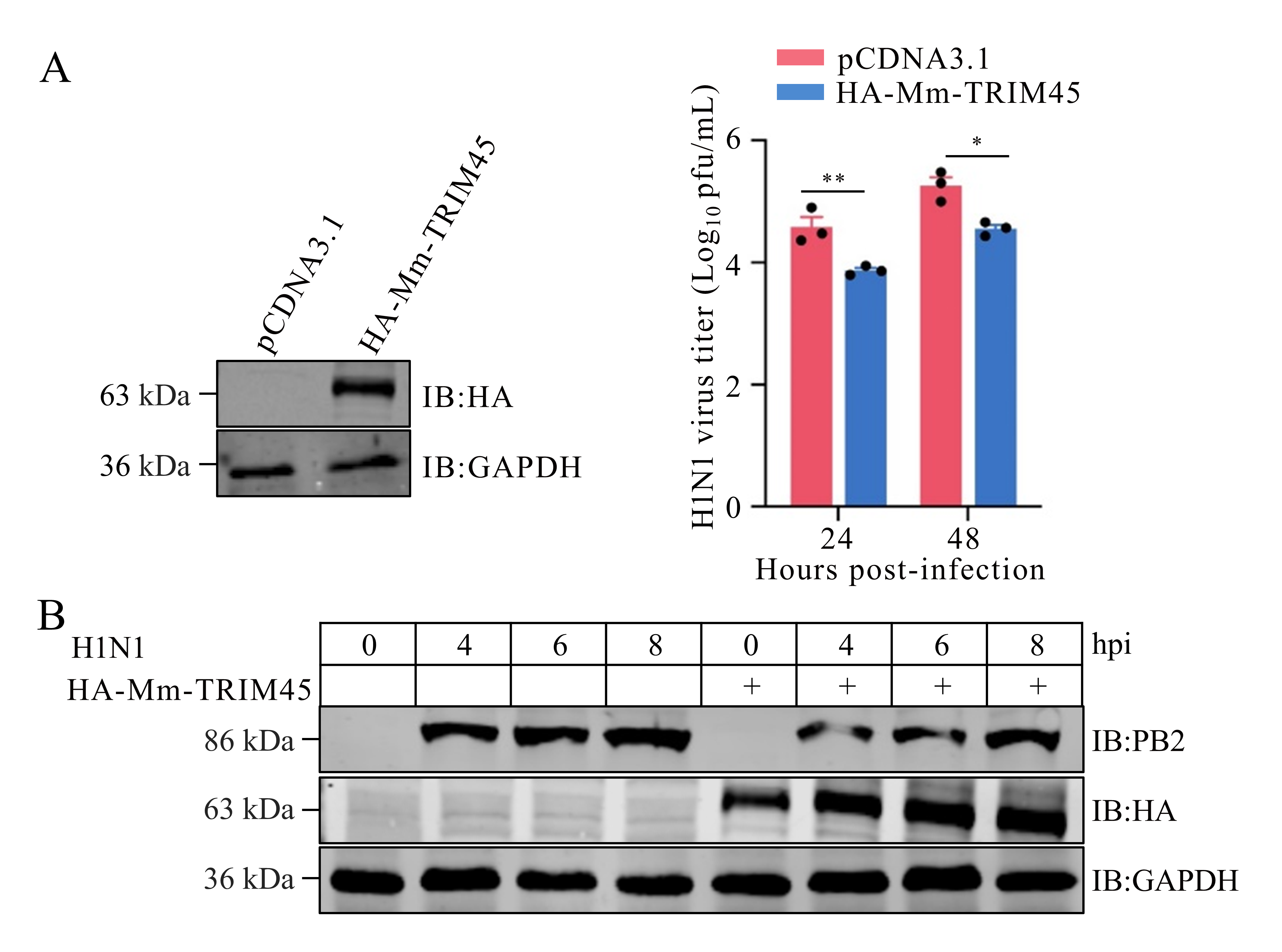

Supplement: S8 Fig — (A) MLE12 cells were transfected with plasmids expressing HA-tagged murine TRIM45 (HA-Mm-TRIM45) or empty vector for 24 h, and then infected with WSN (H1N1) virus at an MOI of 0.1. Virus titers in the supernatants were measured by plaque assay at 24 and 48 h p.i. (B) MLE12 cells were transfected with plasmids expressing HA-Mm-TRIM45 or empty vector for 24 h, and then infected with WSN (H1N1) virus (MOI = 5). At 0, 4, 6, and 8 h p.i., cell lysates were western blotted with a rabbit anti-HA or anti-PB2 pAb. For the right part of A, error bars indicate SEMs calculated from three replicates. n = 3; two-tailed unpaired Student’s t-test. (TIF) [file ppat.1013630.s008.tif]

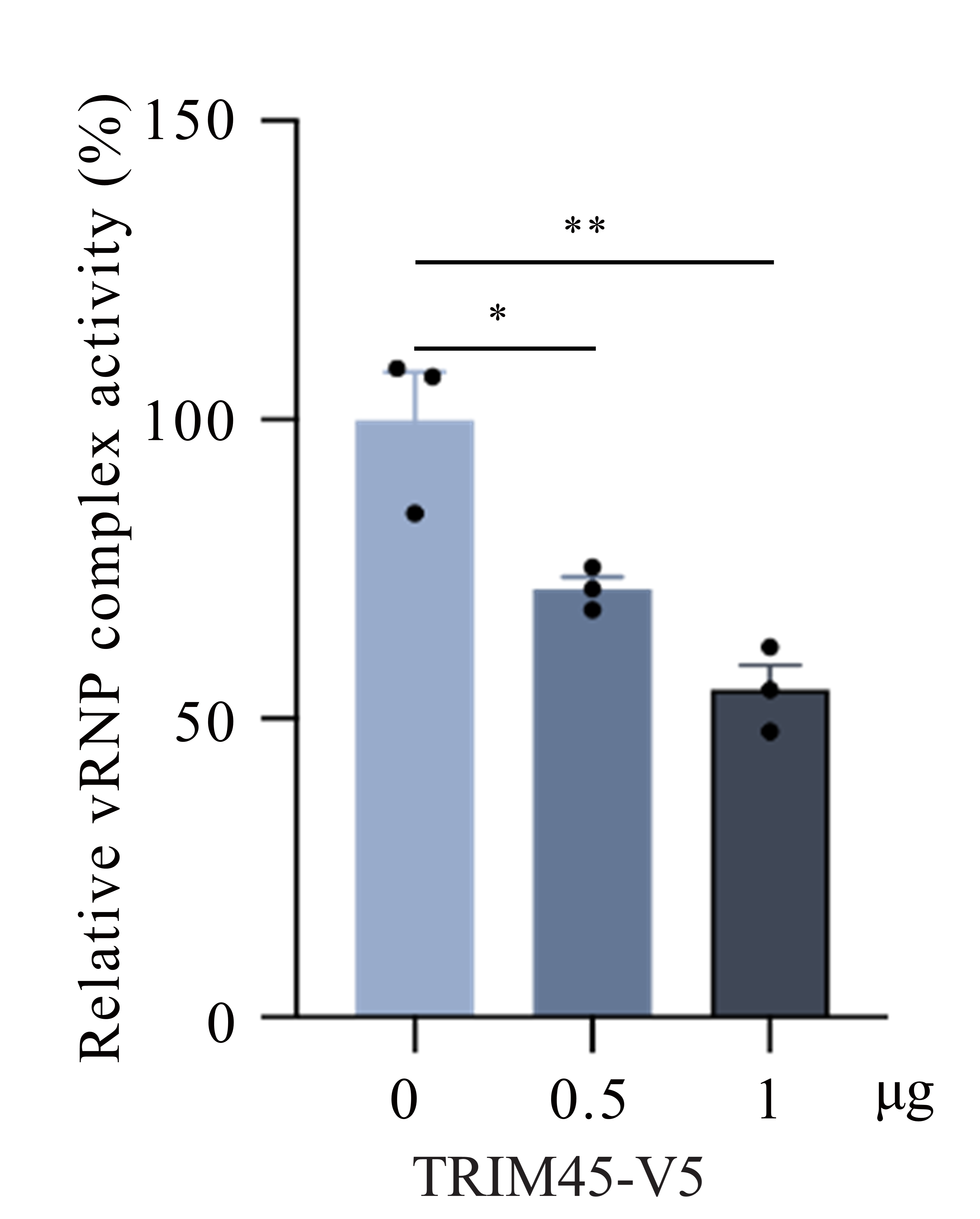

Supplement: S9 Fig — HEK293T cells were transfected with the vRNP complex reconstitution plasmids (pCAGGS-WSN PB2, pCAGGS-WSN PB1, pCAGGS-WSN PA, pCAGGS-WSN NP, and pHH21-SC09NS F-Luc), pRL-TK, together with gradually increasing amount of TRIM45-V5-expressing plasmids. At 24 h post-transfection, cell lysates were subjected to luciferase assay with a dual luciferase reporter assay system. Data were normalized for transfection efficiency by calculating the ratio between the firefly luciferase activity and the Renilla luciferase activity. Error bars indicate SEMs calculated from three replicates. n = 3; two-tailed unpaired Student’s t-test. (TIF) [file ppat.1013630.s009.tif]

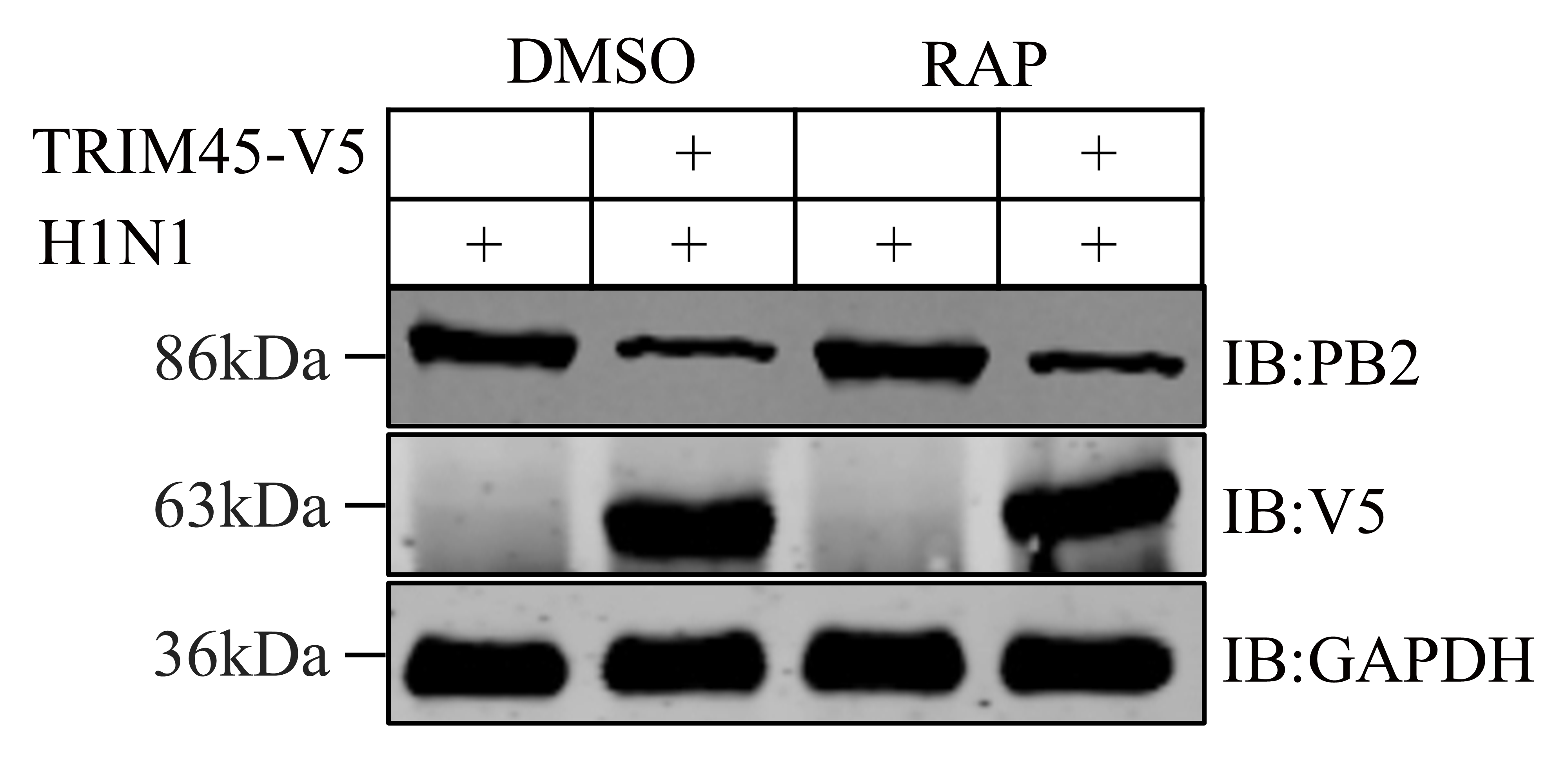

Supplement: S10 Fig — HEK293T cells were transfected with plasmids expressing TRIM45-V5 or empty vector for 12 h, and then infected with WSN (H1N1) virus (MOI = 5). At 12 h p.i., the cells were treated with DMSO or rapamycin (RAP) (100 nM) for 12 h, and cell lysates were western blotted with a rabbit anti-V5 or anti-PB2 pAb. (S10_Fig.TIF) [file ppat.1013630.s010.tif]

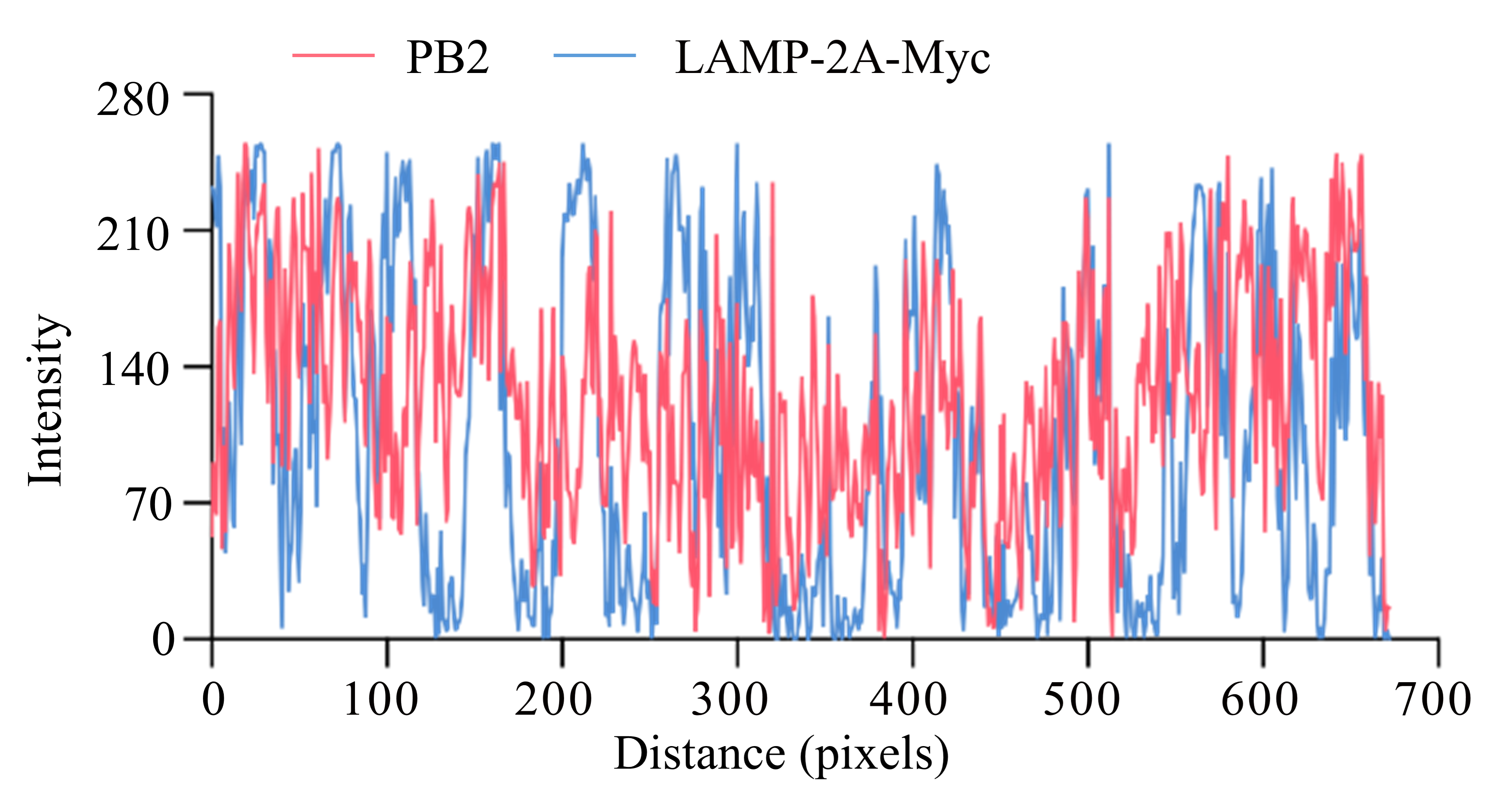

Supplement: S11 Fig — The co-localization of LAMP-2A-Myc and PB2 of WSN (H1N1) virus as in Fig 5K was analyzed by ImageJ (1.53k). (S11_Fig.TIF) [file ppat.1013630.s011.tif]

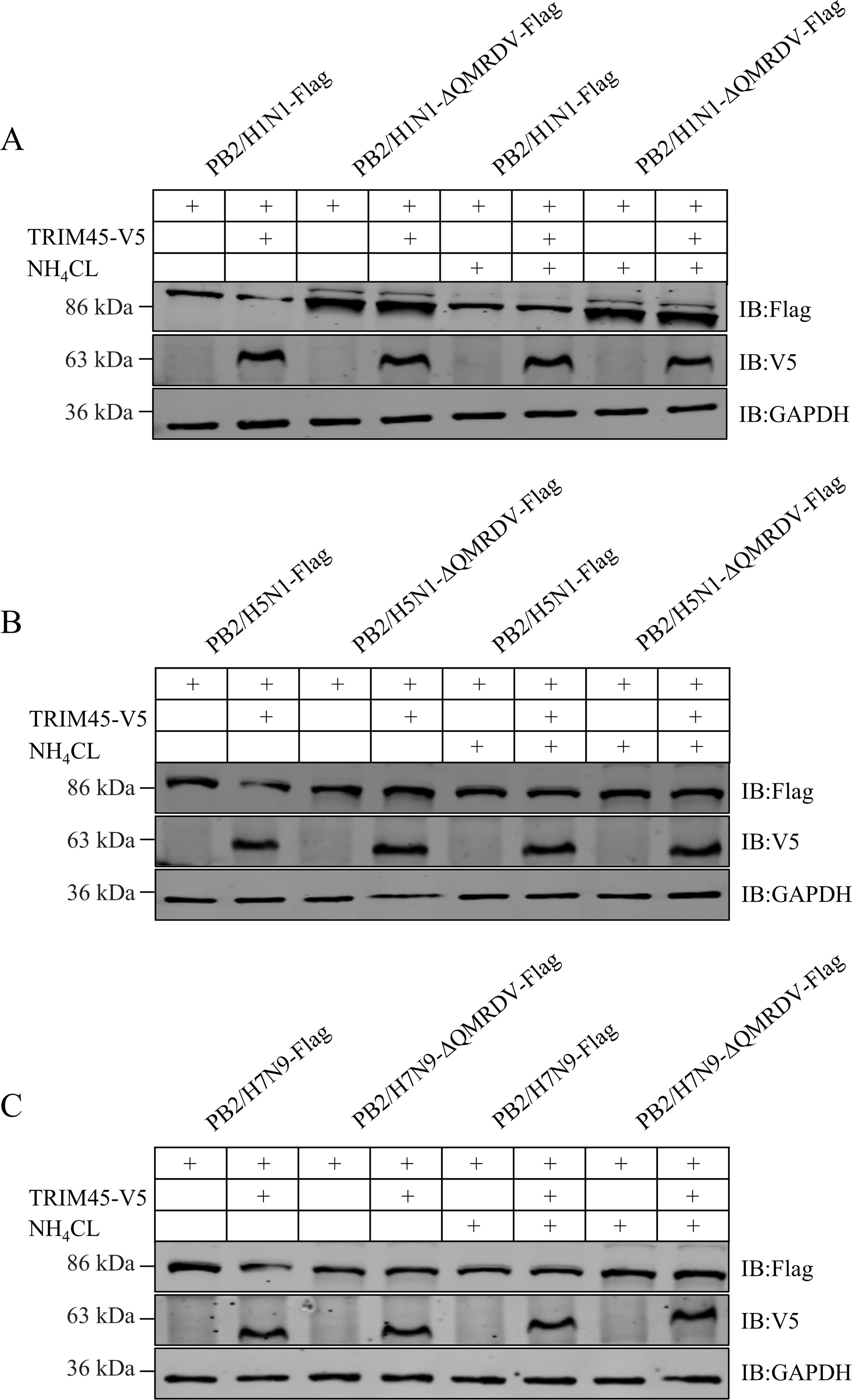

Supplement: S12 Fig — (A-C) HEK293T cells were transfected with the indicated combinations of plasmids expressing TRIM45-V5, and Flag-tagged wild-type PB2 or QMRDV-deleted PB2 of WSN (H1N1) (A), AH05 (H5N1) (B), or AH13 (H7N9) (C) virus. At 16 h post-transfection, the cells were treated with DMSO or NH4Cl (5 mM) for 12 h, and cell lysates were western blotted with a rabbit anti-V5 or anti-Flag pAb. (S12_Fig.TIF) [file ppat.1013630.s012.tif]

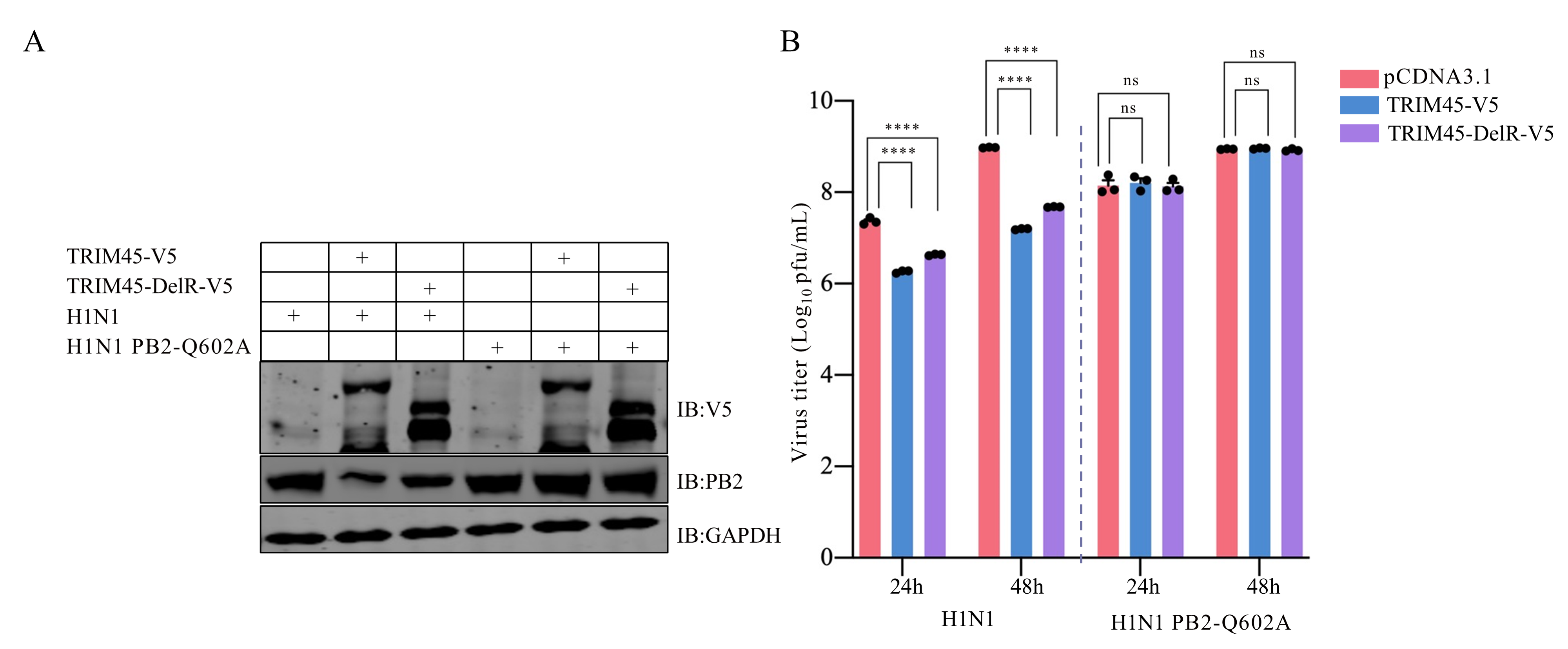

Supplement: S13 Fig — (A) TRIM45_KO A549 cells were transfected with plasmids expressing TRIM45-V5, TRIM45-DelR-V5 or empty vector for 12 h, and then infected with wild-type or PB2-Q602A mutant of WSN (H1N1) virus (MOI = 5). At 12 h p.i., the cell lysates were western blotted with a rabbit anti-V5 or anti-PB2 pAb. (B) TRIM45_KO A549 cells were transfected with plasmids expressing TRIM45-V5, TRIM45-DelR-V5 or empty vector for 24 h, and then infected with wild-type or PB2-Q602A mutant of WSN (H1N1) virus (MOI = 0.1). Virus titers in the supernatants were measured by plaque assays at 24 and 48 h p.i. For B, error bars indicate SEMs calculated from three replicates. n = 3; two-tailed unpaired Student’s t-test. (TIF) [file ppat.1013630.s013.tif]

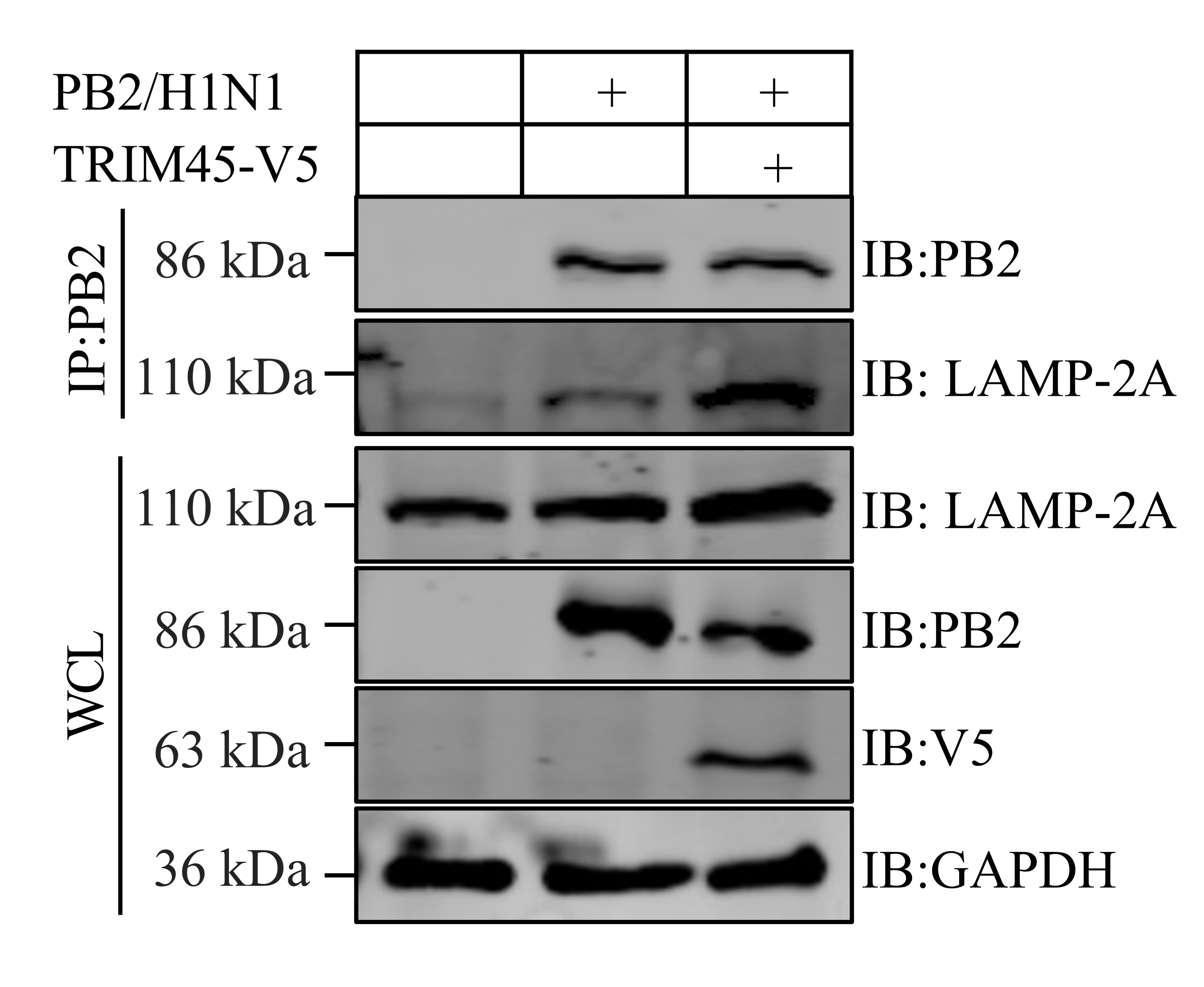

Supplement: S14 Fig — HEK293T cells were transfected with the indicated combinations of plasmids expressing TRIM45-V5 and WSN (H1N1) PB2 for 24 h. The cell lysates were immunoprecipitated with a mouse anti-PB2 mAb, and the bound proteins were western blotted with a rabbit anti-LAMP-2A or anti-PB2 pAb. (S14_Fig.TIF) [file ppat.1013630.s014.tif]

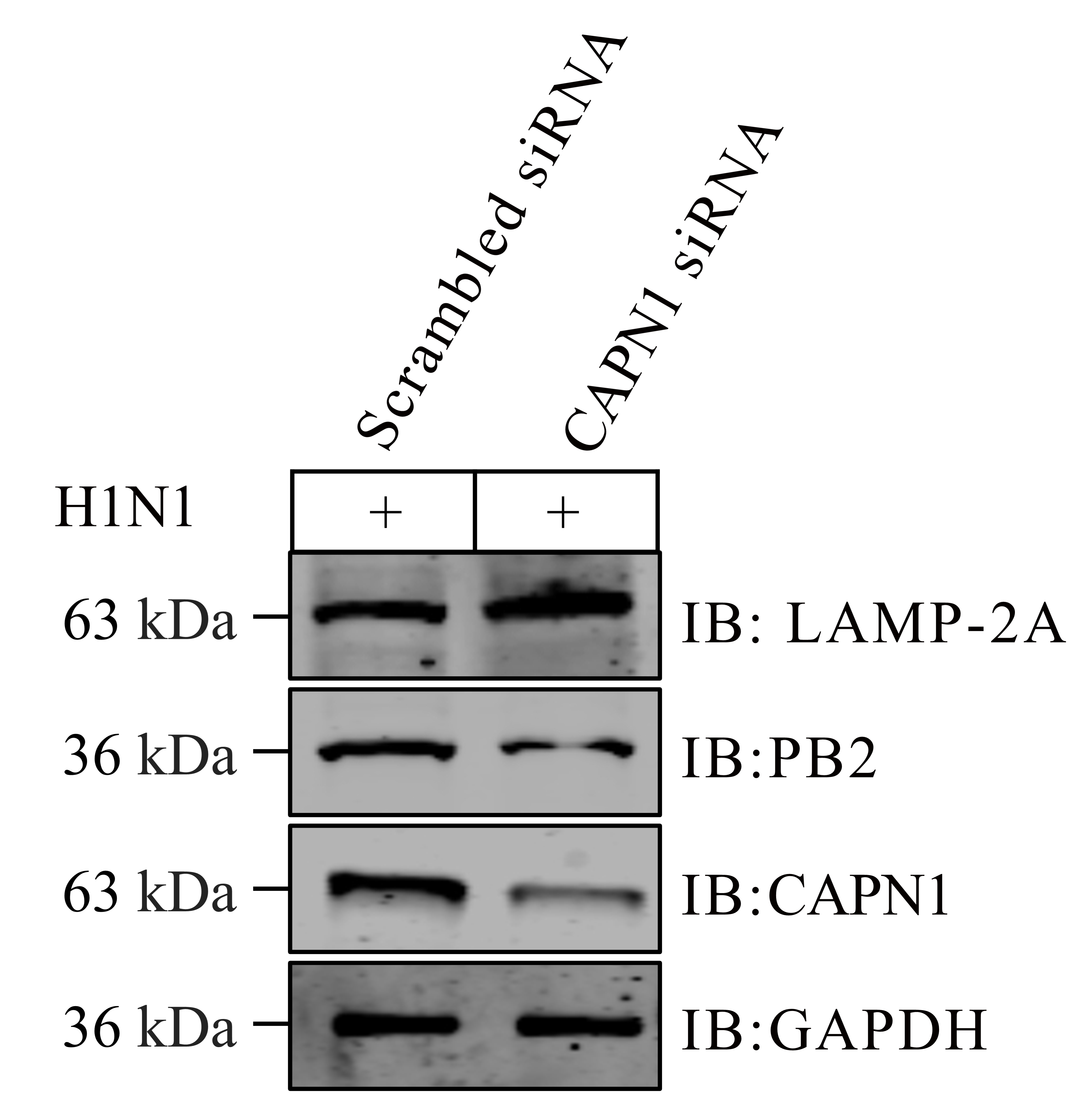

Supplement: S15 Fig — A549 cells were transfected with siRNA targeting CAPN1 or scrambled siRNA for 36 h, and then infected with WSN (H1N1) virus at an MOI of 5. At 12 h p.i., cell lysates were western blotted with a rabbit anti-LAMP-2A, anti-PB2 or anti-CAPN1 pAb. (TIF) [file ppat.1013630.s015.tif]

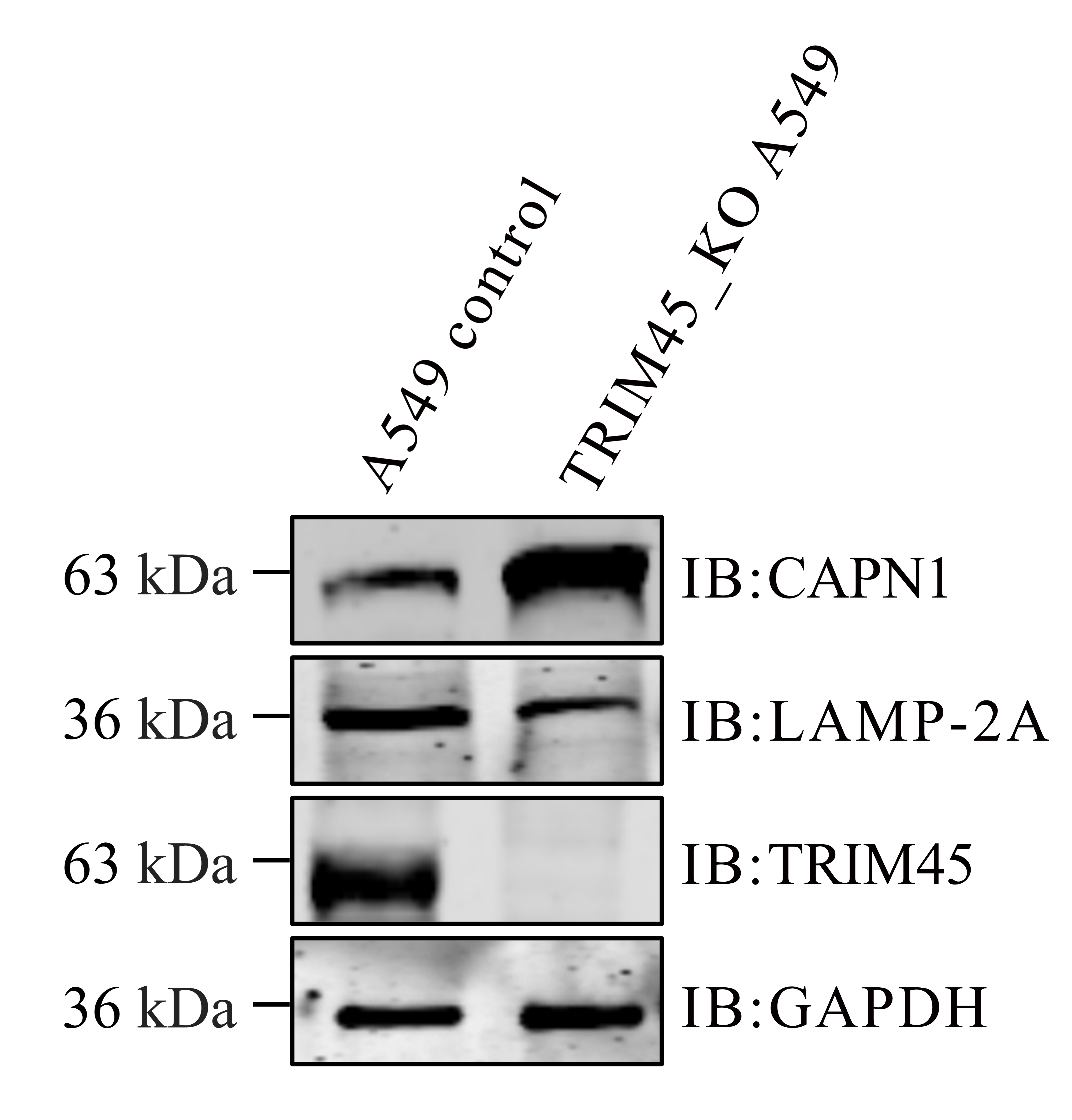

Supplement: S16 Fig — TRIM45_KO or control A549 cells were infected with WSN (H1N1) virus at an MOI of 5. At 12 h p.i., cell lysates were western blotted with a rabbit anti-CAPN1 pAb, a rabbit anti-LAMP-2A pAb or a mouse anti-TRIM45 mAb. (TIF) [file ppat.1013630.s016.tif]

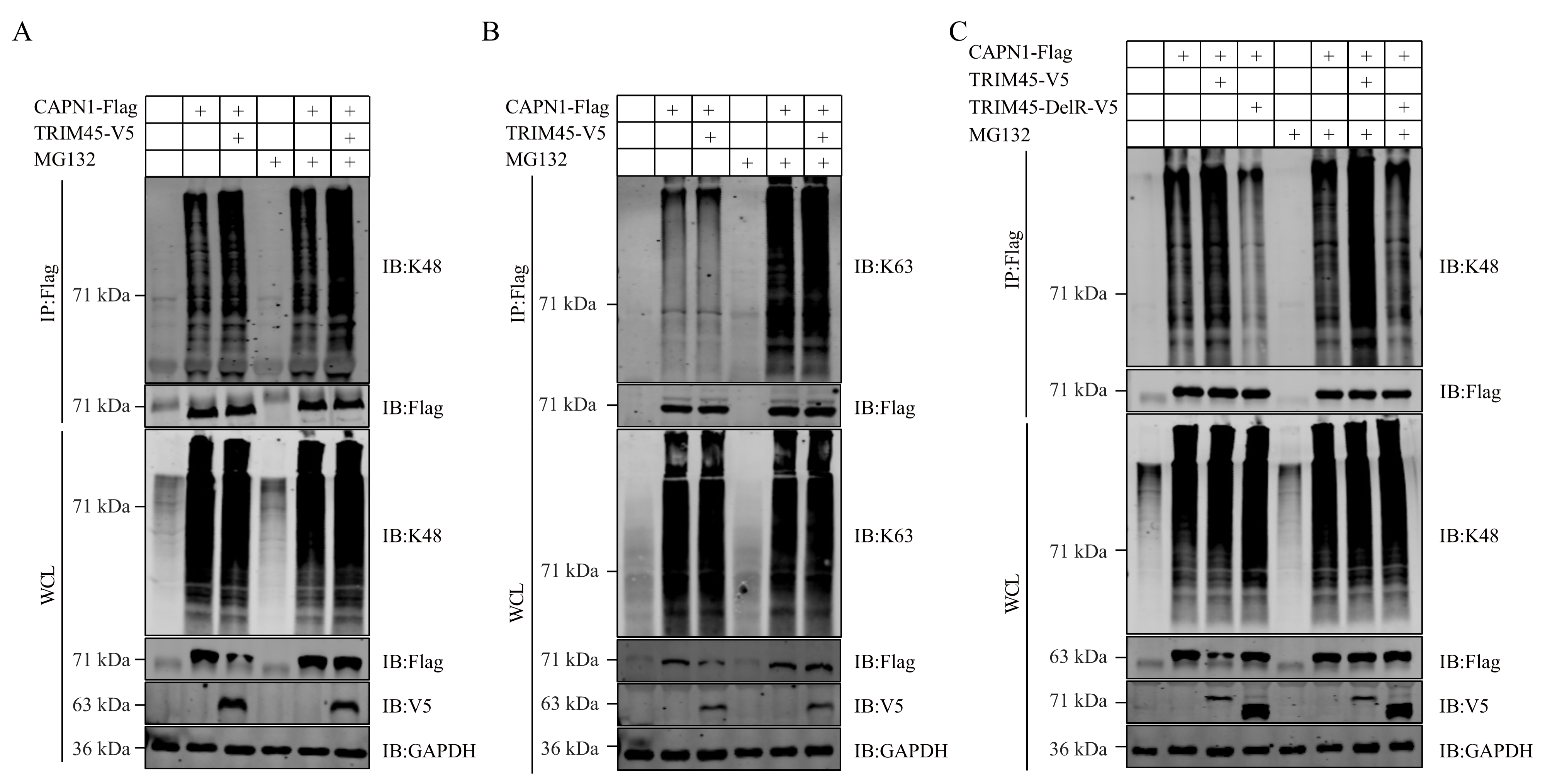

Supplement: S17 Fig — (A-C) HEK293T cells were transfected with the indicated combinations of plasmids expressing CAPN1-Flag, TRIM45-V5 and TRIM45-DelR-V5 for 16 h, and then treated with DMSO or MG132 for 8 h. The cell lysates were immunoprecipitated with a mouse anti-Flag mAb, and the bound proteins were western blotted with a rabbit anti-Flag pAb and a rabbit anti-K48 (A, C) or anti-K63 (B) mAb. (TIF) [file ppat.1013630.s017.tif]
