# Supplementary material for: TRIM45 restricts influenza virus infection through modulating the chaperone-mediated autophagic degradation of viral PB2 protein
Source: PLoS Pathog. 2025 Oct 23;21(10):e1013630. doi: 10.1371/journal.ppat.1013630 (PMC12578348; doi:10.1371/journal.ppat.1013630)
Supplement: S2 File — (PDF) [file ppat.1013630.s019.pdf]

Fig 1A

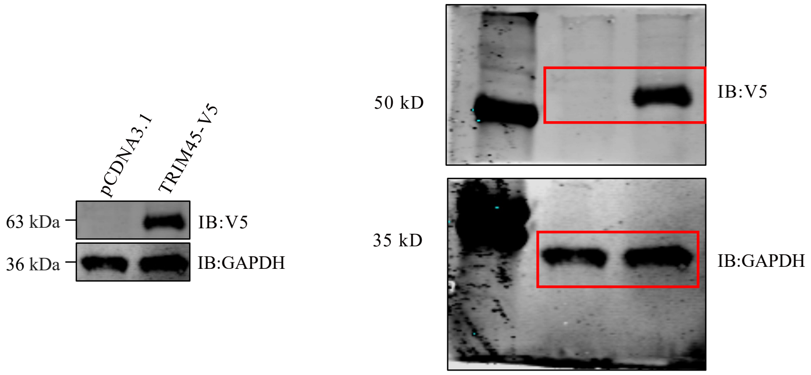

Fig 1E

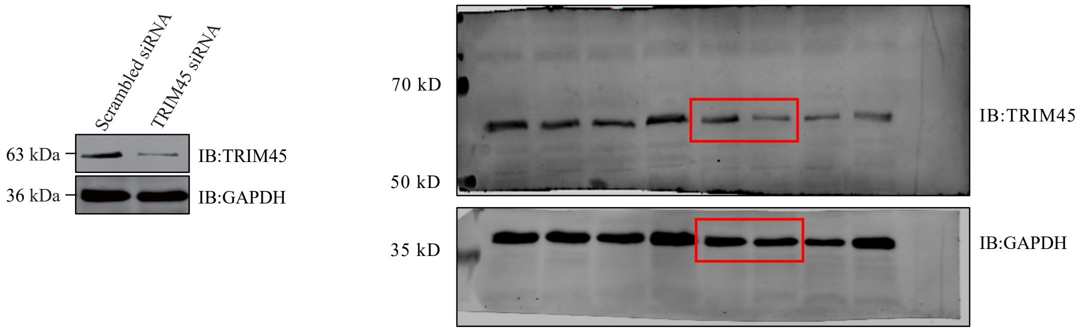

Fig 1I

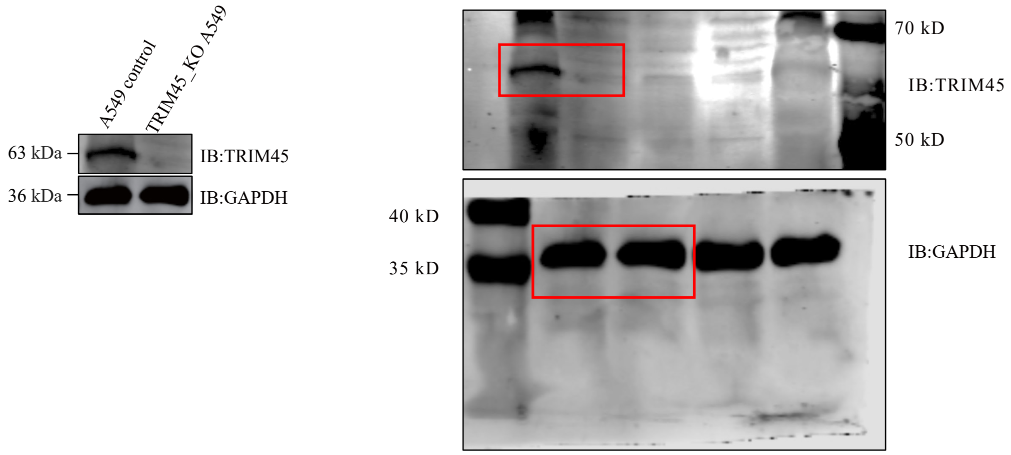

Fig 2A

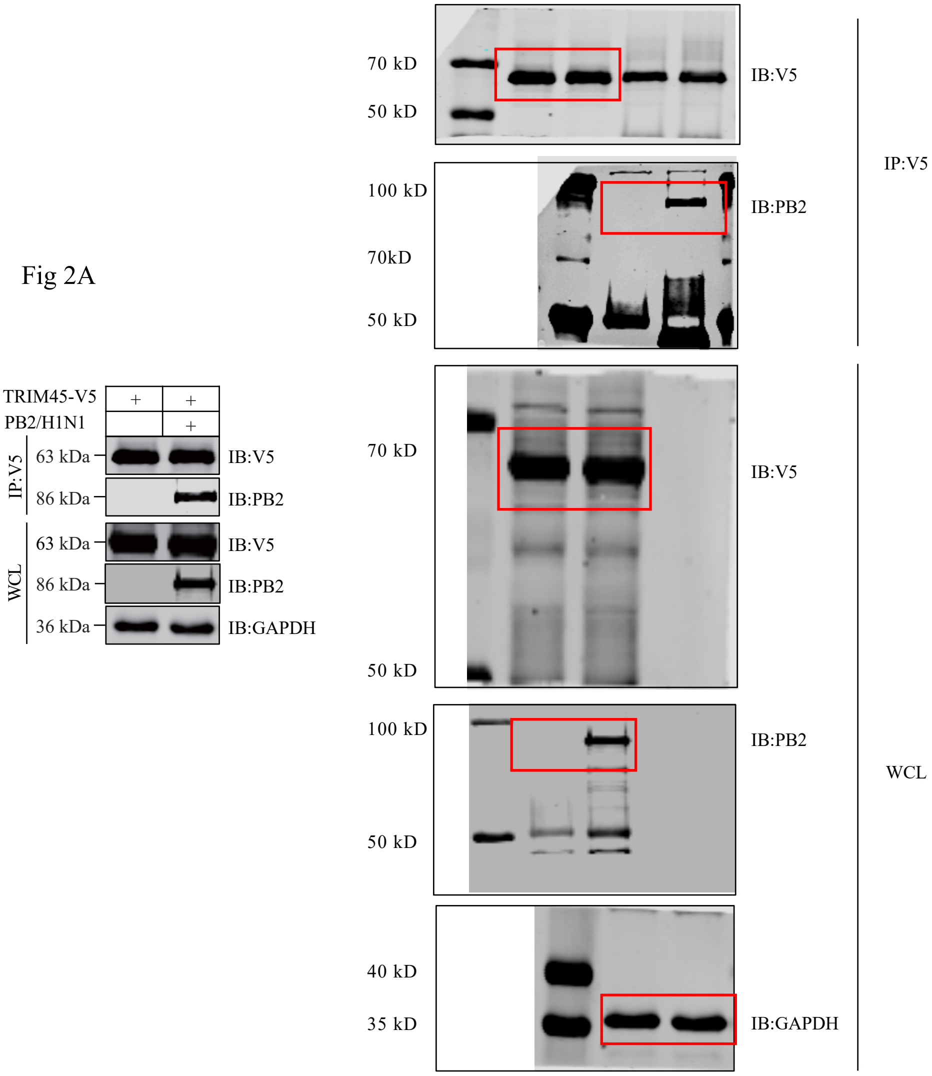
TRIM45-V5
PB2/H1N1
+
+
+
IP:V5
63 kDa
IB:V5
86 kDa
IB:PB2
WCL
63 kDa
IB:V5
86 kDa
IB:PB2
36 kDa
IB:GAPDH
70 kD
50 kD
IB:V5
100 kD
70kD
50 kD
IB:PB2
IP:V5
70 kD
IB:V5
50 kD
100 kD
IB:PB2
50 kD
WCL
40 kD
35 kD
IB:GAPDH

Fig 2A

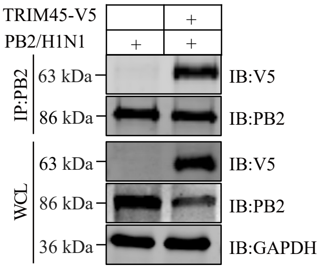

TRIM45-V5
PB2/H1N1
+
+
+
IP:PB2
63 kDa
IB:V5
86 kDa
IB:PB2
WCL
63 kDa
IB:V5
86 kDa
IB:PB2
36 kDa
IB:GAPDH

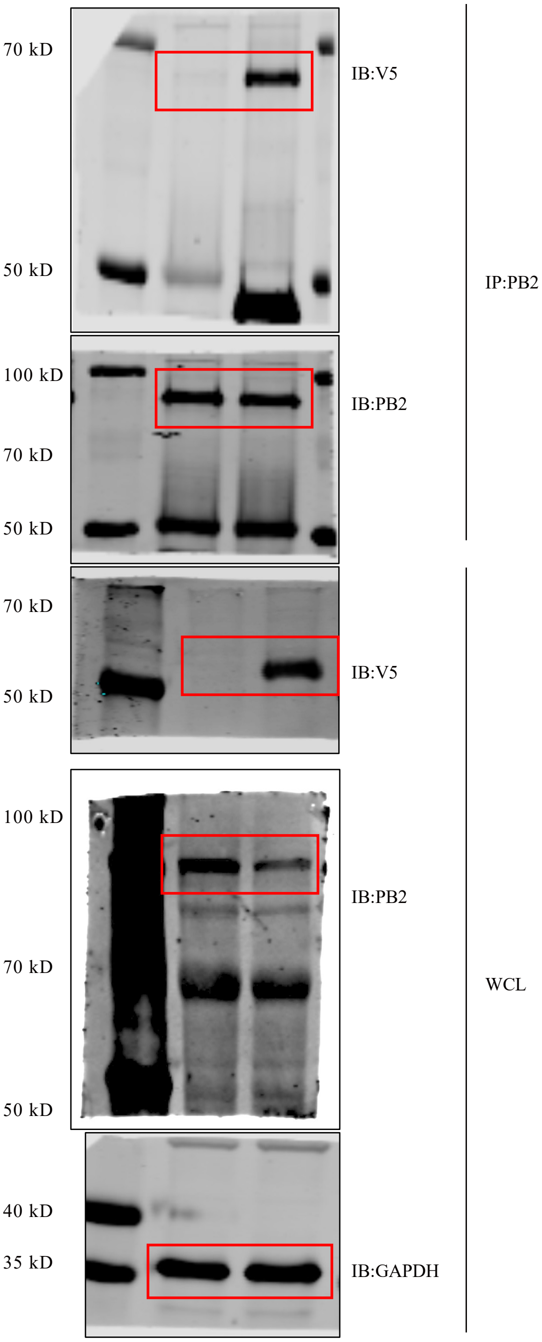

70 kD
50 kD
IB:V5
100 kD
70 kD
50 kD
IB:PB2
IP:PB2
70 kD
50 kD
IB:V5
100 kD
70 kD
50 kD
IB:PB2
40 kD
35 kD
IB:GAPDH
WCL

Fig 2B

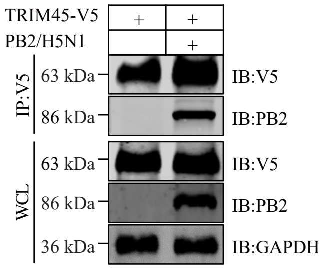
TRIM45-V5
PB2/H5N1
+
+
+
IP:V5
WCL
63 kDa
86 kDa
63 kDa
86 kDa
36 kDa
IB:V5
IB:PB2
IB:V5
IB:PB2
IB:GAPDH

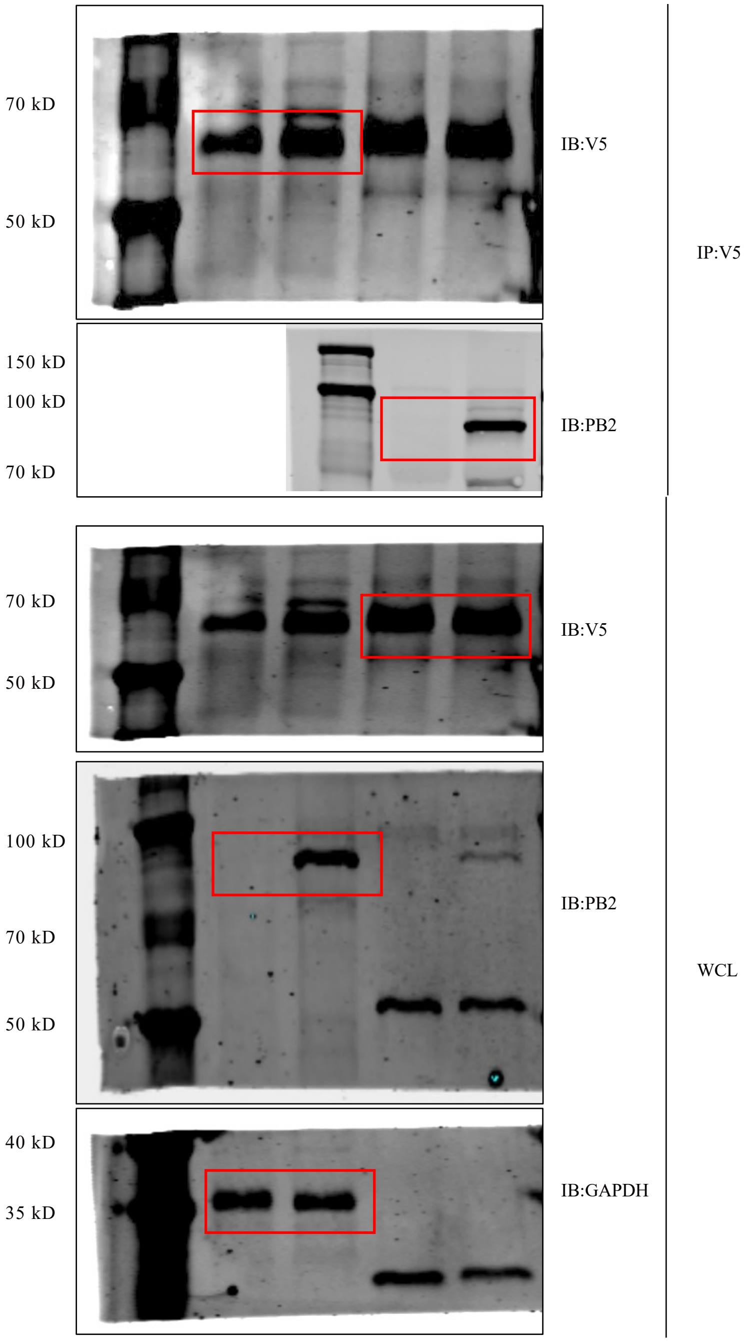
70 kD
50 kD
IB:V5
150 kD
100 kD
70 kD
IB:PB2
IP:V5
70 kD
50 kD
IB:V5
100 kD
70 kD
50 kD
IB:PB2
40 kD
35 kD
IB:GAPDH
WCL

Fig 2C

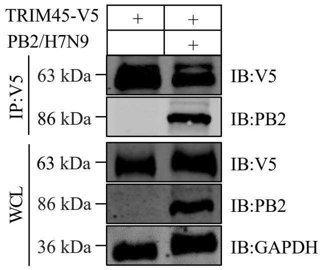

TRIM45-V5
PB2/H7N9
+
+
+
IP:V5
WCL
63 kDa
86 kDa
63 kDa
86 kDa
36 kDa
IB:V5
IB:PB2
IB:V5
IB:PB2
IB:GAPDH

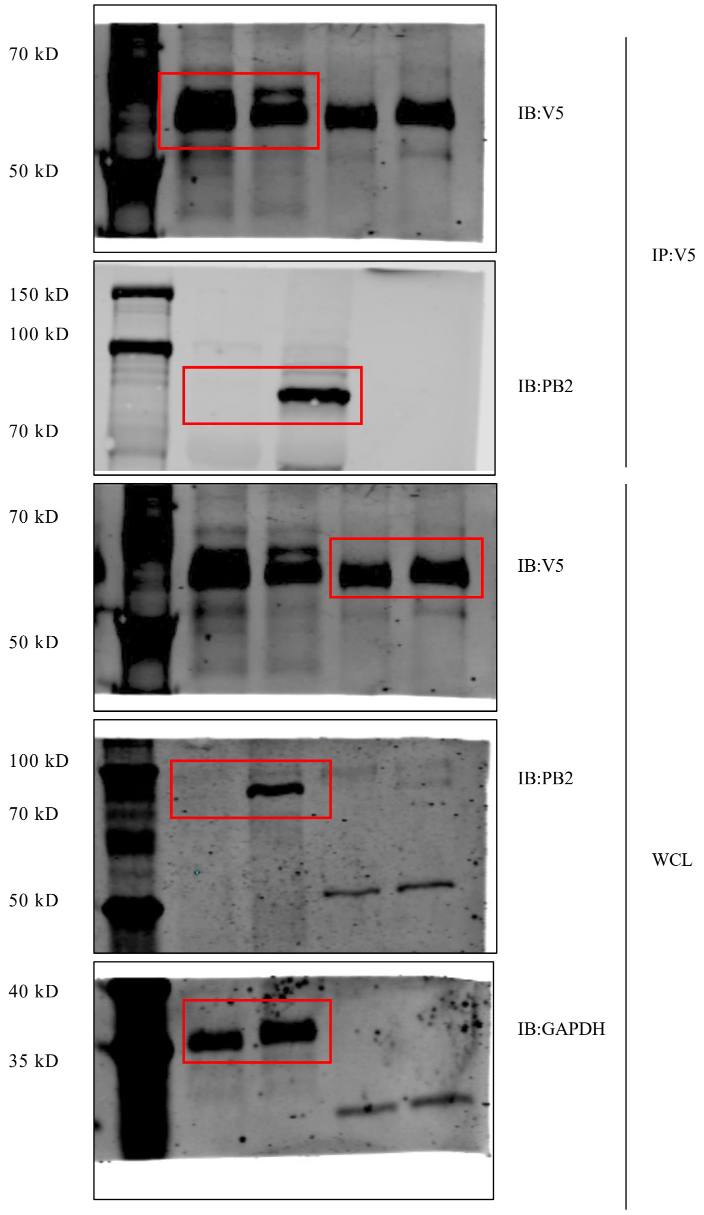

70 kD
50 kD
IB:V5
150 kD
100 kD
70 kD
IB:PB2
IP:V5
70 kD
50 kD
IB:V5
100 kD
70 kD
50 kD
IB:PB2
40 kD
35 kD
IB:GAPDH
WCL

Fig 2D

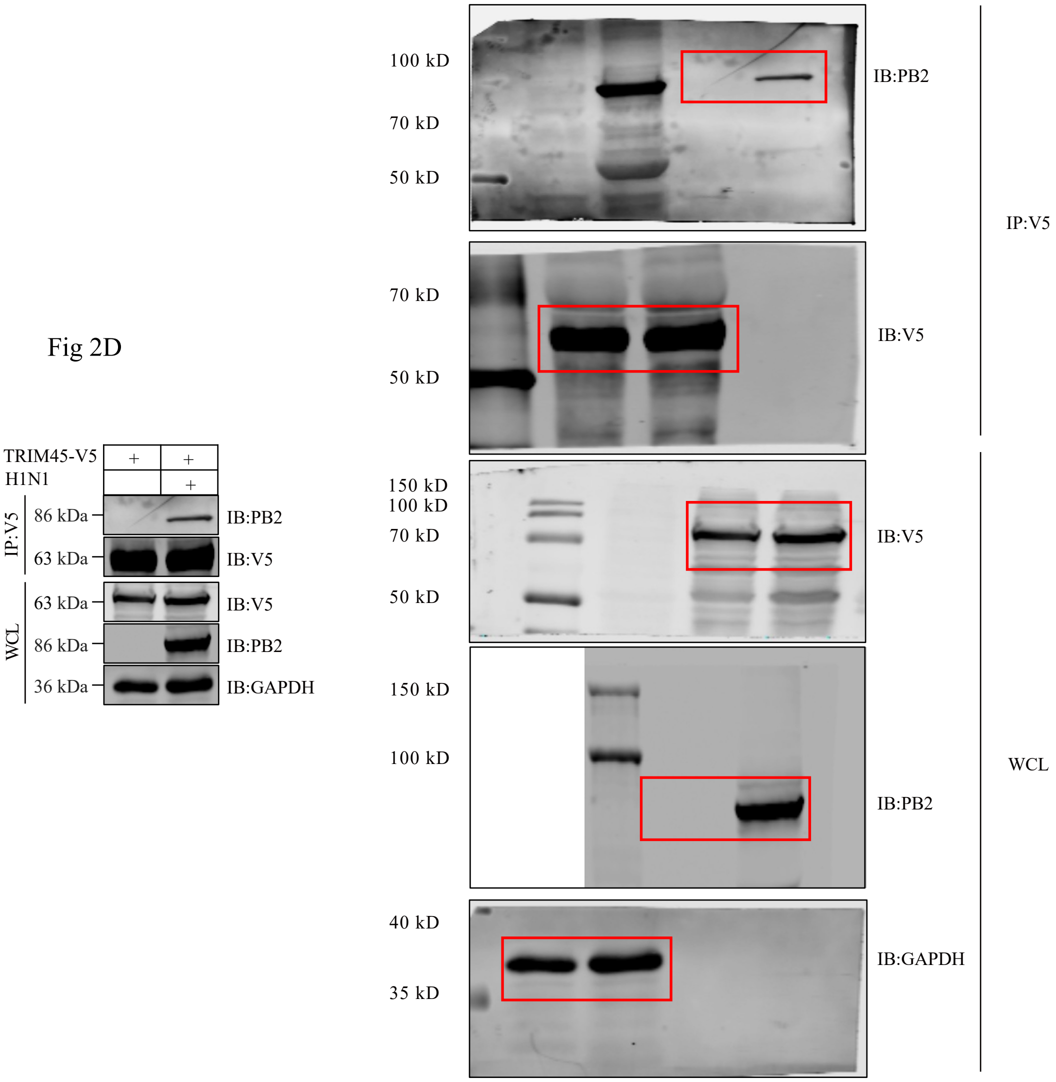

TRIM45-V5
H1N1
IP:V5
WCL
86 kDa
63 kDa
63 kDa
86 kDa
36 kDa
IB:PB2
IB:V5
IB:V5
IB:PB2
IB:GAPDH
100 kD
70 kD
50 kD
IB:PB2
70 kD
50 kD
IB:V5
IP:V5
150 kD
100 kD
70 kD
50 kD
IB:V5
150 kD
100 kD
IB:PB2
WCL
40 kD
35 kD
IB:GAPDH

Fig 2E

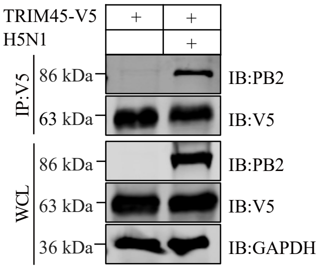
TRIM45-V5
H5N1
+
+
+
IP:V5
WCL
86 kDa
63 kDa
86 kDa
63 kDa
36 kDa
IB:PB2
IB:V5
IB:PB2
IB:V5
IB:GAPDH

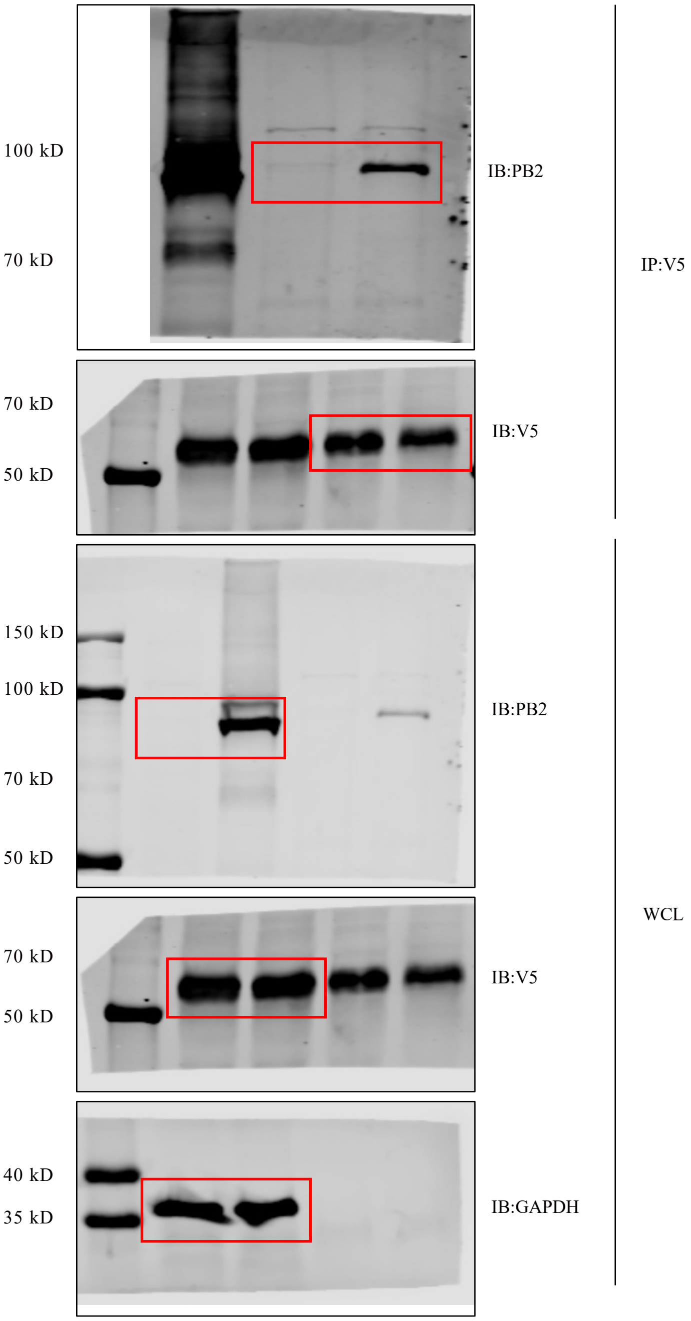
100 kD
70 kD
IB:PB2
IP:V5
70 kD
50 kD
IB:V5
150 kD
100 kD
70 kD
50 kD
IB:PB2
70 kD
50 kD
IB:V5
WCL
40 kD
35 kD
IB:GAPDH

Fig 2F

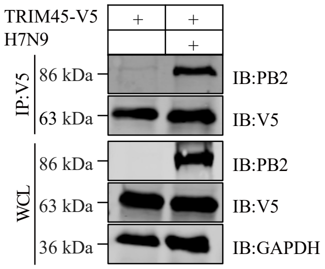
TRIM45-V5
H7N9
+
+
+
IP:V5
86 kDa
IB:PB2
63 kDa
IB:V5
WCL
86 kDa
IB:PB2
63 kDa
IB:V5
36 kDa
IB:GAPDH

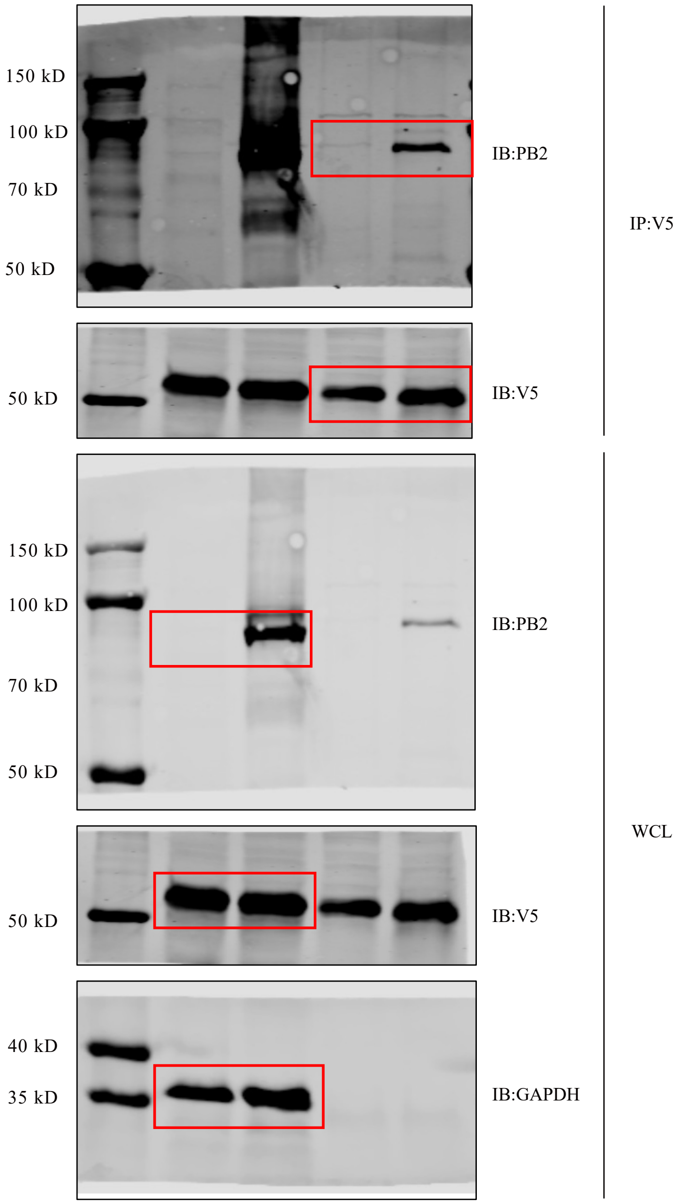
150 kD
100 kD
70 kD
50 kD
IB:PB2
50 kD
IB:V5
IP:V5
150 kD
100 kD
70 kD
50 kD
IB:PB2
50 kD
IB:V5
40 kD
35 kD
IB:GAPDH
WCL

Fig 2I

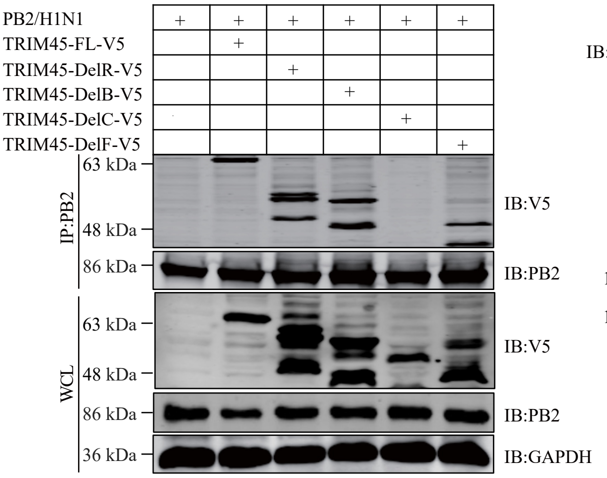

PB2/H1N1
TRIM45-FL-V5
TRIM45-DelR-V5
TRIM45-DelB-V5
TRIM45-DelC-V5
TRIM45-DelF-V5
IP:PB2
WCL
63 kDa
48 kDa
86 kDa
63 kDa
48 kDa
86 kDa
36 kDa
IB:V5
IB:PB2
IB:V5
IB:PB2
IB:GAPDH

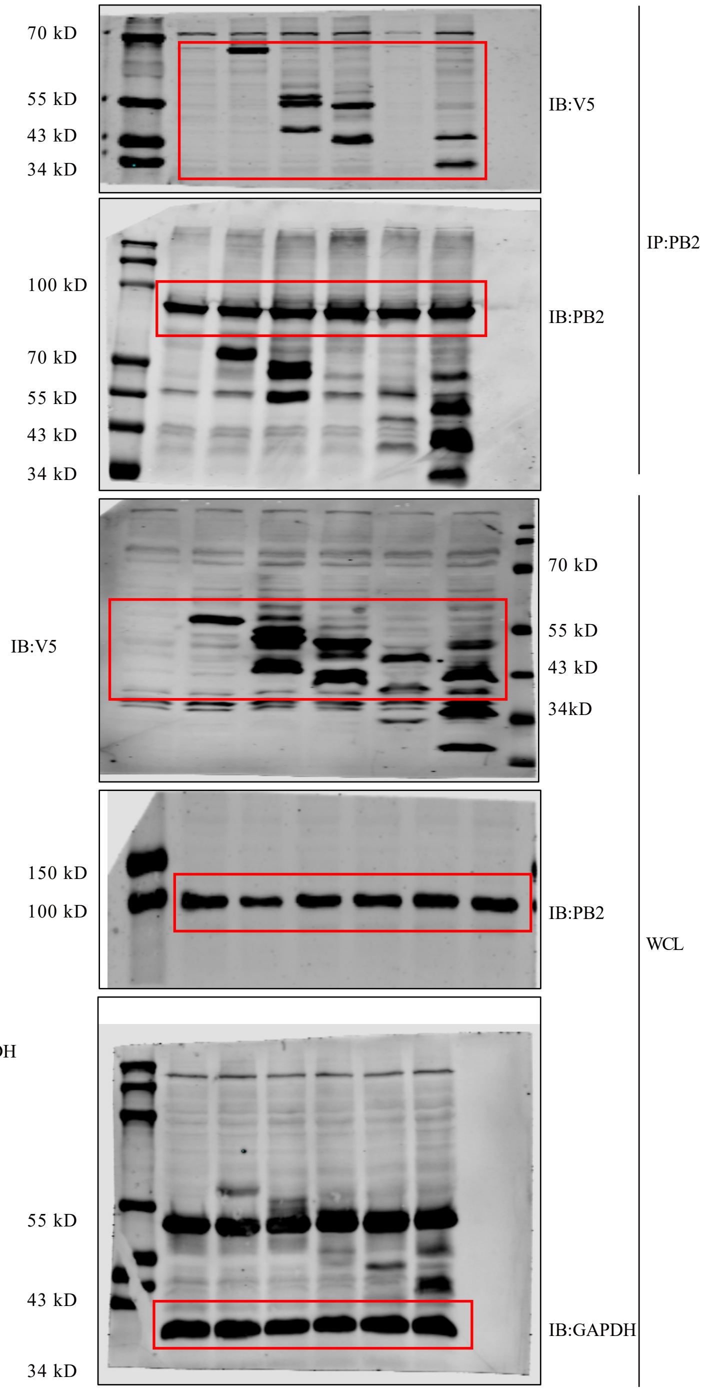

70 kD
55 kD
43 kD
34 kD
IB:V5
100 kD
70 kD
55 kD
43 kD
34 kD
IB:PB2
IP:PB2
IB:V5
70 kD
55 kD
43 kD
34kD
150 kD
100 kD
IB:PB2
WCL
55 kD
43 kD
34 kD
IB:GAPDH

Fig 2J

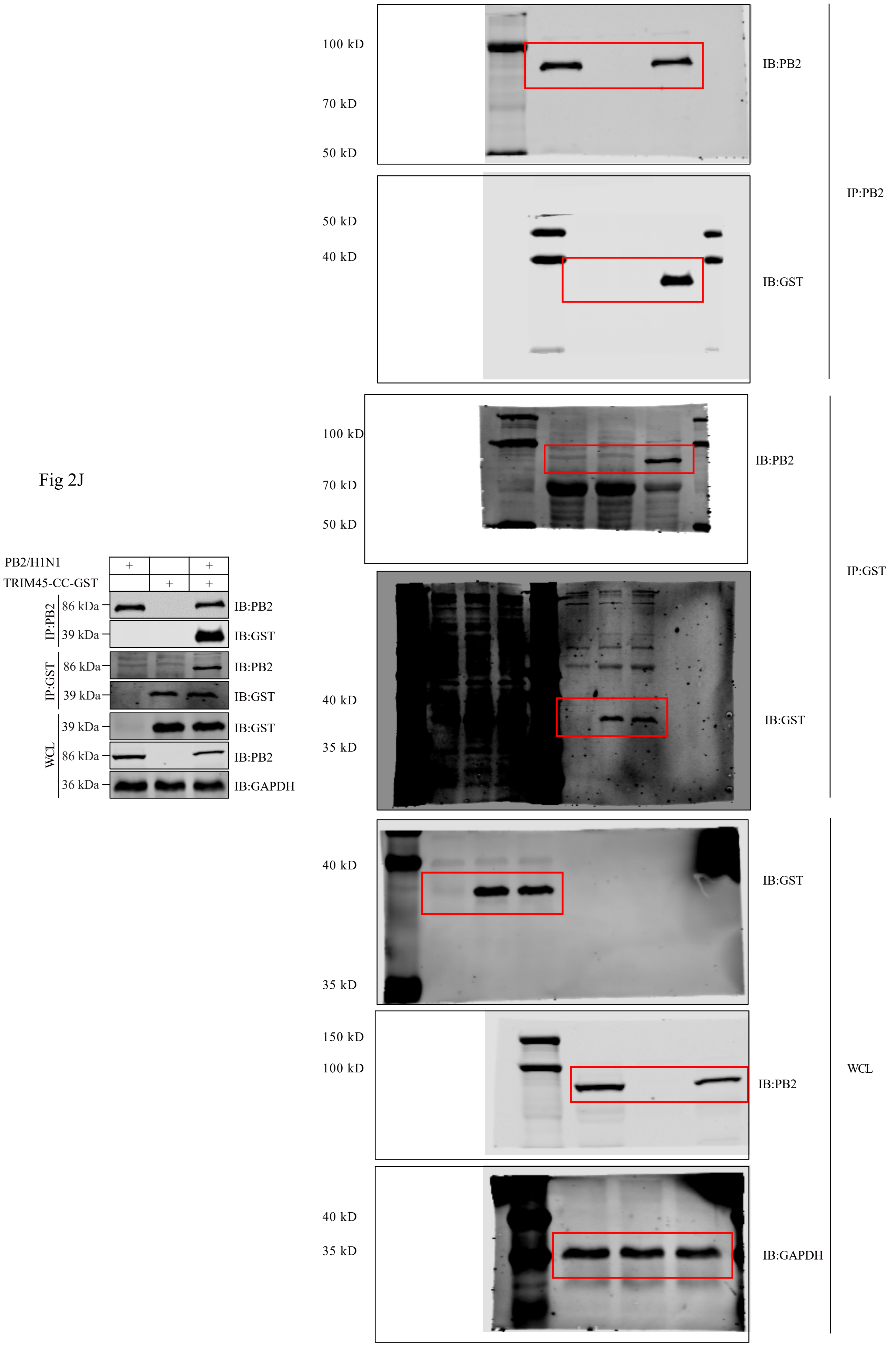

PB2/H1N1
TRIM45-CC-GST
IP:PB2
IP:GST
WCL
86 kDa
39 kDa
36 kDa
IB:PB2
IB:GST
IB:GAPDH
100 kD
70 kD
50 kD
40 kD
35 kD
150 kD

Fig 3A

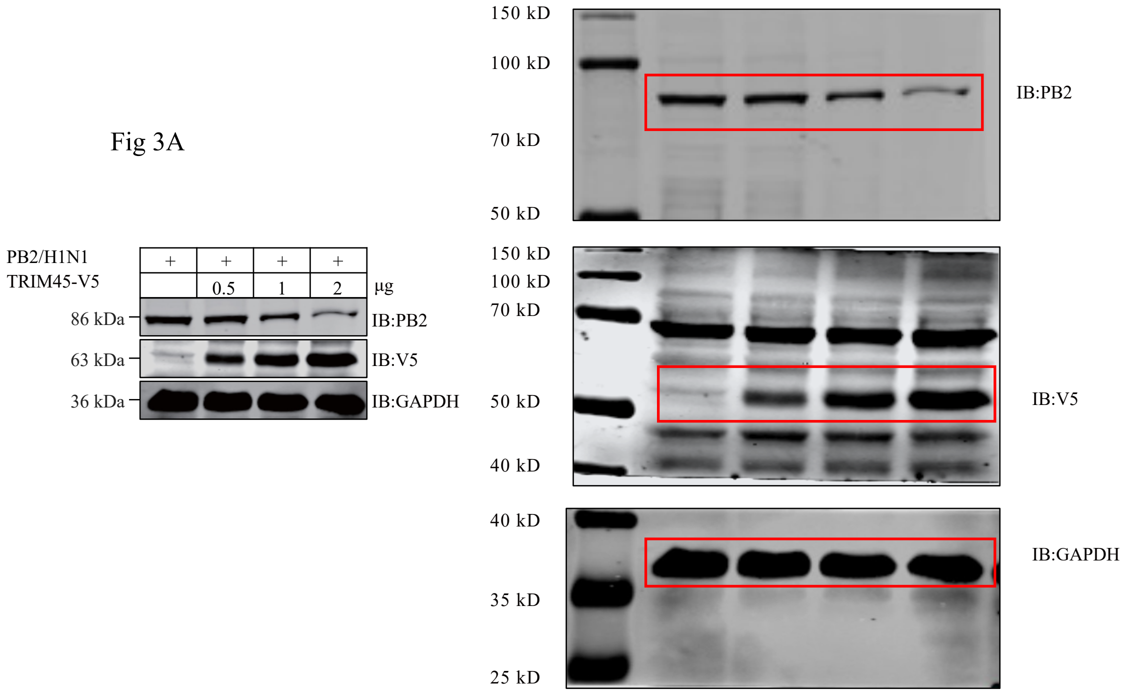
PB2/H1N1
TRIM45-V5
+ + + +
0.5 1 2
μg
86 kDa
63 kDa
36 kDa
IB:PB2
IB:V5
IB:GAPDH
150 kD
100 kD
70 kD
50 kD
IB:PB2
150 kD
100 kD
70 kD
50 kD
40 kD
IB:V5
40 kD
35 kD
25 kD
IB:GAPDH

Fig 3B

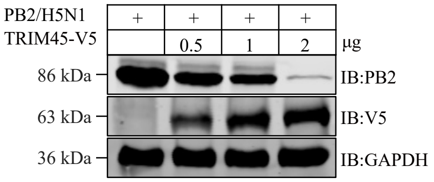

PB2/H5N1
TRIM45-V5
+ + + +
0.5 1 2 μg
86 kDa
63 kDa
36 kDa
IB:PB2
IB:V5
IB:GAPDH

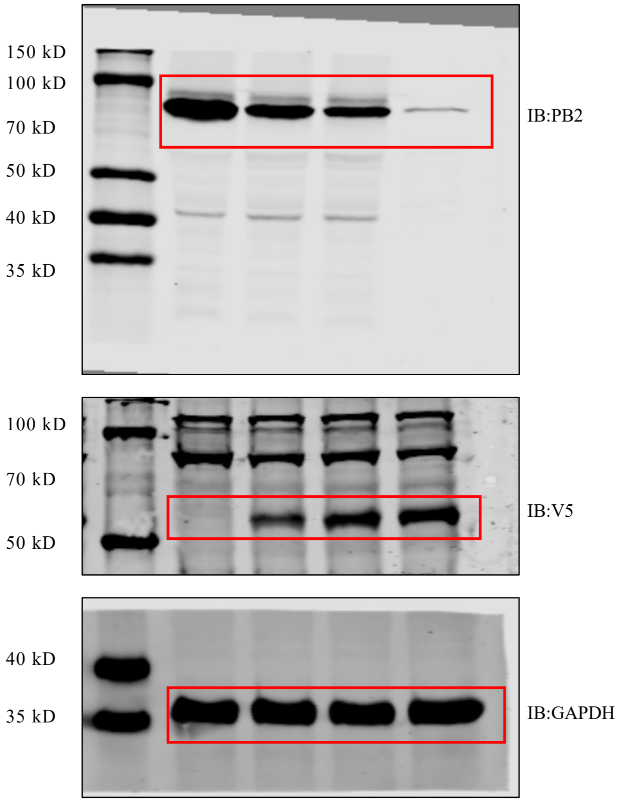

150 kD
100 kD
70 kD
50 kD
40 kD
35 kD
IB:PB2
100 kD
70 kD
50 kD
IB:V5
40 kD
35 kD
IB:GAPDH

Fig 3C

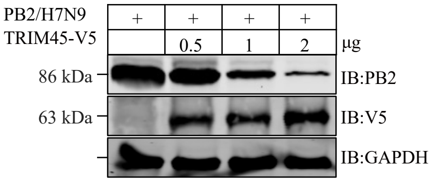

PB2/H7N9
TRIM45-V5
+ + + +
0.5 1 2 μg
86 kDa
IB:PB2
63 kDa
IB:V5
IB:GAPDH

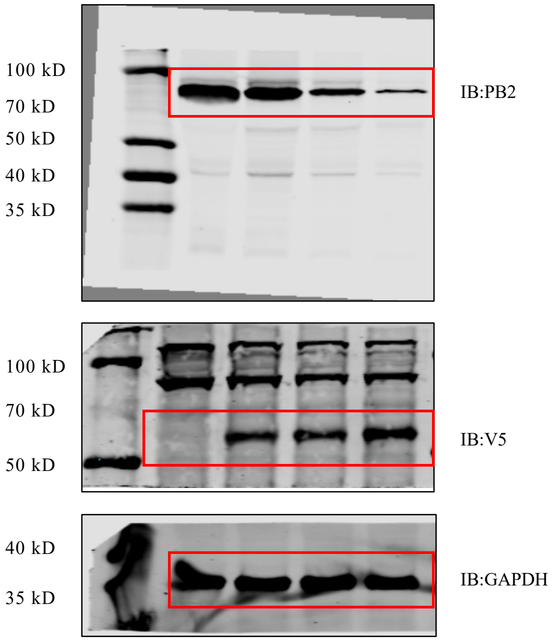

100 kD
70 kD
50 kD
40 kD
35 kD
IB:PB2
100 kD
70 kD
50 kD
IB:V5
40 kD
35 kD
IB:GAPDH

Fig 3D

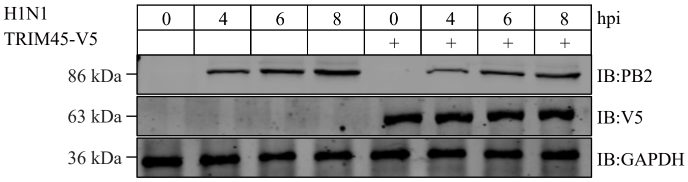

H1N1
TRIM45-V5
0
4
6
8
0
4
6
8
hpi
+
+
+
+
86 kDa
63 kDa
36 kDa
IB:PB2
IB:V5
IB:GAPDH

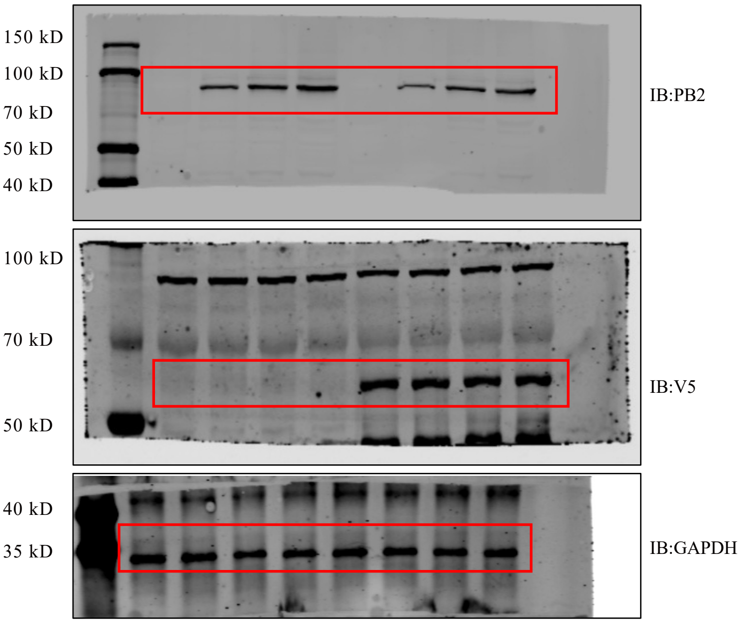

150 kD
100 kD
70 kD
50 kD
40 kD
IB:PB2
100 kD
70 kD
50 kD
IB:V5
40 kD
35 kD
IB:GAPDH

Fig 3E

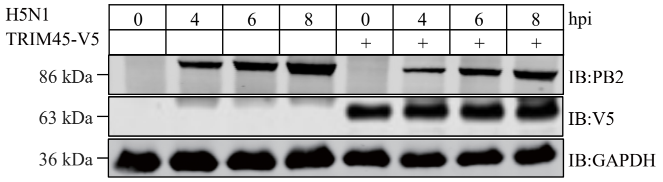
H5N1
TRIM45-V5
0
4
6
8
0
4
6
8
hpi
+
+
+
+
86 kDa
63 kDa
36 kDa
IB:PB2
IB:V5
IB:GAPDH

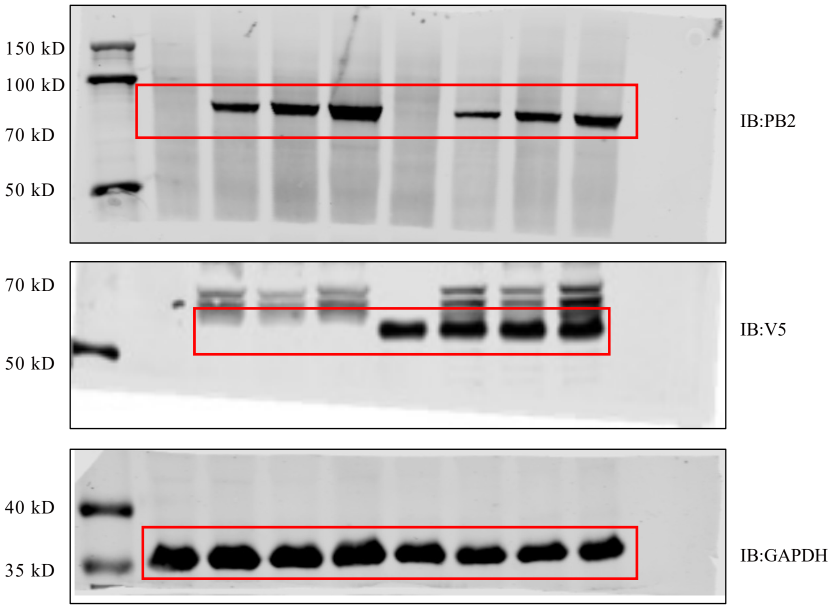
150 kD
100 kD
70 kD
50 kD
IB:PB2
70 kD
50 kD
IB:V5
40 kD
35 kD
IB:GAPDH

Fig 3F

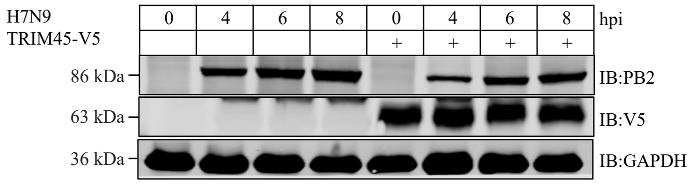

H7N9
TRIM45-V5
0
4
6
8
0
4
6
8
hpi
+
+
+
+
86 kDa
63 kDa
36 kDa
IB:PB2
IB:V5
IB:GAPDH

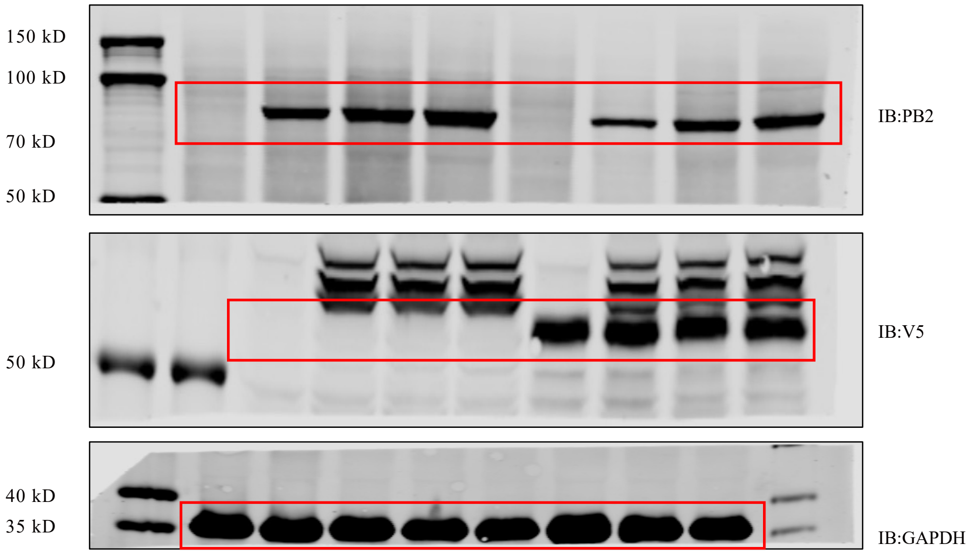

150 kD
100 kD
70 kD
50 kD
IB:PB2
50 kD
IB:V5
40 kD
35 kD
IB:GAPDH

Fig 3G

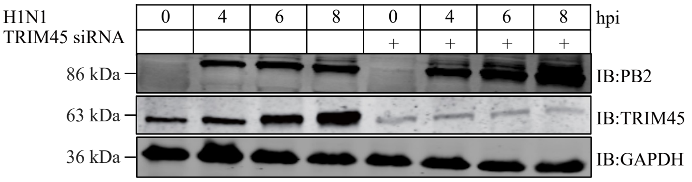
H1N1
TRIM45 siRNA
0
4
6
8
0
4
6
8
hpi
+
+
+
+
86 kDa
63 kDa
36 kDa
IB:PB2
IB:TRIM45
IB:GAPDH

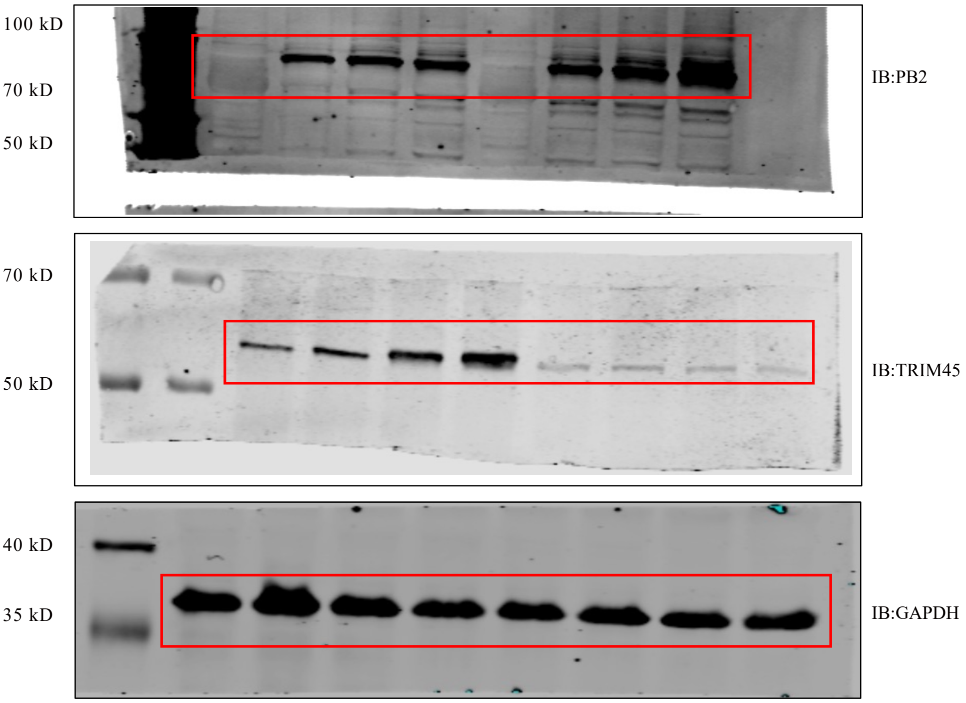
100 kD
70 kD
50 kD
IB:PB2
70 kD
50 kD
IB:TRIM45
40 kD
35 kD
IB:GAPDH

Fig 3H

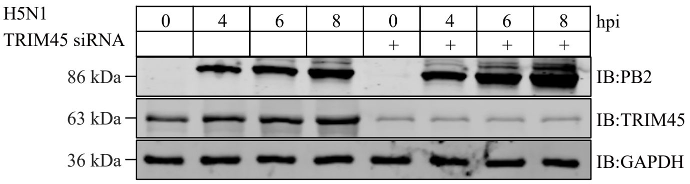
H5N1
TRIM45 siRNA
0
4
6
8
0
4
6
8
hpi
+
+
+
+
86 kDa
63 kDa
36 kDa
IB:PB2
IB:TRIM45
IB:GAPDH

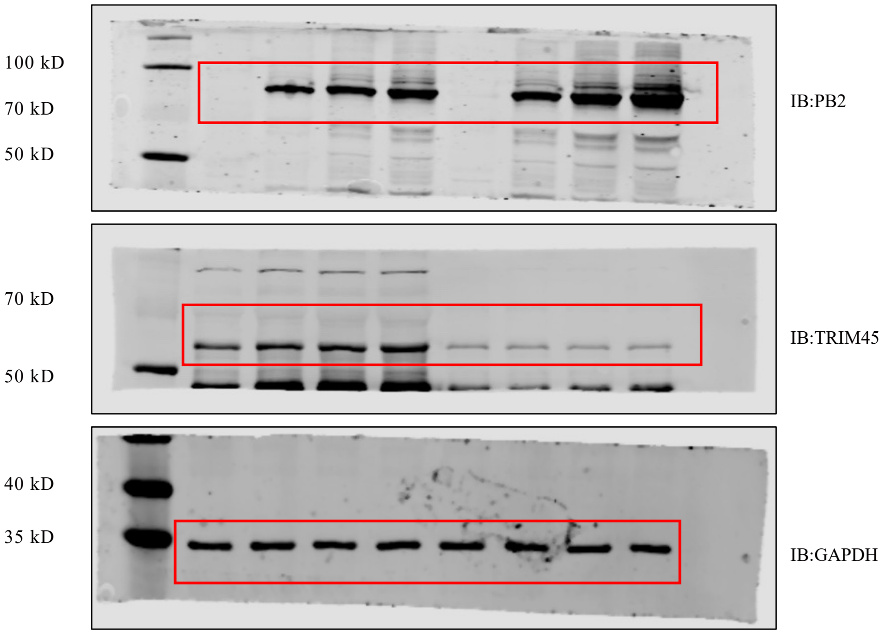
100 kD
70 kD
50 kD
IB:PB2
70 kD
50 kD
IB:TRIM45
40 kD
35 kD
IB:GAPDH

Fig 3I

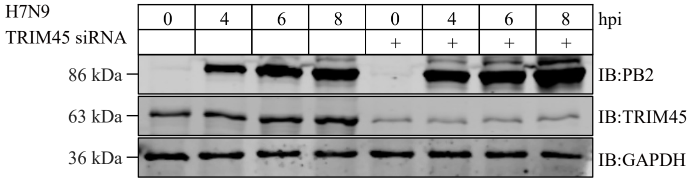
H7N9
TRIM45 siRNA
0
4
6
8
0
4
6
8
hpi
+
+
+
+
86 kDa
63 kDa
36 kDa
IB:PB2
IB:TRIM45
IB:GAPDH

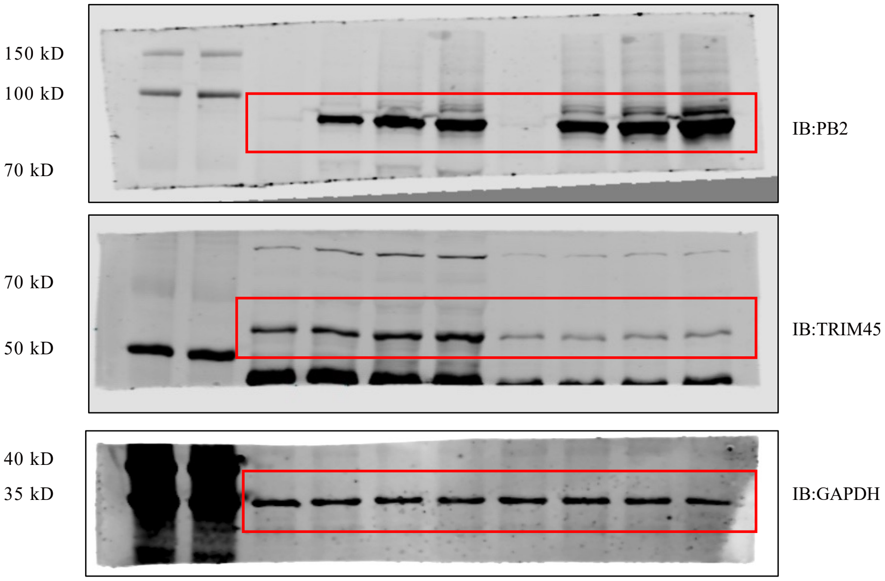
150 kD
100 kD
70 kD
IB:PB2
70 kD
50 kD
IB:TRIM45
40 kD
35 kD
IB:GAPDH

Fig 4A

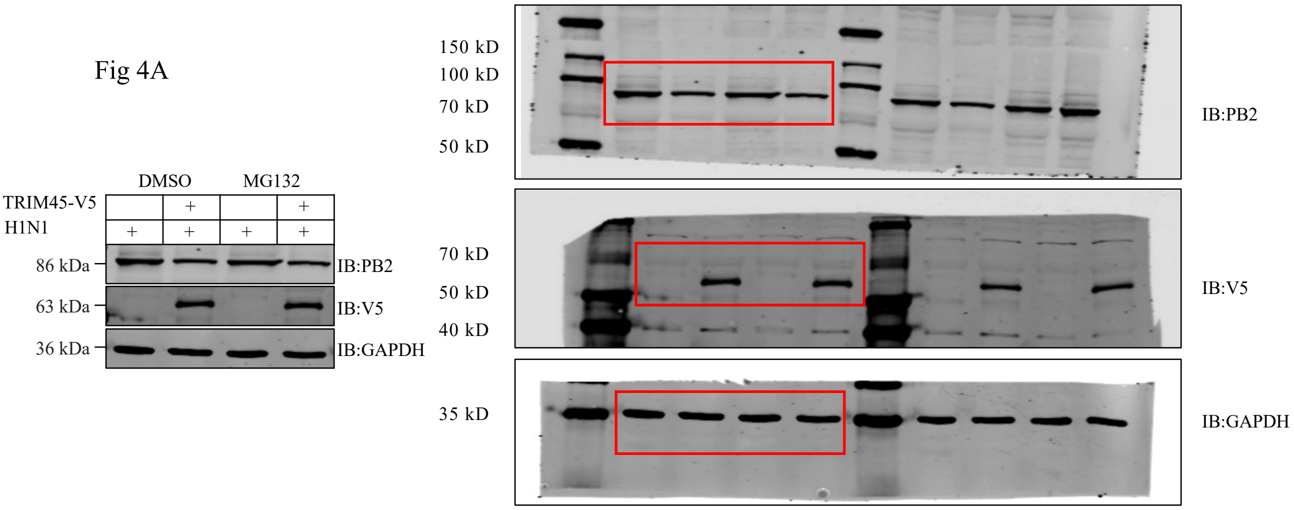
DMSO
MG132
TRIM45-V5
H1N1
+
86 kDa
63 kDa
36 kDa
IB:PB2
IB:V5
IB:GAPDH
150 kD
100 kD
70 kD
50 kD
70 kD
50 kD
40 kD
35 kD
IB:PB2
IB:V5
IB:GAPDH

Fig 4B

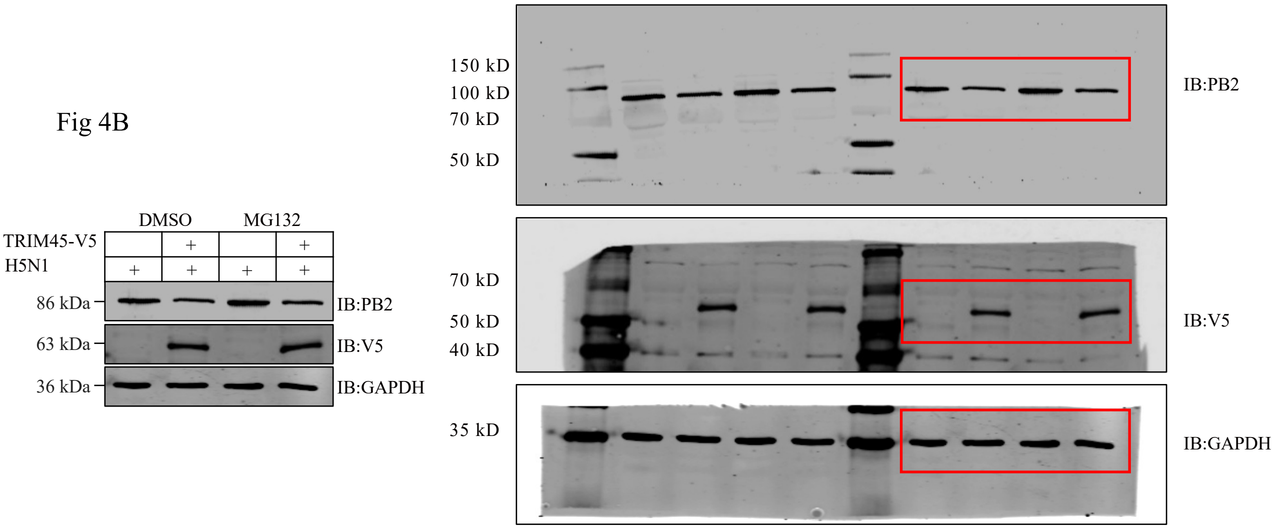
DMSO
MG132
TRIM45-V5
H5N1
+
86 kDa
63 kDa
36 kDa
IB:PB2
IB:V5
IB:GAPDH
150 kD
100 kD
70 kD
50 kD
70 kD
50 kD
40 kD
35 kD
IB:PB2
IB:V5
IB:GAPDH

Fig 4C

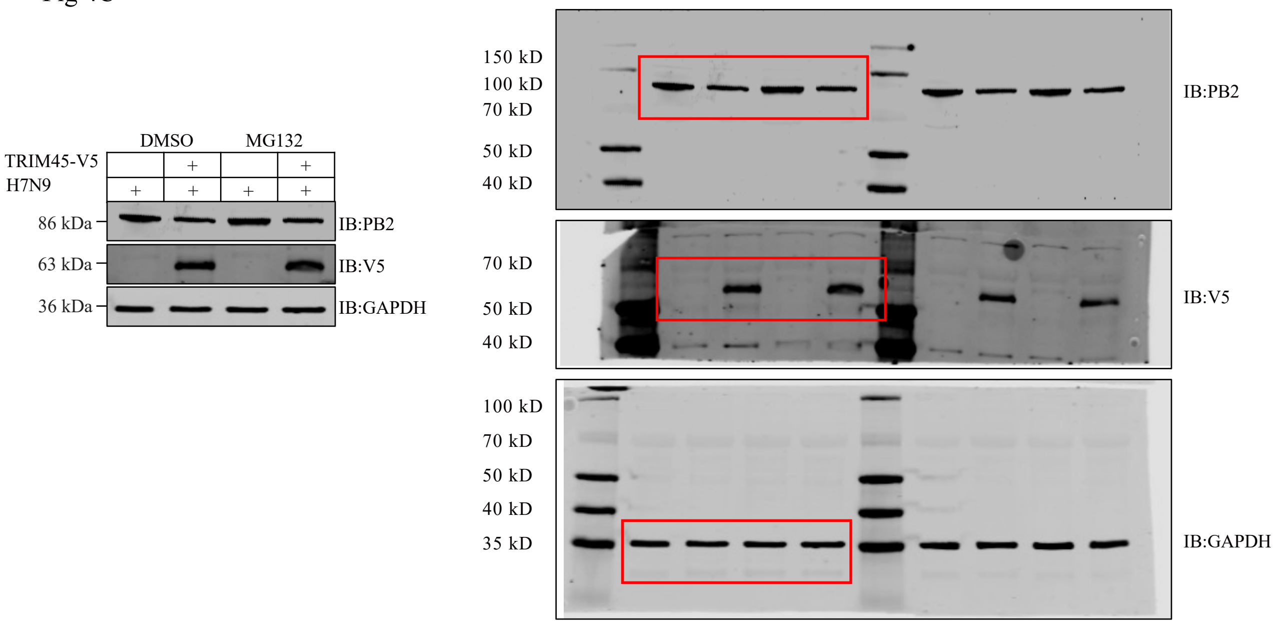
DMSO
MG132
TRIM45-V5
H7N9
+
+
+
+
+
+
86 kDa
IB:PB2
63 kDa
IB:V5
36 kDa
IB:GAPDH
150 kD
100 kD
70 kD
50 kD
40 kD
IB:PB2
70 kD
50 kD
40 kD
IB:V5
100 kD
70 kD
50 kD
40 kD
35 kD
IB:GAPDH

Fig 4D

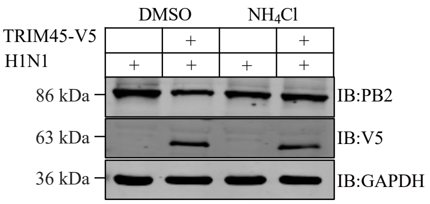

DMSO
NH4Cl
TRIM45-V5
H1N1
+
+
+
+
+
+
86 kDa
63 kDa
36 kDa
IB:PB2
IB:V5
IB:GAPDH

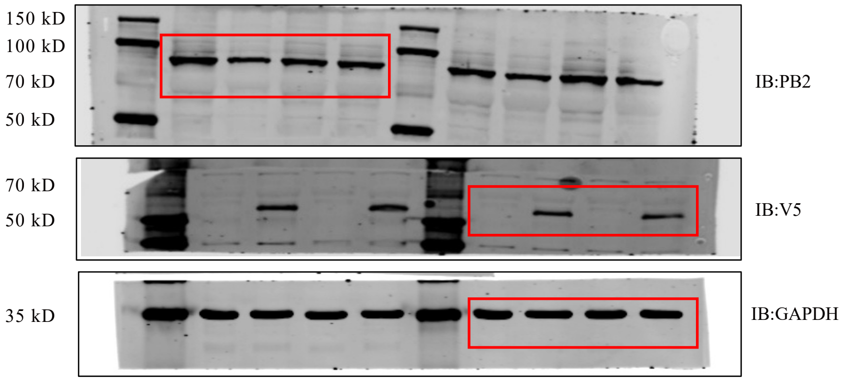

150 kD
100 kD
70 kD
50 kD
IB:PB2
70 kD
50 kD
IB:V5
35 kD
IB:GAPDH

Fig 4E

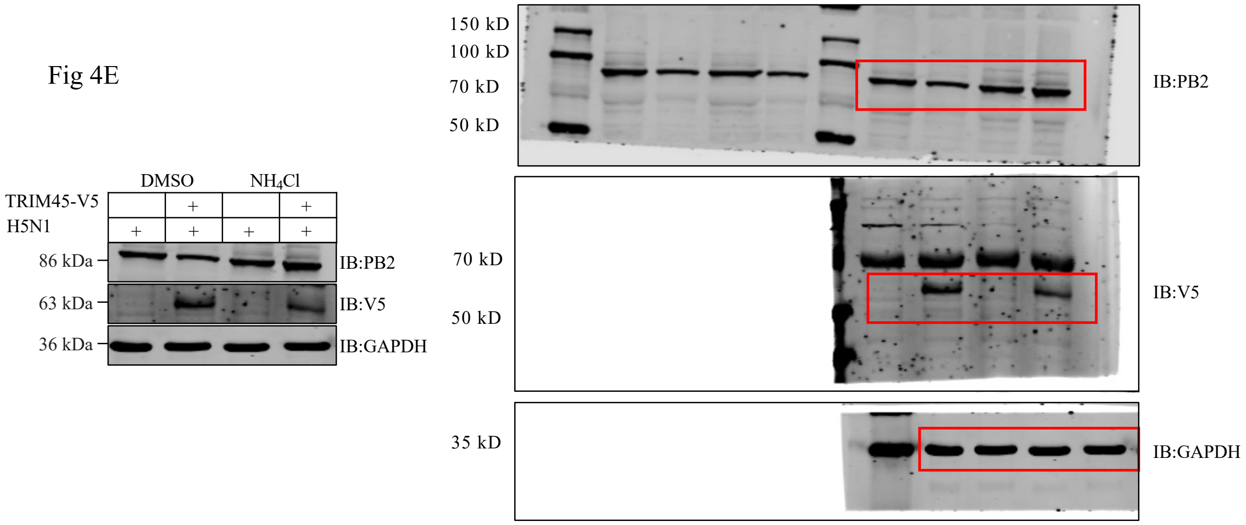
DMSO
NH4Cl
TRIM45-V5
H5N1
86 kDa
63 kDa
36 kDa
IB:PB2
IB:V5
IB:GAPDH
150 kD
100 kD
70 kD
50 kD
IB:PB2
70 kD
50 kD
IB:V5
35 kD
IB:GAPDH

Fig 4F

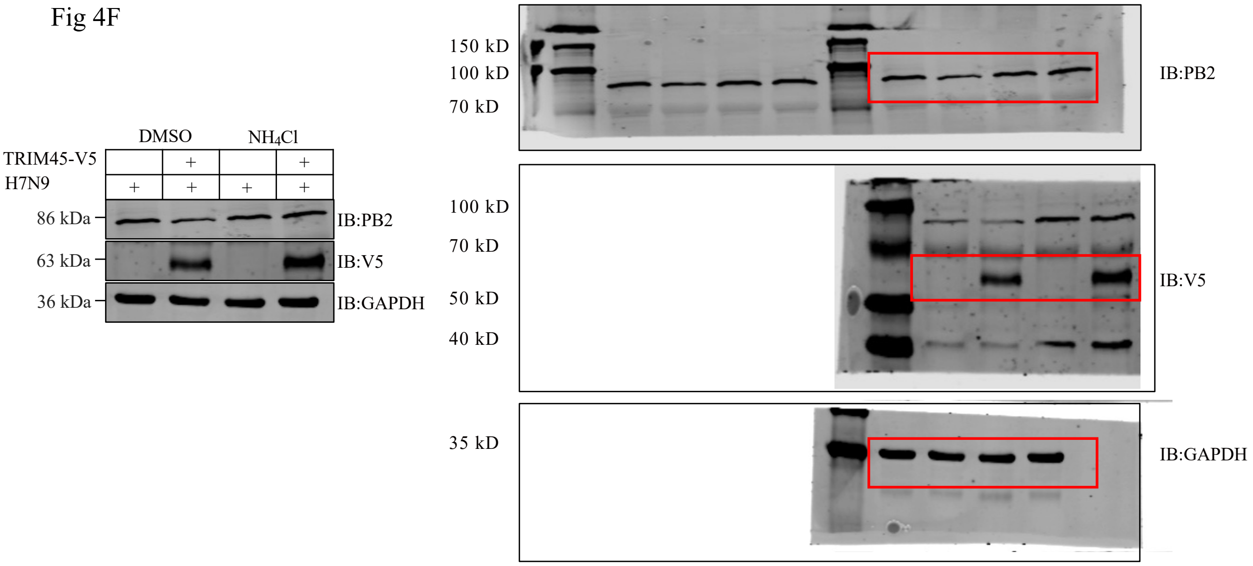

DMSO
NH4Cl
TRIM45-V5
H7N9
+
+
+
+
+
+
86 kDa
63 kDa
36 kDa
IB:PB2
IB:V5
IB:GAPDH
150 kD
100 kD
70 kD
IB:PB2
100 kD
70 kD
50 kD
40 kD
IB:V5
35 kD
IB:GAPDH

Fig 4H

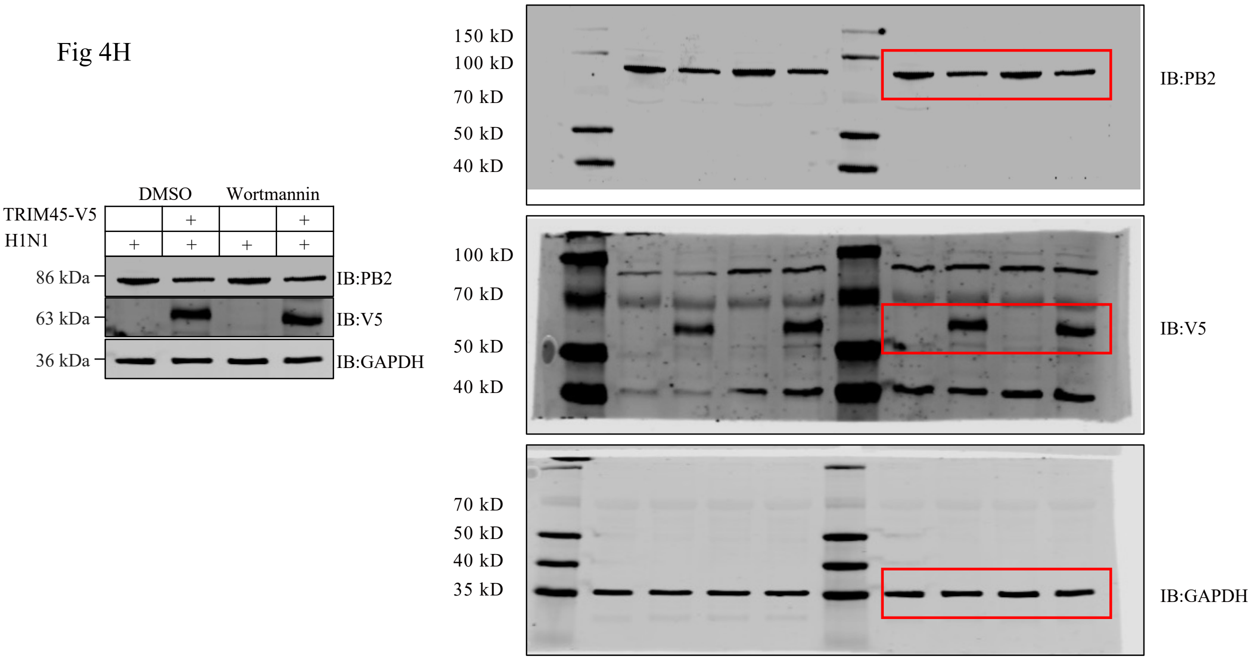
DMSO
Wortmannin
TRIM45-V5
H1N1
86 kDa
63 kDa
36 kDa
IB:PB2
IB:V5
IB:GAPDH
150 kD
100 kD
70 kD
50 kD
40 kD
IB:PB2
100 kD
70 kD
50 kD
40 kD
IB:V5
70 kD
50 kD
40 kD
35 kD
IB:GAPDH

Fig 4I

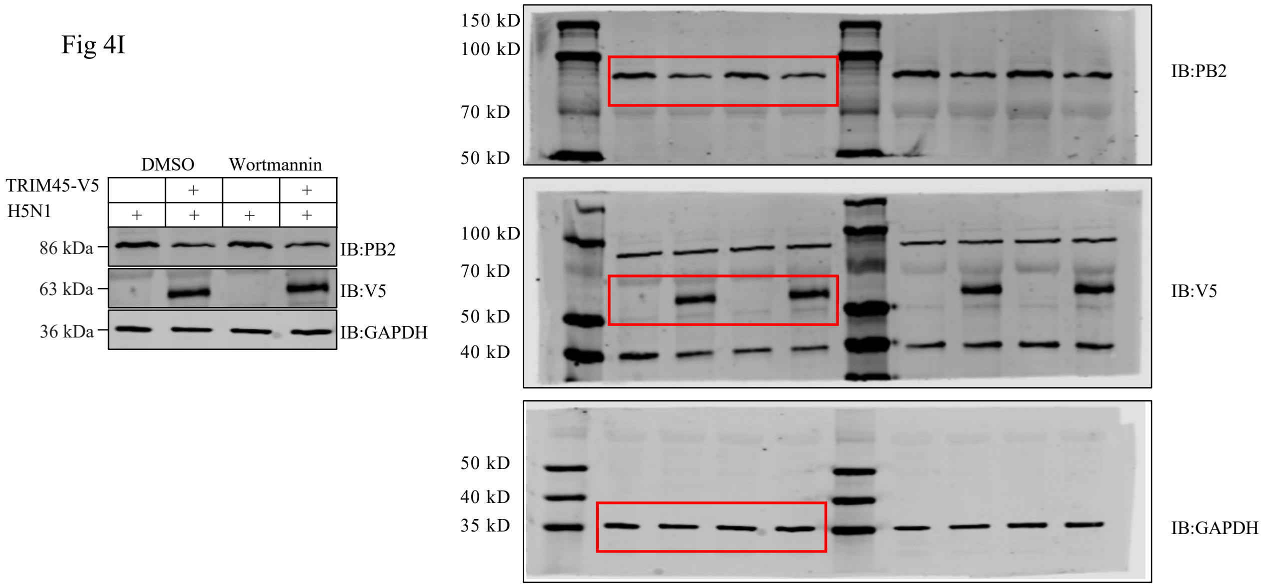
DMSO
Wortmannin
TRIM45-V5
H5N1
86 kDa
63 kDa
36 kDa
IB:PB2
IB:V5
IB:GAPDH
150 kD
100 kD
70 kD
50 kD
100 kD
70 kD
50 kD
40 kD
50 kD
40 kD
35 kD
IB:PB2
IB:V5
IB:GAPDH

Fig 4J

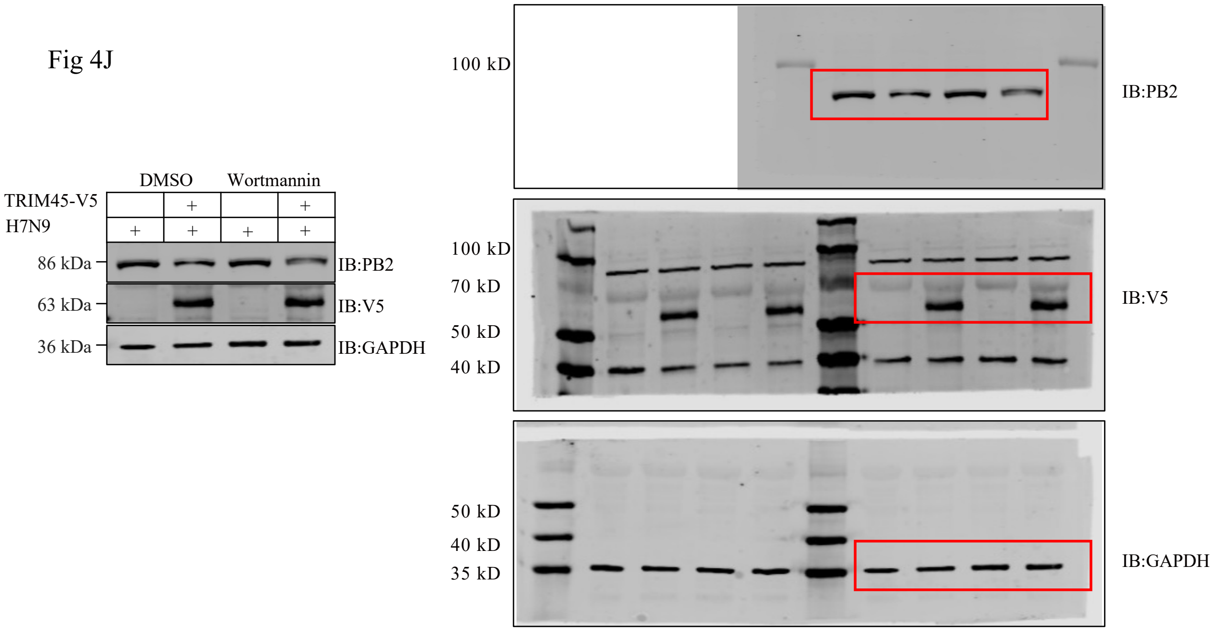

DMSO
Wortmannin
TRIM45-V5
H7N9
86 kDa
63 kDa
36 kDa
IB:PB2
IB:V5
IB:GAPDH
100 kD
100 kD
70 kD
50 kD
40 kD
50 kD
40 kD
35 kD
IB:PB2
IB:V5
IB:GAPDH

Fig 4K

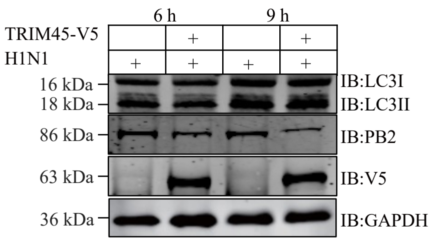
6 h
9 h
TRIM45-V5
H1N1
+
16 kDa
18 kDa
86 kDa
63 kDa
36 kDa
IB:LC3I
IB:LC3II
IB:PB2
IB:V5
IB:GAPDH

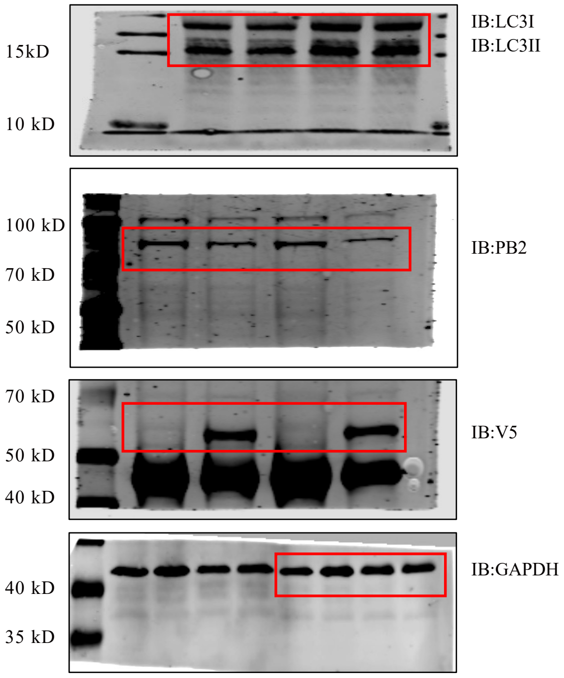
15kD
10 kD
IB:LC3I
IB:LC3II
100 kD
70 kD
50 kD
IB:PB2
70 kD
50 kD
40 kD
IB:V5
40 kD
35 kD
IB:GAPDH

Fig 5A

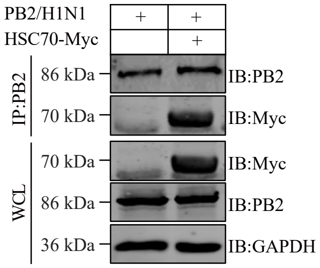
PB2/H1N1
HSC70-Myc
+
+
+
IP:PB2
86 kDa
IB:PB2
70 kDa
IB:Myc
WCL
70 kDa
IB:Myc
86 kDa
IB:PB2
36 kDa
IB:GAPDH

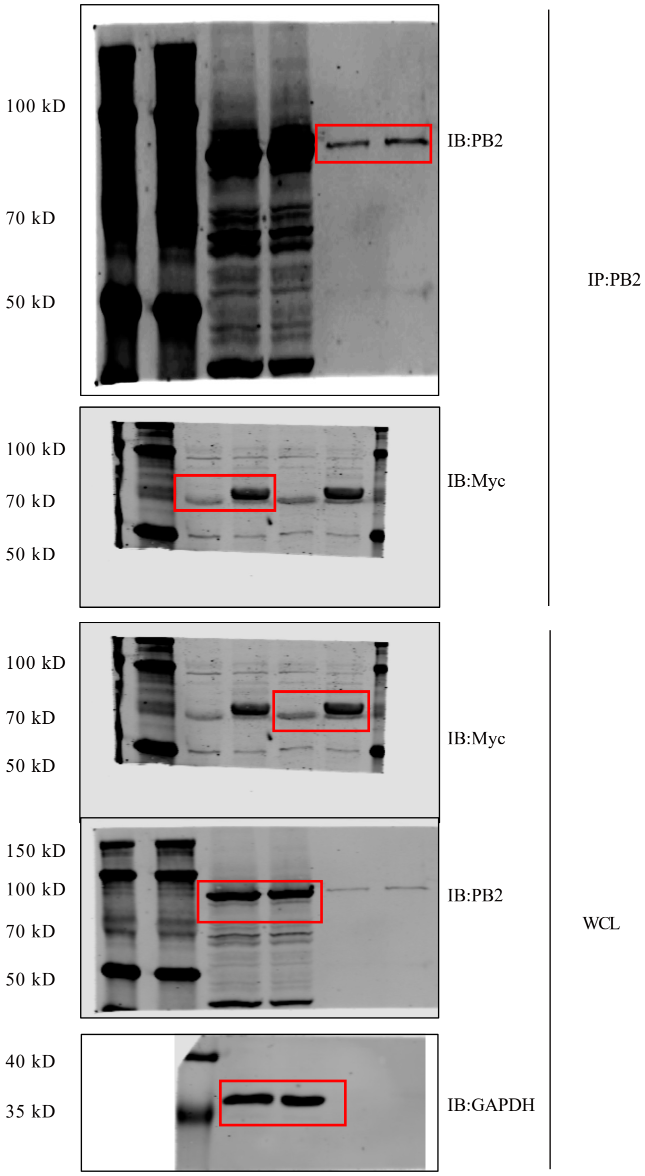
100 kD
70 kD
50 kD
IB:PB2
IP:PB2
100 kD
70 kD
50 kD
IB:Myc
100 kD
70 kD
50 kD
IB:Myc
150 kD
100 kD
70 kD
50 kD
IB:PB2
WCL
40 kD
35 kD
IB:GAPDH

Fig 5B

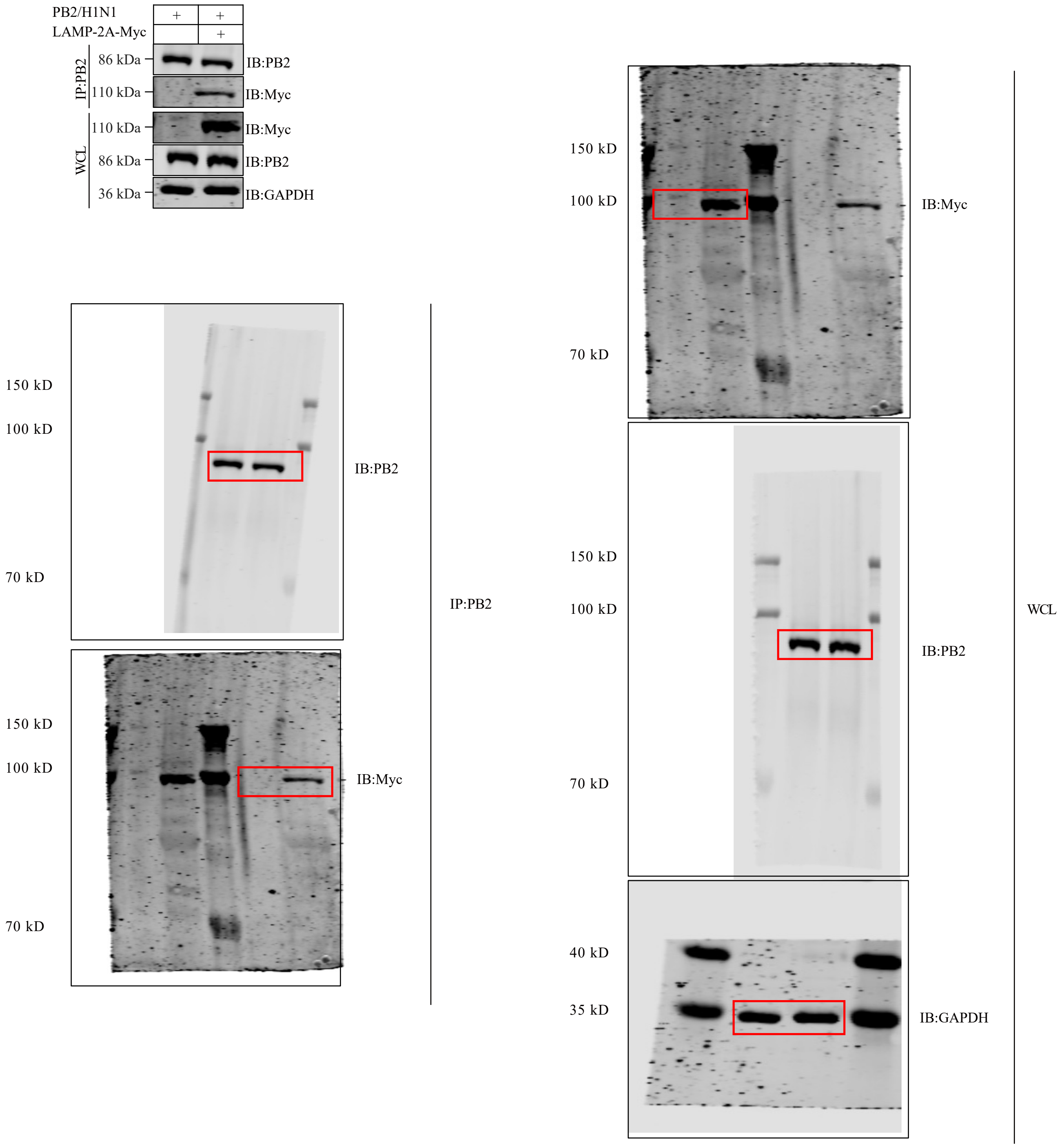

PB2/H1N1
LAMP-2A-Myc
+
+
+
IP:PB2
86 kDa
110 kDa
IB:PB2
IB:Myc
WCL
110 kDa
86 kDa
36 kDa
IB:Myc
IB:PB2
IB:GAPDH
150 kD
100 kD
70 kD
IB:PB2
150 kD
100 kD
70 kD
IB:Myc
IP:PB2
150 kD
100 kD
70 kD
IB:Myc
150 kD
100 kD
70 kD
IB:PB2
40 kD
35 kD
IB:GAPDH
WCL

Fig 5C

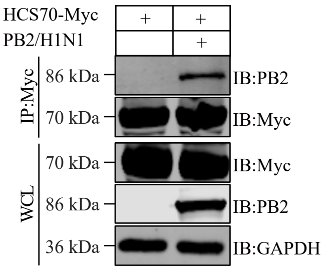

HCS70-Myc
PB2/H1N1
+
+
+
IP:Myc
86 kDa
IB:PB2
70 kDa
IB:Myc
WCL
70 kDa
IB:Myc
86 kDa
IB:PB2
36 kDa
IB:GAPDH

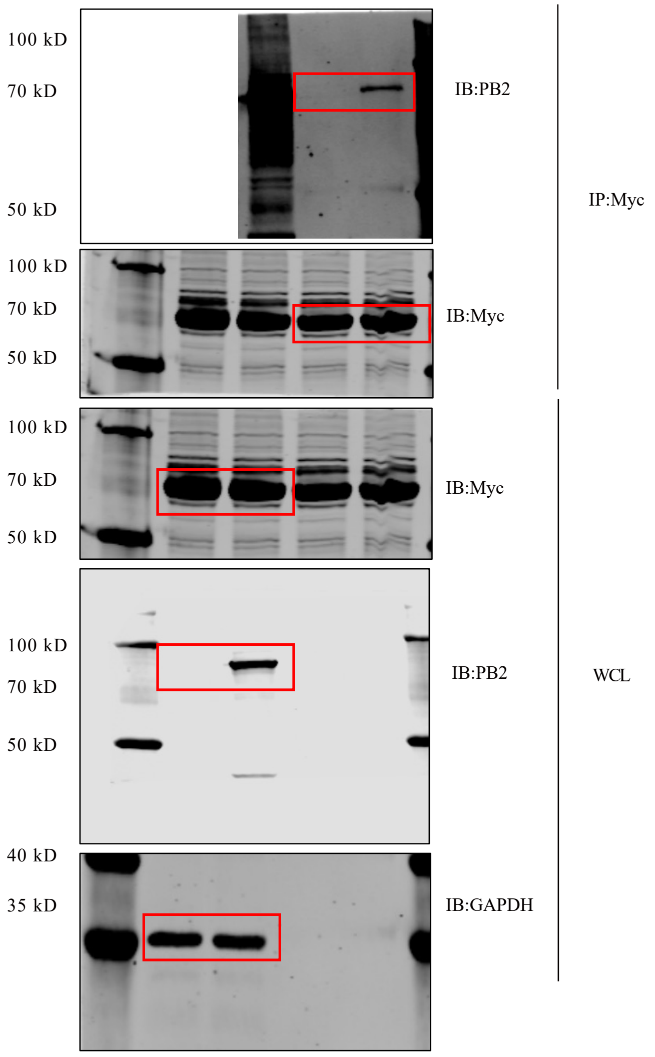

100 kD
70 kD
50 kD
IB:PB2
100 kD
70 kD
50 kD
IB:Myc
IP:Myc
100 kD
70 kD
50 kD
IB:Myc
100 kD
70 kD
50 kD
IB:PB2
WCL
40 kD
35 kD
IB:GAPDH

Fig 5D

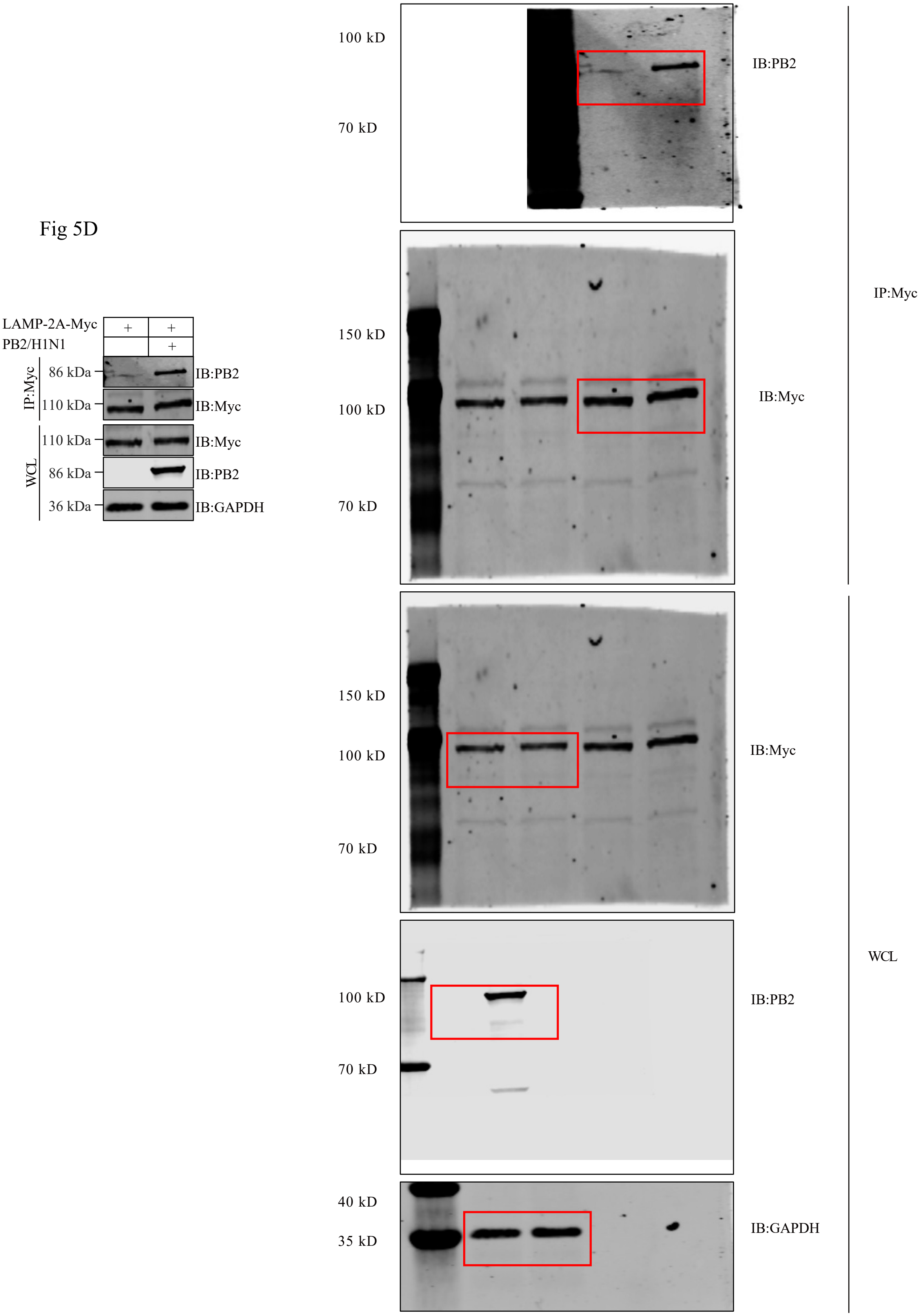

LAMP-2A-Myc
PB2/H1N1
IP:Myc
WCL
86 kDa
110 kDa
36 kDa
IB:PB2
IB:Myc
IB:GAPDH
100 kD
70 kD
150 kD
40 kD
35 kD

Fig 5E

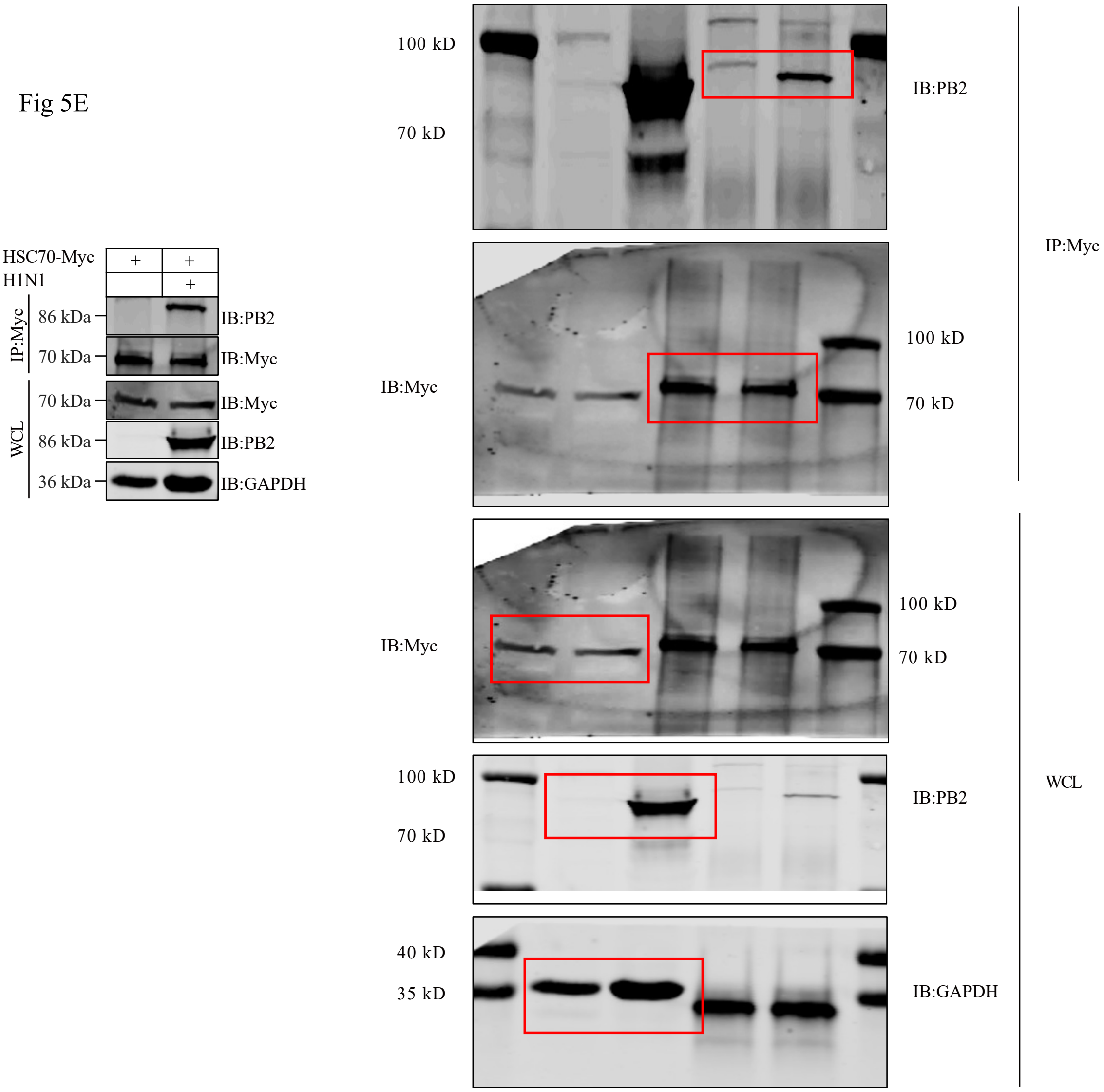
HSC70-Myc
H1N1
+
+
+
IP:Myc
WCL
86 kDa
70 kDa
70 kDa
86 kDa
36 kDa
IB:PB2
IB:Myc
IB:Myc
IB:PB2
IB:GAPDH
100 kD
70 kD
IB:PB2
IP:Myc
IB:Myc
100 kD
70 kD
IB:Myc
100 kD
70 kD
100 kD
70 kD
IB:PB2
WCL
40 kD
35 kD
IB:GAPDH

Fig 5F

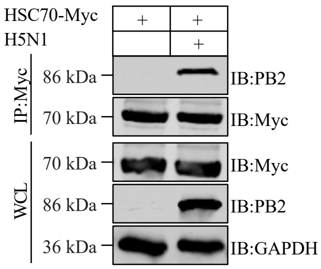

HSC70-Myc
H5N1
+
+
+
IP:Myc
WCL
86 kDa
70 kDa
70 kDa
86 kDa
36 kDa
IB:PB2
IB:Myc
IB:Myc
IB:PB2
IB:GAPDH

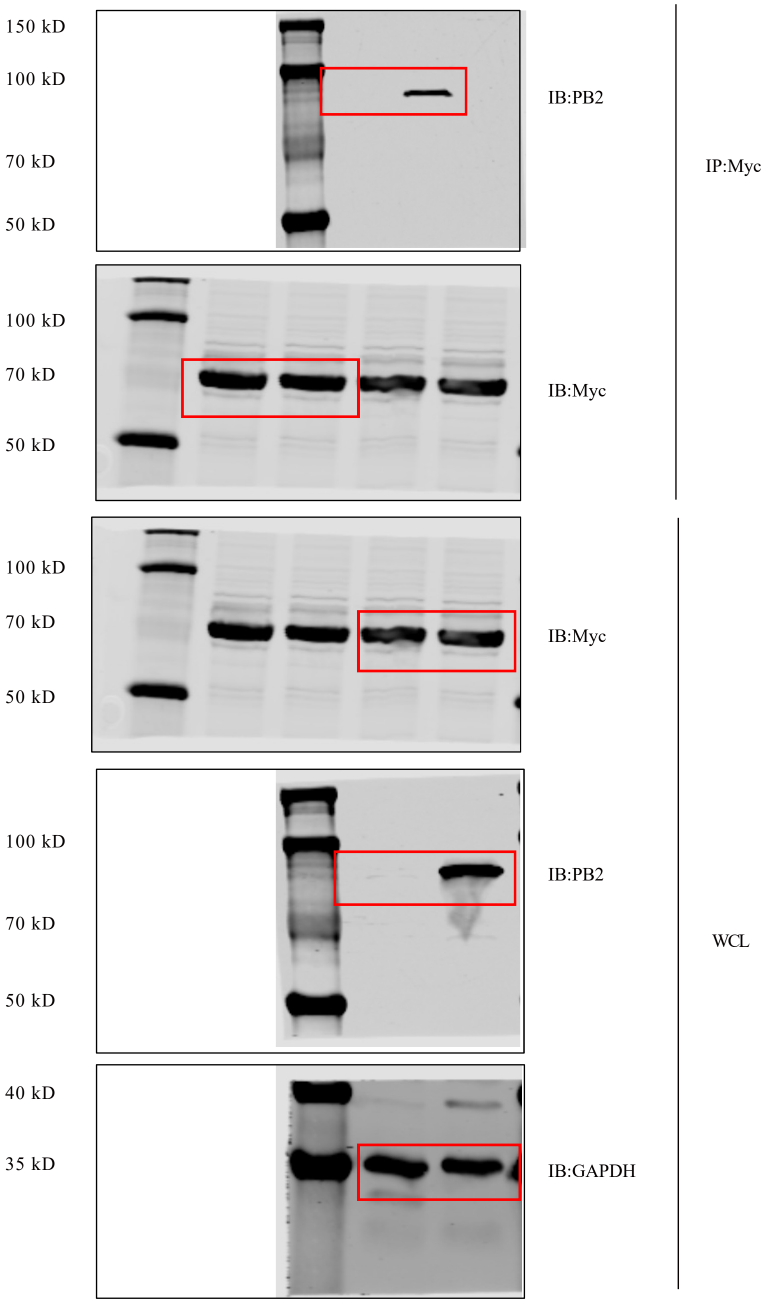

150 kD
100 kD
70 kD
50 kD
IB:PB2
100 kD
70 kD
50 kD
IB:Myc
IP:Myc
100 kD
70 kD
50 kD
IB:Myc
100 kD
70 kD
50 kD
IB:PB2
WCL
40 kD
35 kD
IB:GAPDH

Fig 5G

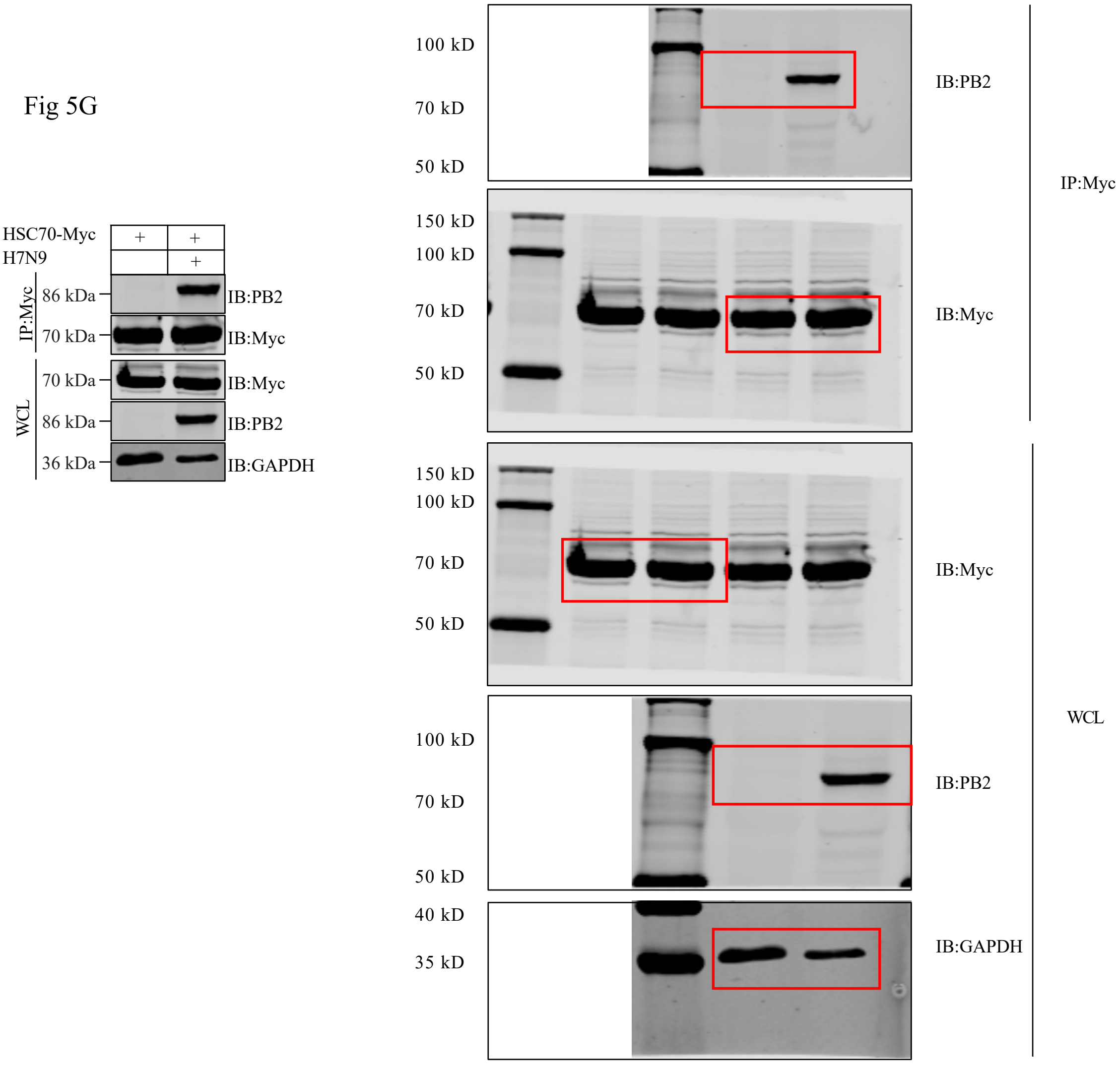

HSC70-Myc
H7N9
+
+
+
IP:Myc
86 kDa
70 kDa
WCL
70 kDa
86 kDa
36 kDa
IB:PB2
IB:Myc
IB:Myc
IB:PB2
IB:GAPDH
100 kD
70 kD
50 kD
150 kD
100 kD
70 kD
50 kD
150 kD
100 kD
70 kD
50 kD
100 kD
70 kD
50 kD
40 kD
35 kD
IB:PB2
IB:Myc
IP:Myc
IB:Myc
WCL
IB:PB2
IB:GAPDH

Fig 5H

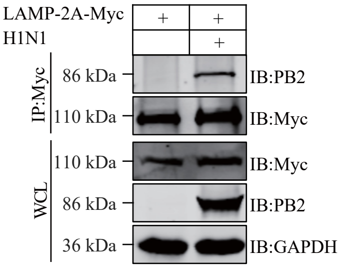

LAMP-2A-Myc
H1N1
IP:Myc
WCL
86 kDa
110 kDa
110 kDa
86 kDa
36 kDa
IB:PB2
IB:Myc
IB:Myc
IB:PB2
IB:GAPDH

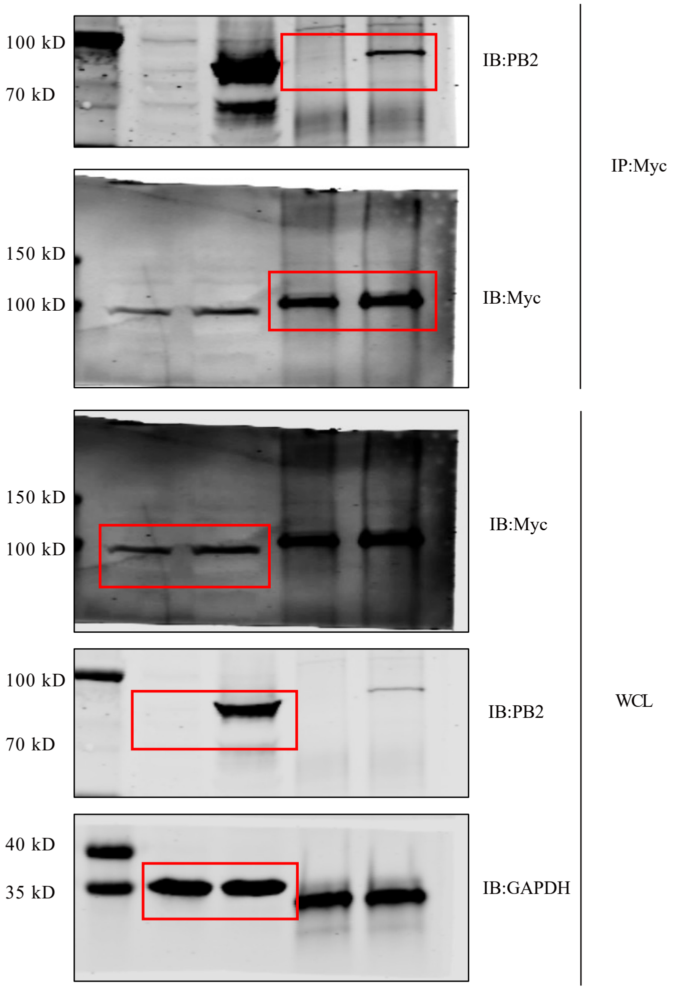

100 kD
70 kD
IB:PB2
150 kD
100 kD
IB:Myc
IP:Myc
150 kD
100 kD
IB:Myc
100 kD
70 kD
IB:PB2
WCL
40 kD
35 kD
IB:GAPDH

Fig 5I

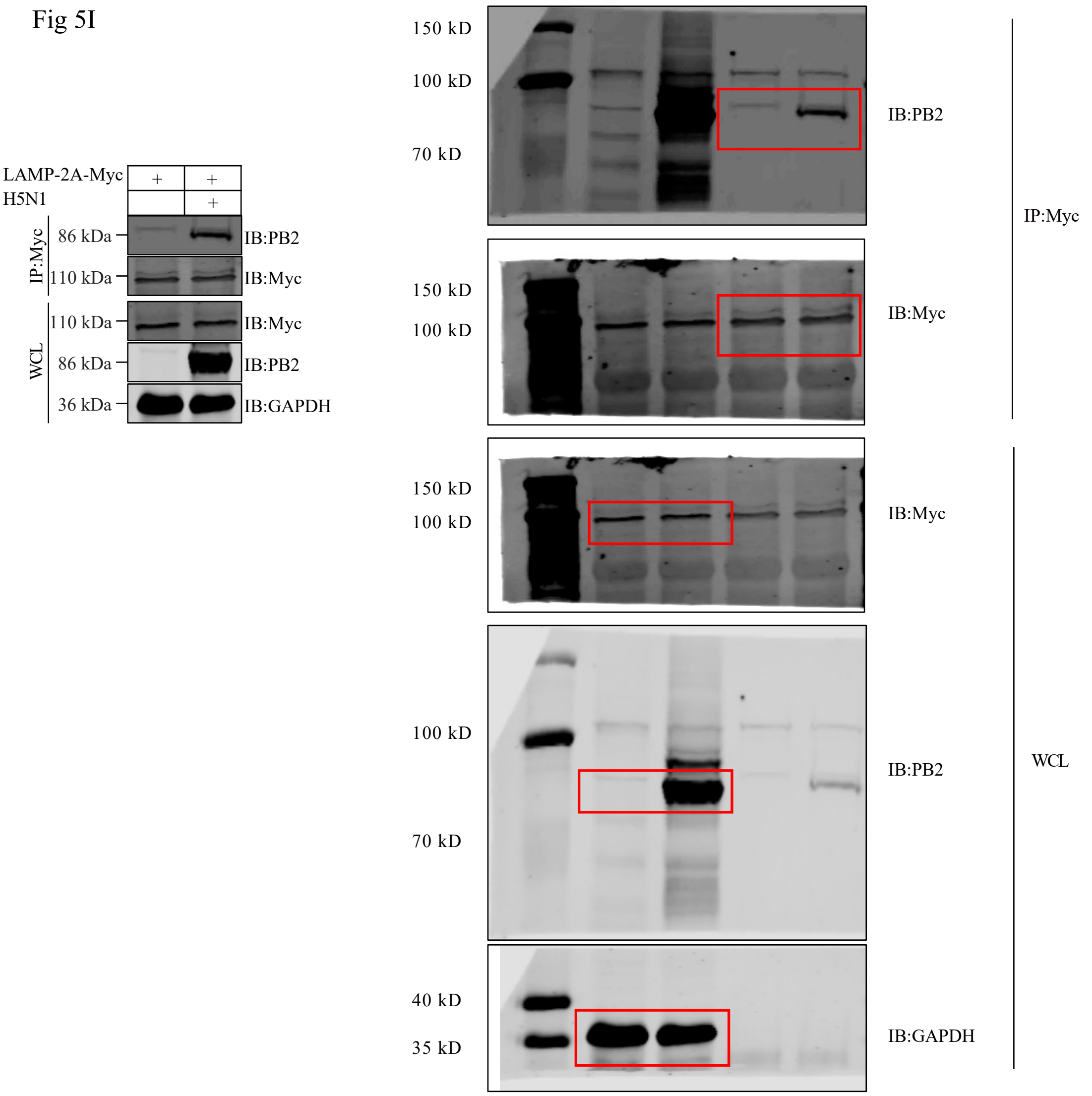

LAMP-2A-Myc
H5N1
+
+
+
IP:Myc
WCL
86 kDa
110 kDa
110 kDa
86 kDa
36 kDa
IB:PB2
IB:Myc
IB:Myc
IB:PB2
IB:GAPDH
150 kD
100 kD
70 kD
IB:PB2
IP:Myc
150 kD
100 kD
IB:Myc
150 kD
100 kD
IB:Myc
100 kD
70 kD
IB:PB2
WCL
40 kD
35 kD
IB:GAPDH

Fig 5J

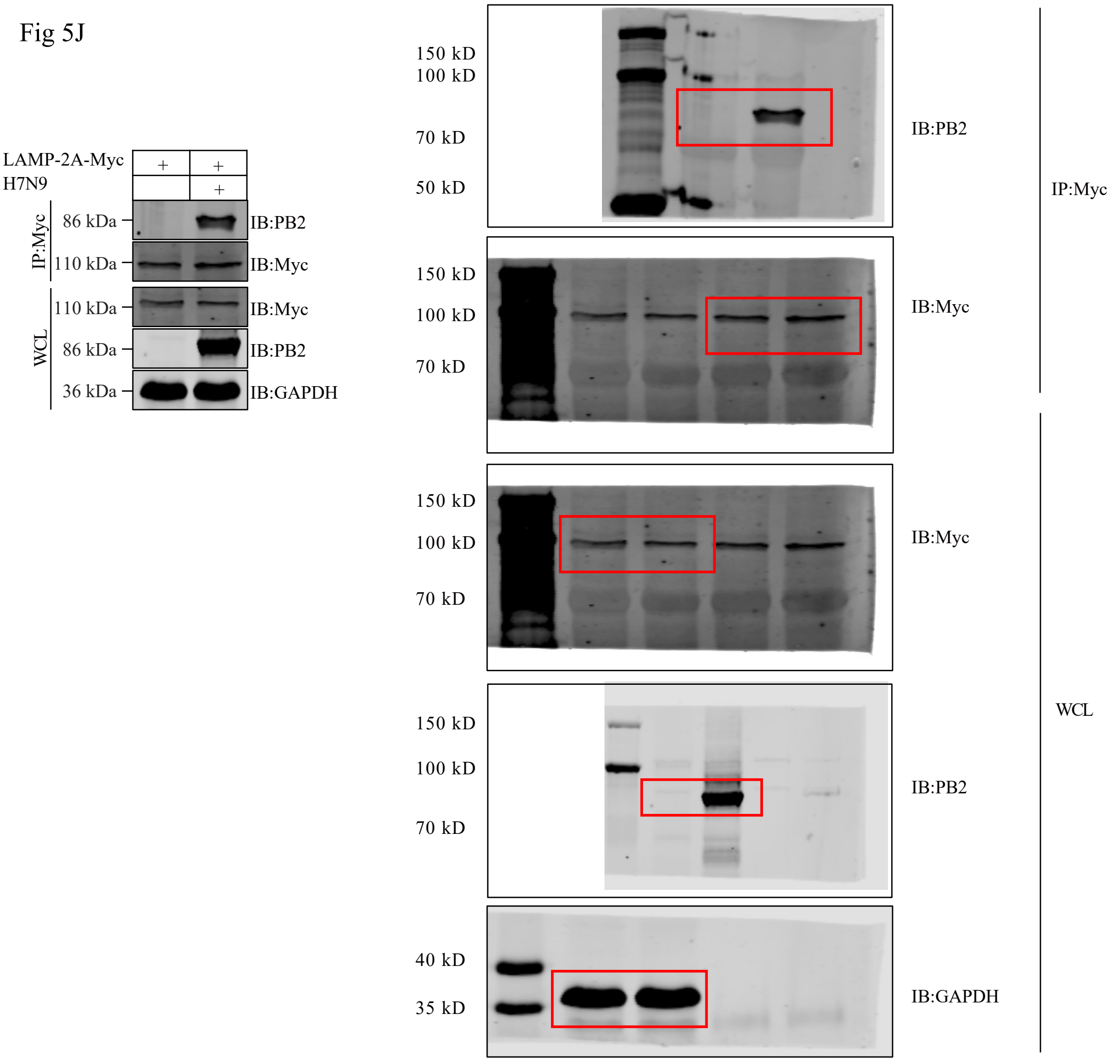
LAMP-2A-Myc
H7N9
+
+
+
IP:Myc
86 kDa
IB:PB2
110 kDa
IB:Myc
WCL
110 kDa
IB:Myc
86 kDa
IB:PB2
36 kDa
IB:GAPDH
150 kD
100 kD
70 kD
50 kD
IB:PB2
IP:Myc
150 kD
100 kD
70 kD
IB:Myc
150 kD
100 kD
70 kD
IB:Myc
WCL
150 kD
100 kD
70 kD
IB:PB2
40 kD
35 kD
IB:GAPDH

Fig 6B

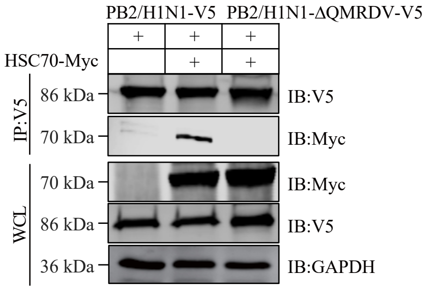

PB2/H1N1-V5
PB2/H1N1-ΔQMRDV-V5
HSC70-Myc
IP:V5
WCL
86 kDa
70 kDa
70 kDa
86 kDa
36 kDa
IB:V5
IB:Myc
IB:Myc
IB:V5
IB:GAPDH

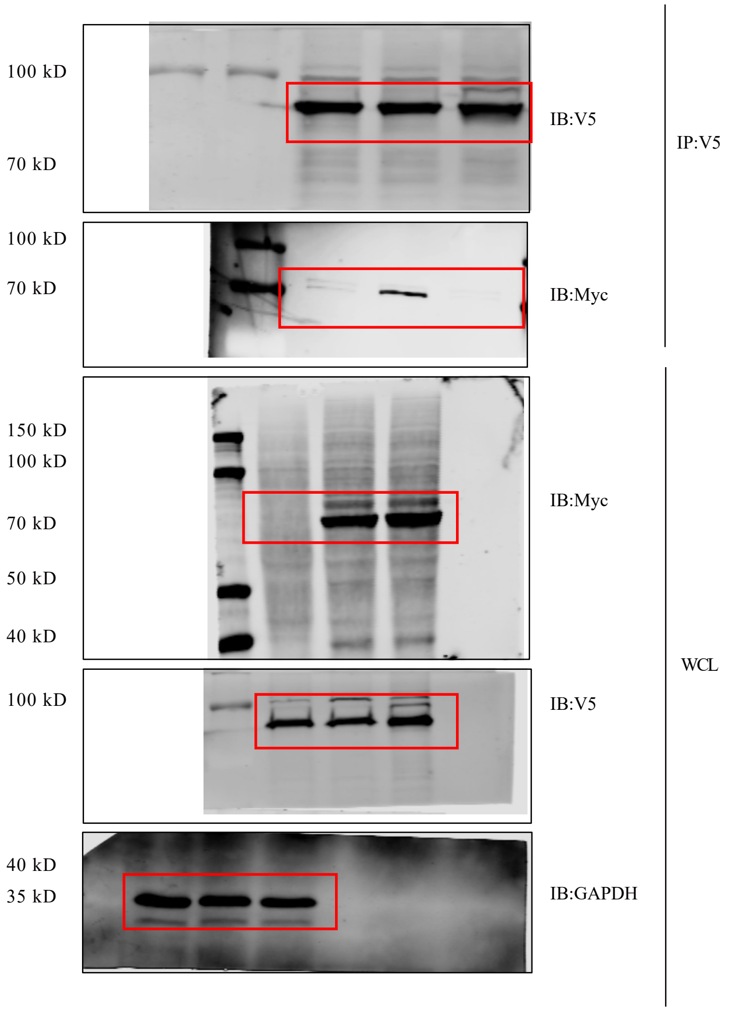

100 kD
70 kD
100 kD
70 kD
150 kD
100 kD
70 kD
50 kD
40 kD
100 kD
40 kD
35 kD
IB:V5
IB:Myc
IB:Myc
IB:V5
IB:GAPDH
IP:V5
WCL

Fig 6C

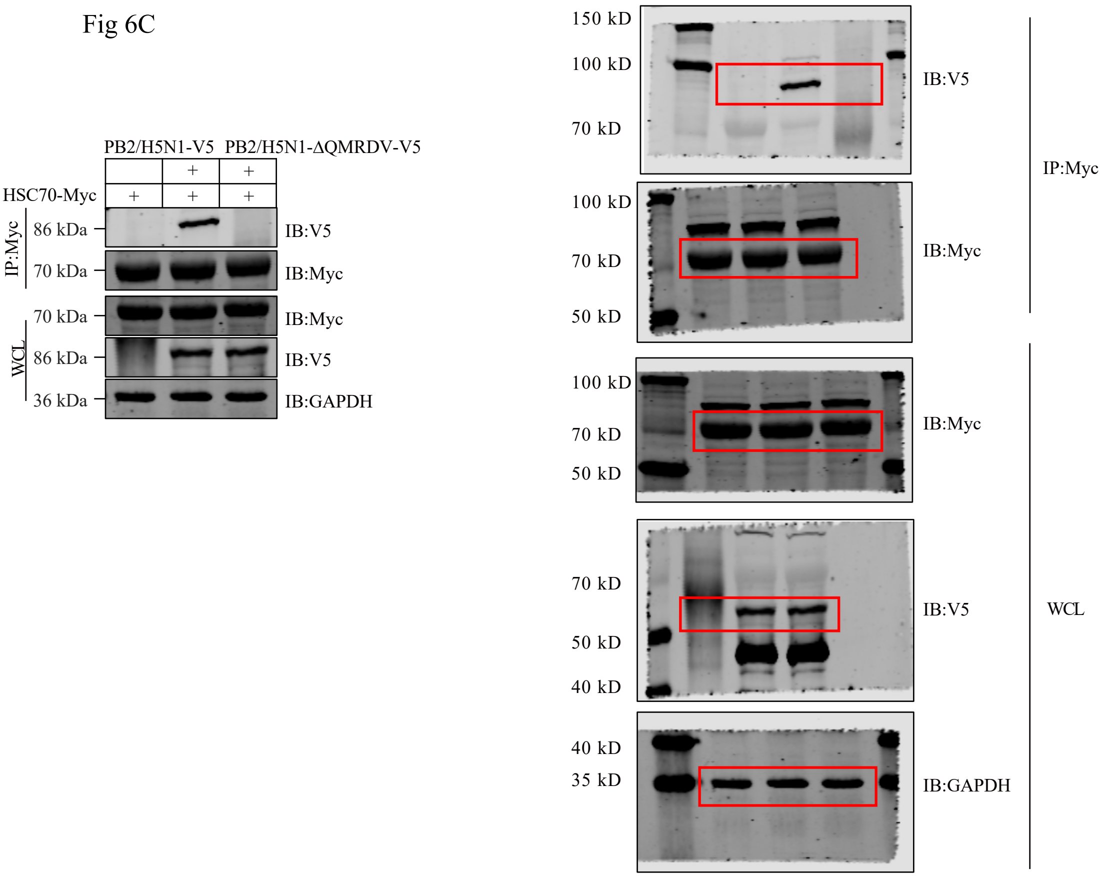
PB2/H5N1-V5
PB2/H5N1-ΔQMRDV-V5
HSC70-Myc
IP:Myc
WCL
86 kDa
70 kDa
36 kDa
IB:V5
IB:Myc
IB:GAPDH
150 kD
100 kD
70 kD
50 kD
40 kD
35 kD

Fig 6D

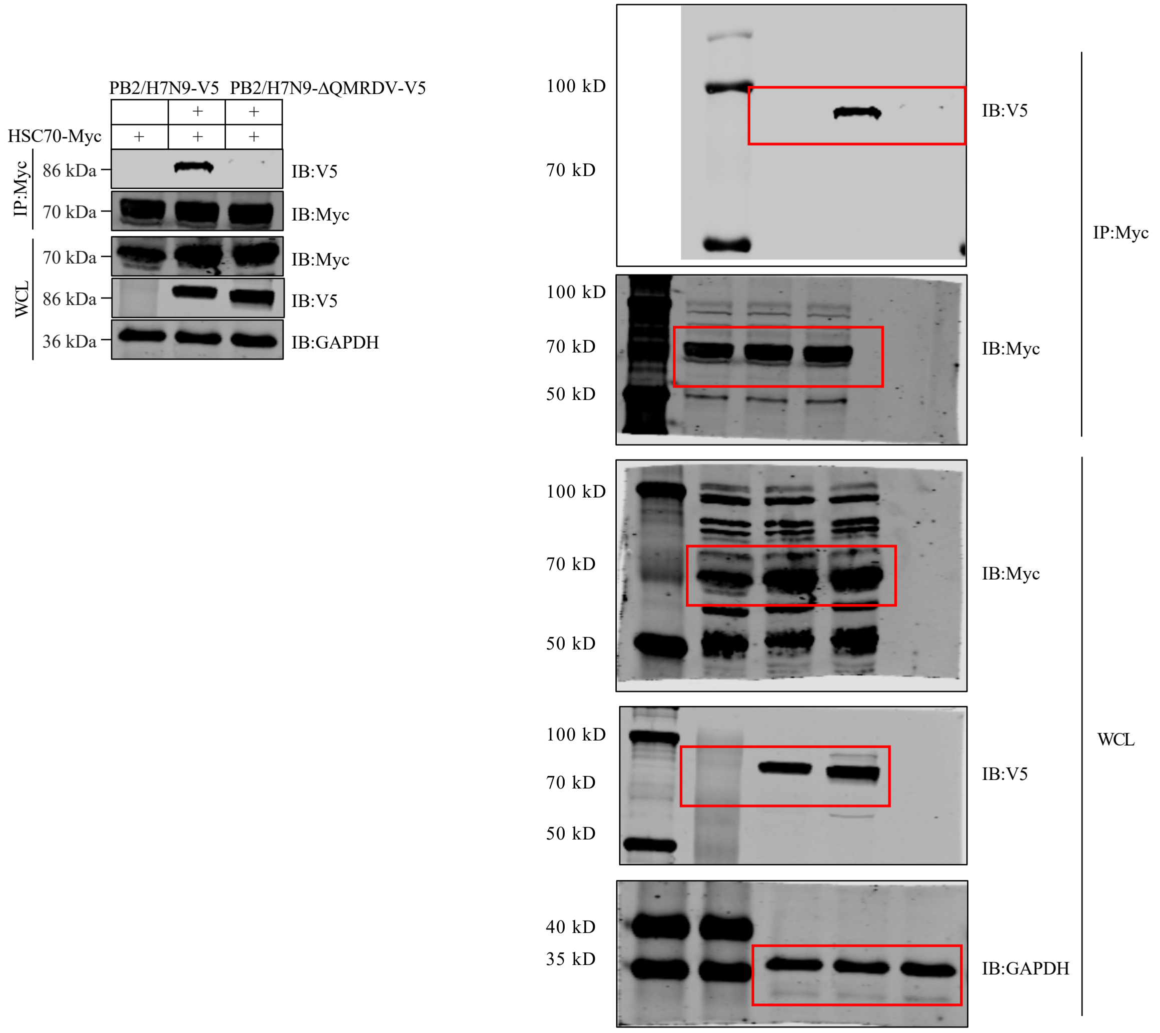

PB2/H7N9-V5
PB2/H7N9-ΔQMRDV-V5
HSC70-Myc
IP:Myc
WCL
86 kDa
70 kDa
70 kDa
86 kDa
36 kDa
IB:V5
IB:Myc
IB:Myc
IB:V5
IB:GAPDH
100 kD
70 kD
100 kD
70 kD
50 kD
100 kD
70 kD
50 kD
100 kD
70 kD
50 kD
40 kD
35 kD
IB:V5
IB:Myc
IB:Myc
IB:V5
IB:GAPDH
IP:Myc
WCL

Fig 6E

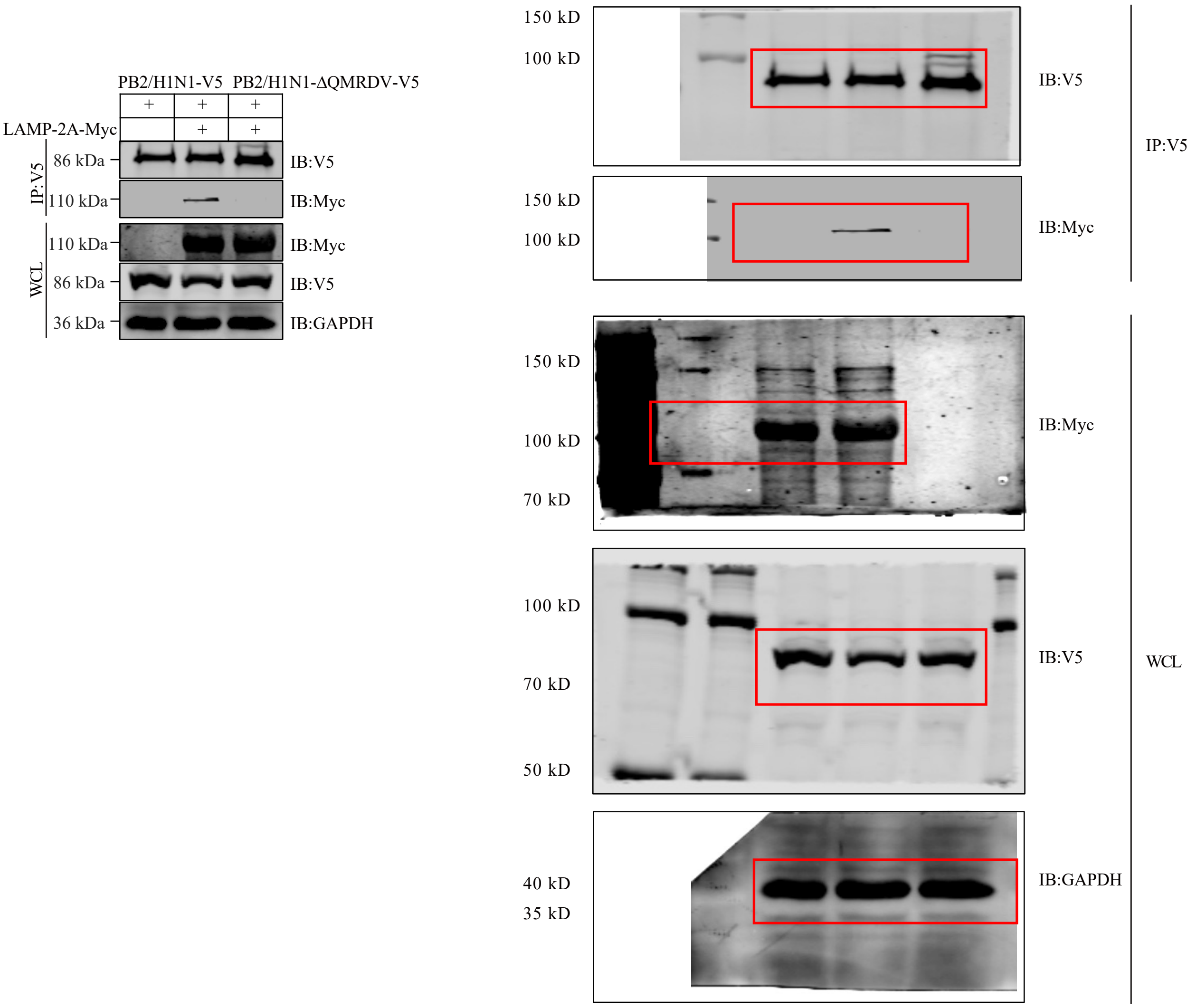
PB2/H1N1-V5
PB2/H1N1-ΔQMRDV-V5
LAMP-2A-Myc
IP:V5
WCL
86 kDa
110 kDa
36 kDa
IB:V5
IB:Myc
IB:GAPDH
150 kD
100 kD
70 kD
50 kD
40 kD
35 kD

Fig 6F

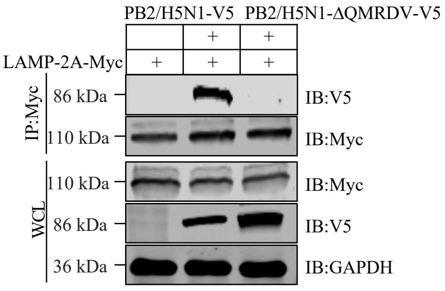
PB2/H5N1-V5
PB2/H5N1-ΔQMRDV-V5
LAMP-2A-Myc
+
+
+
+
+
IP:Myc
WCL
86 kDa
110 kDa
110 kDa
86 kDa
36 kDa
IB:V5
IB:Myc
IB:Myc
IB:V5
IB:GAPDH

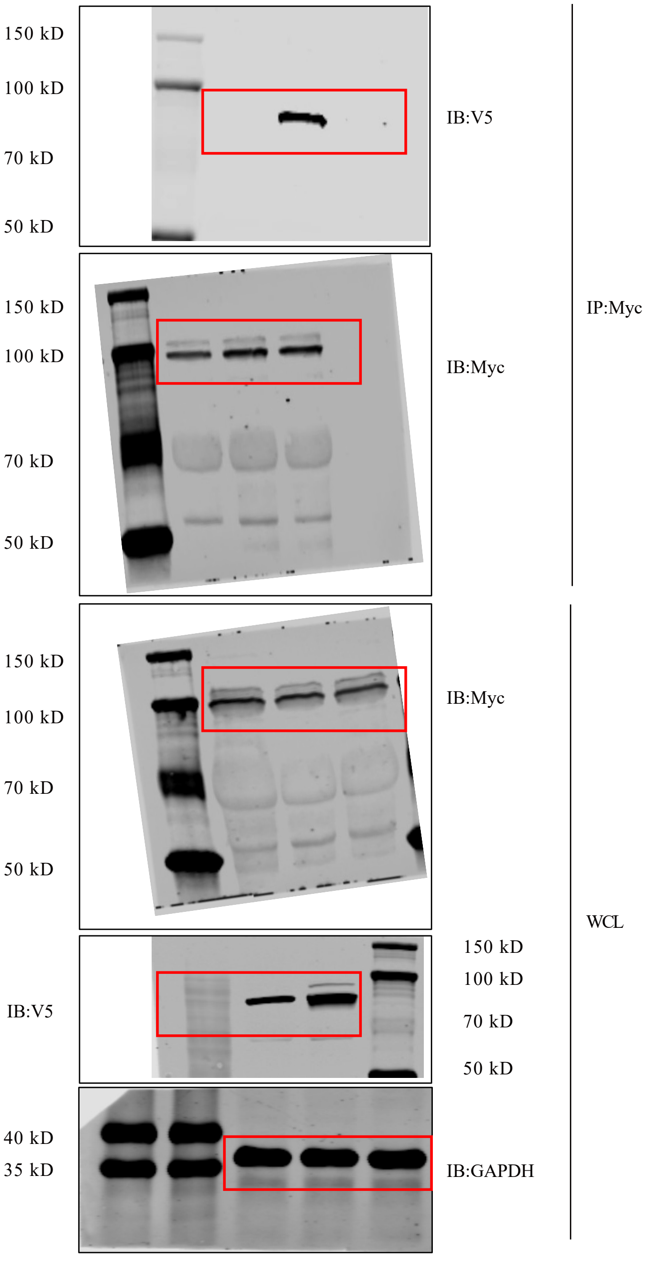
150 kD
100 kD
70 kD
50 kD
IB:V5
150 kD
100 kD
70 kD
50 kD
IB:Myc
IP:Myc
150 kD
100 kD
70 kD
50 kD
IB:Myc
IB:V5
150 kD
100 kD
70 kD
50 kD
40 kD
35 kD
IB:GAPDH
WCL

Fig 6G

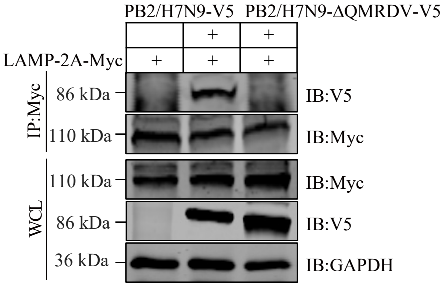
PB2/H7N9-V5
PB2/H7N9-ΔQMRDV-V5
LAMP-2A-Myc
IP:Myc
WCL
86 kDa
110 kDa
110 kDa
86 kDa
36 kDa
IB:V5
IB:Myc
IB:Myc
IB:V5
IB:GAPDH

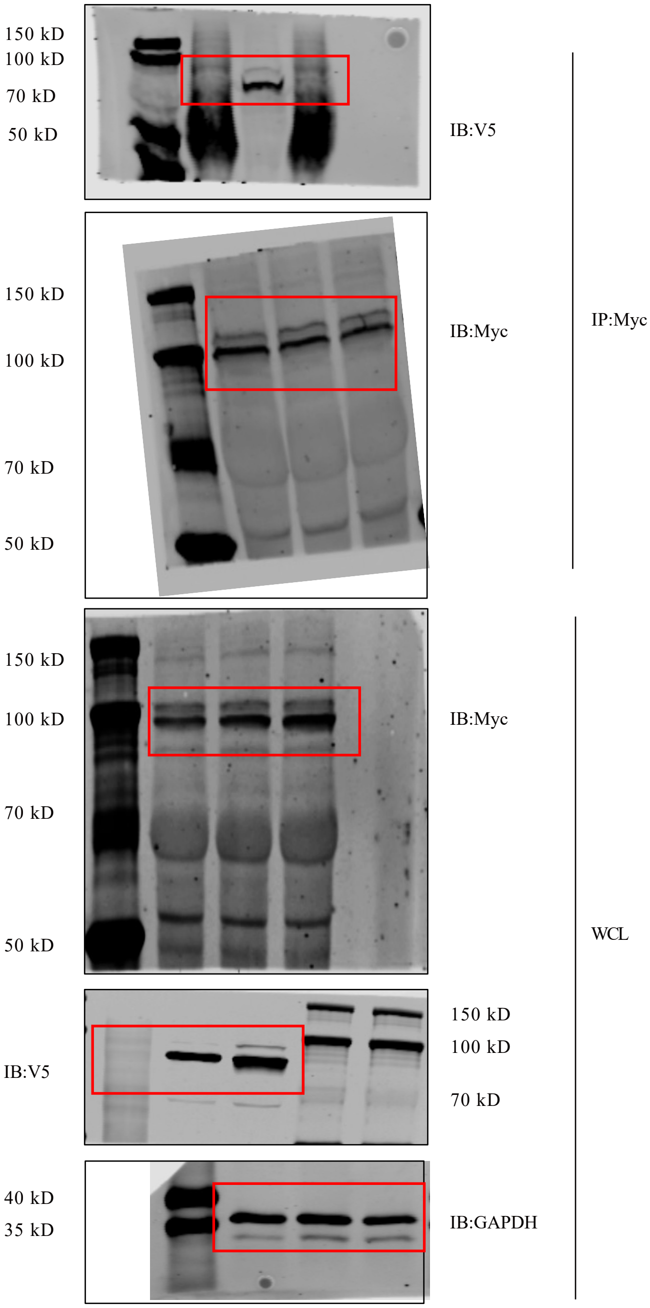
150 kD
100 kD
70 kD
50 kD
IB:V5
IB:Myc
IP:Myc
IB:Myc
WCL
IB:V5
IB:GAPDH
40 kD
35 kD

Fig 6H

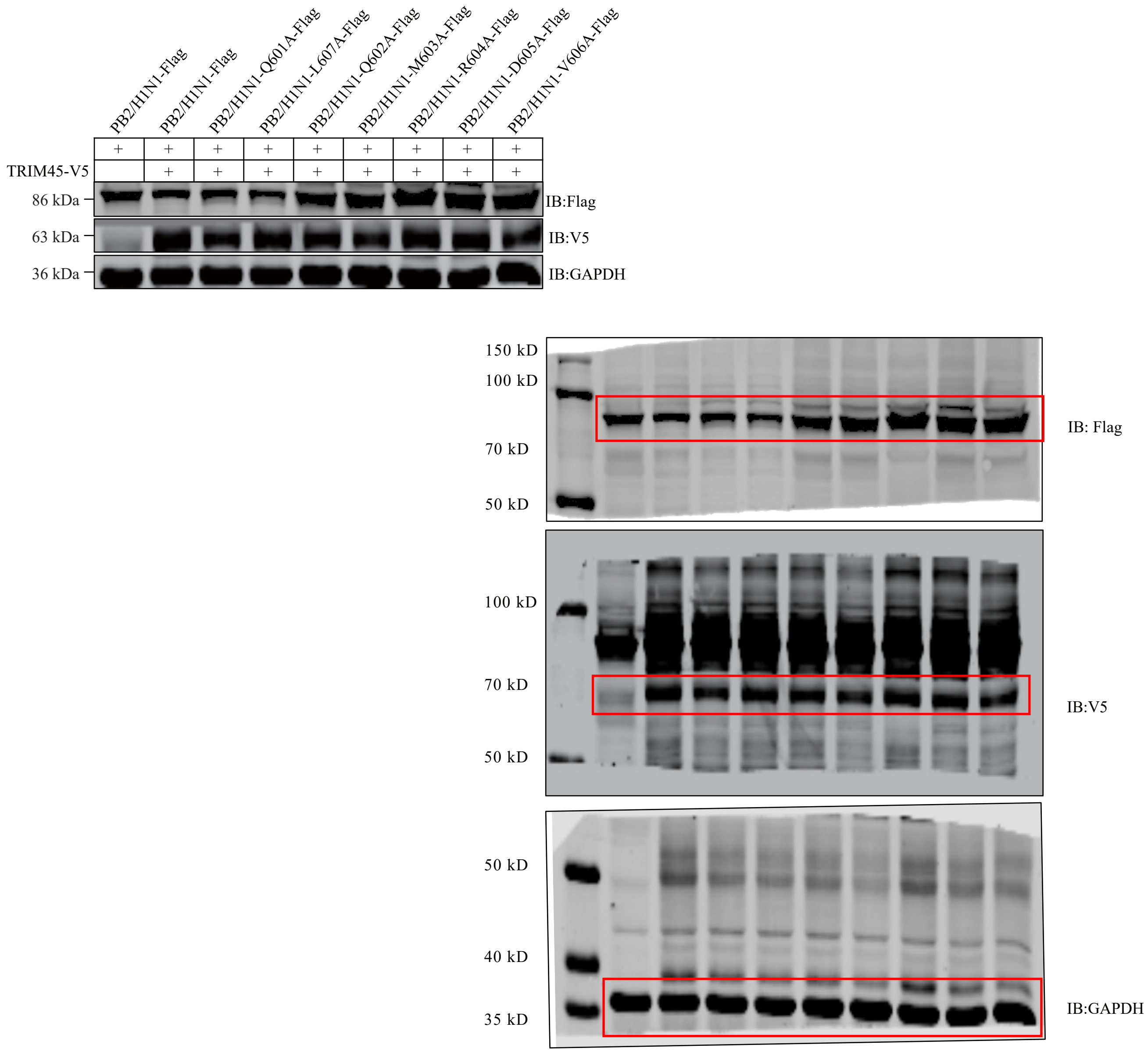

PB2/H1N1-Flag
PB2/H1N1-Flag
PB2/H1N1-Q601A-Flag
PB2/H1N1-L607A-Flag
PB2/H1N1-Q602A-Flag
PB2/H1N1-M603A-Flag
PB2/H1N1-R604A-Flag
PB2/H1N1-D605A-Flag
PB2/H1N1-V606A-Flag
TRIM45-V5
86 kDa
63 kDa
36 kDa
IB:Flag
IB:V5
IB:GAPDH
150 kD
100 kD
70 kD
50 kD
IB: Flag
100 kD
70 kD
50 kD
IB:V5
50 kD
40 kD
35 kD
IB:GAPDH

Fig 6I

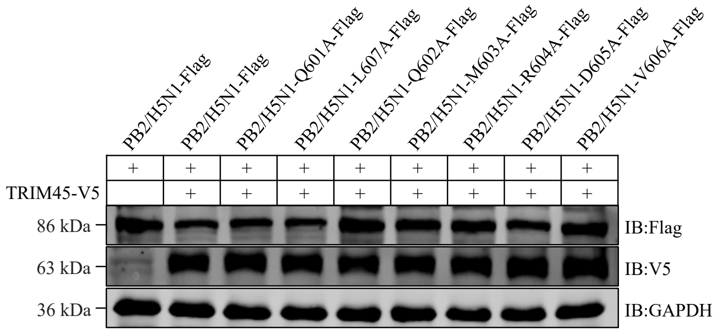
PB2/H5N1-Flag
PB2/H5N1-Flag
PB2/H5N1-Q601A-Flag
PB2/H5N1-L607A-Flag
PB2/H5N1-Q602A-Flag
PB2/H5N1-M603A-Flag
PB2/H5N1-R604A-Flag
PB2/H5N1-D605A-Flag
PB2/H5N1-V606A-Flag
TRIM45-V5
86 kDa
63 kDa
36 kDa
IB:Flag
IB:V5
IB:GAPDH

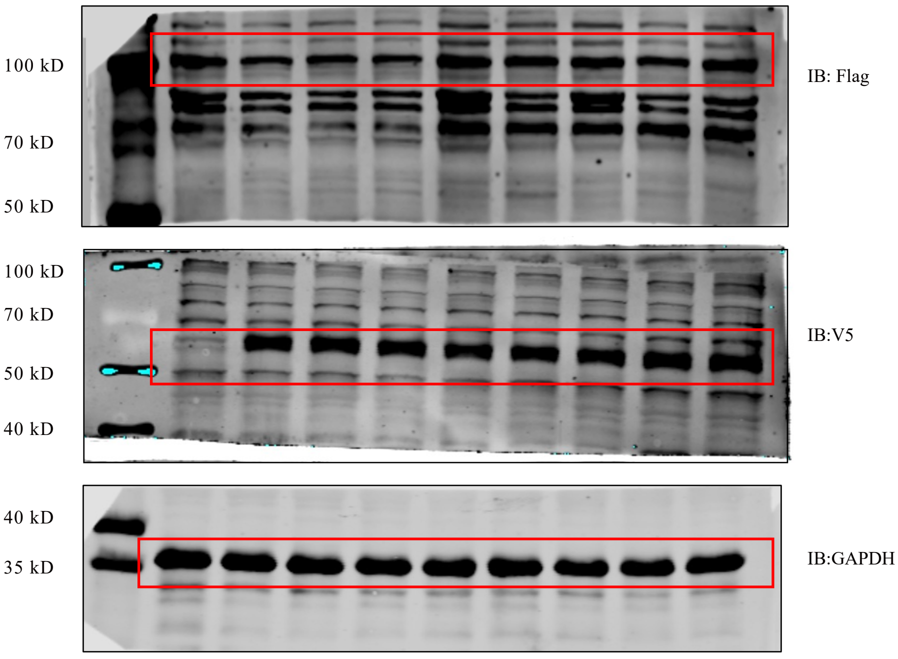
100 kD
70 kD
50 kD
IB: Flag
100 kD
70 kD
50 kD
40 kD
IB:V5
40 kD
35 kD
IB:GAPDH

Fig 6J

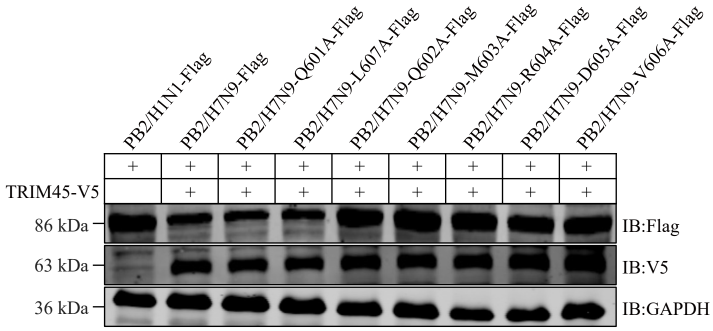
PB2/H1N1-Flag
PB2/H7N9-Flag
PB2/H7N9-Q601A-Flag
PB2/H7N9-L607A-Flag
PB2/H7N9-Q602A-Flag
PB2/H7N9-M603A-Flag
PB2/H7N9-R604A-Flag
PB2/H7N9-D605A-Flag
PB2/H7N9-V606A-Flag
TRIM45-V5
86 kDa
63 kDa
36 kDa
IB:Flag
IB:V5
IB:GAPDH

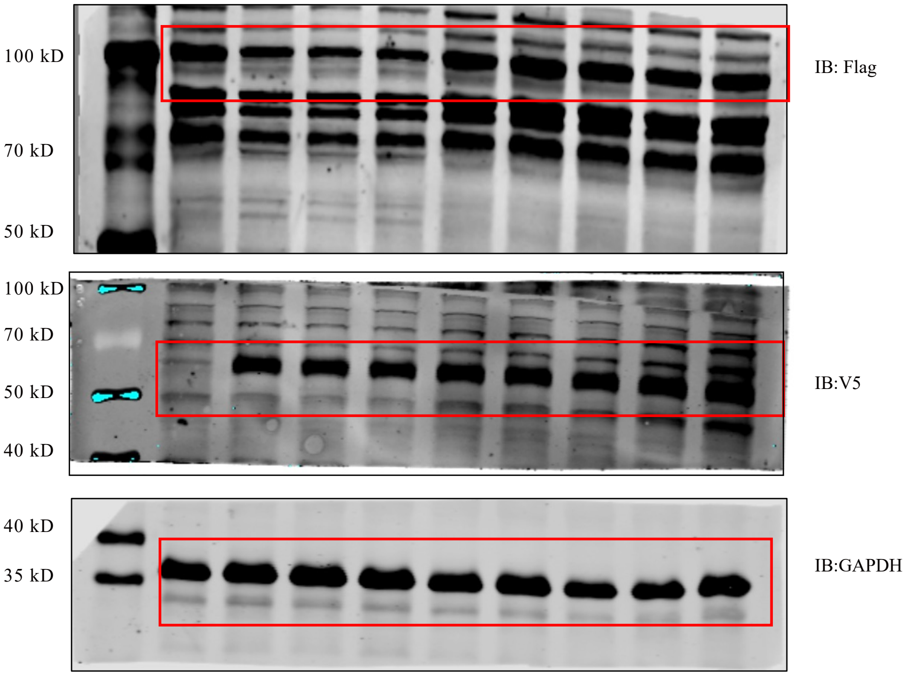
100 kD
70 kD
50 kD
IB: Flag
100 kD
70 kD
50 kD
40 kD
IB:V5
40 kD
35 kD
IB:GAPDH

Fig 7A

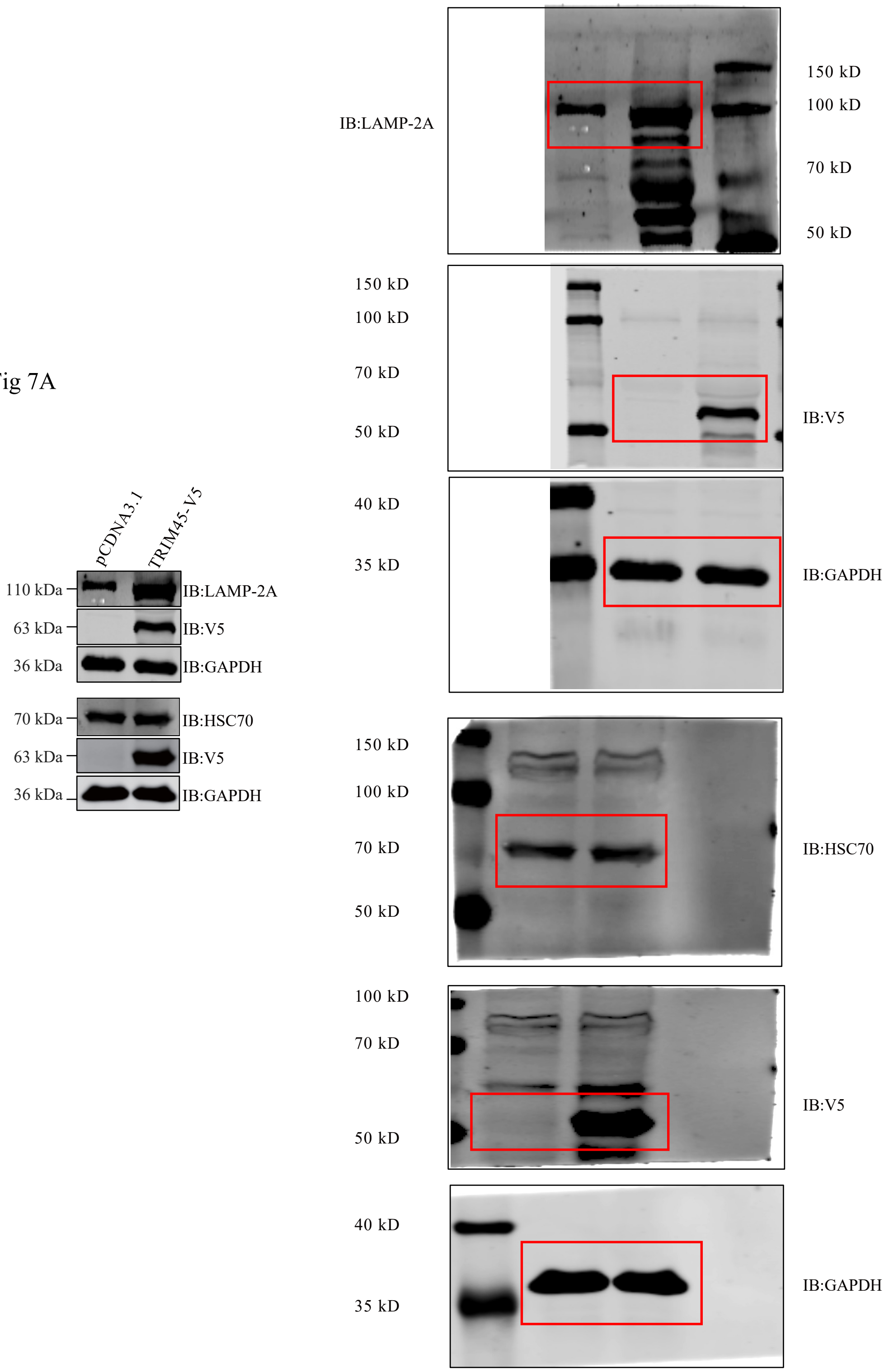

pCDNA3.1
TRIM45-V5
110 kDa
IB:LAMP-2A
63 kDa
IB:V5
36 kDa
IB:GAPDH
70 kDa
IB:HSC70
63 kDa
IB:V5
36 kDa
IB:GAPDH
IB:LAMP-2A
150 kD
100 kD
70 kD
50 kD
150 kD
100 kD
70 kD
50 kD
IB:V5
40 kD
35 kD
IB:GAPDH
150 kD
100 kD
70 kD
50 kD
IB:HSC70
100 kD
70 kD
50 kD
IB:V5
40 kD
35 kD
IB:GAPDH

Fig 7B

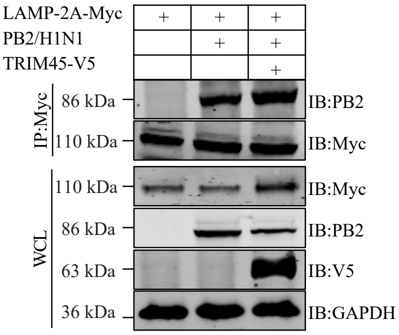
LAMP-2A-Myc
PB2/H1N1
TRIM45-V5
+ + +
+ +
+
IP:Myc
WCL
86 kDa
110 kDa
110 kDa
86 kDa
63 kDa
36 kDa
IB:PB2
IB:Myc
IB:Myc
IB:PB2
IB:V5
IB:GAPDH

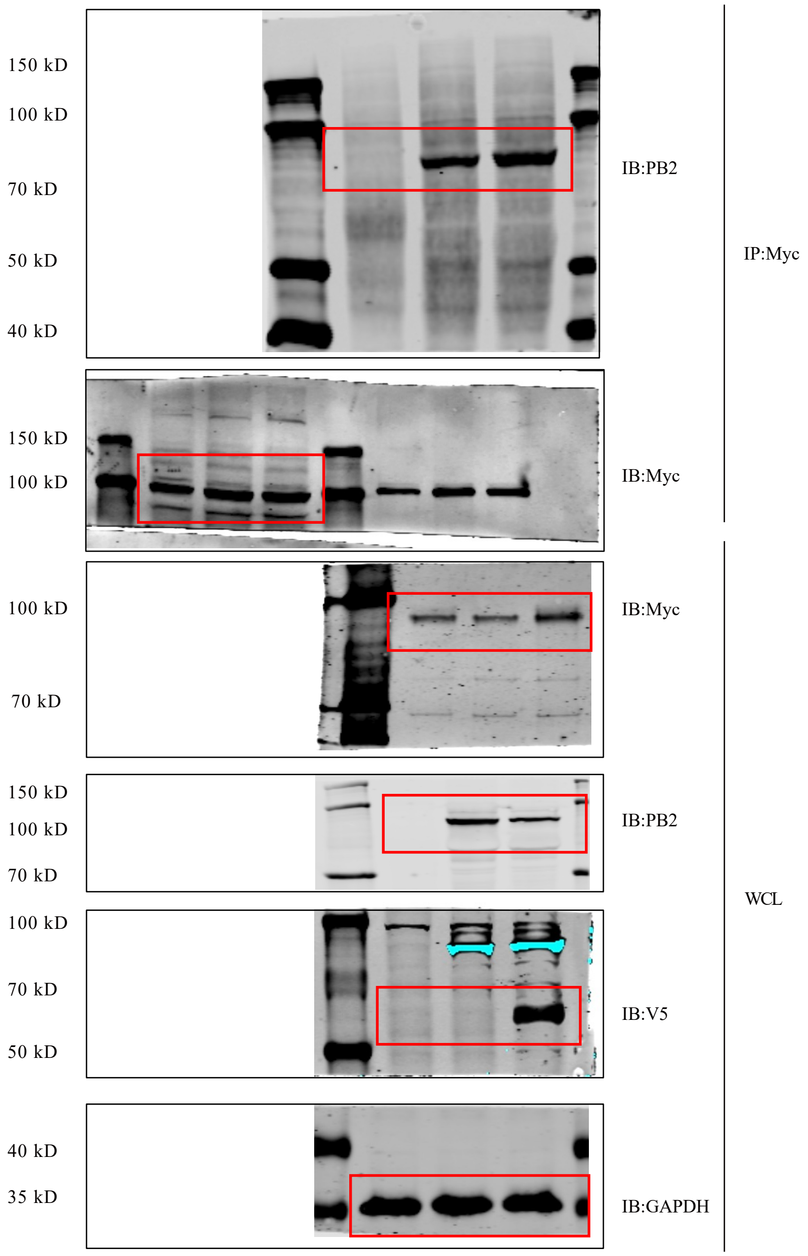
150 kD
100 kD
70 kD
50 kD
40 kD
IB:PB2
IP:Myc
150 kD
100 kD
IB:Myc
100 kD
70 kD
IB:Myc
150 kD
100 kD
70 kD
IB:PB2
WCL
100 kD
70 kD
50 kD
IB:V5
40 kD
35 kD
IB:GAPDH

Fig 7C

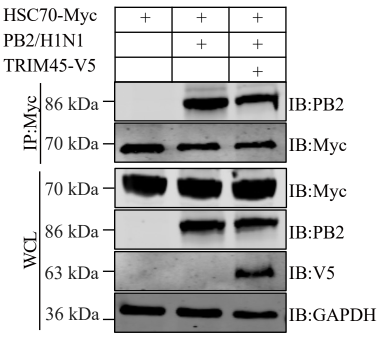

HSC70-Myc + + +
PB2/H1N1 + +
TRIM45-V5 +
IP:Myc
WCL
86 kDa
70 kDa
70 kDa
86 kDa
63 kDa
36 kDa
IB:PB2
IB:Myc
IB:Myc
IB:PB2
IB:V5
IB:GAPDH

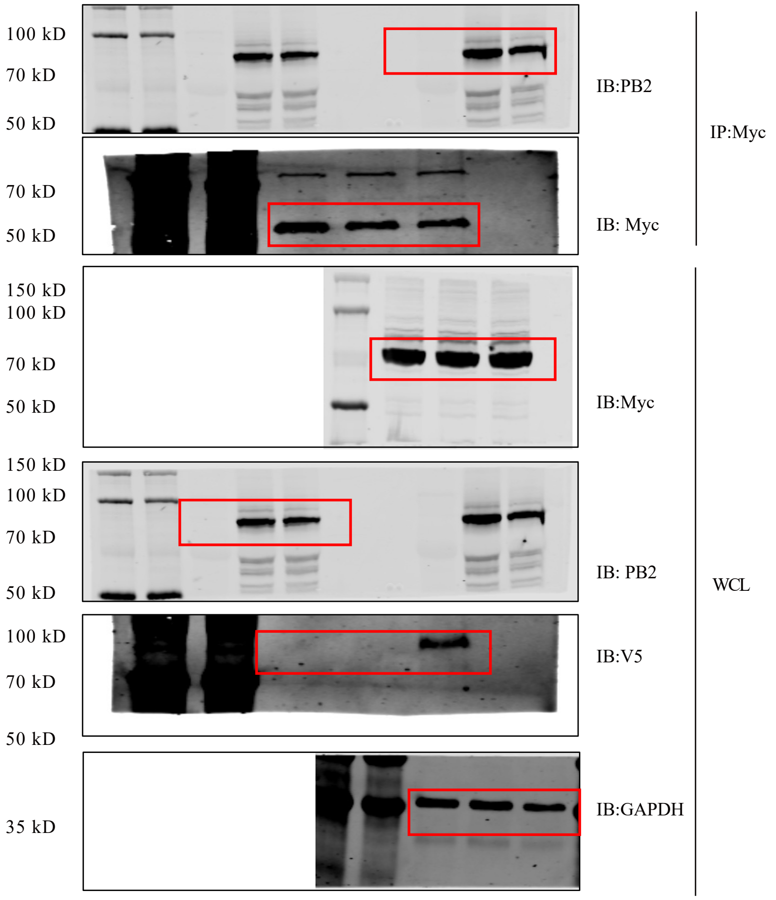

100 kD
70 kD
50 kD
70 kD
50 kD
150 kD
100 kD
70 kD
50 kD
150 kD
100 kD
70 kD
50 kD
100 kD
70 kD
50 kD
35 kD
IB:PB2
IB: Myc
IP:Myc
IB:Myc
IB: PB2
IB:V5
IB:GAPDH
WCL

Fig 7E

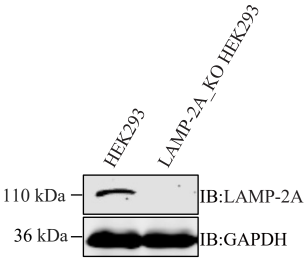
HEK293
LAMP-2A_KO HEK293
110 kDa
36 kDa
IB:LAMP-2A
IB:GAPDH

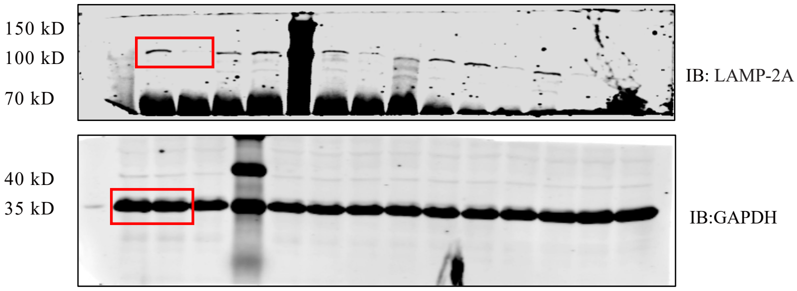
150 kD
100 kD
70 kD
IB: LAMP-2A
40 kD
35 kD
IB:GAPDH

Fig 7F

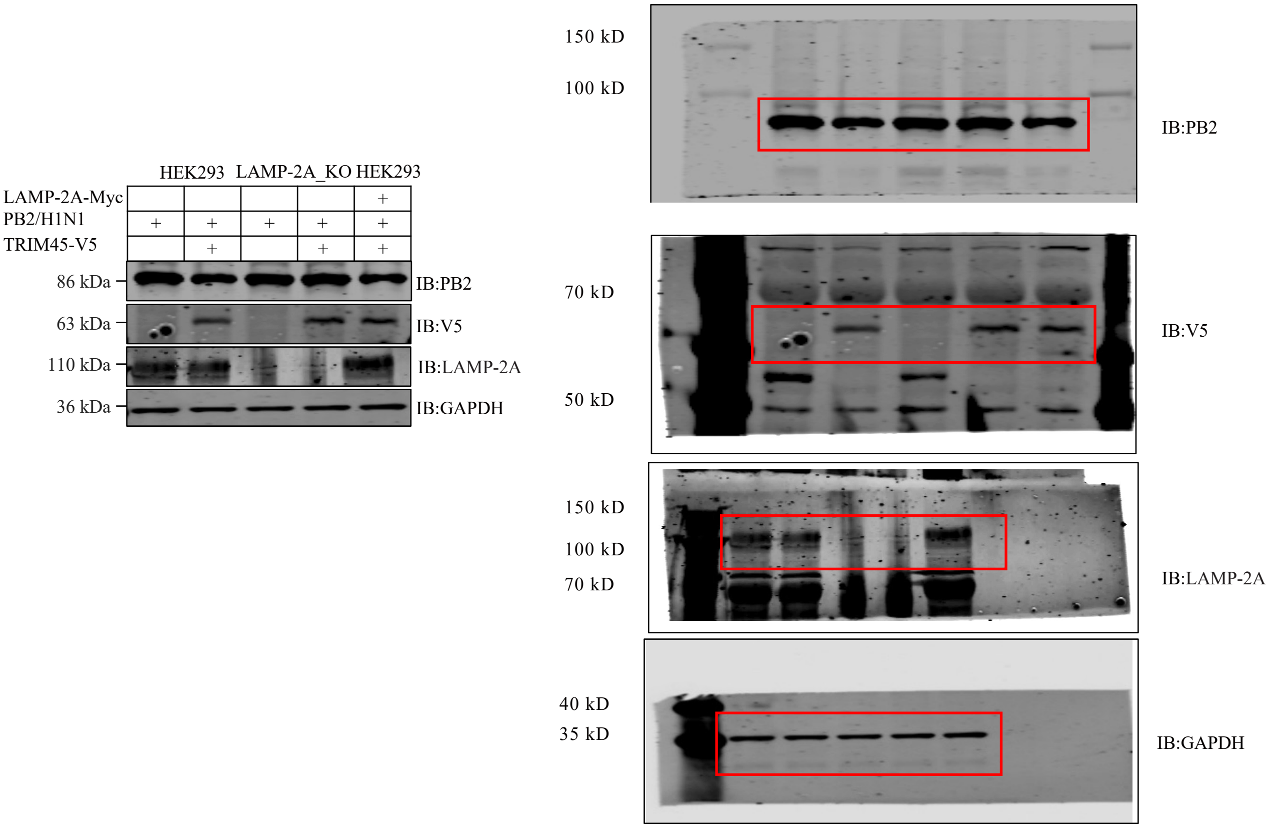

HEK293 LAMP-2A_KO HEK293
LAMP-2A-Myc
PB2/H1N1
TRIM45-V5
86 kDa
63 kDa
110 kDa
36 kDa
IB:PB2
IB:V5
IB:LAMP-2A
IB:GAPDH
150 kD
100 kD
70 kD
50 kD
150 kD
100 kD
70 kD
40 kD
35 kD

Fig 8A

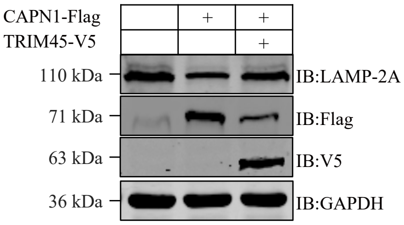
CAPN1-Flag
TRIM45-V5
+
+
+
110 kDa
71 kDa
63 kDa
36 kDa
IB:LAMP-2A
IB:Flag
IB:V5
IB:GAPDH

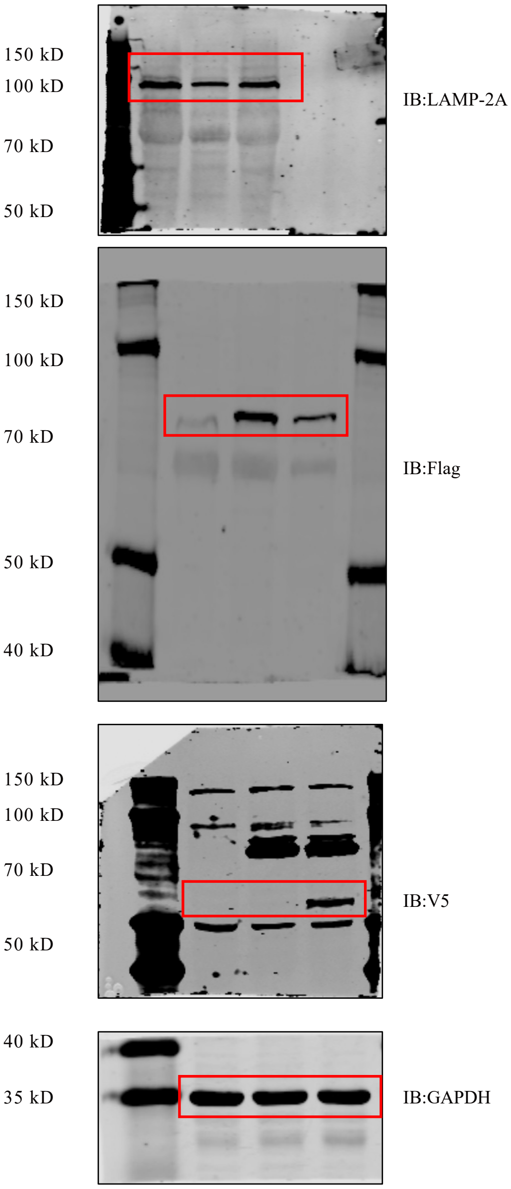
150 kD
100 kD
70 kD
50 kD
IB:LAMP-2A
150 kD
100 kD
70 kD
50 kD
40 kD
IB:Flag
150 kD
100 kD
70 kD
50 kD
IB:V5
40 kD
35 kD
IB:GAPDH

Fig 8B

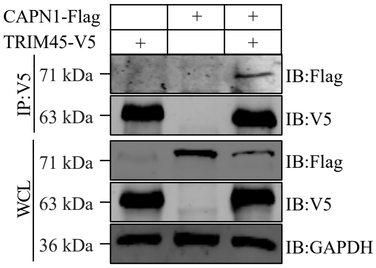

CAPN1-Flag
TRIM45-V5
+
+
+
+
IP:V5
71 kDa
63 kDa
IB:Flag
IB:V5
WCL
71 kDa
63 kDa
36 kDa
IB:Flag
IB:V5
IB:GAPDH

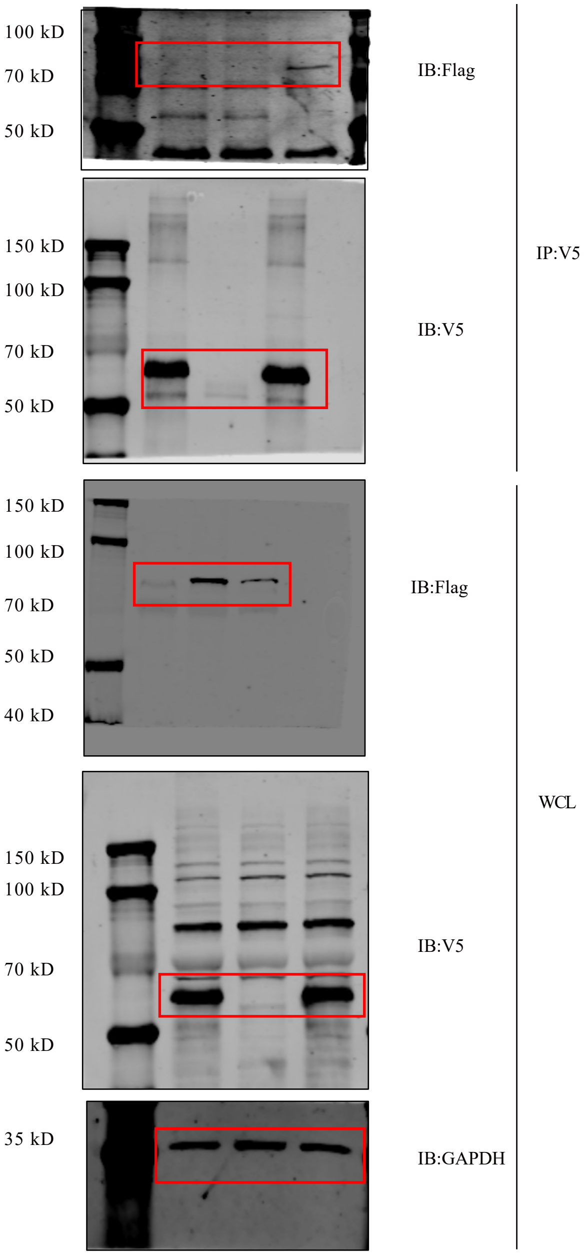

100 kD
70 kD
50 kD
IB:Flag
150 kD
100 kD
70 kD
50 kD
IB:V5
IP:V5
150 kD
100 kD
70 kD
50 kD
40 kD
IB:Flag
150 kD
100 kD
70 kD
50 kD
IB:V5
WCL
35 kD
IB:GAPDH

Fig 8C

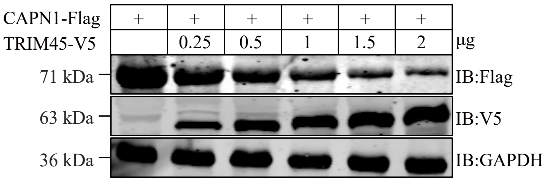
CAPN1-Flag
TRIM45-V5
+ + + + + +
0.25 0.5 1 1.5 2
μg
71 kDa
63 kDa
36 kDa
IB:Flag
IB:V5
IB:GAPDH

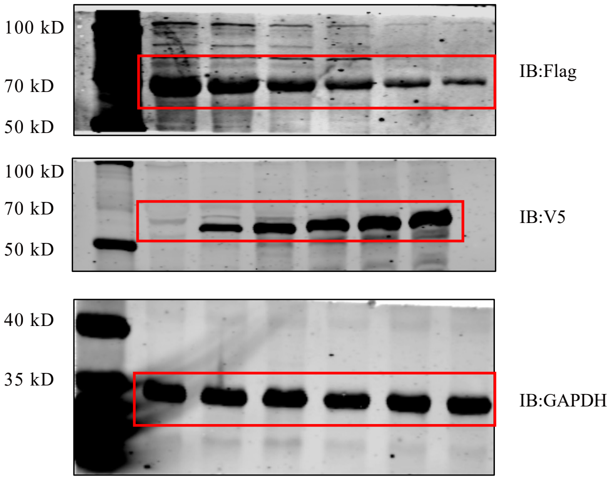
100 kD
70 kD
50 kD
IB:Flag
100 kD
70 kD
50 kD
IB:V5
40 kD
35 kD
IB:GAPDH

Fig 8D

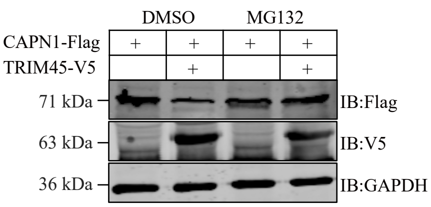
DMSO
MG132
CAPN1-Flag
TRIM45-V5
71 kDa
63 kDa
36 kDa
IB:Flag
IB:V5
IB:GAPDH

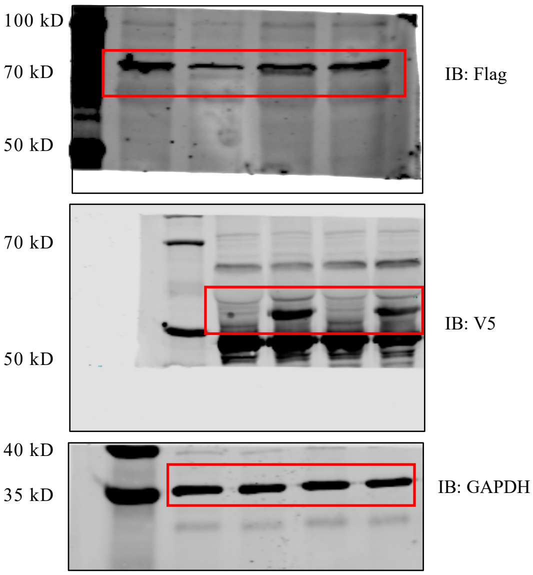
100 kD
70 kD
50 kD
IB: Flag
70 kD
50 kD
IB: V5
40 kD
35 kD
IB: GAPDH

Fig 8E

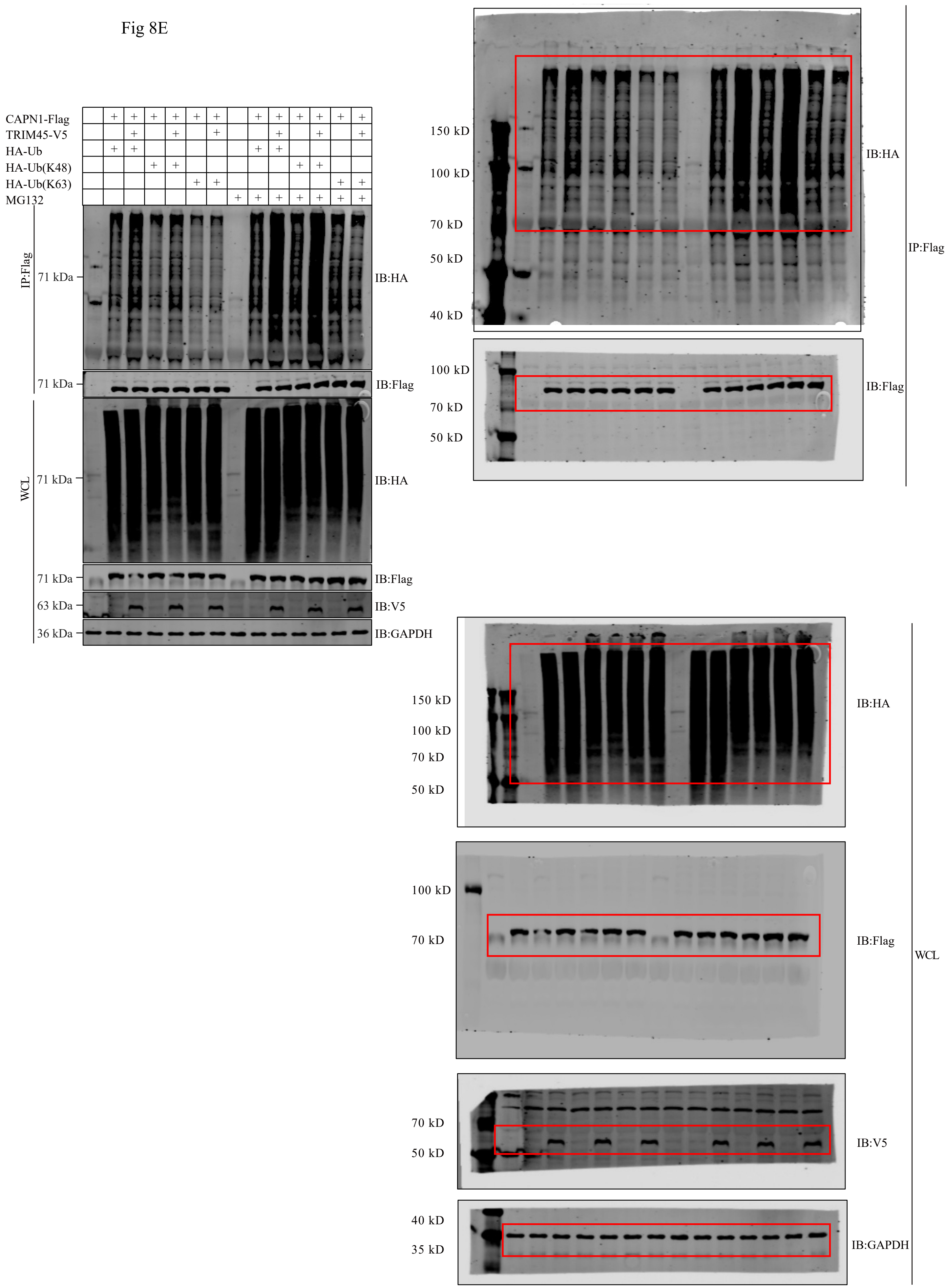

CAPN1-Flag
TRIM45-V5
HA-Ub
HA-Ub(K48)
HA-Ub(K63)
MG132
IP:Flag
71 kDa
IB:HA
71 kDa
IB:Flag
WCL
71 kDa
IB:HA
71 kDa
IB:Flag
63 kDa
IB:V5
36 kDa
IB:GAPDH
150 kD
100 kD
70 kD
50 kD
40 kD
IB:HA
IP:Flag
100 kD
70 kD
50 kD
IB:Flag
150 kD
100 kD
70 kD
50 kD
IB:HA
100 kD
70 kD
IB:Flag
WCL
70 kD
50 kD
IB:V5
40 kD
35 kD
IB:GAPDH

Fig 8F

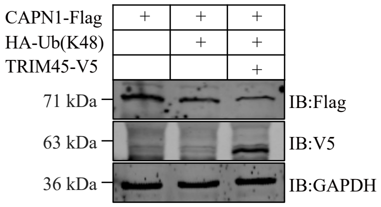
CAPN1-Flag
HA-Ub(K48)
TRIM45-V5
+ + +
+ +
+
71 kDa
63 kDa
36 kDa
IB:Flag
IB:V5
IB:GAPDH

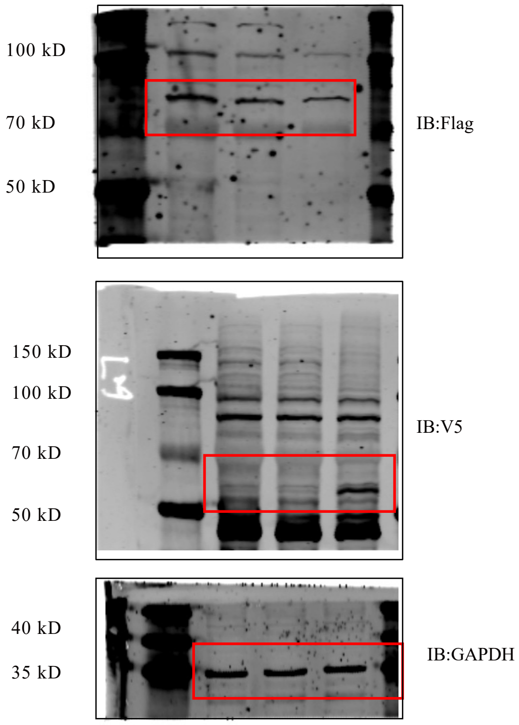
100 kD
70 kD
50 kD
IB:Flag
150 kD
100 kD
70 kD
50 kD
IB:V5
40 kD
35 kD
IB:GAPDH

Fig 8G

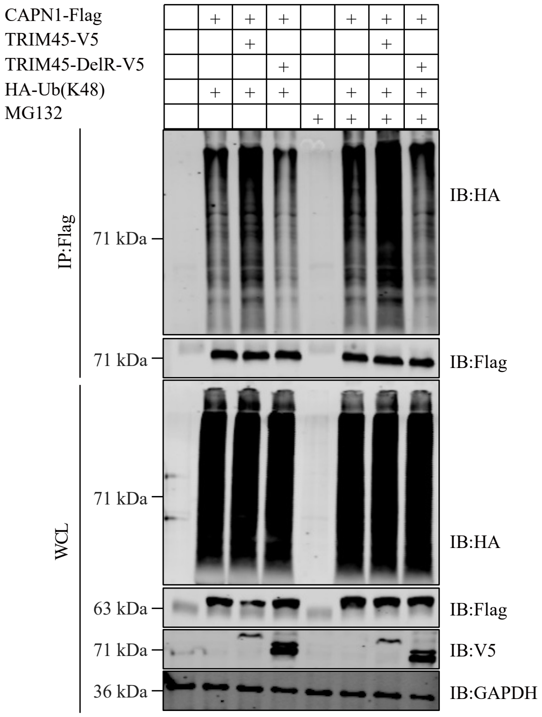

CAPN1-Flag
TRIM45-V5
TRIM45-DelR-V5
HA-Ub(K48)
MG132
IP:Flag
WCL
71 kDa
71 kDa
71 kDa
63 kDa
71 kDa
36 kDa
IB:HA
IB:Flag
IB:HA
IB:Flag
IB:V5
IB:GAPDH

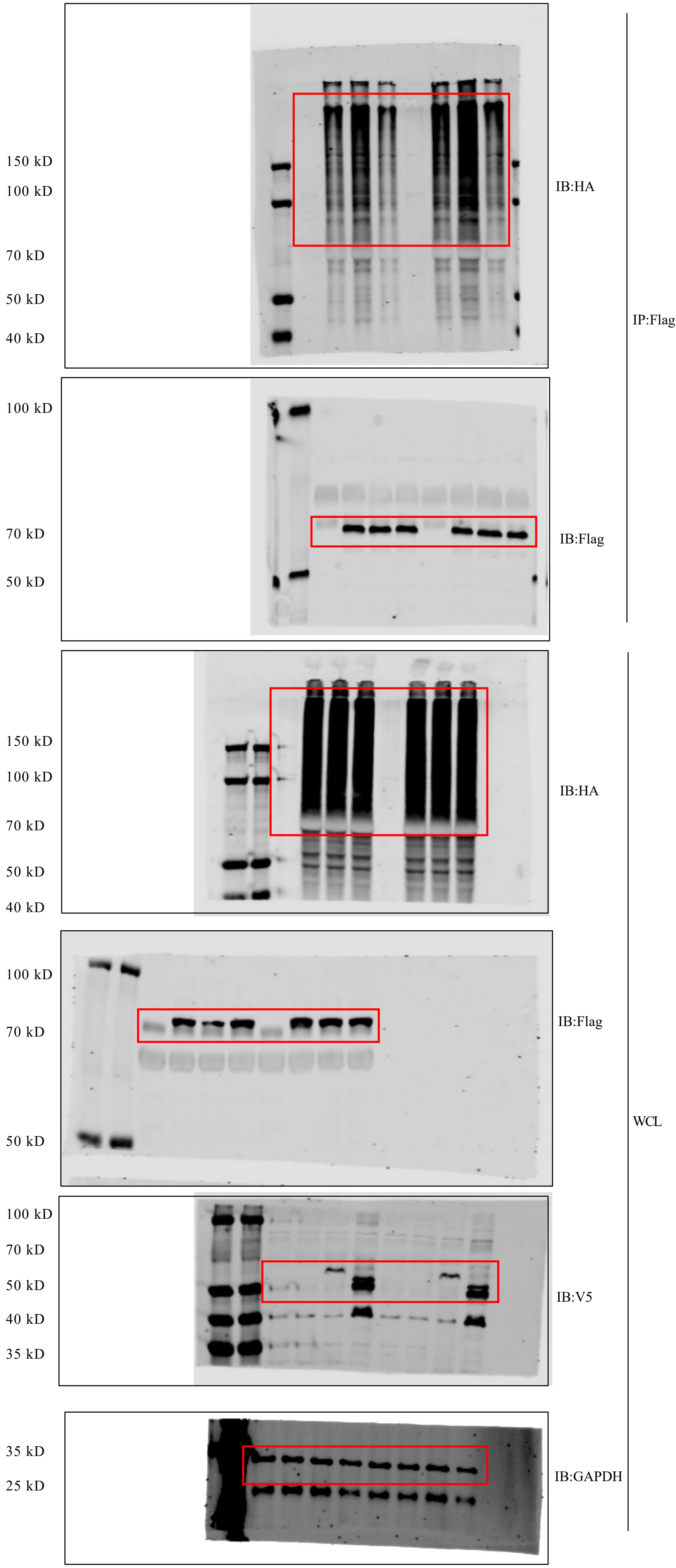

150 kD
100 kD
70 kD
50 kD
40 kD
IB:HA
100 kD
70 kD
50 kD
IB:Flag
IP:Flag
150 kD
100 kD
70 kD
50 kD
40 kD
IB:HA
100 kD
70 kD
50 kD
IB:Flag
100 kD
70 kD
50 kD
40 kD
35 kD
IB:V5
35 kD
25 kD
IB:GAPDH
WCL

Fig 8H

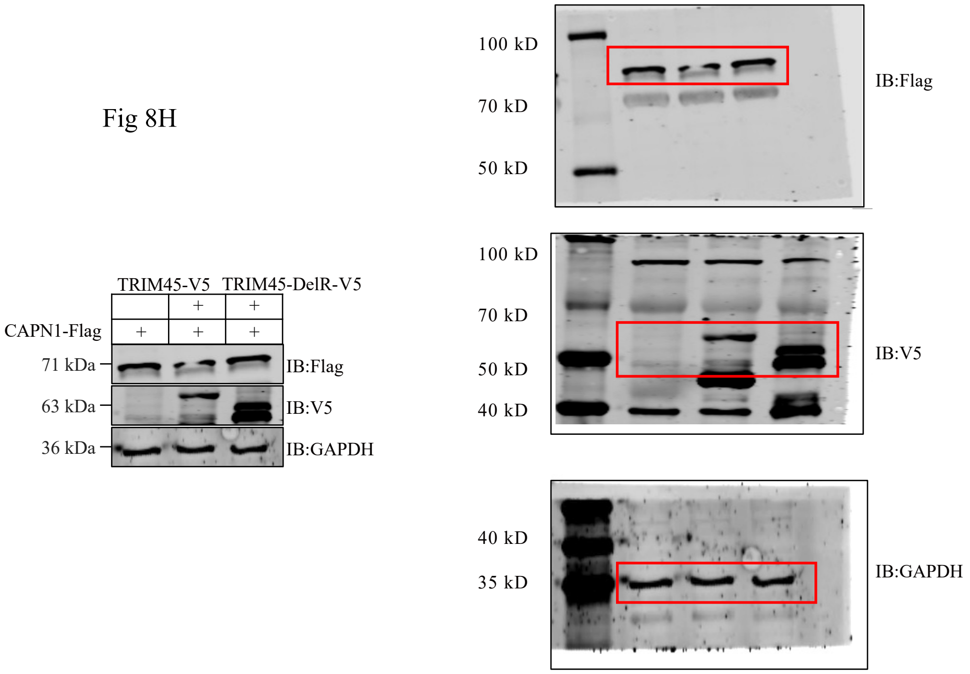
TRIM45-V5
TRIM45-DelR-V5
CAPN1-Flag
+
+ +
+ +
71 kDa
63 kDa
36 kDa
IB:Flag
IB:V5
IB:GAPDH
100 kD
70 kD
50 kD
IB:Flag
100 kD
70 kD
50 kD
40 kD
IB:V5
40 kD
35 kD
IB:GAPDH

Fig 8I

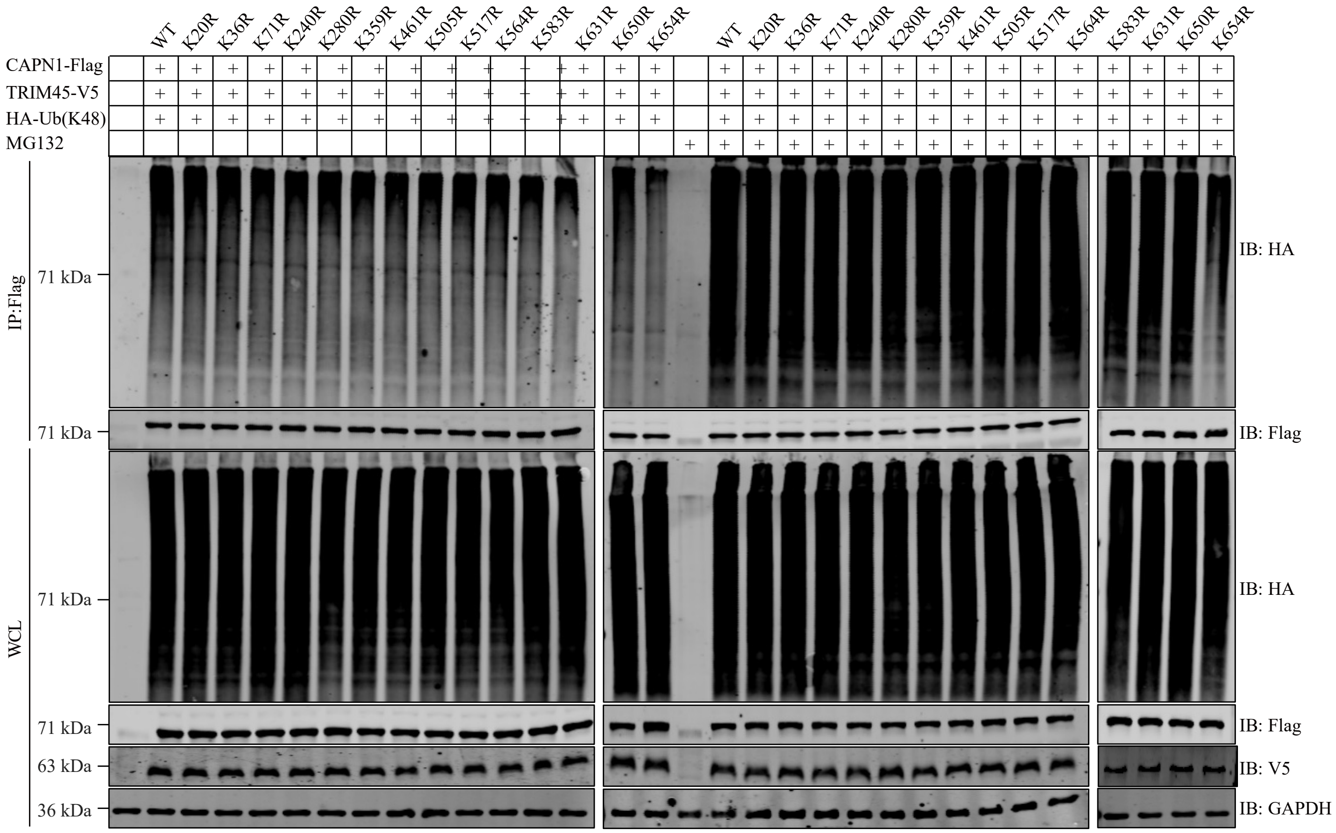

WT
K20R
K36R
K71R
K240R
K280R
K359R
K461R
K505R
K517R
K564R
K583R
K631R
K650R
K654R
CAPN1-Flag
TRIM45-V5
HA-Ub(K48)
MG132
IP:Flag
WCL
71 kDa
63 kDa
36 kDa
IB: HA
IB: Flag
IB: V5
IB: GAPDH

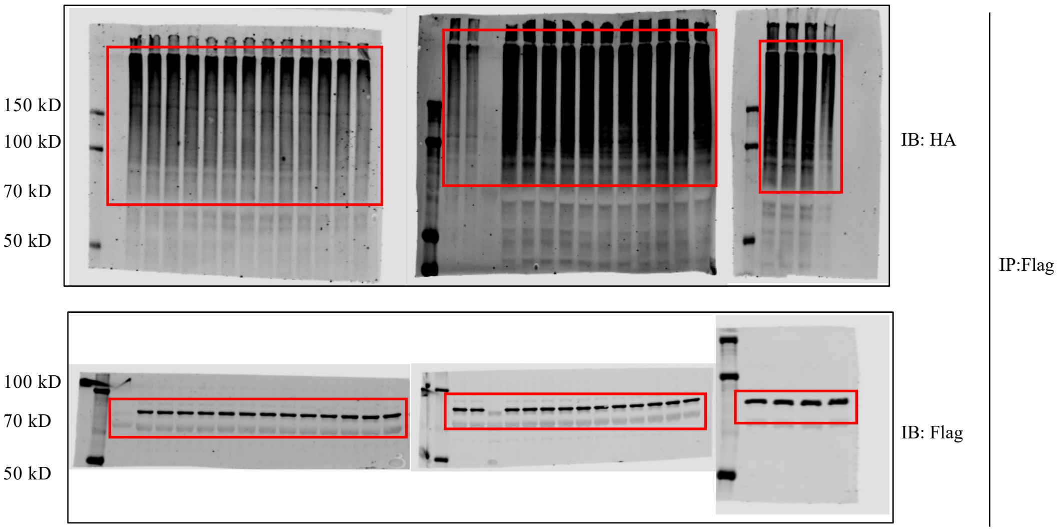

150 kD
100 kD
70 kD
50 kD
IB: HA
IB: Flag
IP:Flag

Fig 8I

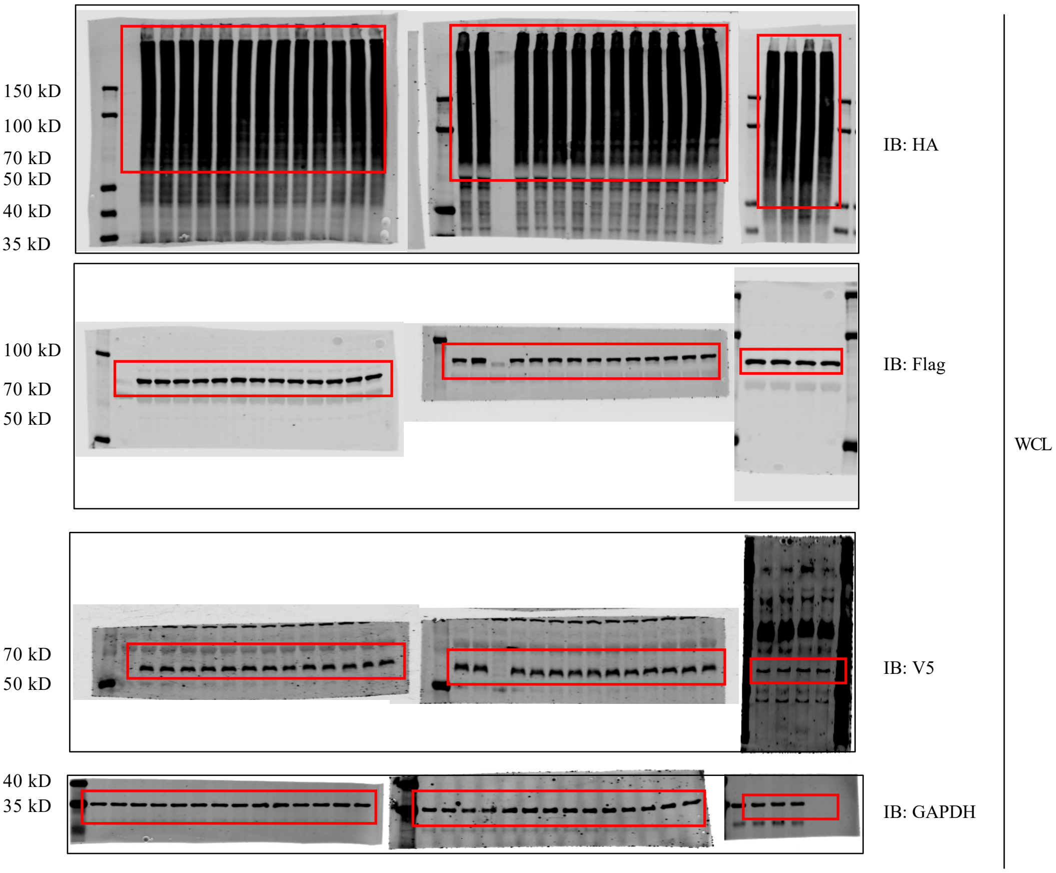
150 kD
100 kD
70 kD
50 kD
40 kD
35 kD
IB: HA
100 kD
70 kD
50 kD
IB: Flag
WCL
70 kD
50 kD
IB: V5
40 kD
35 kD
IB: GAPDH

Fig 8J

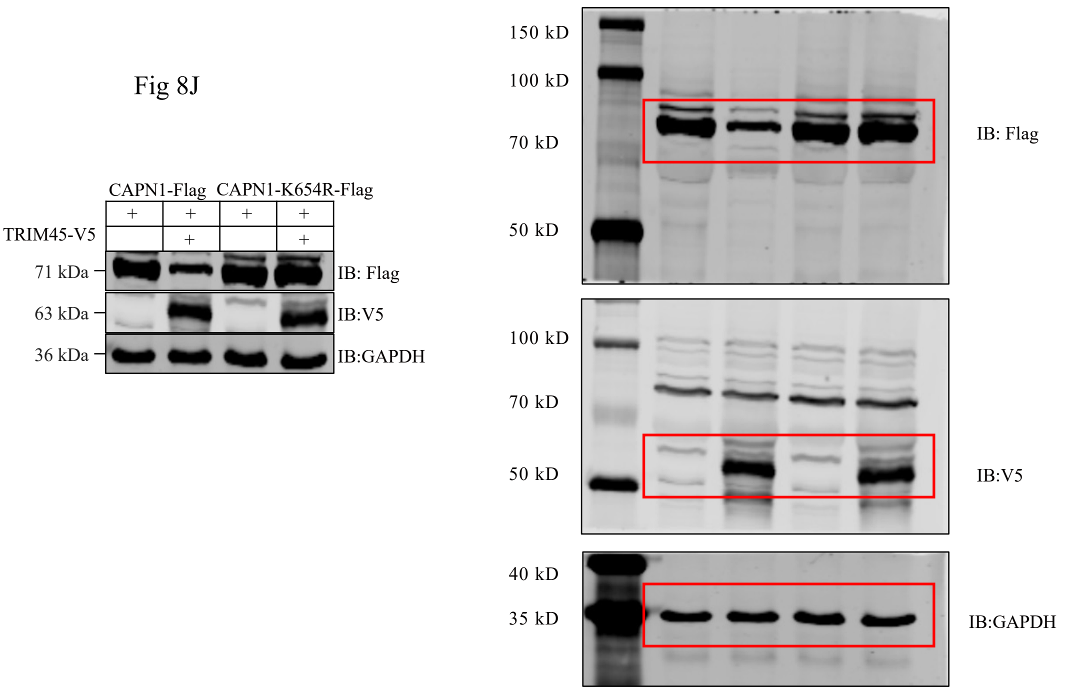
CAPN1-Flag
CAPN1-K654R-Flag
TRIM45-V5
71 kDa
63 kDa
36 kDa
IB: Flag
IB:V5
IB:GAPDH
150 kD
100 kD
70 kD
50 kD
IB: Flag
100 kD
70 kD
50 kD
IB:V5
40 kD
35 kD
IB:GAPDH

Fig S2A

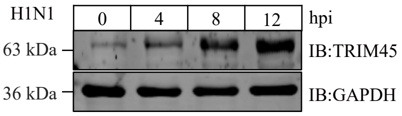

H1N1
0
4
8
12
hpi
63 kDa
36 kDa
IB:TRIM45
IB:GAPDH

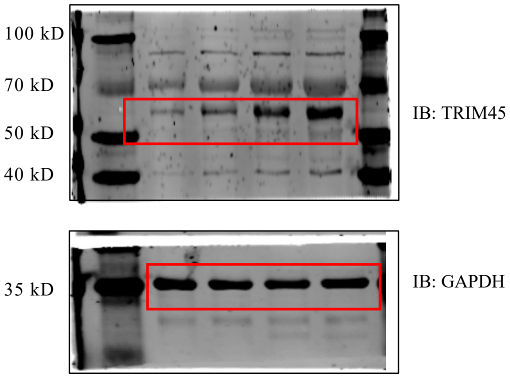

100 kD
70 kD
50 kD
40 kD
IB: TRIM45
35 kD
IB: GAPDH

Fig S3A

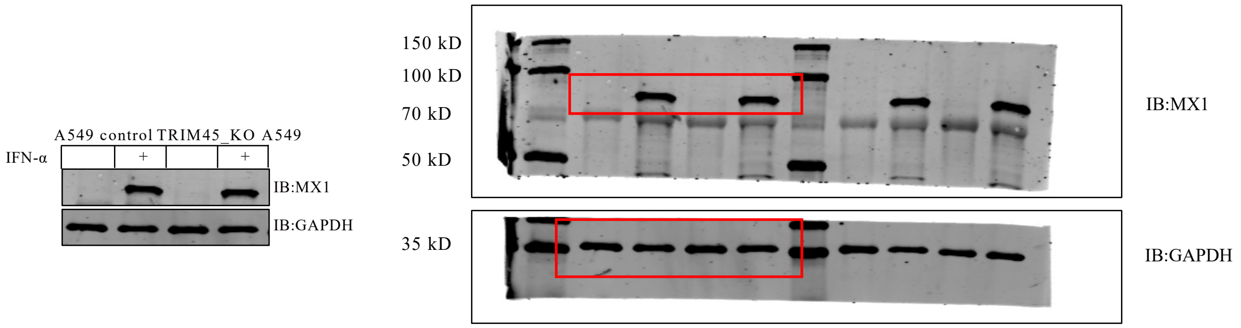
A549 control TRIM45_KO A549
IFN-α
+
+
IB:MX1
IB:GAPDH
150 kD
100 kD
70 kD
50 kD
IB:MX1
35 kD
IB:GAPDH

Fig S3B

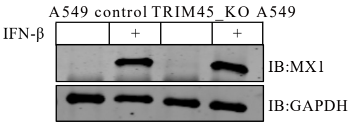

A549 control TRIM45_KO A549
IFN-β
+
+
IB:MX1
IB:GAPDH

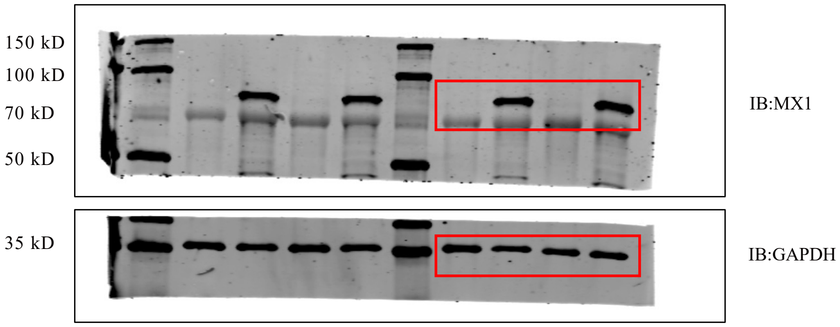

150 kD
100 kD
70 kD
50 kD
35 kD
IB:MX1
IB:GAPDH

Fig S5A

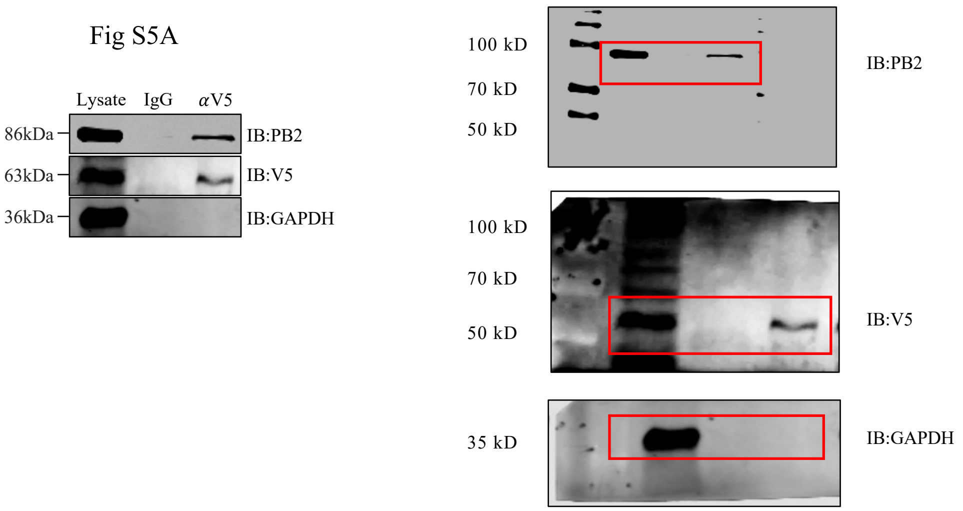

Lysate
IgG
αV5
86kDa
63kDa
36kDa
IB:PB2
IB:V5
IB:GAPDH
100 kD
70 kD
50 kD
IB:PB2
100 kD
70 kD
50 kD
IB:V5
35 kD
IB:GAPDH

Fig S5B

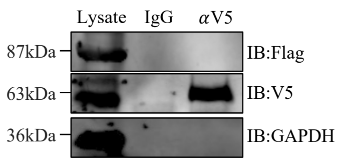
Lysate
IgG
αV5
87kDa
63kDa
36kDa
IB:Flag
IB:V5
IB:GAPDH

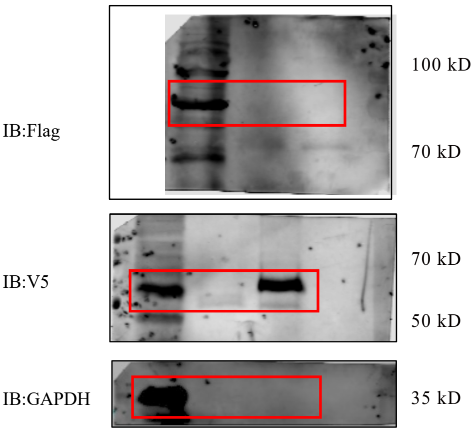
IB:Flag
IB:V5
IB:GAPDH
100 kD
70 kD
70 kD
50 kD
35 kD

Fig S5C

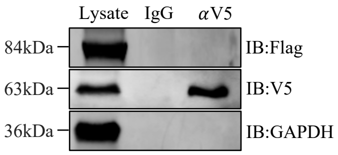

Lysate
IgG
αV5
84kDa
63kDa
36kDa
IB:Flag
IB:V5
IB:GAPDH

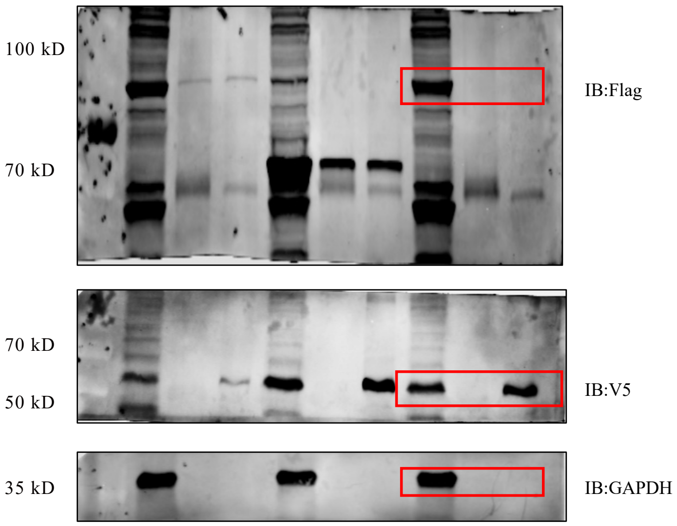

100 kD
70 kD
IB:Flag
70 kD
50 kD
IB:V5
35 kD
IB:GAPDH

Fig S5D

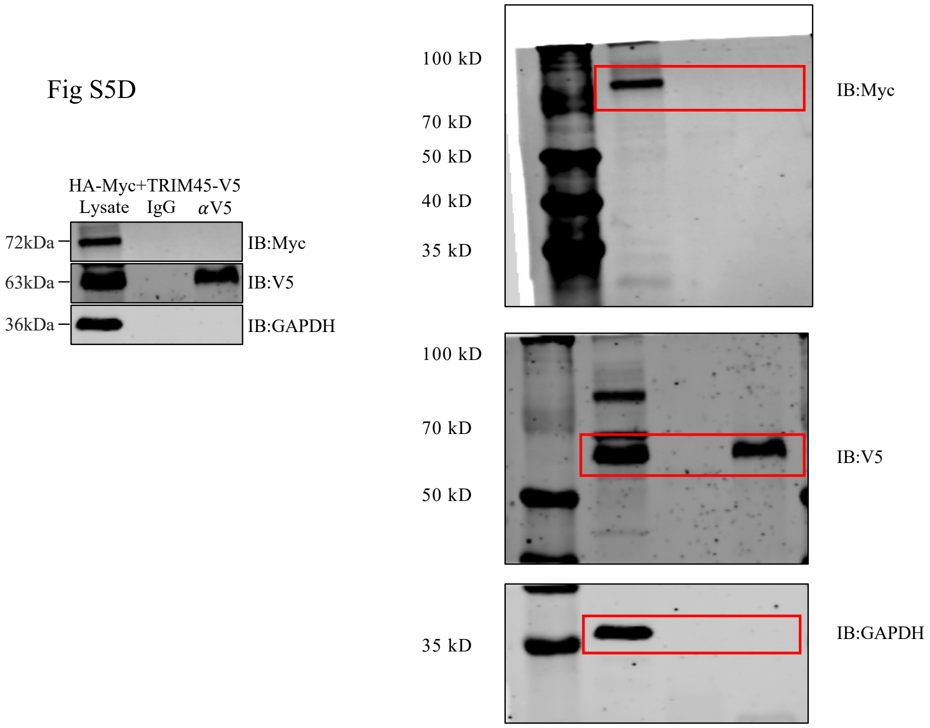
HA-Myc+TRIM45-V5
Lysate
IgG
αV5
72kDa
63kDa
36kDa
IB:Myc
IB:V5
IB:GAPDH
100 kD
70 kD
50 kD
40 kD
35 kD
IB:Myc
100 kD
70 kD
50 kD
IB:V5
35 kD
IB:GAPDH

Fig S5E

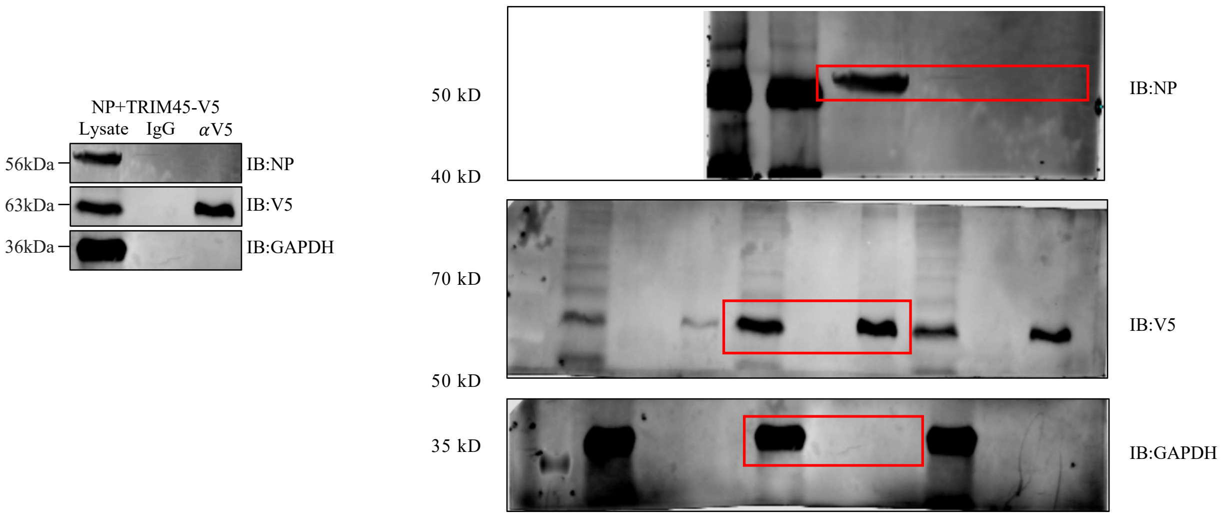
NP+TRIM45-V5
Lysate
IgG
αV5
56kDa
63kDa
36kDa
IB:NP
IB:V5
IB:GAPDH
50 kD
40 kD
70 kD
50 kD
35 kD
IB:NP
IB:V5
IB:GAPDH

Fig S5F

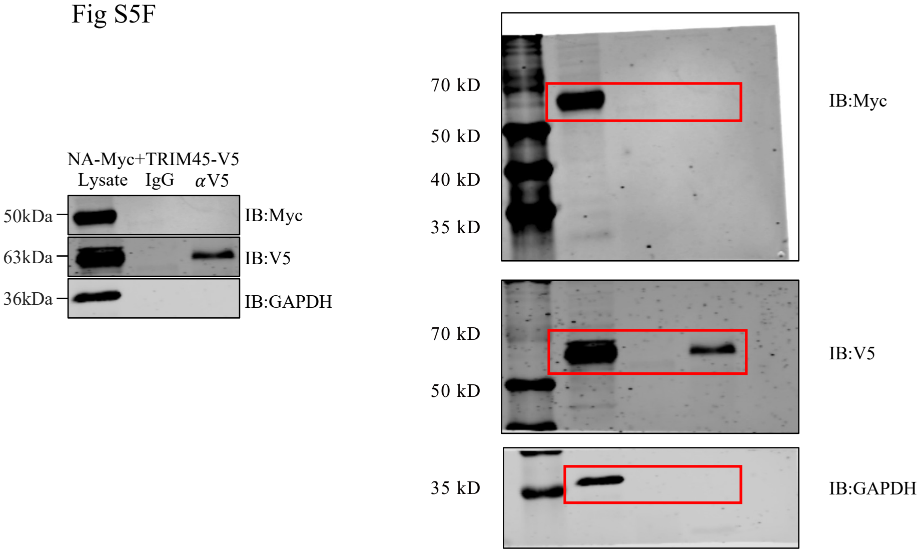

NA-Myc+TRIM45-V5
Lysate
IgG
αV5
50kDa
63kDa
36kDa
IB:Myc
IB:V5
IB:GAPDH
70 kD
50 kD
40 kD
35 kD
IB:Myc
70 kD
50 kD
IB:V5
35 kD
IB:GAPDH

Fig S5G

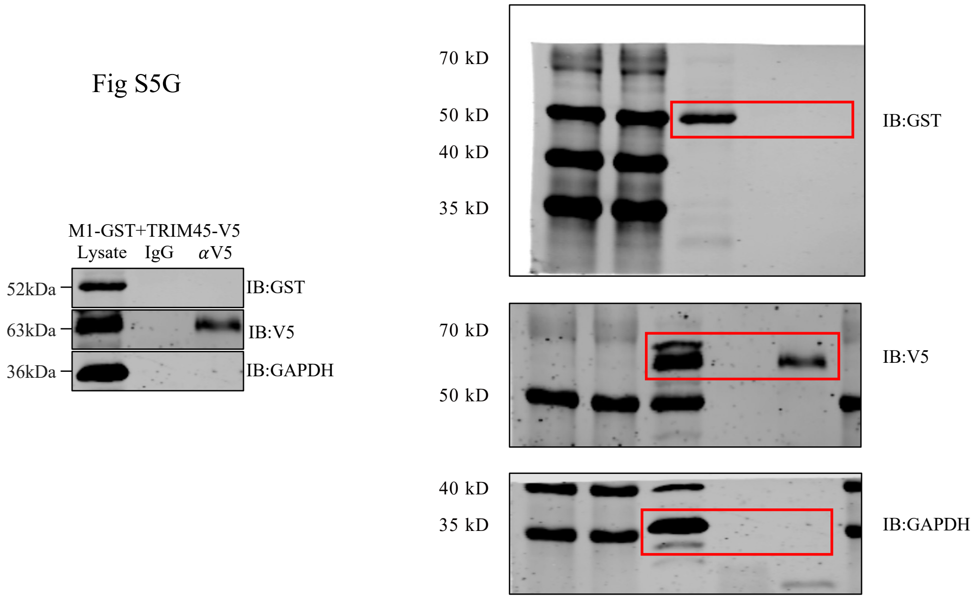

M1-GST+TRIM45-V5
Lysate
IgG
αV5
52kDa
63kDa
36kDa
IB:GST
IB:V5
IB:GAPDH
70 kD
50 kD
40 kD
35 kD
IB:GST
70 kD
50 kD
IB:V5
40 kD
35 kD
IB:GAPDH

Fig S5H

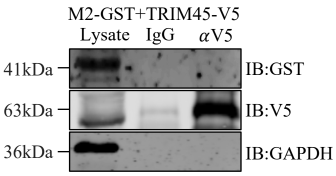

M2-GST+TRIM45-V5
Lysate IgG αV5
41kDa
IB:GST
63kDa
IB:V5
36kDa
IB:GAPDH

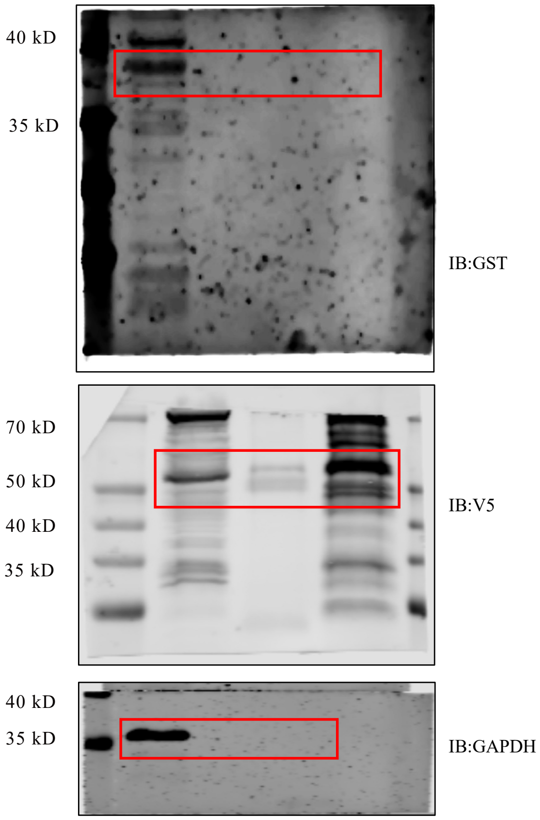

40 kD
35 kD
IB:GST
70 kD
50 kD
40 kD
35 kD
IB:V5
40 kD
35 kD
IB:GAPDH

Fig S5I

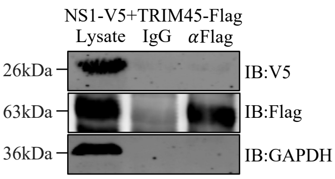
NS1-V5+TRIM45-Flag
Lysate
IgG
αFlag
26kDa
IB:V5
63kDa
IB:Flag
36kDa
IB:GAPDH

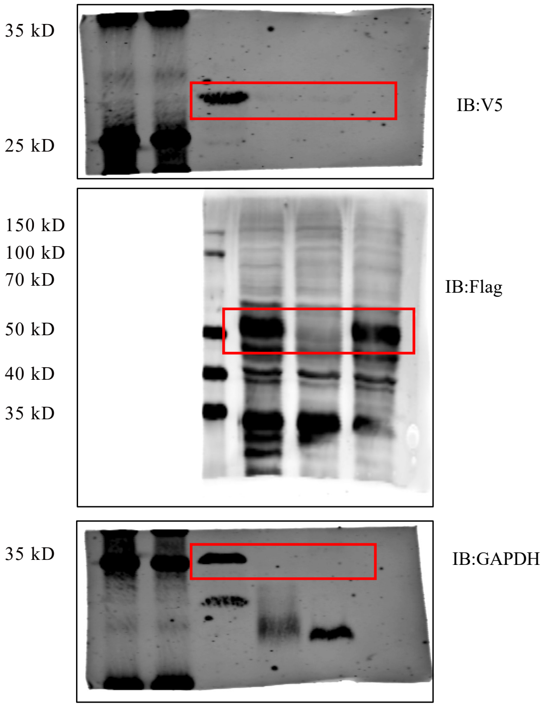
35 kD
25 kD
IB:V5
150 kD
100 kD
70 kD
50 kD
40 kD
35 kD
IB:Flag
35 kD
IB:GAPDH

Fig S5J

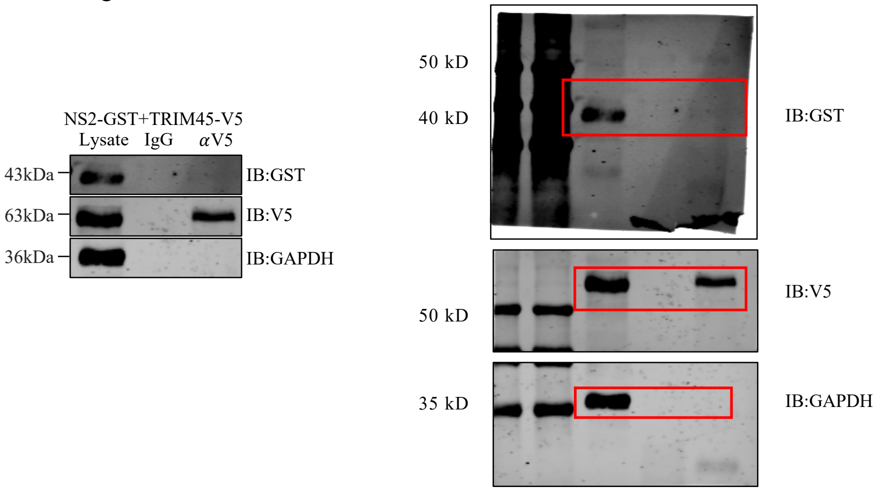
NS2-GST+TRIM45-V5
Lysate
IgG
αV5
43kDa
63kDa
36kDa
IB:GST
IB:V5
IB:GAPDH
50 kD
40 kD
IB:GST
50 kD
IB:V5
35 kD
IB:GAPDH

Fig S6A

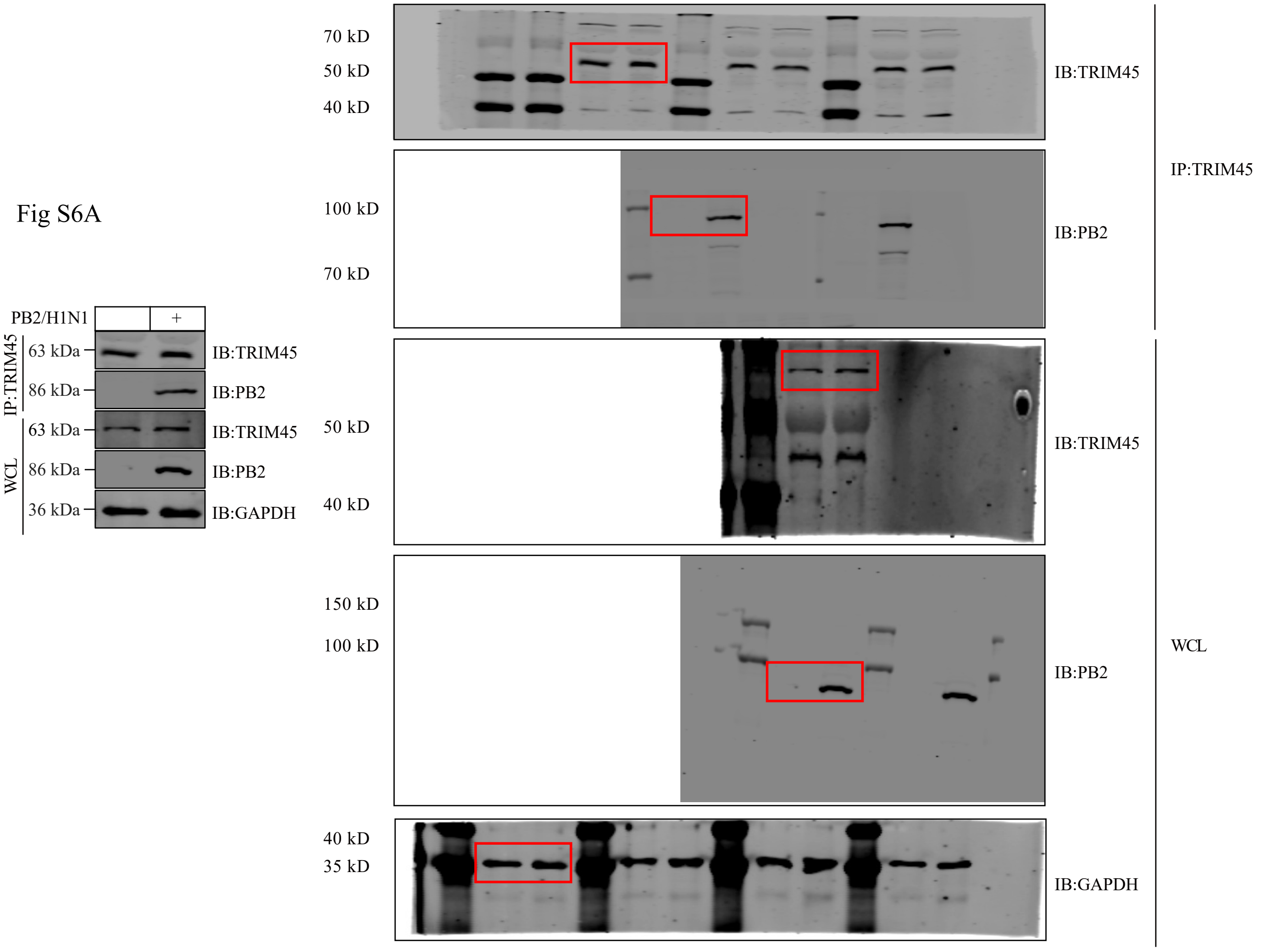
PB2/H1N1
+
IP:TRIM45
WCL
63 kDa
86 kDa
63 kDa
86 kDa
36 kDa
IB:TRIM45
IB:PB2
IB:TRIM45
IB:PB2
IB:GAPDH
70 kD
50 kD
40 kD
100 kD
70 kD
50 kD
40 kD
150 kD
100 kD
40 kD
35 kD
IB:TRIM45
IB:PB2
IB:TRIM45
IB:PB2
IB:GAPDH
IP:TRIM45
WCL

Fig S6B

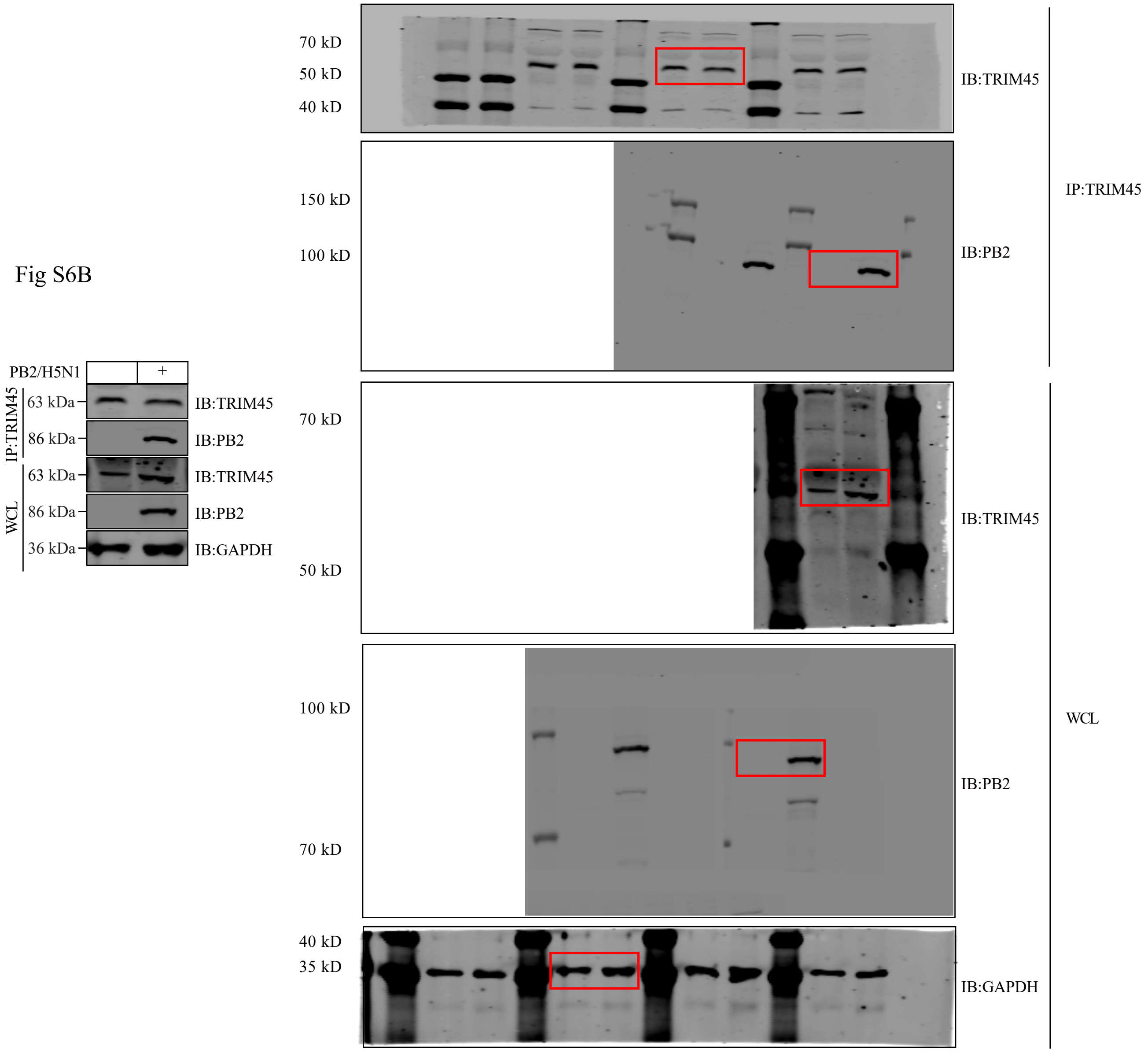
PB2/H5N1
+
IP:TRIM45
63 kDa
IB:TRIM45
86 kDa
IB:PB2
WCL
63 kDa
IB:TRIM45
86 kDa
IB:PB2
36 kDa
IB:GAPDH
70 kD
50 kD
40 kD
IB:TRIM45
150 kD
100 kD
IB:PB2
IP:TRIM45
70 kD
50 kD
IB:TRIM45
100 kD
70 kD
IB:PB2
WCL
40 kD
35 kD
IB:GAPDH

Fig S6C

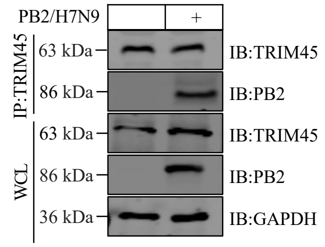
PB2/H7N9
+
IP:TRIM45
WCL
63 kDa
86 kDa
63 kDa
86 kDa
36 kDa
IB:TRIM45
IB:PB2
IB:TRIM45
IB:PB2
IB:GAPDH

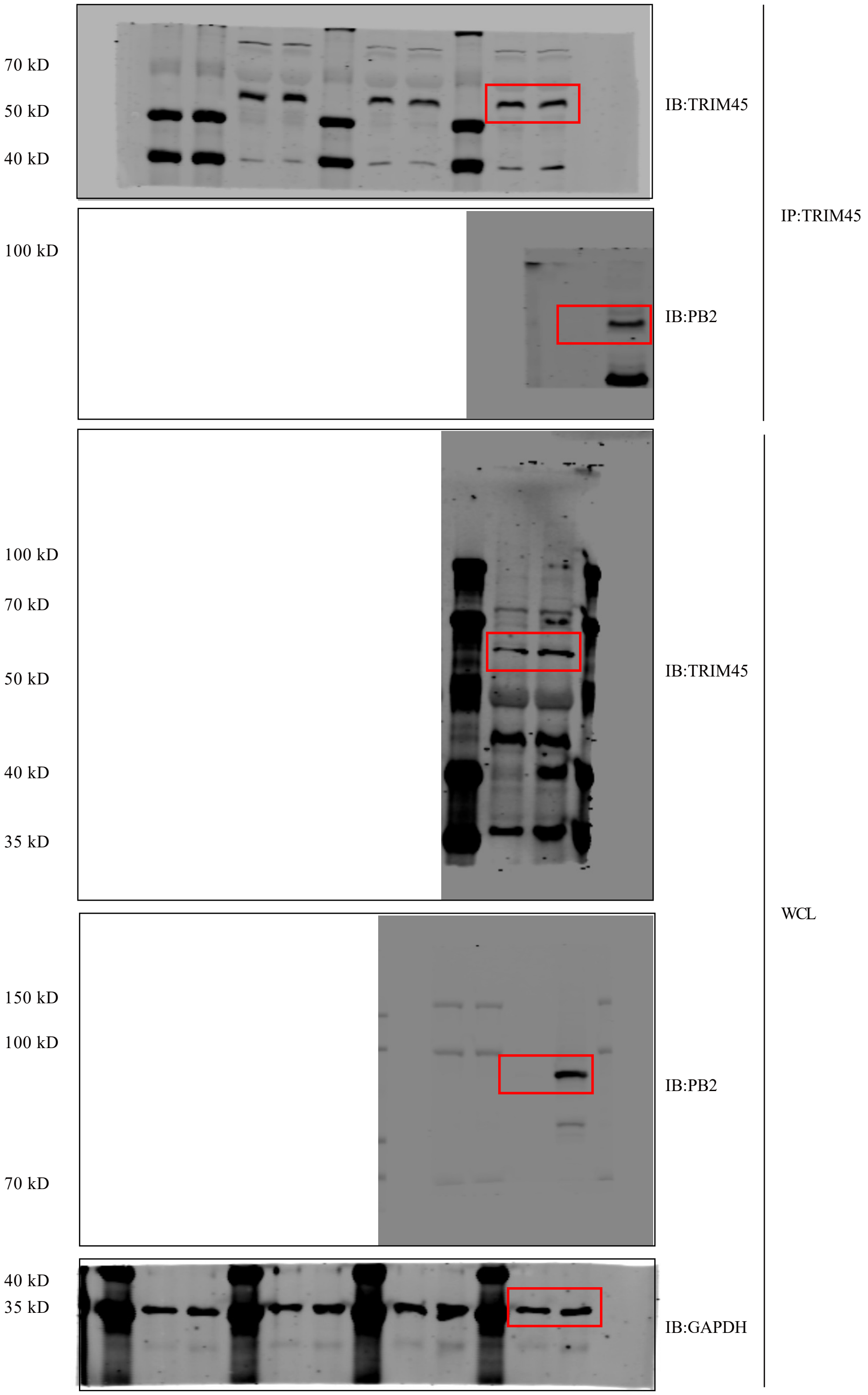
70 kD
50 kD
40 kD
IB:TRIM45
100 kD
IB:PB2
IP:TRIM45
100 kD
70 kD
50 kD
40 kD
35 kD
IB:TRIM45
WCL
150 kD
100 kD
70 kD
IB:PB2
40 kD
35 kD
IB:GAPDH

Fig S8A

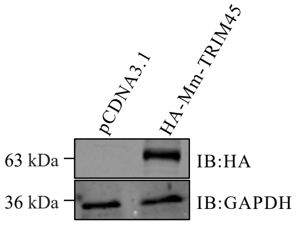

pCDNA3.1
HA-Mm-TRIM45
63 kDa
IB:HA
36 kDa
IB:GAPDH

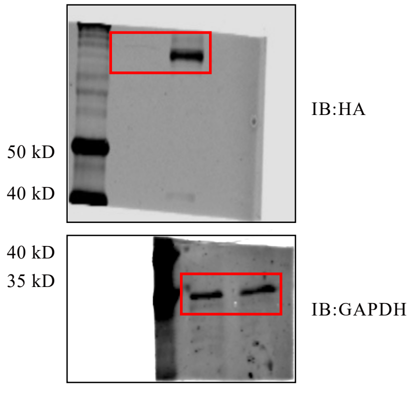

50 kD
40 kD
IB:HA
40 kD
35 kD
IB:GAPDH

Fig S8B

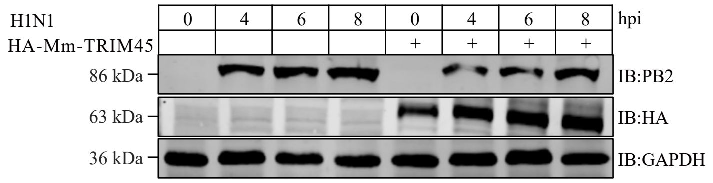
H1N1
0
4
6
8
0
4
6
8
hpi
HA-Mm-TRIM45
+
+
+
+
86 kDa
IB:PB2
63 kDa
IB:HA
36 kDa
IB:GAPDH

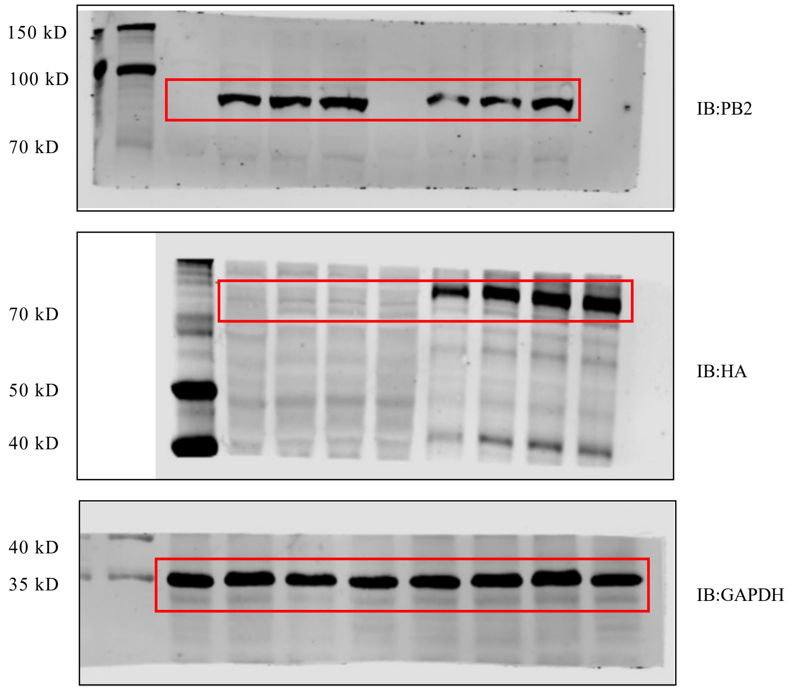
150 kD
100 kD
70 kD
IB:PB2
70 kD
50 kD
40 kD
IB:HA
40 kD
35 kD
IB:GAPDH

Fig S10

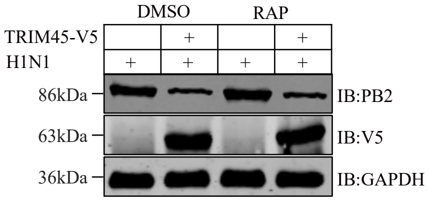

DMSO
RAP
TRIM45-V5
H1N1
86kDa
63kDa
36kDa
IB:PB2
IB:V5
IB:GAPDH

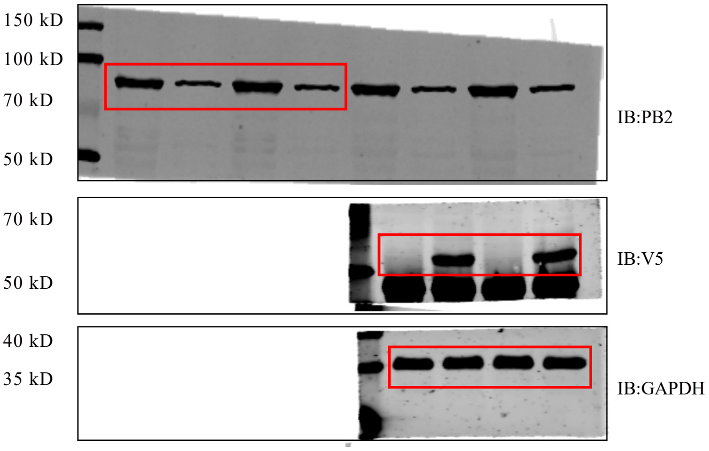

150 kD
100 kD
70 kD
50 kD
IB:PB2
70 kD
50 kD
IB:V5
40 kD
35 kD
IB:GAPDH

Fig S12A

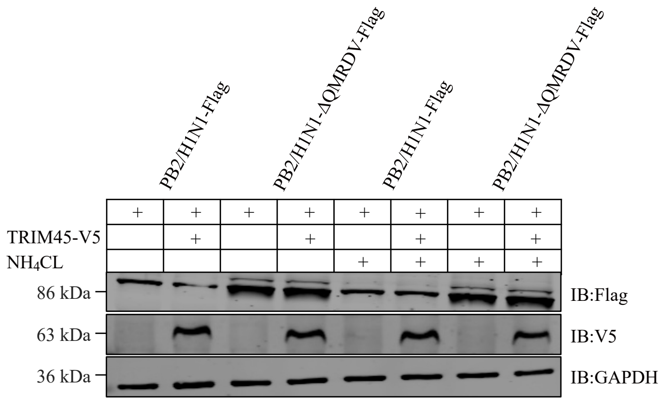
PB2/H1N1-Flag
PB2/H1N1-ΔQMRDV-Flag
PB2/H1N1-Flag
PB2/H1N1-ΔQMRDV-Flag
TRIM45-V5
NH4CL
86 kDa
63 kDa
36 kDa
IB:Flag
IB:V5
IB:GAPDH

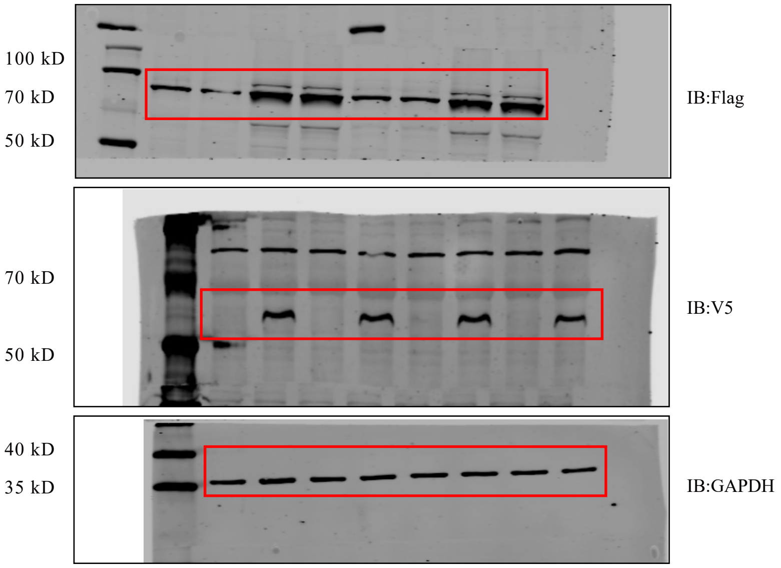
100 kD
70 kD
50 kD
IB:Flag
70 kD
50 kD
IB:V5
40 kD
35 kD
IB:GAPDH

Fig S12B

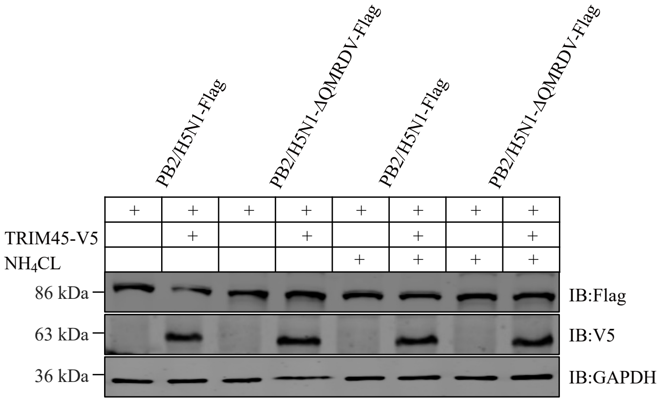
PB2/H5N1-Flag
PB2/H5N1-ΔQMRDV-Flag
PB2/H5N1-Flag
PB2/H5N1-ΔQMRDV-Flag
TRIM45-V5
NH4CL
86 kDa
63 kDa
36 kDa
IB:Flag
IB:V5
IB:GAPDH

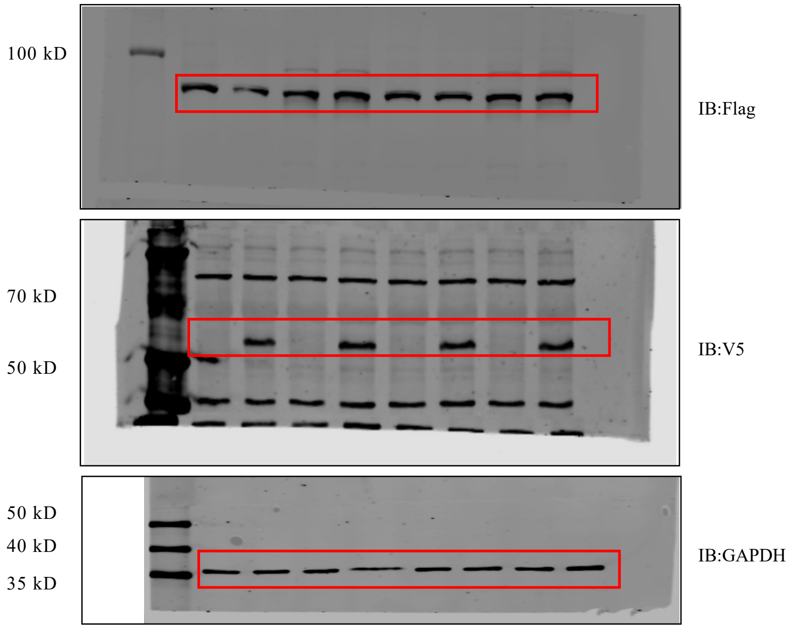
100 kD
IB:Flag
70 kD
50 kD
IB:V5
50 kD
40 kD
35 kD
IB:GAPDH

Fig S12C

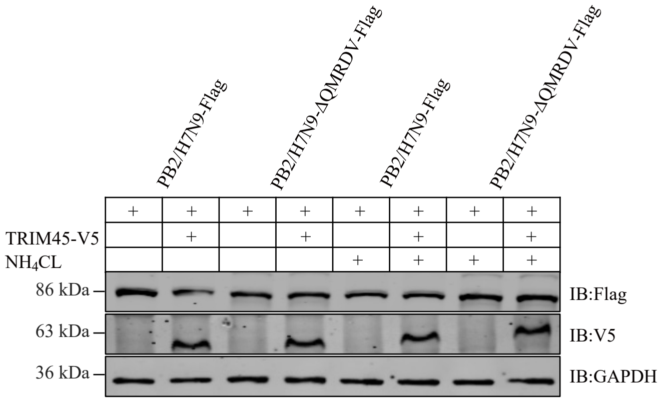

PB2/H7N9-Flag
PB2/H7N9-ΔQMRDV-Flag
PB2/H7N9-Flag
PB2/H7N9-ΔQMRDV-Flag
TRIM45-V5
NH4CL
86 kDa
63 kDa
36 kDa
IB:Flag
IB:V5
IB:GAPDH

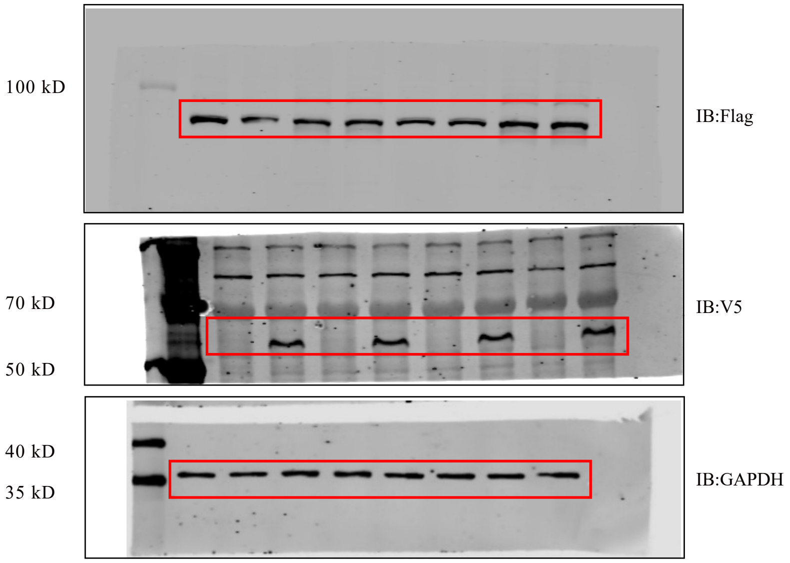

100 kD
70 kD
50 kD
40 kD
35 kD
IB:Flag
IB:V5
IB:GAPDH

Fig S13A

TRIM45-V5		+			+	
TRIM45-DelR-V5			+			+
H1N1	+	+	+			
H1N1 PB2-Q602A				+	+	+

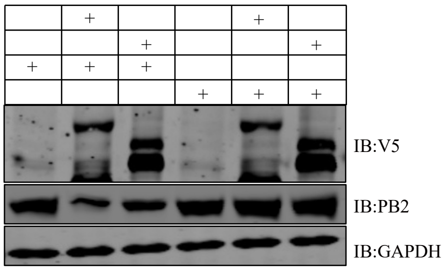

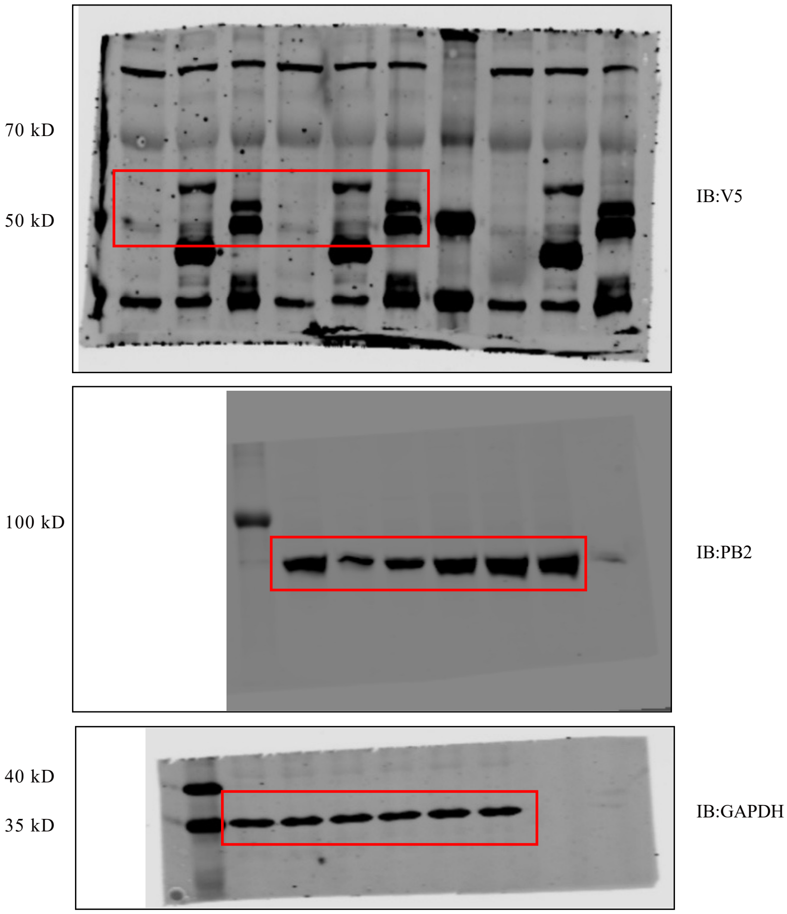

Fig S14

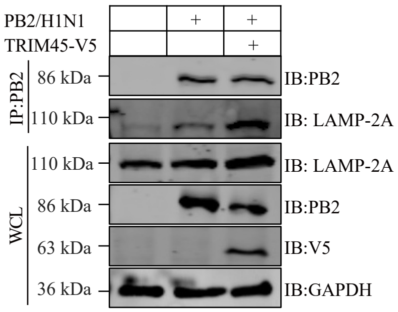
PB2/H1N1
TRIM45-V5
+
+
+
IP:PB2
86 kDa
110 kDa
IB:PB2
IB: LAMP-2A
WCL
110 kDa
86 kDa
63 kDa
36 kDa
IB: LAMP-2A
IB:PB2
IB:V5
IB:GAPDH

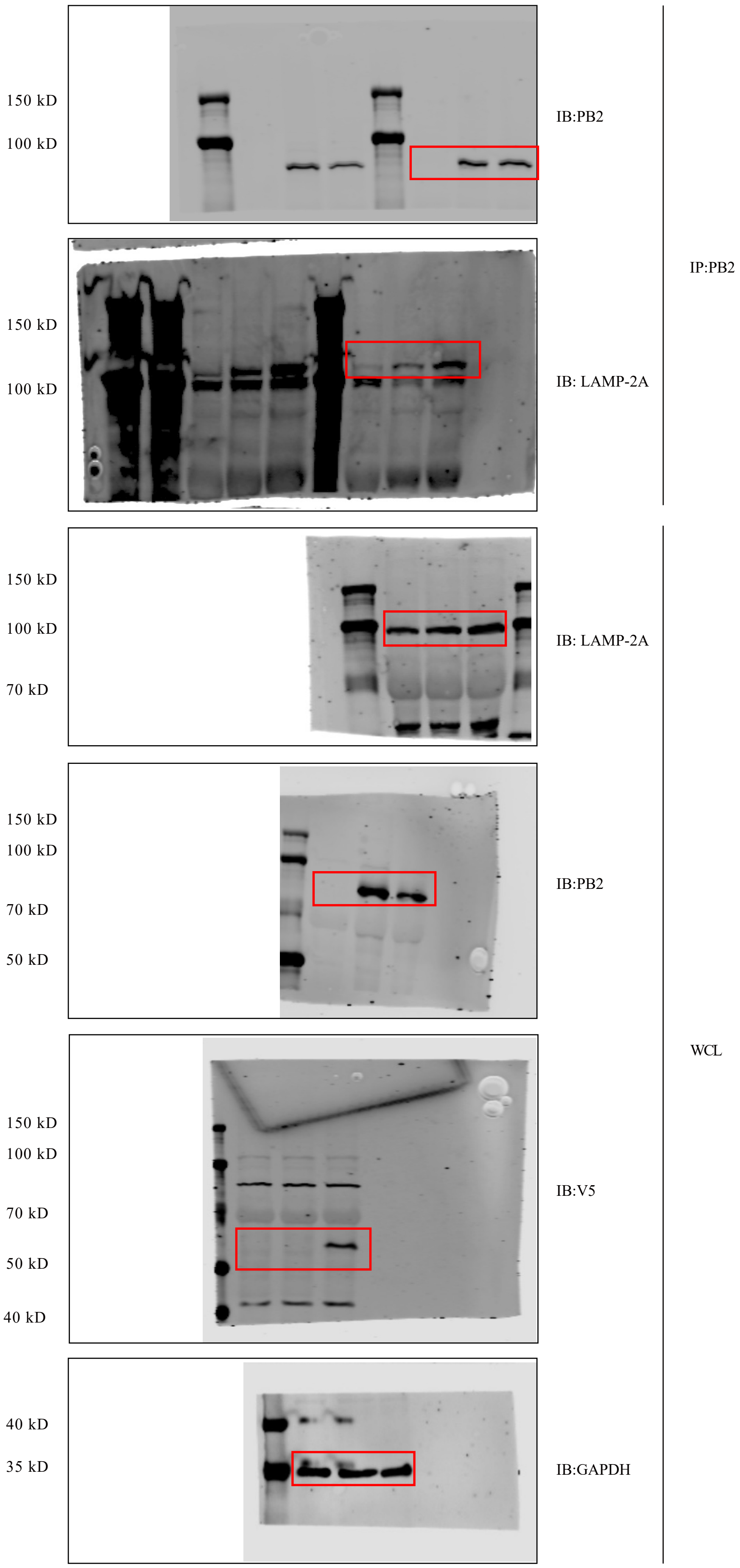
150 kD
100 kD
IB:PB2
150 kD
100 kD
IB: LAMP-2A
IP:PB2
150 kD
100 kD
70 kD
IB: LAMP-2A
150 kD
100 kD
70 kD
50 kD
IB:PB2
150 kD
100 kD
70 kD
50 kD
40 kD
IB:V5
WCL
40 kD
35 kD
IB:GAPDH

Fig S15

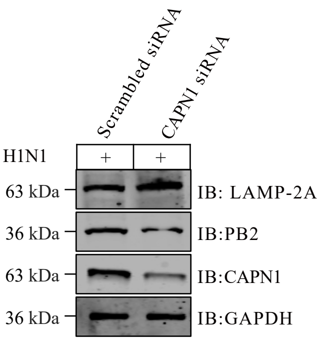

Scrambled siRNA
CAPN1 siRNA
H1N1
+
+
63 kDa
IB: LAMP-2A
36 kDa
IB:PB2
63 kDa
IB:CAPN1
36 kDa
IB:GAPDH

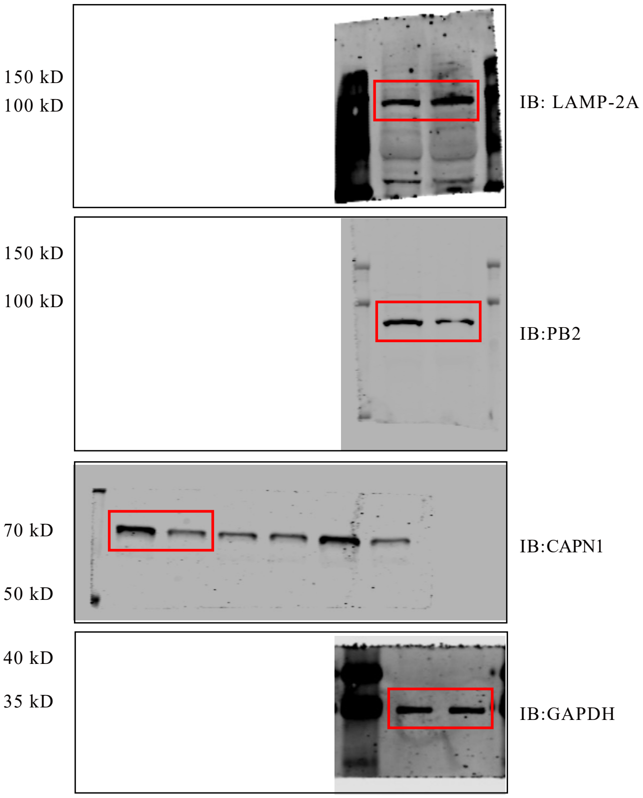

150 kD
100 kD
IB: LAMP-2A
150 kD
100 kD
IB:PB2
70 kD
50 kD
IB:CAPN1
40 kD
35 kD
IB:GAPDH

Fig S16

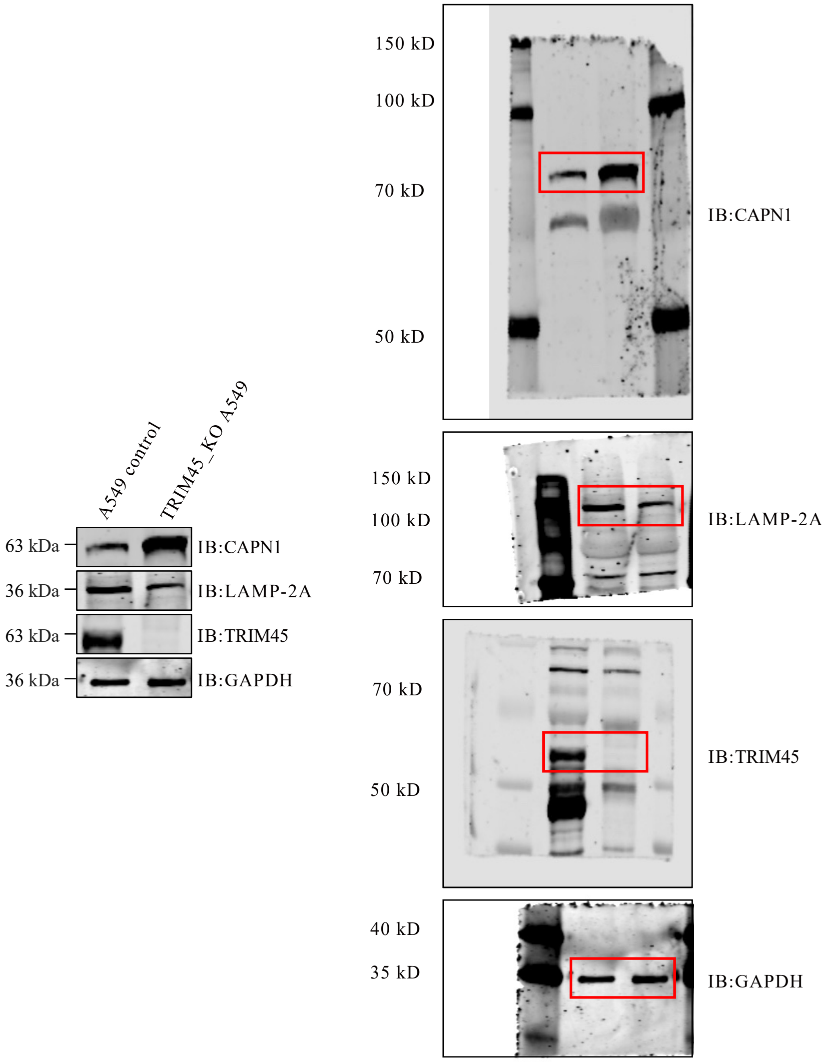
A549 control
TRIM45_KO A549
63 kDa
IB:CAPN1
36 kDa
IB:LAMP-2A
63 kDa
IB:TRIM45
36 kDa
IB:GAPDH
150 kD
100 kD
70 kD
50 kD
IB:CAPN1
150 kD
100 kD
70 kD
IB:LAMP-2A
70 kD
50 kD
IB:TRIM45
40 kD
35 kD
IB:GAPDH

Fig S17A

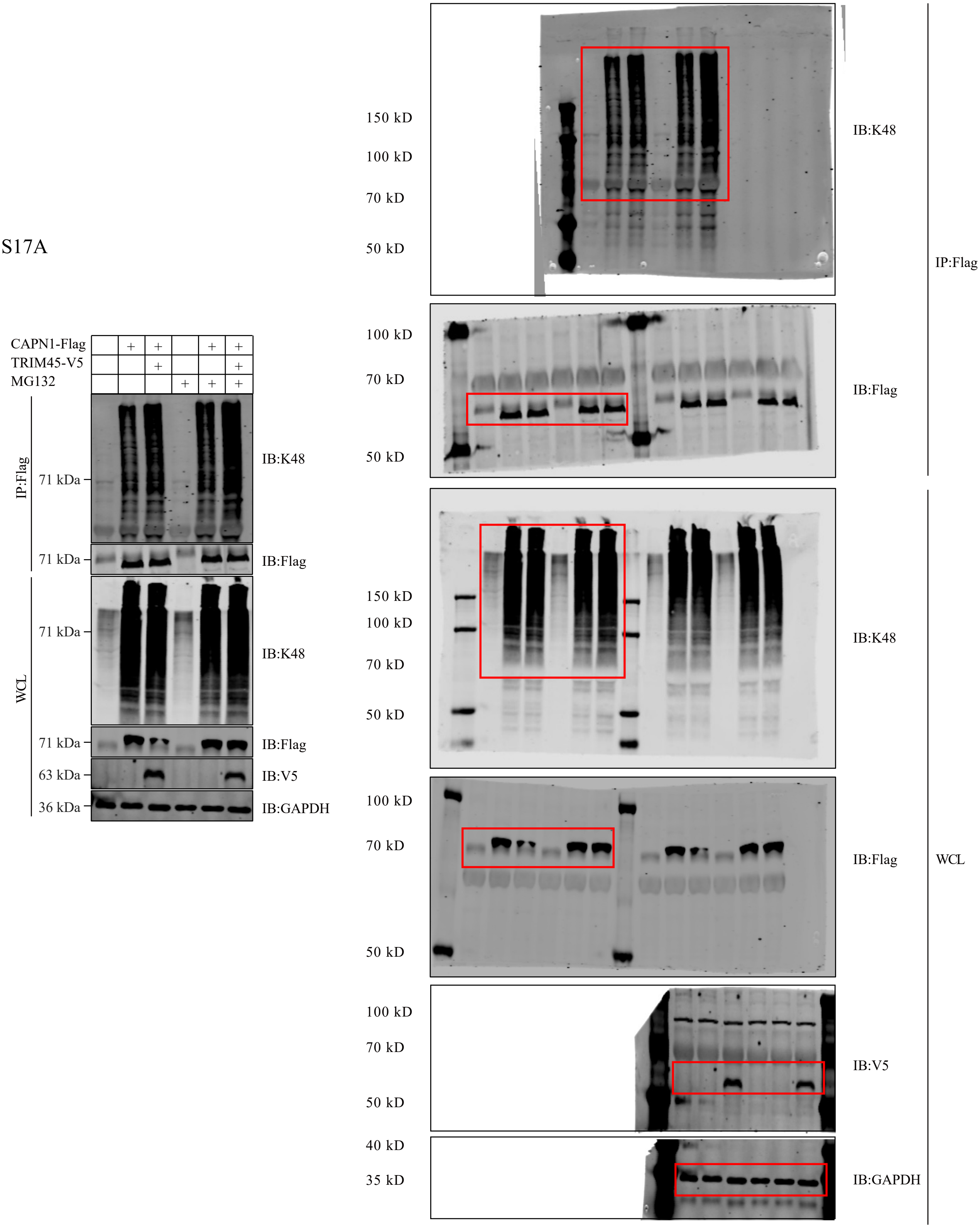
CAPN1-Flag
TRIM45-V5
MG132
+ + + +
+ +
+ + +
IP:Flag
WCL
71 kDa
71 kDa
71 kDa
71 kDa
63 kDa
36 kDa
IB:K48
IB:Flag
IB:K48
IB:Flag
IB:V5
IB:GAPDH
150 kD
100 kD
70 kD
50 kD
100 kD
70 kD
50 kD
150 kD
100 kD
70 kD
50 kD
100 kD
70 kD
50 kD
100 kD
70 kD
50 kD
40 kD
35 kD
IB:K48
IB:Flag
IB:K48
IB:Flag
IB:V5
IB:GAPDH
IP:Flag
WCL

Fig S17B

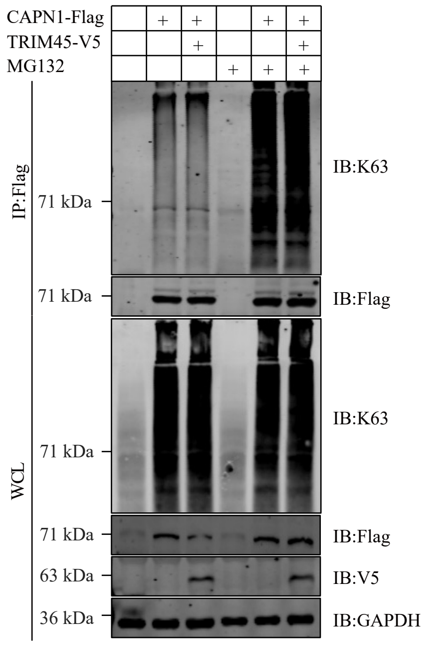
CAPN1-Flag
TRIM45-V5
MG132
IP:Flag
WCL
71 kDa
71 kDa
71 kDa
71 kDa
63 kDa
36 kDa
IB:K63
IB:Flag
IB:K63
IB:Flag
IB:V5
IB:GAPDH

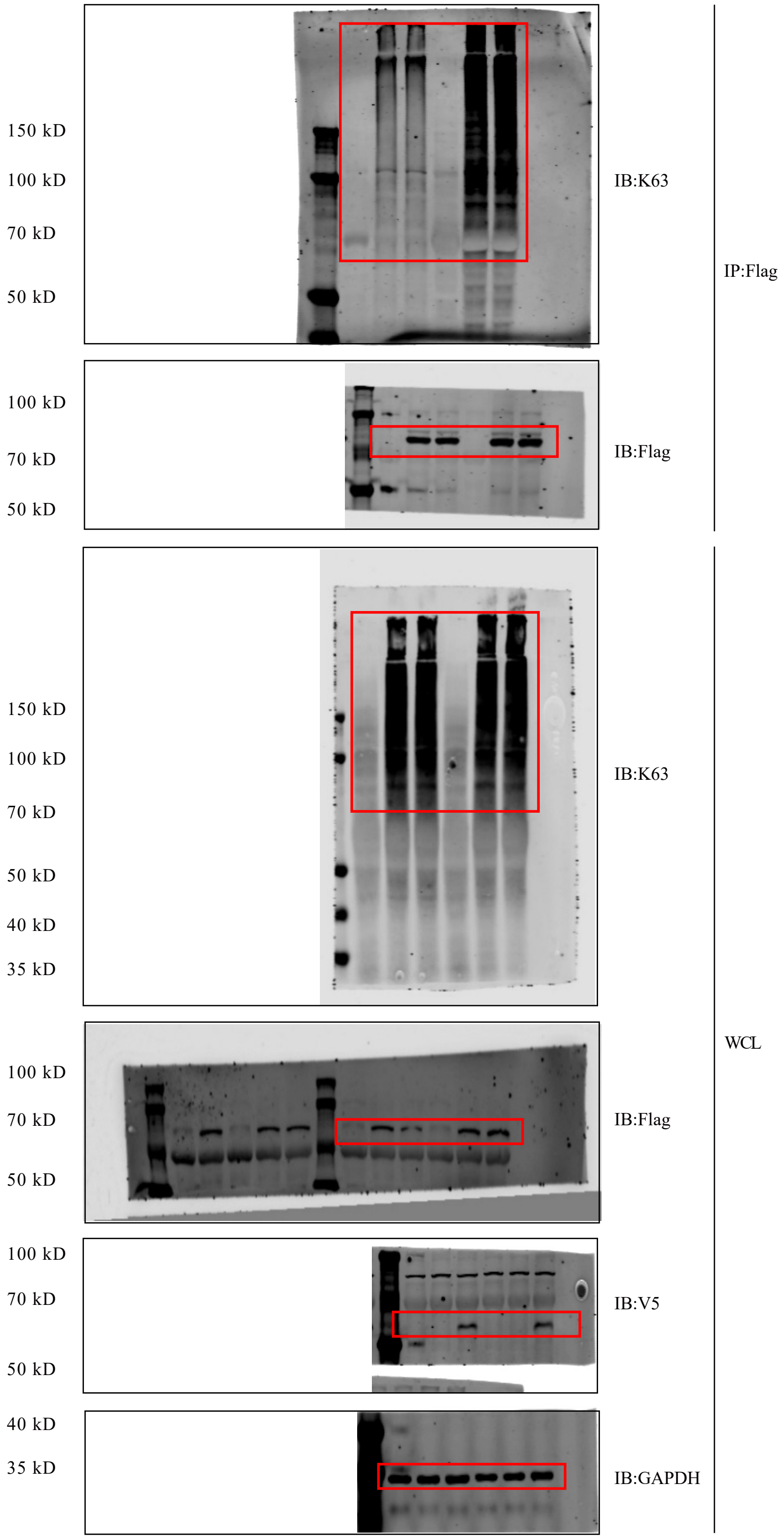
150 kD
100 kD
70 kD
50 kD
IB:K63
100 kD
70 kD
50 kD
IB:Flag
IP:Flag
150 kD
100 kD
70 kD
50 kD
40 kD
35 kD
IB:K63
100 kD
70 kD
50 kD
IB:Flag
100 kD
70 kD
50 kD
IB:V5
40 kD
35 kD
IB:GAPDH
WCL

Fig S17C

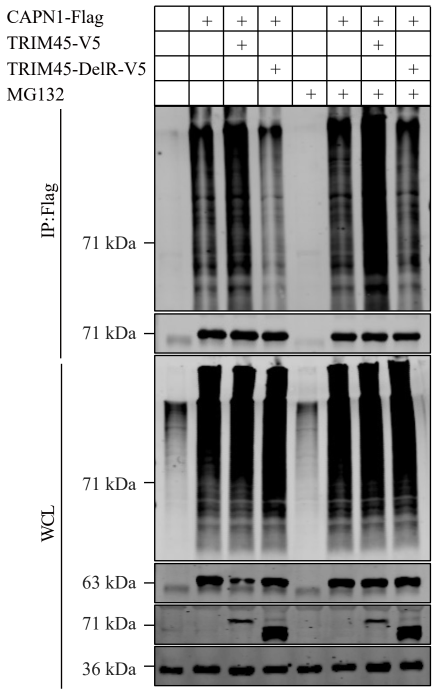
CAPN1-Flag
TRIM45-V5
TRIM45-DelR-V5
MG132
IP:Flag
WCL
71 kDa
71 kDa
71 kDa
63 kDa
71 kDa
36 kDa

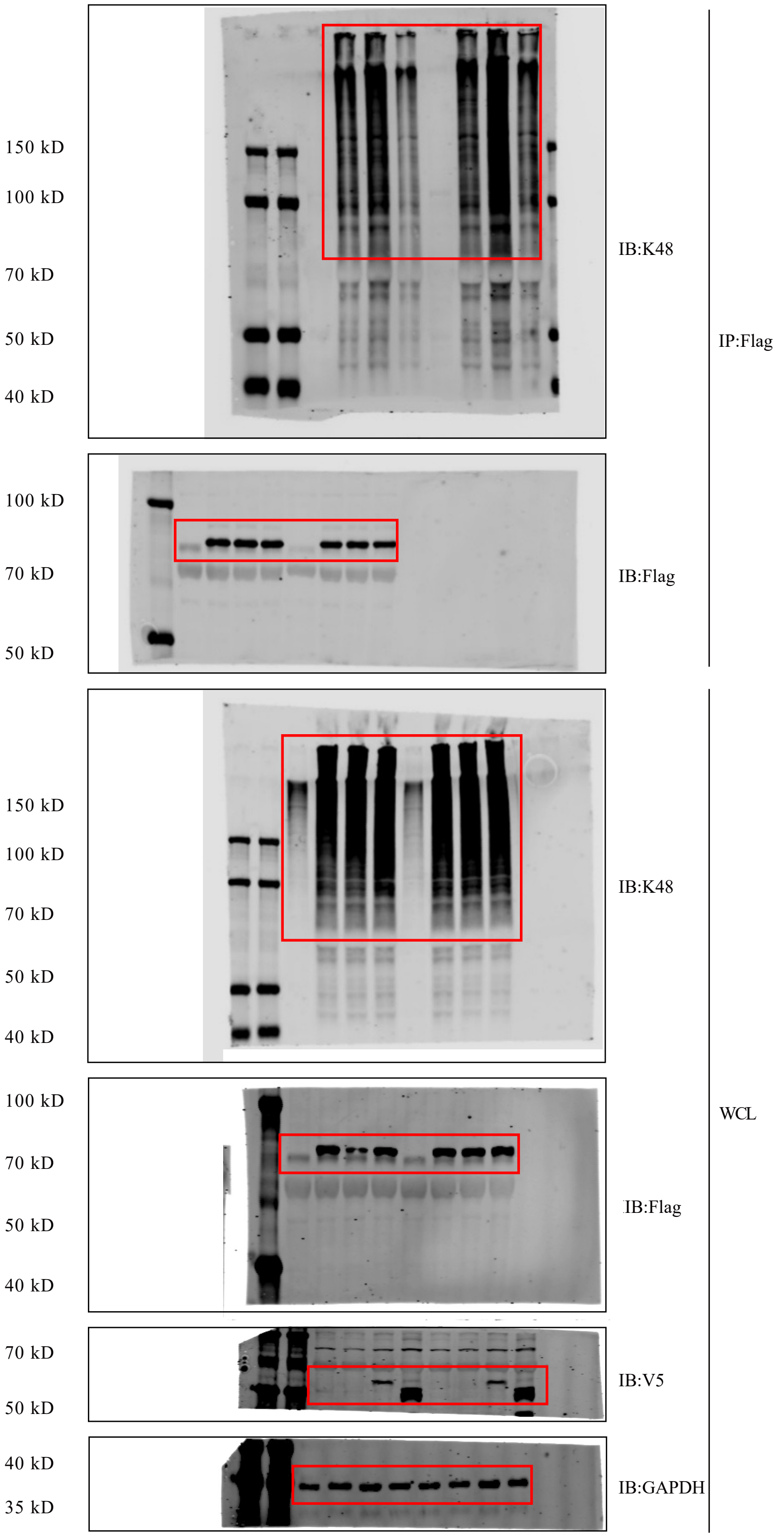
150 kD
100 kD
70 kD
50 kD
40 kD
IB:K48
IP:Flag
100 kD
70 kD
50 kD
IB:Flag
150 kD
100 kD
70 kD
50 kD
40 kD
IB:K48
100 kD
70 kD
50 kD
40 kD
IB:Flag
WCL
70 kD
50 kD
IB:V5
40 kD
35 kD
IB:GAPDH
